# C‑BODIPY Dyads Based on Fused-Ring Organoboron Scaffolds for Efficient Photodynamic Therapy Targeting *Candida albicans* Yeast and Biofilms

**DOI:** 10.1021/acs.jmedchem.6c00280

**Published:** 2026-06-01

**Authors:** Karolina A. Urbanowicz, Paulina H. Marek-Urban, Karolina Wrochna, Marta Rogalska, Anna Lewandowska-Andrałojć, Kamil Kotwica, Sandra Pluczyk-Małek, Agata Blacha-Grzechnik, Izabela Nasiłowska, Maciej Trzaskowski, Julia Gdesz, Jolanta Mierzejewska, Monika Staniszewska, Krzysztof Durka

**Affiliations:** † Faculty of Chemistry, 49566 Warsaw University of Technology, Noakowskiego 3, Warsaw 00-664, Poland; ‡ Faculty of Chemistry, Adam Mickiewicz University, Uniwersytetu Poznańskiego 8, Poznań 61-712, Poland; § Faculty of Chemistry, 49569 Silesian University of Technology, Strzody 9, Gliwice 44-100, Poland; ∥ Centre for Organic and Nanohybrid Electronics, 49569 Silesian University of Technology, Konarskiego 22B, Gliwice 44-100, Poland; ⊥ Centre for Advanced Materials and Technologies, 49566 Warsaw University of Technology, Poleczki 19, Warsaw 02-822, Poland; # Centre of Excellence for Health Technologies (HealthTEC), 49566 Warsaw University of Technology, Pl. Politechniki 1, Warsaw 00-661, Poland

## Abstract

A series of heavy-atom-free C-BODIPY photosensitizers based on five different types of fused-ring organoboron scaffolds was designed for the application in antifungal photodynamic therapy. The dyes were functionalized with a positively charged quaternary ammonium group or zwitterionic 3-(ethyldimethylammonio)­propane-1-sulfonate unit. Spectroscopic, electrochemical, and photocatalytic studies supported by DFT calculations revealed the crucial role of the organoboron structure on triplet-state generation. Most of the BODIPY complexes display high ROS generation with a type I/type II ROS ratio depending on the electronic structure of the boracycle unit. Antifungal studies demonstrated remarkable efficacy against the opportunistic yeast *Candida albicans* at low concentrations (1.6 μg/mL), achieving up to a 7-log reduction in the planktonic cells and significant photoactivity toward the fungal biofilm, reaching up to 4.3-log reduction in MFC values. The studies on the cell death mechanism revealed that cells undergo apoptosis upon photoinactivation. Furthermore, complexes were proven to reduce cell adhesion to biotic surfaces, preventing biofilm formation.

## Graphical abstract

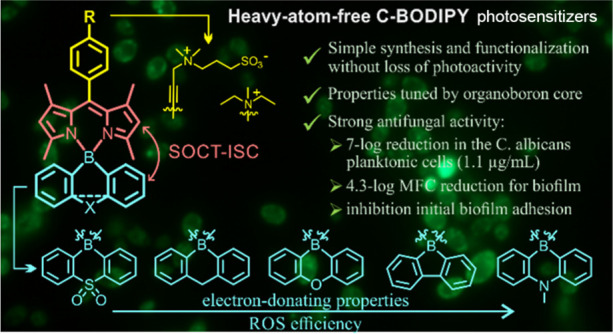

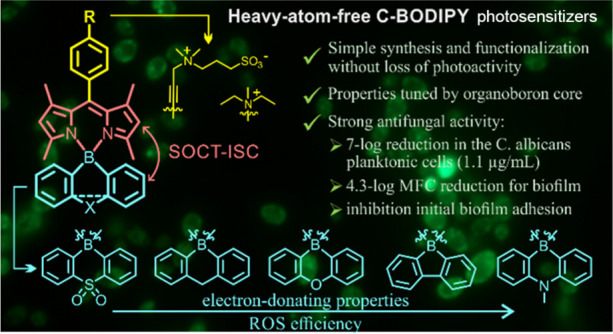

## 1 Introduction

Rising antimicrobial drug resistance is a critical threat to modern medicine.^1–4^ Among pathogenic fungi, *Candida albicans* is an opportunistic yeast that naturally inhabits the human microbiota.^5^ It resides commonly in the oral cavity, gastrointestinal tract, and urinary system.^6^ Although generally harmless, *C. albicans* can cause life-threatening systemic infections of the bloodstream (candidemia) and various organs (invasive candidiasis).^7^ Recent data indicate that invasive *C. albicans* infections are associated with a global mortality rate ranging from 20% to 50%.^8^ Despite its destructiveness to human health and rising antifungal drug resistance, for many years it remained underrated as a potential threat. A particular danger to the health system is the ability of *C. albicans* to form biofilms.^9–12^ A biofilm consists of microorganisms that adhere to a surface and are surrounded by an extracellular polymeric substance matrix. The cells might be attached to abiotic and biotic surfaces, such as medical devices, implants, teeth, or tissues. Cells that form biofilms differ significantly from their planktonic counterparts; they exhibit distinct phenotypes and gene expression, but most importantly, they are more resistant to external factors and drugs.^9,13,14^ The antimicrobial drug concentration required to kill microorganisms effectively is often higher than needed for planktonic cells, even up to 1000-fold.^15–18^ Such concentrations may exceed the dose that can be safely administered systemically. Therefore, research aimed at finding compounds and strategies against this pathogen should include studies on both planktonic cells and formed biofilms.

One promising alternative to conventional antifungal therapy is photodynamic therapy (PDT).^19–23^ It relies on three key elements—light, molecular oxygen, and a photosensitizer (PS). Upon light irradiation, the photosensitizer is excited to the singlet state and undergoes intersystem crossing (ISC) to the triplet state. Subsequently, the excited molecule can interact with molecular oxygen, leading to the generation of reactive oxygen species (ROS) as a result of either electron (I type ROS) or energy transfer (singlet oxygen, II type ROS).^24–27^ The resulting ROS induce oxidative damage to a wide range of biomolecules, including nucleic acids, proteins, and lipids leading to cell death.^28–32^

In this regard, boron dipyrromethene difluoride dyes (4,4-difluoro-4-bora-3*a*,4*a*-diaza-*s*-indacene, BODIPY) have emerged as effective photosensitizers for application in anticancer and antimicrobial PDT due to their favorable photophysical and chemical features, including simple synthesis and ease of postsynthetic structural modifications, strong visible-light absorption, good chemo- and photostability, and low threat to human health.^33–53^ The conventional and widely used approach to introduce photosensitizing properties in BODIPY relies on the introduction of heavy atoms.^35,43,54–59^ However, the presence of heavy atoms usually noticeably reduces hydrophilicity, photostability, and shortens the triplet state lifetimes.^60–64^ As such, heavy-atom-free BODIPYs are of significant interest. The recent approaches include the introduction of spin converting systems, exciton coupling in dimers, thieno-pyrrole-fused BODIPY, and compact orthogonal donor–acceptor architectures exhibiting efficient triplet state formation through a spin–orbit charge transfer intersystem crossing mechanism (SOCT-ISC).^65–80^ However, the rational design of heavy-atom-free BODIPY for PDT is still challenging, as it is difficult to achieve high cellular uptake and ROS production in a biological environment. In addition, the synthetic complexity of such photosensitizers and limited process scalability present significant barriers to their widespread use.

To address these challenges, our group has developed spirocyclic C-BODIPY dyads based on a 9-borafluorene (dibenzoborole) scaffold (Scheme 1) reaching a singlet oxygen generation quantum yield (QY^O^) of 78%.^81^ Notably, spirocyclic C-BODIPY dyes exhibit enhanced stability, attributed to their compact architectures effectively suppressing undesired reactions with ROS, which would lead to their degradation.^82^ Furthermore, the structures of the C-BODIPY can be easily modified at the *meso* position. For instance, we have obtained a borafluorene-BODIPY photosensitizer functionalized at the *meso*-position with a coumarin fragment (QY^O^ = 84% in DMF), acting simultaneously as a light-harvesting antenna and an endoplasmic reticulum-targeting group, which significantly increases the efficiency of PDT toward mammalian cells, while showing no cytotoxicity without light.^83^

**Scheme 1:**
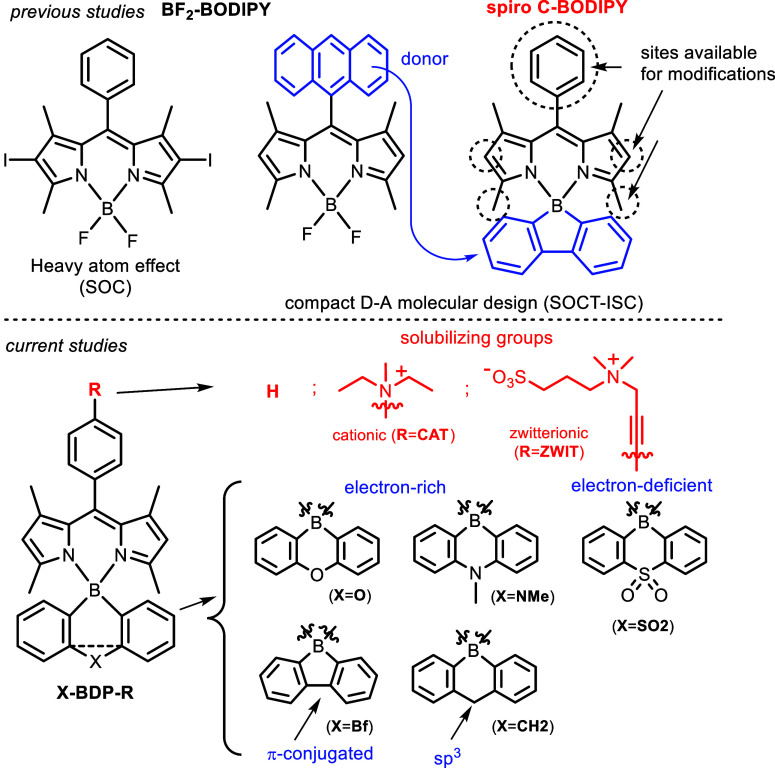
Scope of Studied C-BODIPY Complexes

At this stage, an important question emerges regarding the influence of the organoboron unit and the possibility of tuning the photoactivity and biological performance by modulating the boracyclic structure in C-BODIPY dyes. Consequently, in the current studies we have obtained C-BODIPY complexes based on five different ring-fused organoboron scaffolds varying in their inherent electronic properties, including electron-rich 5-methyldibenzo­[1,4]­azaborinine (**NMe**) and dibenzo­[1,4]­oxaborinine (**O**) systems, an electron-deficient dibenzo­[1,4]­thiaborinine dioxide (**SO2**) scaffold, as well as dibenzoborinine (**CH2**) and 5-membered dibenzoborole (borafluorene, **Bf**) cores. The latter two compounds represent the case of either C-sp^3^-separated (CH_2_ group) or π-conjugated two-aromatic ring systems, and both systems are considered as rather moderate electron donors. The obtained compounds were derivatized at the *meso* position with water-solubilizing groups (quaternary ammonium, **CAT**; and zwitterionic 3-(ethyldimethylammonio)­propane-1-sulfonate groups, **ZWIT**) to adjust their application for antimicrobial photodynamic therapy toward *C. albicans* and its formed biofilm. In total, we obtained 15 compounds abbreviated as **X-BDP-R** (Scheme 1), where **X** denotes the type of organoboron scaffold (**X** = **O**, **NMe**, **Bf**, **CH2**, and **SO2**), **BDP** stands for the dipyrromethene core, and **R** corresponds to the solubilizing group (**R** = H → **X-BDP**; **R** = **CAT** → **X-BDP-CAT**; **R** = **ZWIT** → **X-BDP-ZWIT**). These series were complemented by 3 referential difluoroboron BF_2_-BODIPY complexes (**BF2-BDP**, **BF2-BDP-CAT**, **BF2-BDP-ZWIT**), enabling direct comparison of photophysical effects resulted from the introduction of the organoboron unit.

## 2 Results and Discussion

### 2.1 Synthesis and Basic Characterization

The synthesis of C-BODIPY complexes assumes straightforward complexation of organoboron with dipyrromethene building blocks and further functionalization of the resulting C-BODIPY with water-solubilizing groups. The synthesis of the dipyrromethene (N^N) proligand involves classical condensation of benzaldehyde (or 4-iodobenzaldehyde, 4-*N*,*N*-diethylaminobenzaldehyde) with 2,4-dimethylpyrrole, followed by oxidation with DDQ (Scheme 2). The 6-membered dibenzo­[1,4]­(X)­borinines (X = NMe, CH_2_, SO_2_) were obtained from corresponding dibrominated precursors utilizing double Br/Li exchange followed by the borylation of dilithiated intermediate with BCl_2_N­(*i*-Pr)_2_ and acidic hydrolysis. Dibenzo­[1,4]­oxaborinine was prepared by double *ortho*-metalation of diphenylether with *n*-BuLi, borylation with BCl_2_N­(*i*-Pr)_2_, and acidic hydrolysis, while 9-chloroborafluorene was synthesized directly from 2,2′-dibromobiphenyl *via* a double Br/Li exchange reaction with *n*-BuLi and subsequent borylation with BCl_3_. The obtained dibenzoborininols (**X–OH**, Scheme 2) were converted to corresponding chlorides (**X–Cl**) by treatment with BCl_3_. It should be emphasized that direct borylation of dilithiated species with BCl_3_ is only applicable to 2,2′-dilithiobiphenyl. In the remaining cases, the reaction between the dilithium compound and BCl_3_ typically affords a mixture of products containing triarylboranes and arylboronic chlorides along with targeted dibenzoborinine chlorides.

**Scheme 2:**
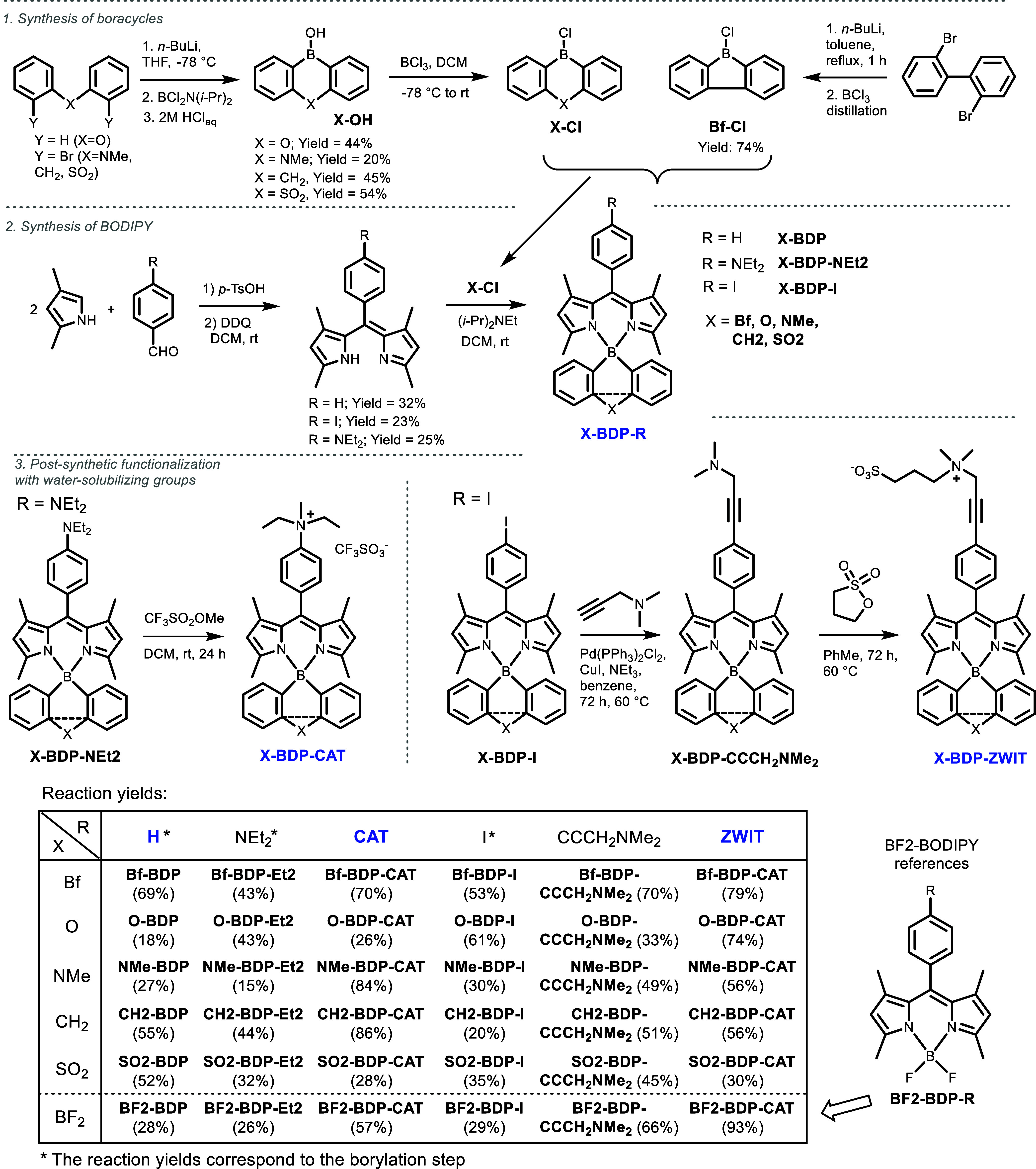
Synthetic Routes of C-BODIPY Dyes Carrying Various Boracyclic Units and Solubilizing Groups

As a result of the complexation of dipyrromethene and organoboron precursors, we have obtained three series of C-BODIPY complexes containing an unsubstituted phenyl group at the *meso* position (**X-BDP**); its derivative functionalized with an iodine atom (**X-BDP-I**) or a NEt_2_ group (**X-BDP-NEt2**). **X-BDP-NEt2** was further methylated with methyl triflate, giving rise to quaternary ammonium salts **X-BDP-CAT**. In turn, **X-BDP-I** was subjected to Sonogashira coupling with 3-dimethylamino-1-propyne. The alkylation of the dimethylamino group with 1,3-propane sultone led to the opening of the 5-membered sultone ring, affording a terminal SO_3_^–^ group in the zwitterionic C-BODIPY system **X-BDP-ZWIT**.

All final BODIPY complexes were purified by column chromatography, giving orange to red crystalline solids. The structures and the purity of all the products were confirmed by NMR spectroscopy, HR-MS spectrometry, elemental analysis (all compounds), and HPLC (most biologically active compounds). In addition, the molecular structures of **NMe-BDP-CAT** (Figure 1) and **BF2-BDP-CAT** (Figure S1, Supporting Information) were confirmed by X-ray diffraction experiments. The molecular geometry of **NMe-BDP-CAT** is consistent with other *spiro* C-BODIPY complexes showing near to orthogonal alignment of dibenzoborinine and dipyrromethene molecular units.^81–84^

**Figure 1:**
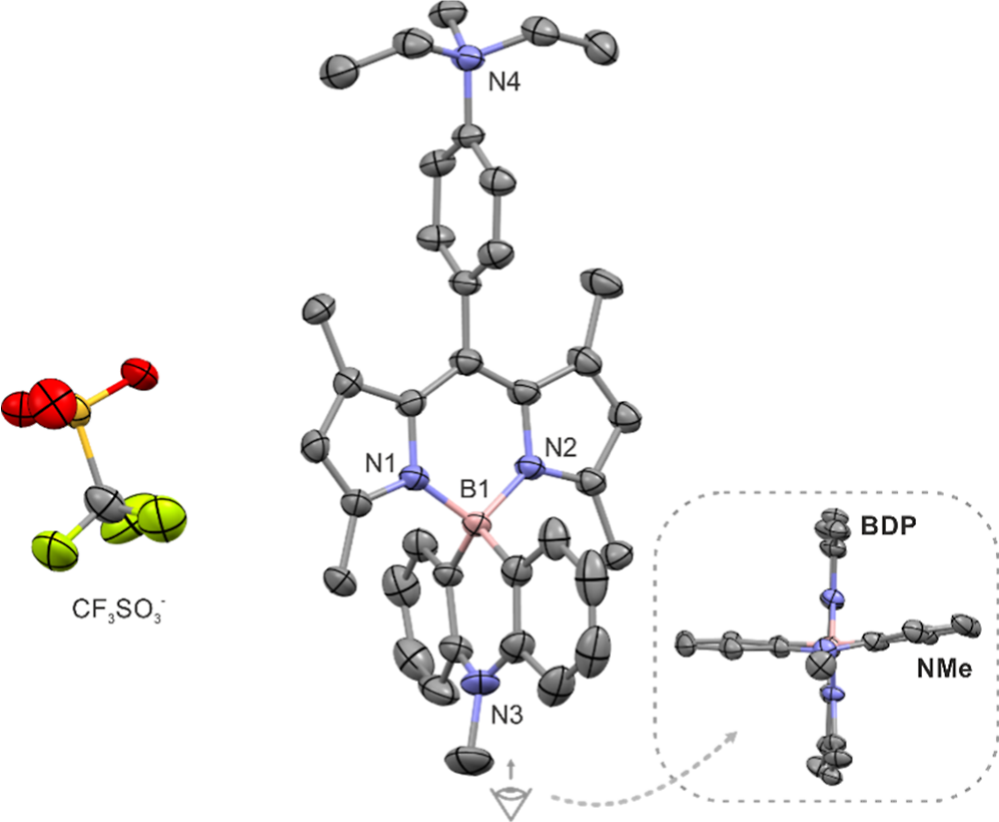
Molecular structure of **NMe-BDP-CAT**. Thermal motions are shown as atomic displacement parameters (ADPs) at the 50% probability level. The hydrogen atoms and CHCl_3_ solvent molecules were omitted for clarity.

### 2.2 Steady-State UV–Vis Spectroscopy and Photostability

The spectroscopic properties of obtained compounds were first determined in diluted (10^–5^ M) CHCl_3_ solutions, and the results are summarized in Table 1. The absorption maxima for complexes are comparable, ranging from 500 to 508 nm (Figures S2–S4, Supporting Information), with high molar absorption coefficients ranging from 61,200 to 109,000 dm^3^·mol^–1^·cm^–1^. Similarly, the emissions λ_max_ do not differ significantly between complexes (508–525 nm, Figures S6–S8, Supporting Information), giving a small separation between absorption and emission bands (Stokes shifts of 280–670 cm^–1^). The fluorescence lifetimes vary in a narrow range of several nanoseconds (1.10–5.09 ns, Figures S12–S14, Supporting Information). These characteristics are consistent with the typical difluoroboron BODIPY dyes.^85^ On the other hand, the significant drop in the fluorescence quantum yields are observed (QY^F^ in range 1–43%). In particular, derivatives based on the dibenzo­[1,4]­azaborinine scaffold (**NMe-BDP-R**) show negligible emission. This suggests the presence of an alternative deexcitation pathway in C-BODIPY, most likely through triplet states. In contrast, **SO2-BDP-R** derivatives display the highest fluorescence intensities among studied C-BODIPY, which, as will be demonstrated later, goes in pair with their lowest triplet state generation efficiency. Finally, the introduction of water-solubilizing groups causes only minor shifts in the absorption and emission bands, but leads to further decrease in fluorescence intensities. Subsequent measurements in water/DMSO (1:1 v/v) solutions of the cationic **X-BDP-CAT** and zwitterion **Bf-DP-ZWIT** compounds revealed that their spectroscopic properties are not dependent on solvent polarity (Figures S5, S9 and S15, Supporting Information). The absorption and emission bands exhibited only minor hypsochromic shifts, accompanied by a slight increase in fluorescence quantum yield (Table 1—values in brackets).

**Table 1:** Summary of Photophysical Data for Studied Compounds in Diluted (*c* = 10^–5^ M) CHCl_3_ Solutions^j^

	λ_abs_ ^a^/nm	ε·10^–4^ ^b^/dm^3^·mol^–1^·cm^–1^	λ_em_ ^c^/nm	Δ^d^/ cm^–1^	τ^e^/ns	QY^F^ ^f^/%	τ_1/2_(deg)^g^/h
**Bf-BDP**	501	8.65	512	430	1.37	20	8.8
**O-BDP**	501	8.81	508	280	2.28	39	11.0
**NMe-BDP**	501	7.43	515	560	-^h^	<1	9.8
**CH2-BDP**	501	9.67	515	560	1.87	37	13.5
**SO2-BDP**	500	7.15	513	510	2.89	43	23.4
**BF2-BDP**	502	10.9	516	540	2.51	73	5.5
**Bf-BDP-ZWIT**	506 (502)	-^i^(6.64)	523 (517)	660 (580)	3.89 (1.49)	11 (14)	2.8 (35 min)
**O-BDP-ZWIT**	504	-^i^	521	670	1.10	15	5.0
**NMe-BDP-ZWIT**	504	-^i^	519	590	-^h^	<1	3.4
**CH2-BDP-ZWIT**	502	-^i^	518	620	4.78	17	2.1
**SO2-BDP-ZWIT**	503	-^i^	517	560	1.47	21	3.3
**BF2-BDP-ZWIT**	506	-^i^	522	630	2.05	33	3.2
**Bf-BDP-CAT**	507 (503)	6.56 (7.30)	525 (518)	660 (580)	5.09 (2.16)	16 (24)	16.1 (27 min)
**O-BDP-CAT**	506 (501)	6.12 (7.48)	521 (515)	590 (540)	1.31 (3.34)	28 (33)	33.8 (84 min)
**NMe-BDP-CAT**	505 (501)	8.75 (6.89)	520 (514)	570 (500)	-^h^	<1 (<1)	16.5 (29 min)
**CH2-BDP-CAT**	506 (502)	8.38 (6.96)	522 (515)	650 (500)	1.35 (2.98)	18 (30)	9.2 (48 min)
**SO2-BDP-CAT**	505 (501)	-^i^(6.45)	520 (515)	570 (540)	1.72 (4.43)	38 (49)	10.1 (59 min)
**BF2-BDP-CAT**	508 (503)	7.43 (8.13)	522 (518)	550 (580)	2.44 (4.80)	46 (47)	18.4 (21 min)

a Absorption maximum.

b Molar absorption coefficient.

c Emission maximum, excitation wavelength λ_ex_ = 470 nm.

d Stokes shift.

e Fluorescence lifetimes recorded with TCSPC (λ_ex_ = 340 nm, EPLED laser).

f Fluorescence quantum yields.

g Photodegradation half-lives were determined at the maximum absorption wavelength based on the drop in absorbance. The values in parentheses were given for measurements in 20% (v/v) serum in water.

h The emission lifetime was not measured because of the negligible fluorescence of the sample.

i The compounds were characterized by very low solubility in CHCl_3_, and therefore, the determination of the molar absorption coefficient produces large errors.

j Values in brackets represent the measurements in water/DMSO (1/1 v/v) solutions.

The obtained complexes show long-term stability under ambient conditions, both in the solid state and organic solutions. The hydrolytic and photochemical stabilities were investigated by monitoring changes in absorption for the samples kept in the dark or irradiated with neutral-white light (40 mW·cm^–2^). Both **X-BDP** and **X-BDP-CAT** remained stable in the diluted solution and exhibited high resistance to light-induced degradation and ROS (Table 1 and Figures S16–S33, Supporting Information). In particular, the **O-BDP-R** shows exceptional hydrolytic and photochemical stability with photodegradation half-life (τ_1/2_(deg)) of 33 h. The dyes with zwitterionic functionalization slowly hydrolyze in the absence of light and are generally more prone to photochemical oxidation as their half-lives range from 2.1 h for **CH2-BDP-ZWIT** to 5.0 h for **O-BDP-ZWIT** (Figures S28–S33, Supporting Information).

The photostability of the compounds decreases in the water environment. The irradiation experiments performed in 20% (v/v) serum (FBS) in water showed the photodegradation half-lives varying in the range of 21–84 min (Table 1 and Figures S34–S40, Supporting Information). Interestingly, the difluoroboron reference system **BF2-BDP-CAT** was the least stable, while also exhibiting low efficiency of ROS generation in aqueous and fungal environments. The functionalization at the boron atom improves photostability, reaching τ_1/2_(deg) = 84 min for **O-BDP-CAT**. It should be noted that such degree of photostability is sufficient for very effective photoinactivation of *C. albicans* and its formed biofilm (see Biological section). Moreover, such moderate stability in aqueous solutions can be advantageous, as it prevents accumulation in the environment or higher organisms, limiting prolonged ROS production and unwanted phototoxicity effects in daylight.

### 2.3 Electrochemical Studies

Redox potentials of **X-BDP** complexes determined using cyclic voltammetry (CV) and differential pulse voltammetry (DPV) in CH_2_Cl_2_ are reported with respect to the Fc/Fc^+^ redox couple (Table 2 and Figures S43–S48, Supporting Information). All complexes apart from **NMe-BDP** display reversible (or quasi-reversible) oxidation and reduction waves. The reduction potentials (*E*_1/2_^red^) are generally similar, spanning in the range between −1.91 V and −1.97 V, and only deviate for **SO2-BDP** (*E*_1/2_^red^ = −1.80 V), reflecting electron-accepting properties of the SO_2_ group in dibenzo­[1,4]­thiaborinine dioxide core. All obtained reduction potentials are generally lower as compared to the respective value determined for difluoroboron BF_2_-BODIPY (*E*_1/2_^red^ = −1.69 V), indicating that the replacement of classical fluoride substituents with organic groups decreases the electron affinity of BODIPY complexes. This finding is consistent with elevated LUMO levels of these compounds as evidenced from DFT calculations (vide infra).

**Table 2:** Redox Potentials and Gibbs Free Energy Change for PET in **X-BDP** Complexes Based on Electrochemical (Given with Respect to the Fc/Fc^+^ Redox Couple) and Spectroscopic Measurements

compound	*E*_1/2_^ox^ ^a^/V	*E*_1/2_^red^ ^b^/V	*E*_0,0_ ^c^/eV	*r* ^d^/Å	*E*_col_ ^e^/eV	Δ*G*_PET_ ^f^/eV
**BF2-BDP**	0.73	–1.69	-	-	-	-
**Bf-BDP**	0.57	–1.93	2.46	3.57	–0.44	–0.40
**O-BDP**	0.60	–1.91	2.46	3.39	–0.47	–0.42
**NMe-BDP**	0.32/0.76	–1.96	2.44	3.34	–0.47	–0.65
**CH2-BDP**	0.55	–1.97	2.45	3.31	–0.48	–0.41
**SO2-BDP**	0.71	–1.80	-^g^	-^g^	-^g^	-^g^

a Half-wave oxidation potential.ik.

b Half-wave reduction potential.

c Energy of S_0_(v = 0)→S_1_(v = 0) transition obtained from the intersection of normalized absorption and emission spectra.

d Distance between centroids of boracycle and BDP (geometries were obtained from DFT calculations).

e Coulombic interaction between positive and negative charges located on boracycle and BDP, respectively.

f Gibbs free energy changes for photoinduced electron transfer.

g Values were not calculated as this system is not a D–A type of molecule due to the low donor properties of the SO2 core.

More significant differences are observed in the oxidation potentials. The oxidation potential values of **O-BDP**, **Bf-BDP**, and **CH2-BDP** cover a rather narrow range of 0.55 V (**CH2-BDP**) to 0.60 V (**O-BDP**). This process can be associated with the oxidation of the organoboron unit. The presence of an electron-accepting group in **SO2-BDP** makes the oxidation of this molecule more difficult, which is manifested by an increase of its oxidation potential to *E*_1/2_^ox^ = 0.71 V. This value is similar to the oxidation potential of **BF2-BDP** (*E*_1/2_^ox^ = 0.73 V), indicating that for both compounds the oxidation occurs at the BDP ligand frame. In contrast, in the case of **NMe-BDP**, two oxidation waves are observed. The first process at *E*_1/2_^ox1^ = 0.32 V can be attributed to the oxidation of the amine part of the molecule to a radical cation, while the second oxidation at *E*_1/2_^ox2^ = 0.76 V corresponds to the BDP core.

The Gibbs free energy changes for photoinduced electron transfer (Δ*G*_PET_) were calculated based on the Rehm–Weller equation for **X-BDP** compounds except for **SO2-BDP** as the electron-donating abilities of the dibenzothia­[1,4]­thiaborinine dioxide core are insufficient to induce PET. The negative Δ*G* values suggest that the PET would be thermodynamically favorable for all these C-BODIPY (Table 2), in particular for **NMe-BDP**, which displays the most negative Δ*G*_PET_.

### 2.4 Nanosecond Transient Absorption Spectroscopy

To directly probe the triplet excited-state properties of **X-BDP**, nanosecond transient absorption spectra were recorded following laser excitation at 480 nm (Figures 2 and S10, Supporting Information). Upon excitation, all of the investigated compounds exhibited ground-state bleaching at 500 nm. This spectral feature matched well with the maximum band position observed in the steady-state absorption spectrum of **X-BDP**.

**Figure 2:**
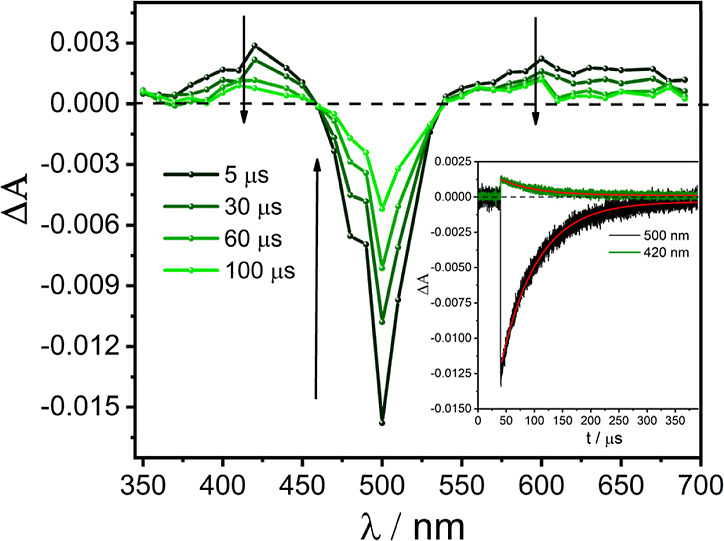
Transient absorption spectra obtained during laser flash photolysis (with excitation at 480 nm) of deoxygenated solutions of **NMe-BDP** in chloroform; time delay after flash from 5 to 100 μs. Inset: decay profiles monitored at 500 nm (black) and 420 nm (green) with monoexponential decay fits (red lines).

Triplet-state absorption was observed as a weak band with a maximum at approximately 420 nm, together with a broad, weak band in the 550–650 nm region. The triplet absorption spectra were consistent with those reported in the literature for BODIPY functionalized with a phenoxazine moiety at the *meso*-carbon atom.^86^ The intensity of the transient absorption signal was the highest for **NMe-BDP** and the lowest for **SO2-BDP**. Considering that all samples had the same absorbance at the excitation wavelength (0.50) and were excited using the same laser power, it is reasonable to assume that the transient absorption signal intensity reflects the efficiency of the intersystem crossing. The strongest signal observed for **NMe-BDP** is consistent with its highest singlet-oxygen generation efficiency.

The triplet lifetime was determined from monoexponential fits of both the transient decay at 420 nm and the bleach recovery at 500 nm (inset in Figures 2 and S11, Supporting Information). In each case, both kinetic traces were characterized by the same time constant (Table 3). Furthermore, a pronounced decrease in the triplet lifetime was observed in the presence of oxygen, indicating that the triplet state of all **X-BDP** derivatives is capable of reacting with molecular oxygen.

**Table 3:** Triplet State Lifetimes for **X-BDP** Compounds in Deoxygenetead and Air-Saturated Solutions Measured with Nanosecond Transient Absorption Spectroscopy

	τ _T_ ^a^/μs	τ _T_ ^b^/ns
**Bf-BDP**	110	455
**O-BDP**	117	493
**NMe-BDP**	66	406
**CH2-BDP**	166	460
**SO2-BDP**	179	-^c^

a Triplet lifetimes for deoxygenated samples obtained from monoexponential decay fits of kinetic profiles at 500 nm after 480 nm laser excitation.

b Triplet lifetimes for air-saturated samples were obtained from monoexponential decay fits of kinetic profiles at 500 nm after 480 nm laser excitation.

c Not determined because of low signal intensity.

### 2.5 Photogeneration of ROS

The ROS generation performance of all studied BODIPY photosensitizers was first investigated in CHCl_3_ solutions. The formation of singlet oxygen was confirmed by the observation of ^1^O_2_ phosphorescence at 1270 nm (Figure S41, Supporting Information) and EPR spectroscopy using 2,2,6,6-tetramethylpiperidine (TEMP) as a ^1^O_2_ trap (Figure S42, Supporting Information). To establish the efficiency of ROS production, 2-furoic acid (2-FA) was selected as a model reductant oxidized with *in situ*-generated ROS to 5-hydroxyfuran-2­(5*H*)-one (Figure 3a). The BODIPY’s solutions in CHCl_3_ were irradiated in a cylindrical photoreactor equipped with a neutral-white LED light source (40 mW·cm^–2^) matching BODIPY excitation range, and chosen for its ubiquity and accessibility. The photosensitizer loading was only 0.05%_mol_ with respect to the starting 2-FA and the conversion was monitored by ^1^H NMR. The reaction profiles are presented in Figure 3b–d and turnover frequencies (TOF) are collected in Table 4.

**Figure 3:**
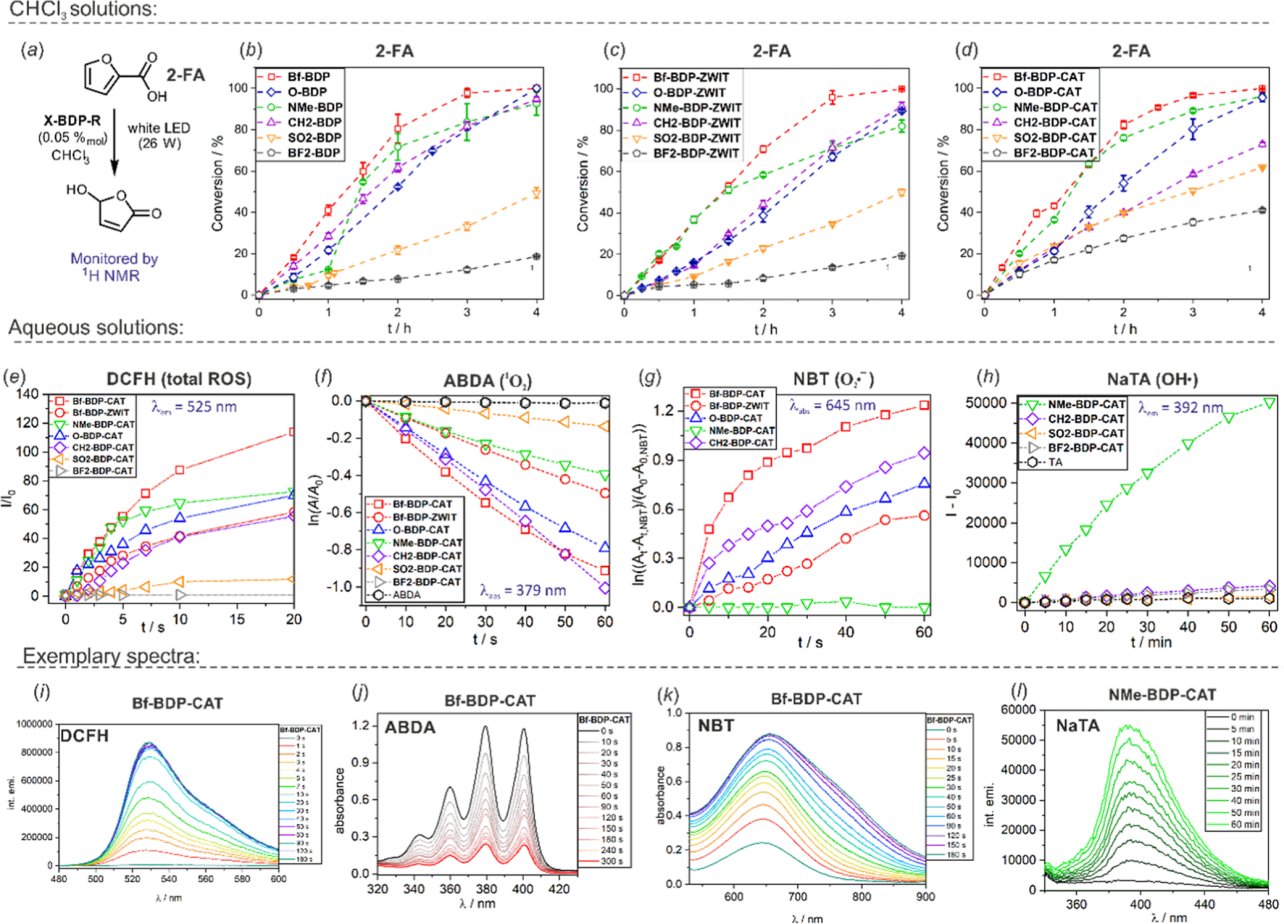
(a) Photocatalytic oxidation of 2-furoic acid and reaction profiles for (b) **X-BDP**, (c) **X-BDP-ZWIT**, and (d) **X-BDP-CAT**. Photocatalytic experiments in aqueous media with ROS scavengers: (e) DCFH (total ROS fluorescent probe), (f) ABDA (^1^O_2_ UV–vis probe), (g) NBT (O_2_^•–^ UV–vis probe), and (h) NaTA (OH^•^ fluorescent probe). Exemplary spectra for experiments with ROS scavengers: (i) DCFH, (j) ABDA, (k) NBT, and (l) NaTA. The remaining spectra are presented in Figures S51–S54 in the Supporting Information. All experiments were performed at room temperature under irradiation with 26 W neutral-white LED light (40 mW·cm^–2^).

**Table 4:** Summary of ROS Generation Experiments in CHCl_3_

	TOF (2-FA)^a^/h^–1^	QY^O^ (DPBF)^b^/%	I^x^/I^r^ (NIR)^c^
**Bf-BDP**	800	78 ^81^	1.00
**O-BDP**	530	81	0.81
**NMe-BDP**	720	90	2.31
**CH2-BDP**	620	78	0.86
**SO2-BDP**	230	12	0.32
**BF2-BDP**	80	-^d^	-^d^
**Bf-BDP-ZWIT**	710	55	0.52
**O-BDP-ZWIT**	490	51	0.35
**NMe-BDP-ZWIT**	580	42	0.87
**CH2-BDP-ZWIT**	440	22	0.51
**SO2-BDP-ZWIT**	230	22	0.34
**BF2-BDP-ZWIT**	80	-^d^	-^d^
**Bf-BDP-CAT**	820	64	1.07
**O-BDP-CAT**	540	44	0.45
**NMe-BDP-CAT**	750	63	0.48
**CH2-BDP-CAT**	400	41	0.98
**SO2-BDP-CAT**	400	44	0.40
**BF2-BDP-CAT**	270	-^d^	-^d^

a Turnover frequency for photocatalytic conversion of 2-furoic acid with BODIPY photocatalyst (0.05%_mol_ with respect to substrate), calculated after 2 h of irradiation with white light.

b Quantum yield of photogeneration of singlet oxygen measured using diphenylisobenzofuran (DPBF) as the ^1^O_2_ trap (diagnostic band λ = 414 nm), excited with Oxxius diode laser 505 nm. The values were calculated with respect to **Bf-BDP**.^81^

c Relative integrals of singlet oxygen phosphorescence (I^x^) from spectroscopic measurements in the NIR region, given with respect to **Bf-BDP** (I^r^).

d The signal was not distinguished from the background.

All studied C-BODIPYs demonstrate significantly higher photoactivity compared to difluoroboron BF_2_-BODIPY. Most C-BODIPY complexes reach >80% conversion of the 2-FA within 4 h (TOF in the range of 440–800 h^–1^), with the exception of **SO2-BDP-R**, which exhibits generally lower photoactivity with TOF in between 230 and 400 h^–1^. From the studied group of C-BODIPYs, the most active are **Bf-BDP-R** and **NMe-BDP-R**. The introduction of a solubilizing group has a rather small impact on the photocatalytic properties of C-BODIPY, with **X-BDP-ZWIT** having generally slightly lower and **X-BDP-CAT** slightly higher photoactivity with respect to corresponding **X-BDP**. Interestingly, the difluoroboron **BF2-BDP-CAT** complex shows enhanced activity (TOF = 270 h^–1^) with respect to **BF2-BDP** and **BF2-BDP-ZWIT** analogues (TOF = 80 h^–1^). We suppose that the generation of triplet states in cationic **X-BDP-CAT** may also involve an alternative SOCT-ISC pathway with oxidative PET (electron transfer to the *meso* CAT moiety), analogous to that postulated for difluoroboron BODIPY with an electron-accepting pyridinium moiety at the *meso* position;^87^ however, more research is required to study this effect.

In the following step, the singlet oxygen quantum yield (QY^O^, Table 4) was studied by monitoring the drop in the intensity of the absorption band (414 nm) of diphenylisobenzofuran (DPBF). For the **X-BDP** series, the highest QY^O^ value of 90% was observed for **NMe-BDP**. The **O-BDP** (QY^O^ = 81%) and **CH2-BDP** (QY^O^ = 78%) were also very active, whereas **SO2-BDP** (QY^O^ = 12%) showed decreased singlet oxygen production. Overall, these values are comparable to the best performing iodinated or brominated BODIPY, where SOC is strongly enhanced by the heavy-atom effect.^42,49,88^ For the cationic group of BODIPY, the highest QY^O^ values were recorded for **NMe-BDP-CAT** (QY^O^ = 63%) and **Bf-BDP-CAT** (QY^O^ = 64%), while for the remaining systems they range from 41 to 44%. Interestingly, **SO2-BDP-CAT** showed improved activity compared to unmodified system **SO2-BDP**; this generally agrees with its higher photocatalytic activity toward 2-FA. In the group of **X-BDP-ZWIT**, **Bf-BDP-ZWIT** and **O-BDP-ZWIT** were the most active compounds with QY^O^ reaching 55% and 51%, respectively, **NMe-BDP-ZWIT** showed slightly lower activity (QY^O^ = 42%), while **CH2-BDP-ZWIT** and **SO2-BDP-ZWIT** were the least active (QY^O^ = 22%).

The comprehensive comparison between **X-BDP**, **X-BDP-ZWIT**, and **X-BDP-CAT** reveal interesting differences in their photocatalytic behavior in organic solutions. **X-BDP-ZWIT** and **X-BDP-CAT** are less efficient photocatalysts in DPBF oxidation than corresponding compounds from **X-BDP** series, while exhibiting similar photoactivity toward 2-FA. These discrepancies can be explained by the varying sensitivities of both probes to different types of generated ROS. DPBF, commonly used as a singlet oxygen probe, can in fact also react with the superoxide radical anion (O_2_^•–^).^89,90^ Similarly, 2-FA can react with singlet oxygen, superoxide radical ion and hydroxyl radical (OH^•^), in a similar way as furfuryl alcohol.^91,92^ Based on the comparison of ^1^O_2_ phosphorescence signal integrals (*I* ^x^/*I* ^r^ values in Table 4), **NMe-BDP** produces singlet oxygen 2.3 times more effectively than **Bf-BDP**. Simultaneously, the reactivities of both compounds toward 2-FA and DPBF are comparable. Furthermore, the NIR signal intensity for **NMe-BDP-CAT** is about half that of **Bf-BDP-CAT**, despite their similar photoactivity in 2-FA and DPBF oxidation. Similar conclusions can be derived from the comparison of **CH2-BDP-CAT** and **SO2-BDP-CAT**, which show comparable photocatalytic activity toward 2-FA and DPBF, but from NIR analysis it stems that the latter system produces singlet oxygen less efficiently. This indicates that other ROS types contribute to the process, and the type I/II ROS ratio presumably depends on both the structure of the organoboron unit and the nature of the pendant groups.

To further verify this hypothesis, we examined the type I and type II ROS generation in aqueous media for the **X-BDP-CAT** group and **Bf-BDP-ZWIT** selected as most photobioactive compounds. First the 2′,7′-dichlorodihydrofluorescein (DCFH) was employed as the total ROS indicator. The formation of the fluorescent product was monitored by recording emission spectra in the range of 480–600 nm (Figures 3i and S51, Supporting Information). The efficiency of total ROS generation follow the order **Bf-BDP-CAT** > **NMe-BDP-CAT** > **O-BDP-CAT** > **Bf-BDP-ZWIT** = **CH2-BDP-CAT**, while **SO2-BDP-CAT** and difluoroboron complex **BF2-BDP-CAT** showed negligible photoactivity (Figure 3e). Next, we studied singlet oxygen production in water using 9,10-anthracenediylbis­(methylene)­dimalonic acid (ABDA), monitored by the drop in absorbance at 379 nm (Figures 3f,j and S52, Supporting Information). According to our results, **Bf-BDP-CAT**, **CH2-BDP-CAT**, and **O-BDP-CAT** exhibited the highest singlet oxygen generation efficiency, while **NMe-BDP-CAT** and **Bf-BDP-ZWIT** were less effective. These results show that the solubilizing group also influences the type of ROS produced, as the unsubstituted **NMe-BDP** was the most efficient singlet oxygen generator in the **X-BDP** series.

Finally, the production of O_2_^•–^ and OH^•^ was evaluated by employing I type ROS scavengers: nitro blue tetrazolium (NBT) and disodium terephthalate terephthalic acid (NaTA). NBT reacts with the superoxide radical anion under basic conditions to form formazan, which shows a distinct absorption band at 645 nm. NaTA reacts with the hydroxyl radical to produce disodium 2-hydroxyterephthalate, which can be tracked by monitoring its emission at 392 nm. The experiments revealed that **Bf-BDP-CAT** generates O_2_^•–^ most efficiently, followed by **CH2-BDP-CAT** > **O-BDP-CAT** > **Bf-BDP-ZWIT** (Figures 3g,k and S54, Supporting Information). **NMe-BDP-CAT** does not produce O_2_^•–^, but this is compensated by its very high generation of OH^•^ (Figures 3h,l and S53, Supporting Information). This contrasts with the other tested complexes, for which the hydroxyl radical yield was negligible, although the results for **Bf-BDP-ZWIT**, **Bf-BDP-CAT** and **Bf-BDP-O** are not clear due to some unspecified side reaction between photosensitizers and scavenger. Overall, in aqueous media, **Bf-BDP-CAT** generates ROS most effectively, with strong contributions from ^1^O_2_ and O_2_^•–^, **NMe-BDP-CAT** primarily produces OH^•^ with some ^1^O_2_, and the remaining compounds generate ^1^O_2_ and O_2_^•–^ in varying ratios, with the exception of **SO2B-BDP-CAT**, which is generally characterized by low activity.

### 2.6 Quantum-Chemical Calculations

To rationalize the differences in observed photocatalytic activity of C-BODIPY complexes, we have performed series of DFT calculations at the B3LYP/6-311++G­(d,p) level of theory. First, we found that the type of a boracyclic fragment only slightly affects the electron density at the dipyrromethene core. This is reflected in the similar Hirshfeld atomic charges of pyrrole ring carbon atoms (Tables S2–S6, Supporting Information), and nearly unchanged chemical shifts of the pyrrole C–H protons in the ^1^H NMR spectra, oscillating close to 5.80 ppm.

For boracyclic **X-BDP** compounds, the LUMO orbital is localized on the dipyrromethene moiety (Figures S55–S57, Supporting Information), with energy remaining nearly constant *c.a.* – 2.38 eV (Figure S58, Supporting Information) except for **SO2-BDP** where it decreases to −2.68 eV (Figure 4a)—note that this is consistent with its less negative oxidative potential derived from electrochemical studies. For **NMe-BDP** and **O-BDP**, the HOMO is localized on the organoboron fragment, with orbital energies of −4.86 eV and −5.47 eV, respectively, whereas in **Bf-BDP** HOMO is also partially spread on BDP (−5.43 eV). In contrast, for **CH2-BDP** and **SO2-BDP**, both HOMO and LUMO reside on the dipyrromethene unit, whereas HOMO-1 localizes on dibenzoborinine (**CH2-BDP**) or is delocalized on entire molecule (**SO2-BDP**). Cationic and zwitterionic functional groups generally do not significantly alter orbital localization. The only difference occurs for **Bf-BDP-ZWIT**, where HOMO localizes mostly on borafluorene, and **O-BDP-ZWIT**, where HOMO resides on BDP and HOMO-1 on dibenzo­[1,4]­oxaborinine fragments. In cationic species, the LUMO levels are naturally lowered, resulting in a decreased HOMO–LUMO energy gap by 0.08–0.15 eV. In the case of zwitterionic compounds, both HOMO and LUMO energies are systematically increased by 0.23–0.30 eV, leaving the HOMO–LUMO gap essentially unaffected (Figures S58–S60, Supporting Information).

**Figure 4:**
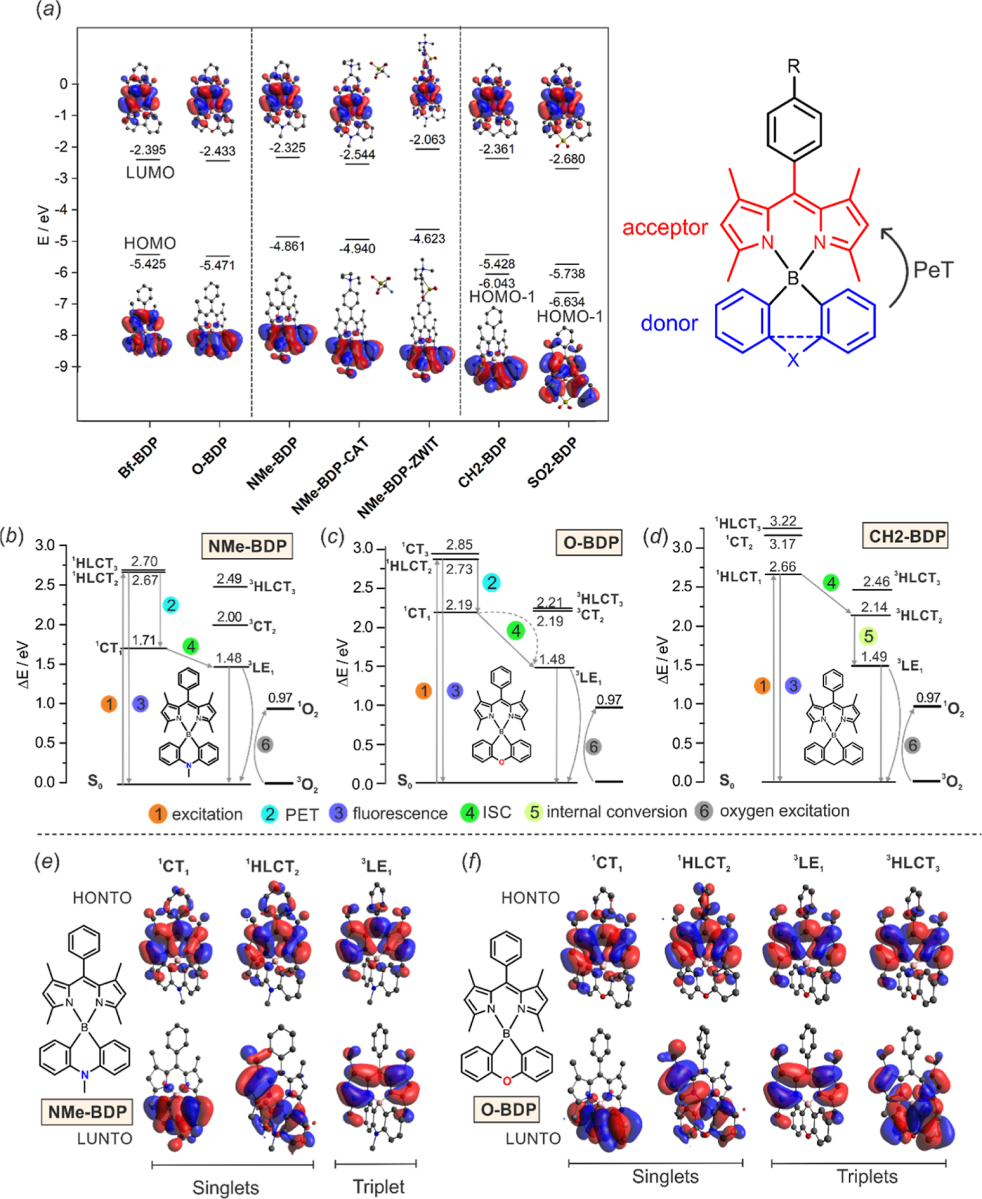
(a) Molecular orbital diagram in **X-BDP** along with **NMe-BDP-CAT** and **NMe-BDP-ZWIT** selected as representative compounds for cationic and zwitterionic C-BODIPY, respectively. Energy diagram demonstrating the photophysical processes in (b) **NMe-BDP,** (c) **O-BDP,** and (d) **CH2-BDP**. NTO orbitals for singlet and triplet states of (e) **NMe-BDP** and (f) **O-BDP**. The molecular orbitals and electronic energy diagrams for the remaining compounds are shown in the Supporting Information (Figures S55–S65).

In the subsequent step, we have calculated the singlet and triplet energy levels using TD-DFT (CAM-B3LYP/6-311++G­(d,p)). The excited-state energy levels were calculated for less structurally complex series of *spiro* **X-BDP**. In addition, natural transition orbital (NTO) analysis was used to characterize the nature of the excited states (Figures S61–S65, Supporting Information), whereas the spin–orbit coupling matrix elements (SOCME) were calculated to reflect the rate of ISC (Table 5). In all studied cases, the lowest energy triplet state is confined on the dipyrromethene moiety (^3^LE_1_ state, LE = local excitation) and it is unaffected by the boracyclic unit, with an energy of ∼1.48 eV (Figure 4e,f). This is in close agreement with values reported for related C-BODIPY photosensitizers.^82^ In **Bf-BDP**, **O-BDP,** and **NMe-BDP**, the lowest singlet excited state is of charge transfer character (^1^CT_1_) with near-zero oscillator strength, while the second singlet state is hybrid local-charge transfer (^1^HLCT_2_) with significant oscillator strength and energy of ∼2.65 eV. Consequently, the electron excitation proceeds to the ^1^HLCT_2_ state, which is followed by PET to more stable ^1^CT_1_. Due to the perpendicular orientation of the boracycle and dipyrromethene, the intersystem crossing from ^1^CT_1_ to ^3^LE_1_ can be expected to be in agreement with the SOCT-ISC mechanism. However, only in the case of **NMe-BDP,** the ^1^CT_1_-^3^LE_1_ energy gap value is small (0.23 eV). The calculated ^1^CT_1_-^3^LE_1_ SOCME value for **NMe-BDP** is 0.95 cm^–1^, which, combined with small energy gap, facilitates direct SOCT-ISC (Figure 4b). In contrast, **Bf-BDP** and **O-BDP** exhibit larger ^1^CT_1_-^3^LE_1_ energy gaps of 0.69 and 0.71 eV, respectively, with SOCME values of *c.a.* 0.51 cm^–1^. On the other hand, the energy gap between ^1^CT_1_ and higher-energy triplet state (^3^HLCT_2_ for **Bf-BDP** or ^3^HLCT_3_ for **O-BDP**) is very small (0.12 eV for **Bf-BDP** and 0.01 eV for **O-BDP**), and the corresponding SOCME values are large (1.27 cm^–1^ for **Bf-BDP** and 2.84 cm^–1^ for **O-BDP**), indicating that the intersystem crossing is likely mediated by a higher-level triplet state (Figure 4c). In case of **CH2-BDP**, the ^1^CT_2_ level is above the ^1^HLCT_1_, potentially reducing ISC efficiency, although it should also be noted that charge transfer states are generally stabilized by the interaction with a polar solvent. Thus, SOCT-ISC is expected to remain operative for this compound in a polar environment. Alternatively, ISC may occur between the ^1^HLCT_1_ and ^1^HLCT_2_ levels, as suggested by the large SOCME value (1.20 cm^–1^), followed by internal conversion to ^3^LE_1_ (Figure 4d). In **SO2-BDP** the strong electron-accepting properties of SO_2_ group elevates the CT state far above the singlet LE and HLCT states precluding SOCT-ISC. Finally it should be noted that for all studied compounds except **SO2-BDP**, the SOCME between ^3^LE_1_ and ground S_0_ state are very small, which is beneficial for their long triplet-state lifetimes.

**Table 5:** Calculated SOCME (cm^–1^), Singlet and Triplet Energy Gap (eV) Values in **X-BDP** ^b^

compound	S_1_-T_1_	S_1_-T_2_	S_1_-T_3_	T_1_-S_0_	Δ*E*_S1‑T1_	Δ*E*_S1‑T2_	Δ*E*_S1‑T3_
**Bf-BDP**	0.51	1.27	0.00	0.07	0.69	0.12	-^a^
**O-BDP**	0.52	0.00	2.84	0.07	0.71	0.01	-^a^
**NMe-BDP**	0.95	0.05	3.47	0.12	0.23	-^a^	-^a^
**CH2-BDP**	0.01	1.20	0.00	0.01	1.17	0.51	0.20
**SO2-BDP**	0.12	0.30	0.06	0.54	1.46	0.65	0.42

a *E*_T_ > *E*_S_.

b The transitions most likely responsible for ISC are bolded.

### 2.7 Biological Studies – Initial Screening

The applicability of the obtained photosensitizers in photodynamic therapy against *C. albicans* was investigated. Fluorescence microscopy images of *C. albicans* ATCC 10231 cells stained with BODIPY complexes demonstrated that the molecules can effectively penetrate the yeast cell wall, confirming efficient cellular uptake (Figures 5 and S66, Supporting Information). Costaining experiments with DAPI excluded the dye accumulation inside the cell nucleus and indicated its localization in the cytoplasm, most likely around the vacuoles.

**Figure 5:**
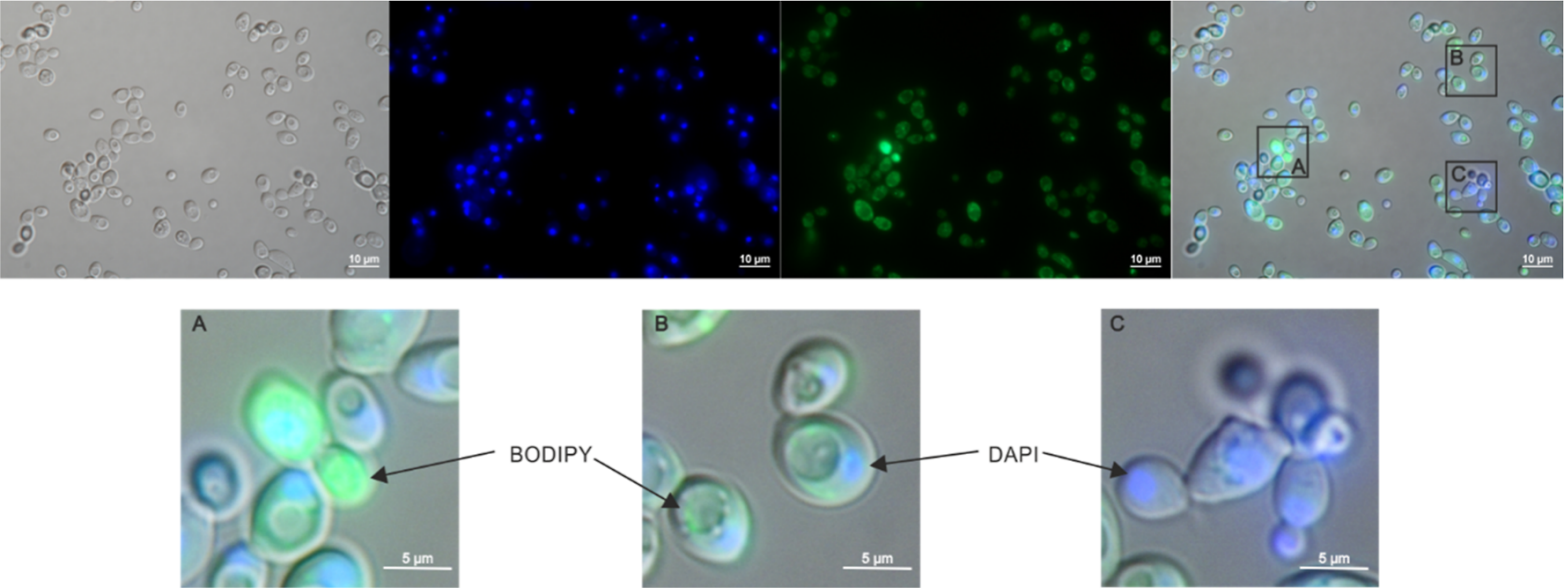
Fluorescence microscopy images of *C. albicans* costained with DAPI and **O-BDP-CAT** (6.3 μM). From left: images taken in white light, DAPI fluorescence, BODIPY fluorescence, and merged images. The fluorescence microscopy images for **Bf-BDP-ZWIT**, **Bf-BDP-CAT**, **CH2-BDP-CAT**, and **BF2-BDP-CAT** (DAPI fluorescence, BODIPY fluorescence and merged images) are presented in Figure S66, Supporting Information.

After confirming that the BODIPY effectively penetrated the cells, their photodynamic effect on *C. albicans* was evaluated under light irradiation. A flat-panel irradiation device equipped with neutral-white 26 W LED light source was used (Figure S50, Supporting Information). The experiments were performed on 24- and 96-well plates to ensure consistent and effective exposure to light. The irradiation duration was 1 h. The distance between the plates and the planar light source during irradiation was approximately 10 cm (40 mW·cm^–2^). Parallel biological experiments were conducted in the absence of light to determine the dark anti-*Candida* action. We have also confirmed that the irradiation does not affect the yeast cells in the absence of BODIPY. Yeast viability under both irradiation and dark conditions was initially assessed by measuring the optical density of the yeast suspension at OD_600_. In addition, the solution of the photosensitizer without cell suspension was used as a reference to ensure that the absorption of the compound did not artificially inflate the results.

Initially, the efficiency of the C-BODIPY photosensitizers was investigated using 25 μM concentrations of the dyes (Figure 6). Despite their high photocatalytic activity in organic solutions, the BODIPY lacking hydrophilic functionalities (**X-BDP**) were completely ineffective in a biological environment. This is due to their poor solubility in aqueous media and negligible penetration through the fungal cell wall. In contrast, significant photoactivity was observed for dyes bearing cationic or zwitterionic substituents. Specifically, a pronounced reduction in cell viability (<1% survival rate) was observed for **Bf-BDP-CAT**, **O-BDP-CAT**, **NMe-BDP-CAT**, **CH2-BDP-CAT**, **BF2-BDP-CAT**, and **Bf-BDP-ZWIT** upon irradiation, based on optical density measured at an OD_600_ value relative to the untreated control. It should be noted that **Bf-BDP-CAT** and **O-BDP-CAT** in 25 μM concentration show considerable dark anti-*Candida* action. The generally higher activity of cationic BODIPY dyes can be attributed to their enhanced ability to penetrate the fungal cell wall.^93,94^ In turn, the zwitterionic dyes exhibit lower solubility in an aqueous environment compared to their cationic counterparts, although **Bf-BDP-ZWIT** showed very high photoactivity combined with low dark anti-*Candida* action.

**Figure 6:**
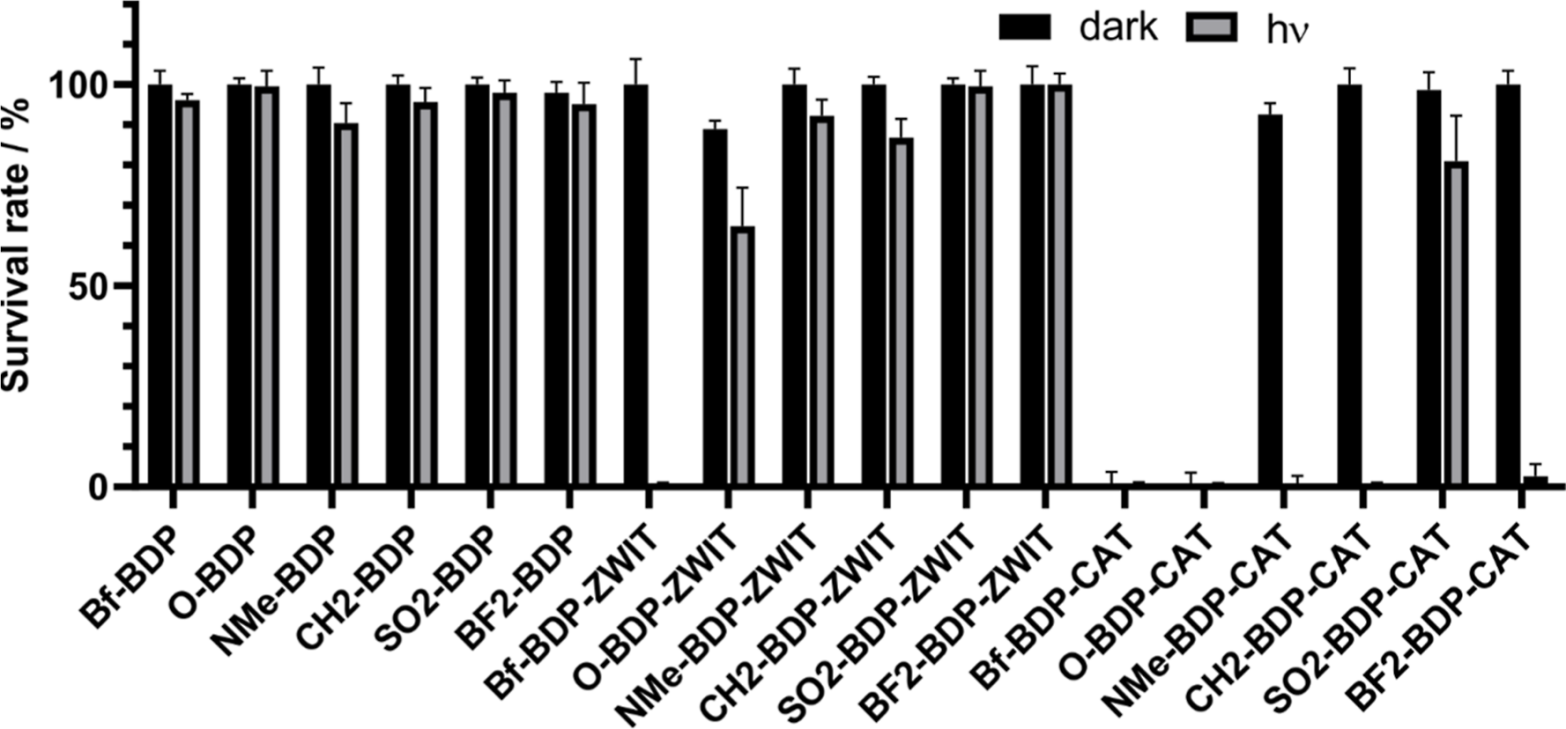
Results of the PDT studies against *C. albicans* using BODIPY complexes at a 25 μM concentration. The bars represent the means of four independent repetitions.

### 2.8 PDT Studies against *C. albicans* MFC Determination and the Cell Death Mechanism

In the next phase, we focused on the group of most phototoxic BODIPY complexes: **Bf-BDP-CAT**, **O-BDP-CAT**, **NMe-BDP-CAT**, **CH**_2_ **-BDP-CAT**, and **Bf-BDP-ZWIT**. The group was complemented by **BF2-BDP-CAT**, serving as a reference for C-BODIPY, and also demonstrated improved photocatalytic performance compared to the two remaining BF_2_-BODIPY. The concentration dependence was evaluated over the range of 0.05–25 μM. For each BODIPY, the 2–4 lowest concentration values were selected, corresponding to survival rates of less than 20% under irradiation and negligible dark anti-*Candida* activity. MFC values were established by assessing the log reduction of *C*. *albicans via* colony-forming unit (CFU) enumeration on agar plates.

The studied compounds demonstrated very high photoactivity toward planktonic *C. albicans*, resulting in a 7-log reduction in cell counts, corresponding to a survival rate of less than 0.000001% (Figure 7 andTable [Table tbl6]). The MFC values are remarkably low, ranging from 1.6 μM for **Bf-BDP-CAT**, **NMe-BDP-CAT**, and **CH2-BDP-CAT** to 6.3 μM for **O-BDP-CAT** and **Bf-BDP-ZWIT**. By comparison, the reference **BF2-BDP-CAT** system displayed an MFC of 12.5 μM. It should be noted that despite its higher MFC value, **O-BDP-CAT** still achieved significant log reductions of 3.21 and 2.68 at 3.2 μM and 1.6 μM, respectively. Importantly, no cytotoxic effect was observed in the absence of irradiation.

**Figure 7:**
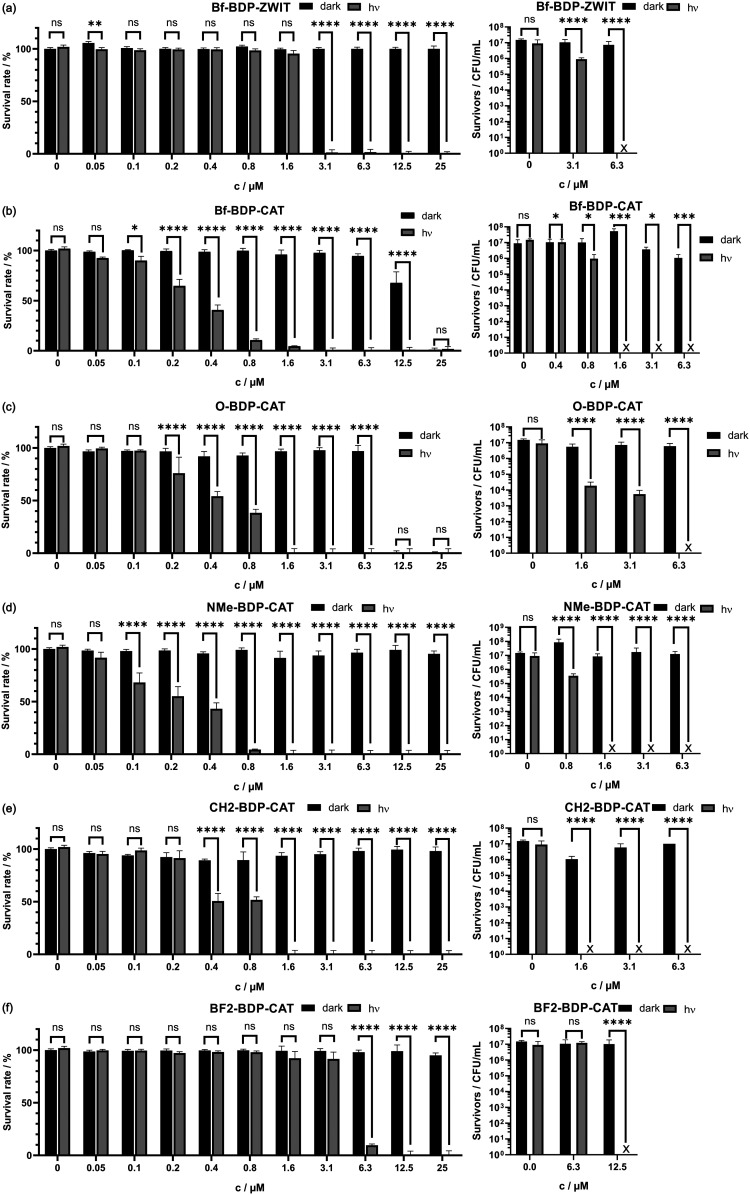
Results of PDT studies against *C. albicans* with (a) **Bf-BDP-ZWIT**, (b**) Bf-BDP-CAT**, (c) **O-BDP-CAT**, (d) **NMe-BDP-CAT**, (e) **CH2-BDP-CAT**, and (f) **BF2-BDP-CAT** photosensitizers, at concentrations ranging from 0 to 25 μM expressed as the mean percentage survival rate (based on 4 independent repetitions), calculated from OD_600_ values relative to the nonirradiated control. Graphs illustrating CFU/mL reduction at the selected, most effective concentrations based on grown colonies on agar plates (based on 4 independent repetitions) after their incubation at 37 °C for 24 h are presented in the left column. The “X” on the graph means that the growth of *C. albicans* was not observed even for the nondiluted cell suspension after treatment. *p < 0.05, **p < 0.01, ***p < 0.001, ****p < 0.0001, ^ns^ *p* > 0.05 compared with the dark control at the same concentration.

**Table 6:** MFC Values for the Reduction of Planktonic *C. albicans* Cells under Exposure to Irradiation for 1 h (26 W, Neutral-White Light)

compound	MFC/μM	MFC/μg mL^–1^
**Bf-BDP-CAT**	1.6	1.08
**O-BDP-CAT**	6.3	4.34
**NMe-BDP-CAT**	1.6	1.12
**CH2-BDP-CAT**	1.6	1.10
**BF2-BDP-CAT**	12.5	6.99
**Bf-BDP-ZWIT**	6.3	4.04

Overall, the developed heavy atom-free C-BODIPY dyes show an excellent photodynamic therapeutic effect, outperforming most known halogenated (Br, I) or heavy-atom free BODIPY photosensitizers, typically reaching 2–6 log reduction of *C. albicans* under irradiation at concentrations higher than 5–10 μM.^35,37,55,88,95–99^ Aforementioned activity is achieved without additives (e.g., KI),^100^ or it is not induced by other effects such as aggregation-induced ROS generation in AIE-based phototherapeutic agents.^101–103^ Our results position C-BODIPY among the most active photosensitizers reported to date, such as diiodinated BODIPY functionalized with charged 3-pyridinium moiety at the *meso* position, which reach the MFC values of 0.63–1.25 μM with 3.8–6.7 log reduction in viability of *C. albicans* cells upon irradiation at 90 mW·cm^–2^ for 15 min^55^

To confirm that the studied photosensitizers induce oxidative stress within the fungal cells, we performed an assay with CellRox Deep Red Reagent (oxidative stress indicator). Representative images (for **Bf-BDP-ZWIT** at its MFC) from CLSM (confocal laser scanning microscopy) are shown in Figure 8. Images for the **X-BDP-CAT**, along with positive and negative controls, are presented in the Supporting Information (Figures S74–S80). Conducted studies confirm that the high antifungal activity of the compounds is indeed a result of the generated ROS upon exposure to the visible light, as indicated by the bright-red fluorescence resulting from the oxidized form of the oxidative stress indicator (Figure 8a). Simultaneously, the compounds do not induce oxidative stress in the absence of light (Figure 8b).

**Figure 8:**
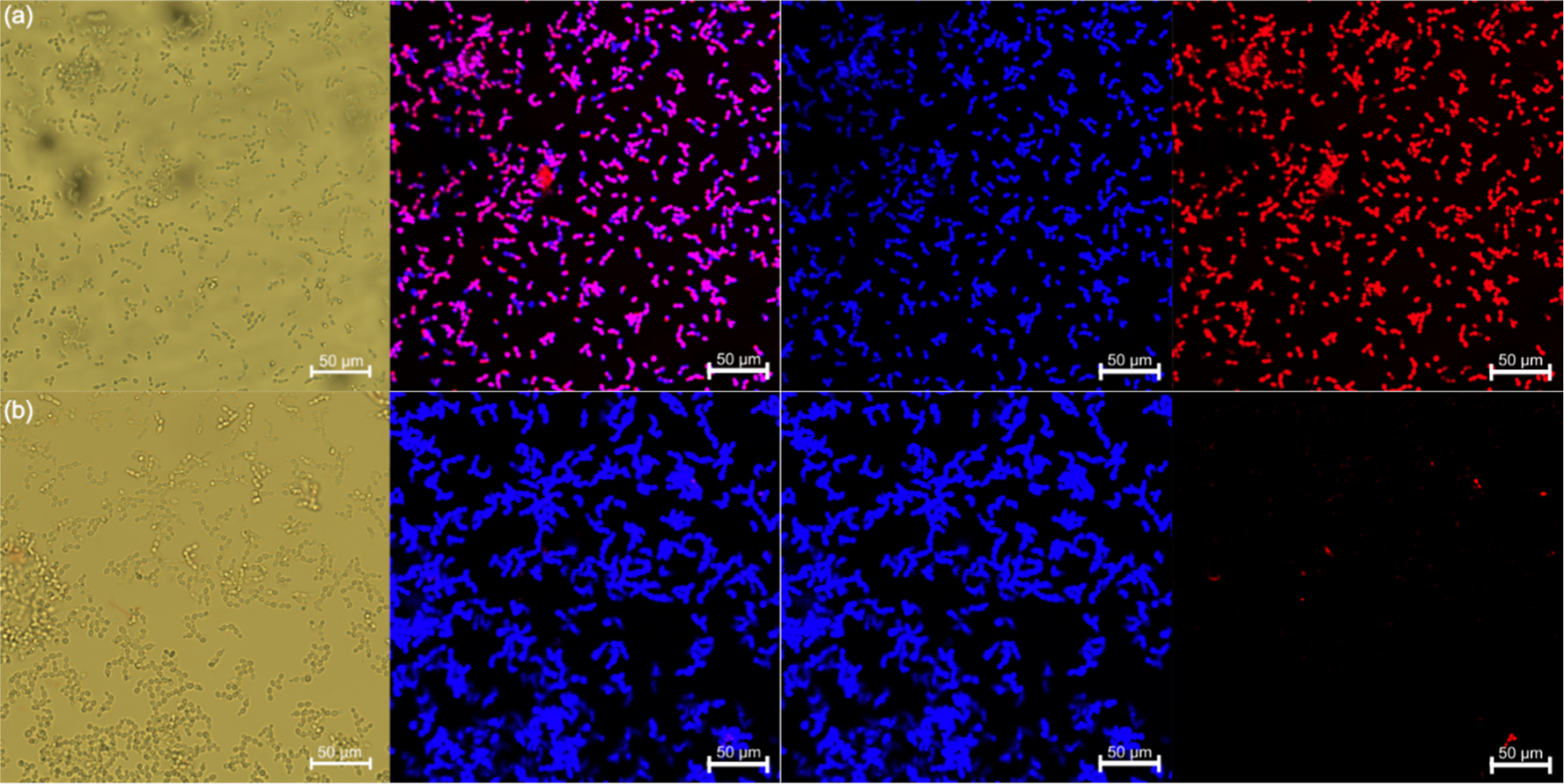
CLSM images demonstrating the oxidative stress induced with PDT in the presence of the **Bf-BDP-ZWIT** photosensitizer. (a) Treated and irradiated cells, (b) treated cells incubated in the absence of light. From the left: cells in the bright field, merged fluorescence images, cells stained with calcofluor white, cells stained with CellRox Deep Red Reagent.

To further investigate the impact of the exposure of the BODIPY photosensitizer upon light irradiation on *C. albicans* cells, the experiments in sub-MFC concentrations were performed, followed by staining the cells with Calcofluor White, Hoechst 33342, and propidium iodine (PI). The images from CLSM for **O-BDP-CAT** (3.1 μM) are presented in Figure 9. The images for **Bf-BDP-ZWIT** and **X-BDP-CAT** are presented in the Supporting Information (Figures S67–S74, Supporting Information). Calcofluor White staining of the cell wall is comparable in samples exposed and nonexposed to light, indicating that the fungal cell wall is not the main target of the studied complexes. Moreover, there is no overlap between Calcofluor White and photosensitizer fluorescence, confirming that the compounds do not localize within its structure. Similarly, the Hoechst 33342 fluorescence is similar in the studied conditions, which suggests that no significant nuclear damage occurred. Additionally, no noticeable differences in PI staining were observed between irradiated and nonirradiated samples, indicating that in sub-MFC concentrations, the compounds inhibit fungal growth, without substantially increasing the membrane permeabilization significantly.

**Figure 9:**
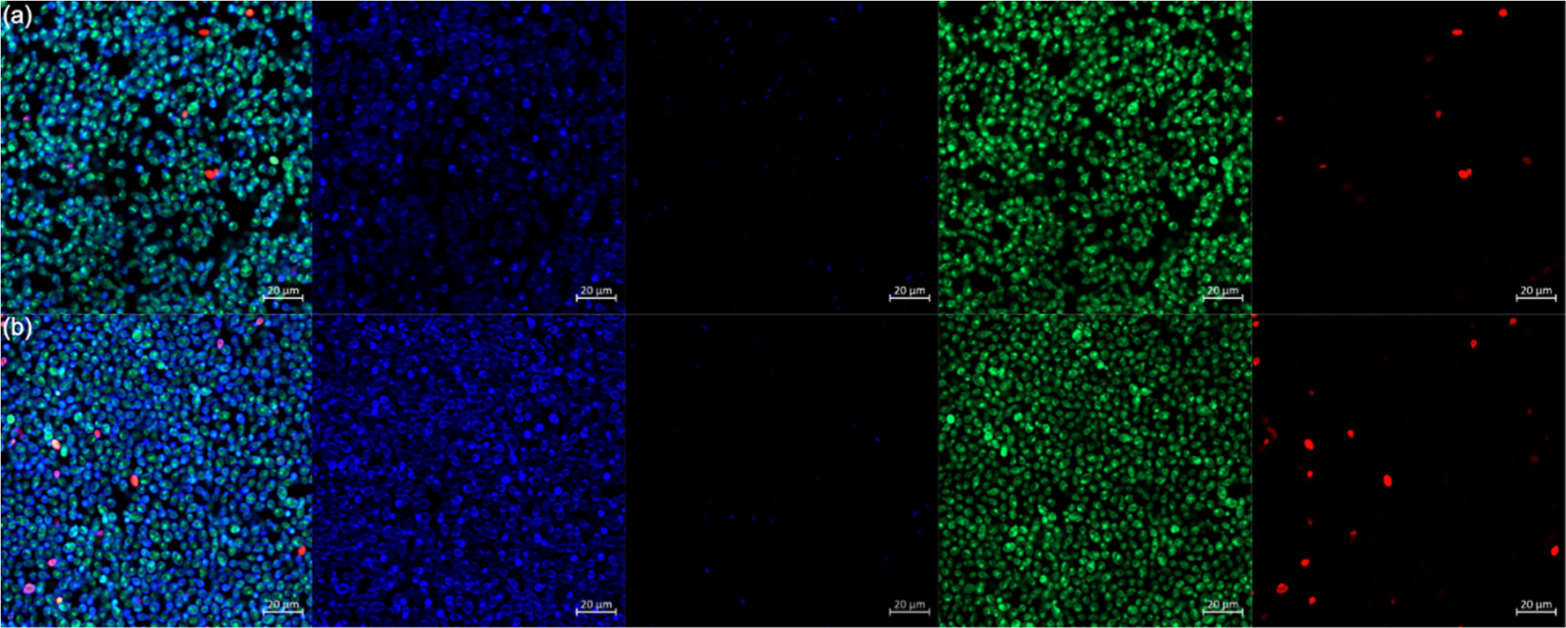
Images from CLSM for **O-BDP-CAT** dye (3.1 μM): (a) treated cells after exposure to light, (b) treated cells incubated in the absence of light. From left: merged fluorescence photos, calcofluor white fluorescence, Hoechst 33342 fluorescence, BODIPY fluorescence, PI fluorescence.

In the subsequent step, the cell death mechanism of *C. albicans* SC5314 planktonic cells after photoinactivation treatment were studied, and the results are demonstrated in Figure 10. The studies were performed at the determined MFC concentrations. The measurements of treated and untreated (control) cells after exposure to light with a flow cytometer demonstrated that for all studied compounds, the vast majority of cells (>90%) were in the early apoptotic stage. A fraction of cells were in the late apoptotic stage, with the highest cell number of 5.85% observed for **O-BDP-CAT**. No viable cells were distinguished, except for the control, or they were present at very low levels (less than 0.1%). There were no necrotic cells observed, indicating that the cells treated with the studied photosensitizers after irradiation underwent apoptosis.

**Figure 10:**
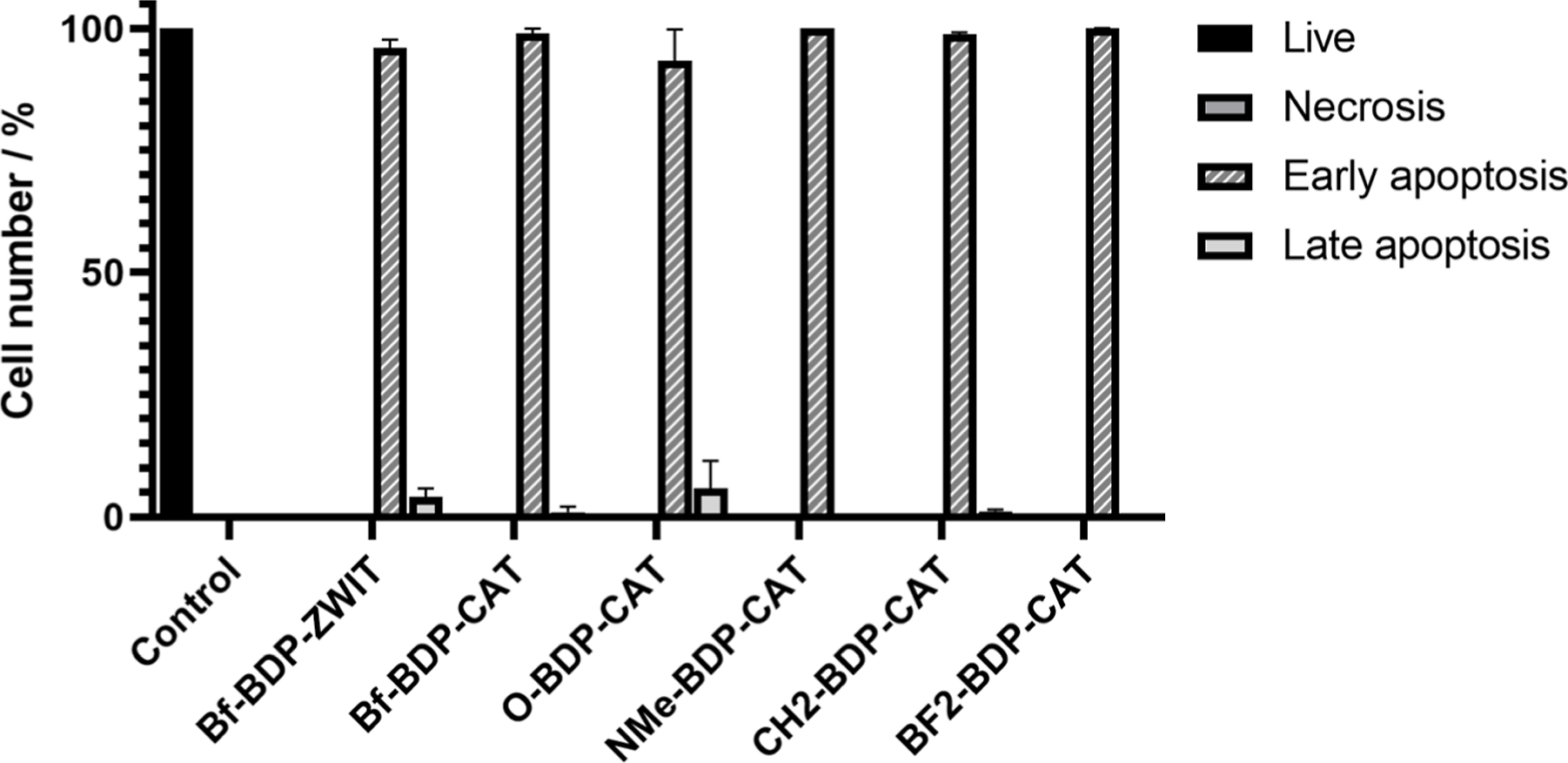
Effects of studied photosensitizers on cell death after photoinactivation of planktonic *C. albicans* SC5314 cells. After treatment, test samples and untreated cells (control) were stained with propidium iodine (PI) and Annexin-V. The staining pattern was described as follows: live cells were Annexin-V-negative and PI-negative. Necrotic cells were Annexin-V-negative and PI-positive. Early apoptotic cells were Annexin-V-positive and PI-negative, whereas late apoptotic cells were positive for both Annexin-V and PI. Concentrations: **Bf-BDP-ZWIT** (6.3 μM), **Bf-BDP-CAT** (1.6 μM), **O-BDP-CAT** (6.3 μM), **NMe-BDP-CAT** (1.6 μM), **CH2-BDP-CAT** (1.6 μM), **BF2-BDP-CAT** (12.5 μM).

### 2.9 Photoactivity toward the *C. albicans* Biofilm

The PDT activity of **Bf-BDP-ZWIT**, **Bf-BDP-CAT**, **O-BDP-CAT**, **NMe-BDP-CAT**, **CH2-BDP-CAT**, and **BF2-BDP-CAT** was studied against the biofilm formed by yeast cells.^10^ For these studies, *C. albicans* SC5314 ATCC was selected because of its robust ability to form biofilms.^104^ Photoinactivation experiments were conducted on mature biofilms cultured on a biotic surface consisting of a monolayer of L929 mouse fibroblasts. The complexes were tested at MFC concentrations determined for planktonic cells of *C. albicans* (Figure 11).

**Figure 11:**
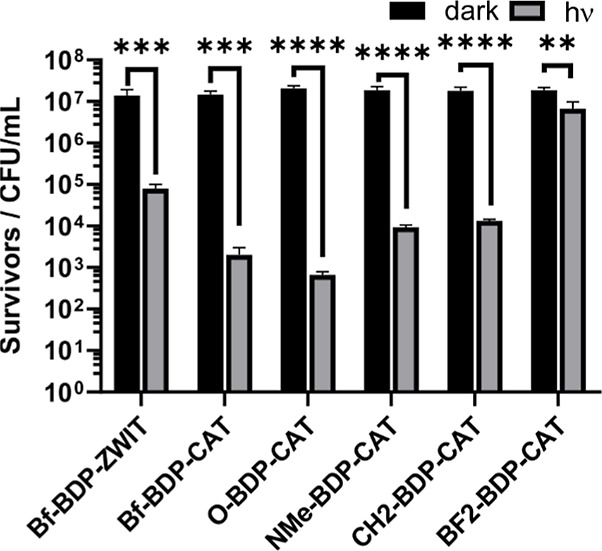
Results of BODIPY dye photoactivity against the *C. albicans* biofilm. Concentrations: **Bf-BDP-ZWIT** (6.3 μM), **Bf-BDP-CAT** (1.6 μM), **O-BDP-CAT** (6.3 μM), **NMe-BDP-CAT (**1.6 μM), **CH2-BDP-CAT** (1.6 μM), **BF2-BDP-CAT** (12.5 μM). *p < 0.05, **p < 0.01, ***p < 0.001, ****p < 0.0001, ^ns^ *p* > 0.05 compared with the dark control at the same concentration.

The obtained results showed that C-BODIPY complexes exhibited significant photoactivity toward the biofilm formed by *C. albicans* cells. The highest biofilm photoinactivation was observed for **O-BDP-CAT** (6.3 μM) and **Bf-BDP-CAT** (1.6 μM) with 4.32 and 3.85-log reduction, respectively, which is an excellent value compared to other BODIPY systems.^105,106^ For instance, the diiodinated BODIPY studied by Shi, Li, and co-workers achieved 0.82-log reduction at a much higher concentration of 40 μM of photosensitizer, but at lower light dose of 3.6 J·cm^–2^.^35^ The remaining studied complexes also exhibited photoactivity toward the biofilm in MFC concentrations determined against planktonic *C. albicans*—**Bf-BDP-ZWIT** (6.3 μM)—2.24-log reduction, **NMe-BDP-CAT** (1.6 μM)—3.20-log reduction, and **CH2-BDP-CAT** (1.6 μM)—3.02-log reduction. On the contrary, the referential **BF2-BDP-CAT** (12 μM) exhibited almost no photoactivity toward the mature biofilm at the studied concentration (only a 0.32-log reduction), despite its high photoactivity toward planktonic cells (a 7-log reduction).

Morphological analyses of the 48 h *C. albicans* biofilm were performed for the most active compounds (**Bf-BDP-CAT** at concentration 1.6 μM; **O-BDP-CAT** at concentration 6.3 μM) using scanning electron microscopy (SEM, Figure 12). After irradiation, the cells showed a loss of firmness—the surface irregularities and signs of deformations are clearly visible (Figure 12a,c), unlike untreated controls or dark-incubated samples. Some cells appear collapsed, indicating significant destruction of their structure. Notably, on some surfaces of cells treated with **O-BDP-CAT** and light, the vesicle-like structures were visible (Figure 12c). These structures were not observed in the samples treated with **Bf-BDP-CAT**, nor in the control samples. This might indicate that **O-BDP-CAT** induces additional leakage of cellular material due to the damage of the cell wall and/or cell membrane. Moreover, photosensitizers and light treatment markedly inhibited *C. albicans* budding, which reduces cellular proliferation.

**Figure 12:**
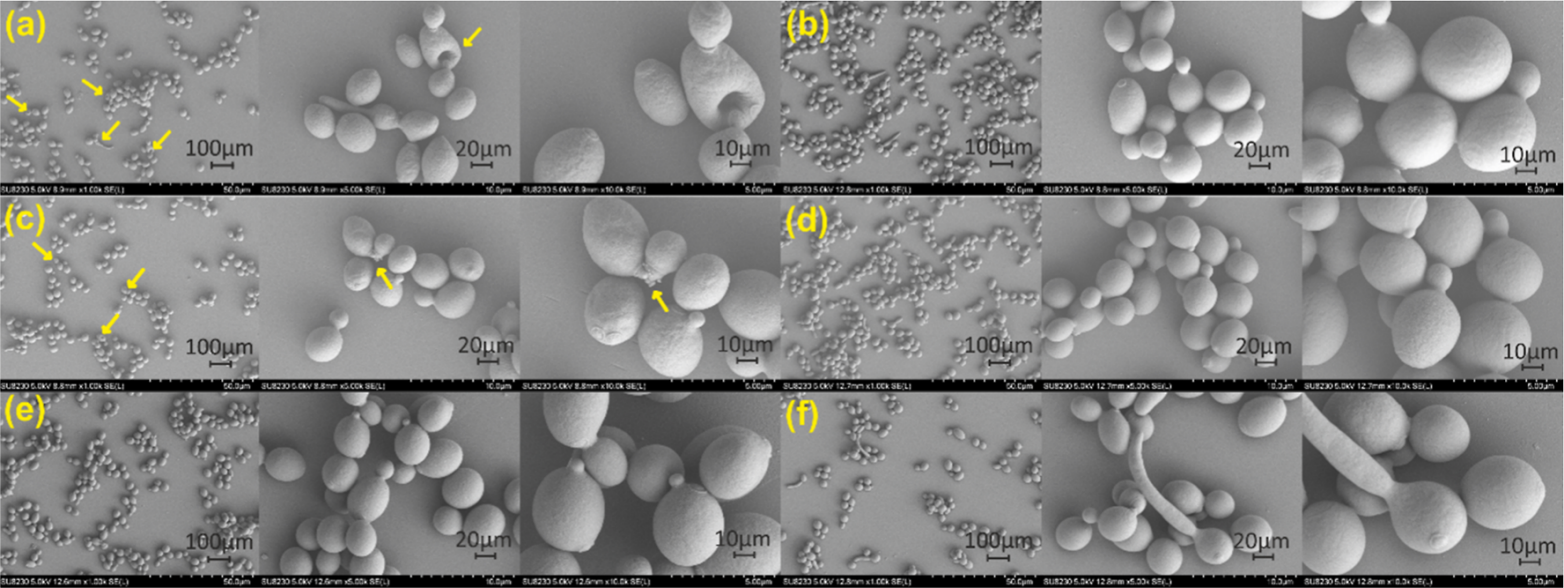
Morphological analyses of 48-h biofilms produced by *C. albicans* SC5314: (a) biofilm treated with **Bf-BDP-CAT** (1.6 μM) and irradiated with white light, (b) biofilm treated with **Bf-BDP-CAT** (1.6 μM) in the absence of light, (c) biofilm treated with **O-BDP-CAT** (6.3 μM) and irradiated with white light, (d) biofilm treated with **O-BDP-CAT** (6.3 μM) in the absence of light, (e) untreated biofilm irradiated with white light, (f) untreated biofilm in the absence of light. The most visible irregularities were marked with yellow arrows.

Furthermore, the adhesion of pretreated cells of *C. albicans* on the monolayer of the L929 cell line was examined. The attachment of the cells to the surface is the first step in biofilm formation. Thus, the inhibition of cell adherence might hinder the development of potential infection. The concentrations of the studied complexes were two times lower than the determined MFC values. Interestingly, a significant reduction in the adhesion of *C. albicans* cells to the monolayer of the L929 cell line was observed, even without irradiation (Figure 13). The reduction of yeast cells’ adherence ranged from 52% for **BF2-BDP-CAT** (6.3 μM) to 87% for **Bf-BDP-ZWIT** (3.1 μM). Moreover, upon irradiation, the cells have almost completely lost the ability to adhere. Therefore, it is also highly likely that, following the application of the therapy, the surviving fungal cells will not attach to the surfaces and form a biofilm, thereby limiting the progression of infection.

**Figure 13:**
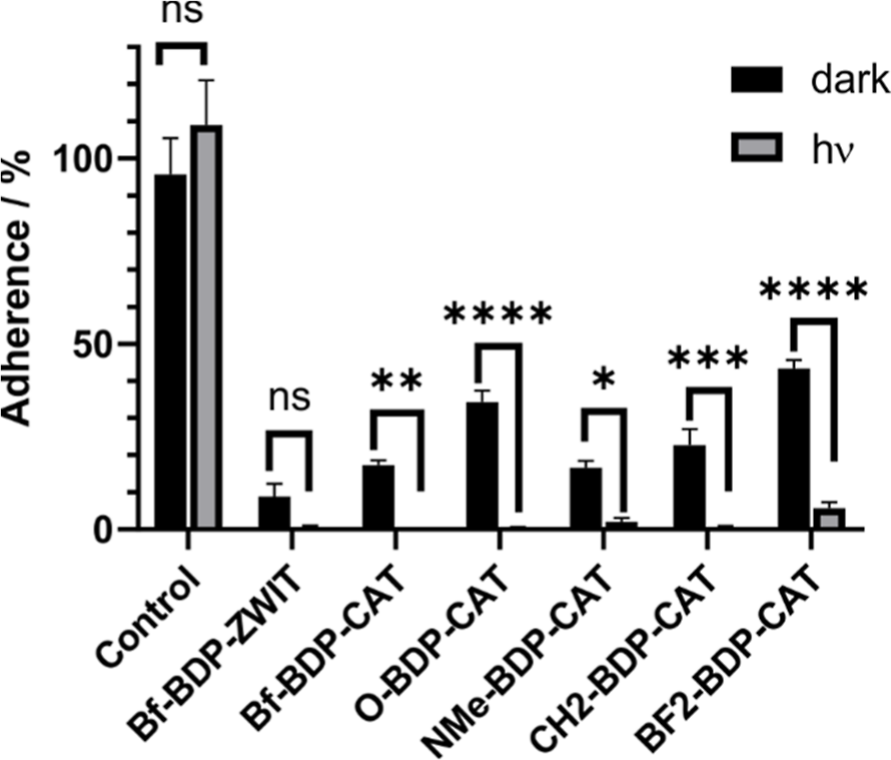
Effect of the studied BODIPY complexes on the adherence of *C. albicans* cells to the L929 cell line monolayer, expressed as the mean percentage of adherent cells (3 independent repetitions), relative to the total number of added cells. The compounds were tested at concentrations twice lower than MFC—**Bf-BDP-ZWIT** (3.1 μM), **Bf-BDP-CAT** (0.8 μM), **O-BDP-CAT** (3.1 μM), **NMe-BDP-CAT** (0.8 μM), **CH2-BDP-CAT** (0.8 μM), **BF2-BDP-CAT** (6.3 μM). *p < 0.05, **p < 0.01, ***p < 0.001, ****p < 0.0001, ^ns^ *p* > 0.05 compared with the dark control at the same concentration.

### 2.10 Cytotoxicity against Mammalian Cells

The in vitro cytotoxicity of the studied photosensitizers against the L929 cell line *via* MTS assay was determined. For the most photoactive compounds on *C. albicans* cells, concentrations corresponding to 4-fold the MFC, 2-fold the MFC, exact MFC, half the MFC, and one-quarter of the MFC were evaluated (Figure 14). Overall, the studied BODIPY dyes did not demonstrate a significant cytotoxic effect on the L929 cell line. A minor reduction in cell viability was observed only at the highest tested concentrations (4-fold the MFC). Namely, for **Bf-BDP-ZWIT** (25 μM), **O-BDP-CAT** (25 μM), **NMe-BDP-CAT** (6.3 μM), and **BF2-BDP-CAT** (50 μM) the cell viability was 83.0%, 91.8%, 84.3%, and 90.1%, respectively. Meanwhile, the **O-BDP-CAT** exhibits significant dark cytotoxicity against planktonic *C. albicans* at a concentration of 12.5 μM. These findings indicate that the studied C-BODIPY dyes can be considered harmless for mammalian cells.

**Figure 14:**
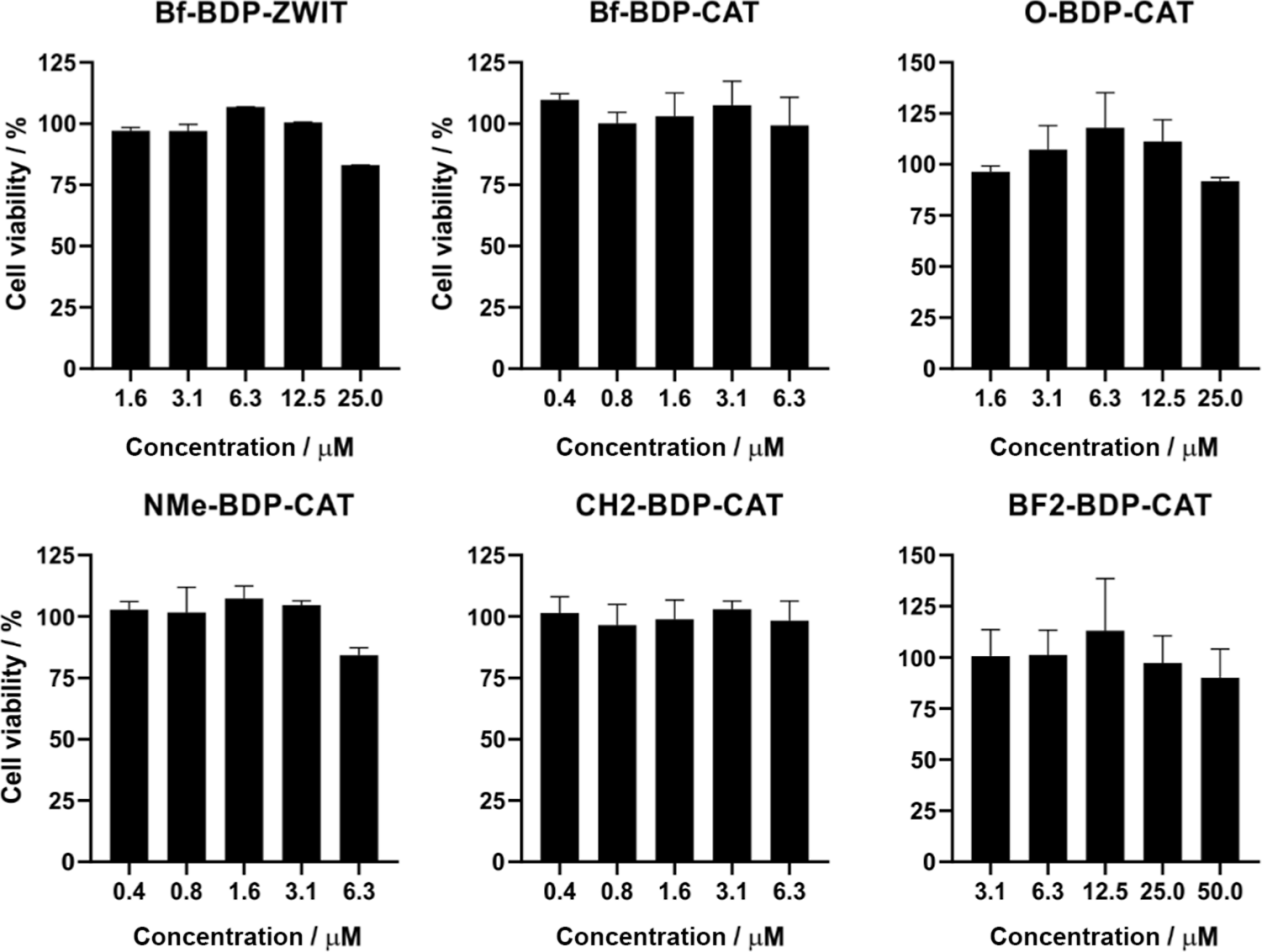
Cytotoxicity assays of the BODIPY dyes against the L929 cell line at different concentrations (4-fold the determined MFC, 2-fold the MFC, MFC, half of the MFC and one-quarter of the MFC).

### 2.11 In Vivo Studies

The therapeutic efficacy of **NMe-BDP-CAT** (selected as one of the most active photosensitizer) was investigated in a simple *in vivo* model—*Galleria mellonella* larvae (moth from the *Pyralidae* family). The larvae were infected with the *C. albicans* SC5314 strain and the injected concentration of the dye was 1.6 μM (MFC) corresponding to 0.06 mg·kg^–1^ per larvae. The survival curves (Figure 15) from the photoirradiation experiments indicate that the studied photosensitizer efficiently hampers the development of infection, even in relatively low doses. In the experiments performed without photosensitizers, the population of *G. mellonella* decreased much faster. These experiments also indicate that the BODIPY complexes are not toxic to larvae.

**Figure 15:**
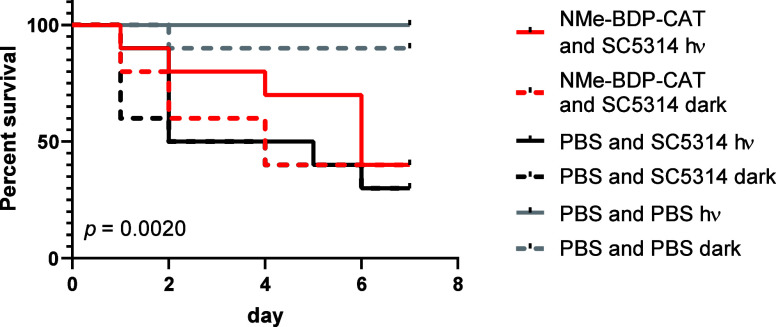
Photoinactivation studies on *C. albicans*-infected *G. mellonella* larvae. To determine the *p*-value, log-rank (Mantel–Cox) test was performed.

## 3 Conclusions

We have developed a new approach to heavy-atom-free BODIPY photosensitizers for antimicrobial photodynamic therapy. The proposed compounds are relatively simple, easily synthesizable, and structurally tunable, allowing their physicochemical properties to be adjusted without compromising their photocatalytic performance. They show strong absorption in the visible region and are characterized by enhanced stability in comparison to BF_2_-BODIPY counterparts. The orthogonal arrangement of organoboron and dipyrromethene moieties facilitates SOCT-ISC to the triplet state, though DFT calculations indicate variations in photophysical mechanism arising from the different electronic properties of organoboron cores. The triplet state lifetimes vary in range 66–179 μs, which is sufficient for the activation of ROS. Concordantly, photocatalytic experiments with ROS probes confirm strong photoactivity for the **NMe-BDP-R**, **O-BDP-R**, **Bf-BDP-R**, and **CH2-BDP-R** systems, which correlates with high biological activity of the corresponding cationic species. Importantly, these compounds produce both types of ROS, with the total yield and type I/type II ratio strongly depending on the structure of the organoboron unit.

Antifungal testing demonstrated remarkable photocytotoxicity against the opportunistic yeast *C. albicans* at low concentrations (1.1 μg/mL), achieving a reduction of up to 7-log in the planktonic cells. Additionally, the C-BODIPY exhibited significant photoactivity toward the fungal biofilm, achieving a 4.3-log reduction in MFC values. In particular, C-BODIPYs modified with ternary ammonium groups were the most active among the studied systems, placing them among the most active photosensitizers reported to date. The photosensitizers functionalized with the zwitterionic group displayed lower activity due to their generally lower solubility in an aqueous environment. Exceptionally, **Bf-BDP-ZWIT** showed high photoactivity combined with low dark anti-*Candida* activity. The cell death occurred primarily through the apoptosis mechanism. All compounds showed no dark cytotoxic effects on mammalian cells at concentrations 4-fold higher than the MFC. Photodynamic treatment proved highly effective against the first step of biofilm formation, specifically reducing cell adhesion to biotic surfaces, thereby preventing the development of fungal infections. The therapeutic efficacy of selected cationic C-BODIPY (**NMe-BDP-CAT**) was also confirmed in photoinactivation studies on *C. albicans*-infected *G. mellonella* larvae. Furthermore, the performed studies suggest that C-BODIPY derivatives exhibit rather similar intracellular localization patterns, presumably localizing close to vacuoles. It seems that the observed differences in their photodynamic activity result mostly from differences in inherent electronic structures of boracyclic cores, affecting PET and ISC processes, ultimately leading to variations in the amount and type of ROS produced.

Finally, this work, together with our previous research in this area, proved that the proposed molecular design of heavy-atom-free photosensitizers has an universal character and can be easily adopted for various targets. The photoactivity of the spirocyclic C-BODIPY mostly results from the structure of the organoboron unit, which governs the efficiency of SOCT-ISC and ROS production. Simultaneously, various modifications can be independently introduced to improve cell selectivity and biocompatibility. Subsequent work will demonstrate the application of this group of compounds to cancer treatment.

## 4 Experimental Section

### 4.1 General

Synthetic procedures for all studied compounds and intermediates, along with the NMR (Figures S81–S173) and HR-MS (Figures S174–S210) analytical data, are placed in the Supporting Information. Compounds are >95% pure by elemental analysis (all compounds) and HPLC (**X-BDP-CAT** series and **Bf-BDP-ZWIT**). HPLC chromatograms are placed in the Supporting Information (Figures S202–S207, Supporting Information).

All the used reagents were purchased from Merc, Acros, Apollo Scientific, and Alfa Aesar. Starting materials including BCl_3_ (1 M in hexane), benzaldehyde, 4-iodobenzaldehyde, 4-diethylaminobenzaldehyde, DDQ, *p*TsOH, N­(*i*Pr)_2_Et, BF_3_·OEt_2_, CF_3_SO_2_OCH_3_, 1-dimethylamino-2-propyne, CuI, Pd­(PPh_3_)_2_Cl_2_, and 1,3-propanesultone were used as received without additional purification. 2,4-Dimethylpyrrole was purified by vacuum distillation directly before the reaction. It is stored in Schlenk with a Rotaflo valve under an argon atmosphere. DCM, THF, benzene, and toluene were purified using MBraun SPS and stored over 4 Å molecular sieves under argon. Reactions and manipulations involving air and moisture-sensitive reagents were carried out under an argon atmosphere.

### 4.2 NMR and HR-MS

^1^H, ^19^F, and ^13^C­{^1^H} NMR spectra were recorded on Agilent NMR 400 MHz DDR2, JEOL JNM-ECZL 600 MHz, and Bruker Advance III 300 MHz spectrometers. ^1^H and ^13^C­{^1^H} chemical shifts were referenced to TMS by using known chemical shifts of solvent residual peaks. In the ^13^C­{^1^H} NMR spectra, the resonances of boron-bound carbon atoms were not observed in most cases as a result of their broadening by the quadrupolar boron nuclei. HR-MS analyses were performed on a GCT Premier mass spectrometer equipped with an EI ion source and a Maldi SYNAPT G2-S HDMS spectrometer equipped with an ESI ion source.

Elemental analysis was performed using Elementar Vario EL III apparatus. Before measurement, each sample was kept under vacuum (3·10^–3^ mbar) at 50 °C for 3 h. Each elemental composition was reported as an average of two analyses.

### 4.3 HPLC Analysis

The UV–vis chromatograms were obtained using an Agilent 1260 Infinity III Prime Liquid Chromatography system equipped with an Agilent 1260 Infinity III Diode Array Detector WR. Separation was carried out on an Agilent ZORBAX RRHD Eclipse Plus C18 column (2.1 × 50 mm, 1.8 μm, 1200 bar) maintained at 30 °C. Dissolved samples were injected at a volume of 5 μL. The mobile phase, consisting of an aqueous solution of 0.1% formic acid (A) and methanol (B), was delivered at a flow rate of 0.25 mL/min in gradient mode: 0 min, 5% B; 12 min, 95% B; 20 min, 95% B; 20.10 min, 5% B; 27 min; 5% B. Detection was performed at 500 nm with a reference wavelength of 700 nm to eliminate baseline drift. The chromatograms shown were corrected by subtracting the corresponding blank.

### 4.4 EPR Spectroscopy

EPR spectroscopic experiments were conducted using a JEOL JES-FA 200, X-band CW-EPR spectrometer operating at 100 kHz field modulation. Measurements were carried out using the microwave power equal to 0.995 mW and modulation width equal to 0.1 and 0.01 mT. The *g*-factor value was determined using the JEOL internal manganese (Mn) standard. The freshly prepared solutions containing an equimolar amount of BODIPY and TEMP (*c* = 5 × 10^–5^ M) were initially measured with EPR, showing no signals associated with paramagnetic species. After 5 min of irradiation with neutral-white light (40 mW·cm^–2^), the formation of TEMPO (*g*-factor of 2.006) was clearly observed.

### 4.5 Crystallography

X-ray diffraction data were collected on a SuperNova diffractometer equipped with an Atlas detector using Cu–Kα radiation at 100 K. The crystal structures were obtained *via* X-ray data refinement employing the Independent Atom Model (IAM). Data reduction and analysis were carried out with the CrysAlisPro program.^107^ All structures were solved by intrinsic phasing using SHELXT^108^ and refined using SHELXL-2014 ^109^ with the Olex2 suite.^110^ Crystal structures were deposited in Cambridge Crystallographic Data Centre, no. 2525436 (**BF2-BDP-CAT**) and 2525435 (**NMe-BDP-CAT**).

### 4.6 Theoretical Calculations

Single-molecule calculations were performed using the Gaussian16 ^111^ program at the DFT B3LYP/6-311++G­(d,p)^112–115^ level of theory. Excited state geometries were obtained with TD-DFT methods at the CAM-B3LYP/6-311++G­(d,p)^116^ level of theory. Natural transition orbitals (NTO) were calculated for each excited state. The calculations show that molecular geometries of singlet and triplet excited states are generally preserved from the corresponding ground states. The molecular orbitals and natural transition orbitals were visualized with the Avogadro software.^117^

### 4.7 Electrochemistry

Cyclic voltammetry was conducted in a three-electrode, one-compartment cell using a Autolab potentiostat PGSTAT 20 (EcoChemie, The Netherlands). Measurements were performed in 0.1 M Bu_4_NBF_4_ (DCM). All solutions were bubbled with argon prior to measurement and the measurement was conducted in an inert atmosphere. Concentration of the studied compounds: 1 × 10^–3^ M. Electrodes: working (glassy carbon disk, diameter 2 mm), counter (Pt wire), reference (Ag/0.1 M AgNO_3_/CH_3_CN, calibrated against ferrocene). The potential of the reference electrode versus the ferrocene redox couple was checked before and after experiments. All cyclic voltammetry measurements were performed at room temperature with a scan rate of 50 mV·s^–1^. Differential pulse voltammetry experiments were performed with a modulation time of 50 ms, a modulation amplitude of 10 mV, and a step potential of 5 mV.

### 4.8 UV–Vis Spectroscopy

The UV–vis absorption spectra were recorded using a Hitachi UV-2300II spectrometer. The emission spectra of solutions were recorded using Edinburgh FS5 equipped with an enhanced range photomultiplier detector (PMT-EXT). The measurements were performed at room temperature, according to published procedures.^118^ Suprasil quartz cuvettes (10.00 mm) were used. Quantum yields of emission were determined in diluted solutions (*A* ≈ 0.03–0.05 for the longest wavelength band) by comparison with known standards—fluorescein (0.1 M NaOH_(aq)_, QY^F^_r_ = 0.92).^119^ All measurements were carried out at room temperature. The concentration of BODIPY complexes was adjusted to reach similar absorbance to absorbance of the reference solution at the excitation wavelength. Fluorescence quantum yields were calculated according to the equation

$${QY}^{F}={QY}_{r}^{F}\cdot \frac{F_{x}}{F_{r}}\cdot \frac{1-10^{-A_{r}}}{1-10^{-A_{x}}}\cdot \frac{n_{x}^{2}}{n_{r}^{2}}$$

where *F* is the relative integrated photon flux of sample (*x*) and reference (*r*), *A* is the absorbance at the excitation wavelength, and *n* is the refractive index of used solvents.

Fluorescence lifetime measurements were acquired using a time-correlated single photon counting (TCSPC) system equipped with a picosecond pulsed 340 nm EPLED source. Phosphorescence was not observed at room temperature (experiments were performed for degassed solutions using the freeze–pump–thaw method).

The Gibbs free energy changes for photoinduced electron transfer (Δ*G*_PET_) were calculated based on the Rehm–Weller equation

$${\Delta G}_{PET}={eE}_{1/2}^{ox}-{eE}_{1/2}^{red}-E_{0,0}+E_{col}$$

where *E*_1/2_^ox^ and *E*_1/2_^red^ are oxidative and reduction potentials, respectively; *E*_0,0_ is the energy of the S_0_(*v* = 0)→_S_1­(*v* = 0) transition obtained from the intersection of normalized absorption and emission spectra; *E*_col_ is the Coulombic interaction between positive and negative charges located on the boracycle and BDP, respectively.

### 4.9 Photostability

In 4 mL vials, 1.5 mL of BODIPY complex solutions in CHCl_3_ or in 20% (v/v) serum (FBS, Thermo Fisher Scientific) in water (*c* = 5 × 10^–5^ M) was irradiated using 26 W neutral-white LED strips (40 mW·cm^–2^) for 10 h (CHCl_3_)/60 min (serum). At hourly (CHCl_3_) or 5–10 min intervals (serum), the absorption spectra were measured to monitor the decomposition processes. Simultaneously, the experiments were conducted in the absence of light to assess the hydrolytic stability. For all experiments, the half-lives of photodegradation were calculated based on the drop in the absorbance values at the maximum absorption wavelength of the specific BODIPY complex assuming pseudo-first-order kinetics.

### 4.10 Nanosecond Transient Absorption

Laser flash photolysis (LFP) experiments were performed using a tunable primoScan optical parametric oscillator (GWU-Lasertechnik), pumped by the third harmonic (355 nm) of a Q-smart 450 Nd/YAG laser (Quantel), which has been described in detail elsewhere.^120^ Samples were excited at 480 nm with pulse energies adjusted to approximately 1 mJ to avoid multiphoton excitation artifacts. The absorbance of the sample at the excitation wavelength was adjusted to 0.5. Transient absorption kinetic traces were recorded at 10 nm intervals across the 350–700 nm spectral region. Prior to measurements, solutions were deoxygenated by purging with high-purity argon for at least 20 min. All experiments were conducted in 1 × 1 cm quartz cuvettes.

### 4.11 Photocatalytic Properties

Catalytic oxidations of 2-furoic acid were conducted using a photoreactor equipped with a neutral-white LED strip with 56 diodes (40 mW·cm^–2^; CIE 1931 coordinates: 0.38, 0.38; 4000 K) at r.t. (Figure S49, Supporting Information). The reaction vials contained 1.5 mL of chloroform solutions of 2-furoic acid (12 mg·mL^–1^, 0.107 M) and BODIPY complex (0.05 mol % relative to the substrate −5 × 10^–5^ M) and a cross-shaped stir bar. The distance of the reaction vial from the light source was equal for each sample (25 mm). The conversion of 2-furoic acid was monitored by ^1^H NMR spectroscopy.

Quantum yield of photogeneration of singlet oxygen was investigated indirectly with UV–vis spectroscopy and diphenylisobenzofuran (DPBF) used as the ROS trap. The DCM solution containing a singlet oxygen trap and an investigated compound (at concentration of 0.02 mM) was constantly illuminated with an Oxxius diode laser: 505 nm model LBX-505-70-CSB-PPA operating at 10 mW, respectively. UV–vis spectra of the solution were collected in even time periods with an AvaSpec-ULS2048XL-EVO-RS-UA spectrometer. A drop in the absorbance of DPBF at 414 nm in time was observed, indicating photogeneration of the singlet oxygen. The quantum yield of singlet oxygen photogeneration was determined by the relative method with respect to **Bf-BDP** (QY^O^ = 78%), which was measured in our previous work.^81^ The following equation was used

$${QY}_{x}^{O}={QY}_{r}^{O}\frac{k_{x}}{k_{r}}\frac{1-10^{-A_{r}}}{1-10^{-A_{x}}}$$

where *x* and *r* stand for the substance under study and reference (**Bf-BDP**), respectively; *k* denotes DPBF consumption rates, and *A* represents the absorbance of the investigated compound or reference at 505 nm.

The generation of singlet oxygen in an aqueous environment (PBS, Thermo Fisher Scientific) was assessed by employing 9,10-anthracenediylbis­(methylene)­dimalonic acid (ABDA) as a UV–vis indicator. The solutions of ABDA (*c* = 10^–4^ M) and BODIPY complex (*c* = 10^–5^ M) were irradiated with white light (40 mW·cm^–2^) in given time intervals. A drop in absorbance of the ABDA scavenger at 379 nm was observed with the progress of reaction. An analogous experiment was carried out without addition of a photosensitizer, showing a negligible drop in absorbance upon irradiation.

For total ROS detection in aqueous solutions we used 2′,7′-dichlorodihydrofluorescein (DCFH). The DMSO/water (2% v/v) solutions (3 mL) containing DCFH (*c* = 5 × 10^–5^ M) and BODIPY complex (*c* = 10^–5^ M) were irradiated with white light (40 mW·cm^–2^) in given time intervals. After irradiation, the emission spectra of DCFH were monitored in a range of 480–600 nm and the increase in emission intensity of fluorescent product at 525 nm was recorded.

The generation of the hydroxyl radical (OH^•^) was monitored by employing disodium terephthalate (NaTA). NaTA (*c* = 5 × 10^–4^ M) and BODIPY complex (*c* = 2 × 10^–5^ M) were solubilized in PBS and irradiated with white light (40 mW·cm^–2^) followed by the detection of an emission band at λ_em_ = 392 nm, resulting from formed disodium 2-hydroxyterephtalate. In both borafluorene systems (**Bf-BDP-CAT**, **Bf-BDP-ZWIT**) and **O-BDP-CAT** an unexpected band at 334 nm emerged upon irradiation (Figure S53, Supporting Information). It might suggest a side reaction between PS and the scavenger.

For superoxide anion radical (O_2_^•–^) detection, nitro blue tetrazolium (NBT, *c* = 5 × 10^–5^ M), NaOH (*c* = 0.1 M), and BODIPY complex (*c* = 2 · 10^–5^ M) were solubilized in a water/DMSO mixture (2:1) and placed in a quartz cuvette, which was irradiated in given time intervals and then absorption spectra were registered. The increase in the absorption band at 645 nm indicates formazan formation. A slow blue coloration was also observed in the irradiated NBT/NaOH mixture without the photosensitizer. Thus the absorbance at 645 nm was corrected by subtracting the blank sample value at corresponding time points. In the case of **BF2-BDP-CAT** and **SO2-BDP-CAT**, addition of the photosensitizer to the scavenger solution produced an intense blue color, even without irradiation. The subsequent increase in the formazan band was insignificant. This suggests a side reaction between BODIPY and the NBT scavenger.

### 4.12 The Fluorescence Microscopy Measurements

The images were taken with a fluorescence microscope (Eclipse Ni, Nikon, Japan) using a 100× objective lens with immersion oil. White, blue (filter block FF01-392/23 nm excitation, FF02-447/60 emission), and green (filter block FF01-474/27 excitation, FF02-525/45 emission) light was applied. The fungal suspension of *C. albicans* ATCC 10231 (ATCC, Washington, USA) was incubated at 37 °C without the presence of light with the solution of the studied BODIPY dye in MFC concentration for 30 min. Subsequently, the suspension was centrifuged and the pellet was resuspended in the 0.9% m/v NaCl in H_2_O solution. Afterward, the standard staining with DAPI to localize the cell nucleus was applied.

### 4.13 Antimicrobial Photoinactivation Assays

Approximately 10 mL of liquid Sabouraud broth (Biomaxima, Lublin, Poland) was inoculated with a single colony of *C. albicans* ATCC 10231. Cells were grown overnight at 37 °C in aerobic conditions with shaking at 220 rpm (Benchtop shaker SI-600R LabCompanion, Jeio Tech, Daejeon, South Korea). The photosensitizer stock solutions of 5 mM were prepared in DMSO and subsequently 100-fold diluted in a proper liquid. Antimicrobial photoinactivation assays were performed in 96-well plates with microbial suspension in concentrations of 2·10^3^ CFU mL^–1^. A 100 μL of each photosensitizer solution was added to a well in eight repetitions. Thereafter, 100 μL of the microbial suspension was added to four wells, while 100 μL of the liquid medium was introduced to the remaining four wells to give a final volume of 200 μL in each well. The final concentration of photosensitizer was 25 μM. The 96-well plate was incubated at 37 °C and exposed to neutral-white light (CIE 1931 coordinates: 0.38, 0.38; 4000 K) for 1 h using an irradiation device equipped with LED (14 strips × 48 diodes = 672 diodes in total) attached to an aluminum plate (20 × 23 cm) with a radiator, mounted on threaded legs (Figure S50, Supporting Information). This design enables straightforward adjustment of the irradiation distance. In all experiments, the distance between the light source and the plate was fixed at 10 cm, ensuring homogeneous light distribution (40 mW·cm^–2^) without a measurable rise in temperature within the incubator. In a parallel experiment, the 96-well plate was incubated in the dark under identical temperature conditions. Subsequently, both plates were incubated overnight at 37 °C in the absence of light. Cell growth was analyzed with UV–Vis spectroscopy (optical density at 600 nm, OD_600_) with Synergy H4, BioTek. Measured OD_600_ for wells without microbial suspension were used as references. The survival fraction was estimated from the untreated control upon irradiation or in the absence of light. Subsequently, concentration experiments were conducted for photosensitizers that were deemed the most efficacious. On a 96-well plate, 100 μL of photosensitizers solutions (50 μM) was added in four repetitions each. Subsequently, a series of 2-fold serial dilutions in the vertical wells was prepared. Then, 100 μL of microbial suspension was added, resulting in a final volume of 200 μL and the desired concentrations of photosensitizers. Each 96-well plate was duplicated, with one being incubated and irradiated for 1 h at 37 °C in an aerobic environment, while the remaining plates were incubated in the dark under the same conditions. Subsequently, the plates were incubated overnight in the absence of light. Therefore, on 96-well plates, a series of 10-fold serial dilutions of the incubated microbial suspensions in sterile saline solution (10^–1^ to 10^–8^) were prepared. Then, 10 μL of each dilution was applied as droplets on a single Sabouraud agar plate (Biomaxima, Lublin, Poland). After the droplets were allowed to dry, the Petri-dishes were incubated overnight at 37 °C. The colonies were counted and a survival fractions were determined using an untreated control upon irradiation or in the absence of light as a reference.

### 4.14 Oxidative Stress Assay

10 mL of YEPD liquid medium (20 g/L peptone, 10 g/L yeast extract, 20 g/L glucose, A&A Biotechnology, Gdańsk, Poland) was inoculated with a single colony of *C. albicans* SC5314 strain and incubated for 18 h at 37 °C. Then, the yeast suspension was diluted to a density of 1 × 10^6^ cells/mL. The yeast suspension was added to a 24-well plate (1980 μL), followed by the addition of the solution of the studied BODIPY photosensitizers in DMSO (20 μL) to obtain the determined MFC concentrations. Simultaneously, the negative control was prepared by the addition of 20 μL of 96% DMSO instead of the photosensitizer solution, while the positive control consisted of *C. albicans* cells treated with 3% H_2_O_2_ solution. The plate was duplicated: one was incubated for 1 h at 37 °C in the absence of light, while the second was irradiated for 1 h at 37 °C. Subsequently, 495 μL of each suspension was transferred into CELLview cell culture dishes with four compartments, followed by the addition of 5 μL of CellRox Deep Red Reagent (final concentration 2.5 μM) and 5 μL of Calcofluor white (final concentration 2.5 μg/mL, Sigma-Aldrich). The culture dishes were incubated at 37 °C for 0.5 h, and the microscope observations were carried out using confocal laser scanning microscopy (CLMS) with Zeiss Axio Observer 7 (Zeiss, Germany).

### 4.15 Confocal Laser Scanning Microscopy Measurements

10 mL of YEPD liquid medium was inoculated with a single colony of *C. albicans* SC5314 strain (ATCC, Washington, USA) and incubated for 18 h at 37 °C. Then, the yeast suspension was diluted to a density of 1 × 10^2^ cells/mL. The yeast suspension was added to a 24-well plate (1980 μL), followed by the addition of the solution of the studied BODIPY photosensitizers in DMSO (20 μL) to obtain the 0.5 x MFC concentrations. As a control, 20 μL of 96% DMSO instead of the photosensitizer solution was used. The plate was duplicated. Subsequently, the photoinactivation assay on planktonic cells was conducted as described above, while the duplicated plate was incubated without light under the same conditions. Subsequently, 495 μL of each suspension was transferred into CELLview cell culture dishes with four compartments, followed by the addition of the dyes: Calcofluor White (2.5 μg/mL, Sigma-Aldrich), Hoechst 33342 (1 μg/mL, Thermo Fisher Scientific), and propidium iodine (1 μg/mL, Thermo Fisher Scientific). The dishes were incubated for 0.5 h at 37 °C in the dark, and the microscope observations were carried out using confocal laser scanning microscopy (CLMS) with a Zeiss Axio Observer 7 (Zeiss, Germany). Due to excessive growth inhibition and the resulting low cell density that prevented accurate measurements, the experiments were repeated using the 1 × 10^5^ cells/mL density for compounds **Bf-BDP-ZWIT**, **Bf-BDP-CAT**, **O-BDP-CAT**. Additionally, the experiments for **BF2-BDP-CAT** were repeated at a lower concentration (0.13 x MFC) due to its high fluorescence signal, which caused detector saturation at a 0.5 x MFC concentration. For the CLSM measurements, the same procedure as for the lower cell density was used.

### 4.16 Flow Cytometry

10 mL of YEPD liquid medium was inoculated with a single colony of *C. albicans* SC5314 and incubated for 18 h at 37 °C in a shaker incubator (250 rpm, Innova 40, New Brunswick, Edison, USA). Subsequently, 1980 μL of cell suspension (10^3^ CFU·mL^–1^) was placed on 24-well plates, followed by the addition of 20 μL of 100-fold concentrated photosensitizer solutions in DMSO to obtain determined MFC concentrations: **Bf-BDP-ZWIT** (6.3 μM), **Bf-BDP-CAT** (1.6 μM), **O-BDP-CAT** (6.3 μM), **NMe-BDP-CAT** (1.6 μM), **CH2-BDP-CAT** (1.6 μM), **BF2-BDP-CAT** (12.5 μM). The experiment was performed in four technical repetitions. After exposure, the cell suspensions (1 mL) were transferred into Eppendorf tubes and centrifuged for 5 min at 250 rcf (Centrifuge 5910 Ri, rotor FA-20 × 5, Eppendorf, Hamburg, Germany). The cells were washed twice with 1 mL of PBS (phosphate-buffered saline), and suspended in Annexin V binding buffer (100 μL). The cells were stained with Annexin-V (Sigma-Aldrich) and propidium iodide (PI) (Sigma-Aldrich) using the Annexin-V FLOUS Staining Kit (Roche Diagnostics GmbH, Mannheim, Germany). The samples were measured using a FACSLyric Flow Cytometer (BD Biosciences, San Jose, USA).

### 4.17 Biofilm Photoinactivation Assays

The *C. albicans* SC5314 strain was employed in studies due to its high capability of biofilm formation.^104^ As a biotic surface, the monolayer of L929 cell line (mouse fibroblasts) was used (Merck, Darmstadt, Germany). The biofilm photoinactivation assays were conducted on 24-well plates. 10 mL of YEPD liquid medium was inoculated with a single colony and incubated for 18 h at 37 °C in a shaker incubator (250 rpm, Innova 40, New Brunswick, Edison, USA). Simultaneously, L929 cells suspended in MEM (10% Fetal Bovine Serum, 1% Penicillin/Streptomycin, Gibco, Thermo Fisher Scientific, USA) were seeded into 24-well plates (1 × 10^5^ cells/mL) and incubated at 37 °C and 5% CO_2_ for 18 h to obtain a monolayer. Subsequently, the liquid media was removed with a Pasteur pipet and replaced with a fresh 900 μL portion of supplemented MEM. The overnight culture of *C. albicans* was diluted in sterile PBS to obtain a density of 1 × 10^6^ cells·mL^–1^. Afterward, the 10-fold dilutions in PBS (10^–1^ to 10^–5^) were prepared. 100 μL of 10^–5^ dilution was cultivated on Petri dishes containing YEPD agar in three repetitions as a control of the yeast suspension. The plates were incubated at 37 °C for 18 h. 100 μL of 10^–4^ dilution was added on 24-well plates with a fibroblast monolayer. The plates were incubated for 24 h at 37 °C to allow for the adherence of fungi to the mammalian cells and biofilm formation. Afterward, the medium was disposed from the 24-well plates to remove nonadherent fungi and the remaining cells were washed twice with sterile PBS. Subsequently, 990 μL of fresh MEM was added to each well. The 100-times concentrated solutions of the studied BODIPY complexes were prepared in DMSO. 10 μL of photosensitizer solutions were added to the wells containing *C. albicans* and L929 cells in three repetitions. Each 24-well plate was duplicated to determine the photoinactivation effect as well as the potential dark anti-*Candida* activity. The plates were incubated for 1 h at 37 °C in the absence of light to allow for the photosensitizer uptake. Subsequently, one of the plates was irradiated with a homemade device for 1 h at 37 °C and the second plate was still incubated in the same conditions in the dark. After the incubation, the medium was removed and the wells were washed twice with sterile PBS to reduce the photosensitizer concentration. Subsequently, 1000 μL of H_2_O was added to each well and the plates were placed on ice for 30 min to induce the lysis of mammalian cells and detachment of fungal cells. Afterward, the cell suspensions were transferred to centrifuge tubes and centrifuged for 10 min at 12 000 rpm (17700*g*; Centrifuge 5910 Ri, rotor FA-20 × 5, Eppendorf, Hamburg, Germany) at 19 °C. The water was removed and the pellet was resuspended in 1000 μL of PBS. 10-fold dilutions were prepared and inoculated on Petri dishes with YEPD agar. The plates were incubated for 18 h at 37 °C and subsequently the colonies were counted. The biofilm photoinactivation was established with respect to the untreated control.

### 4.18 Scanning Electron Microscope Measurements

10 mL of YEPD liquid medium was inoculated with a single colony of *C. albicans* SC5314 strain and incubated for 18 h at 37 °C. Then, the yeast suspension was diluted in YEPD liquid medium to a density of 1 × 10^3^ cells·mL^–1^. Subsequently, 1 mL of the yeast suspension was added to a 24-well plate containing glass slides. The plate was duplicated. After 48 h of incubation (37 °C), the liquid medium was discarded, and the samples were washed with PBS (3 × 1 mL) to remove nonadherent cells. Then, a fresh portion of the YEPD liquid medium (990 μL) was added, followed by 10 μL of **Bf-BDP-CAT** and **O-BDP-CAT** solutions in DMSO to reach the determined MFC values. For the control, 10 μL of 96% DMSO was added instead of the photosensitizer. The plates were incubated for 1 h at 37 °C in the absence of light to allow compound uptake. Then, one of the identical 24-well plates was incubated for 1 h at 37 °C in the absence of light, while the second was irradiated for 1 h at 37 °C (40 mW·cm^–2^). After the incubation, the following sequence of washes (1 mL per washing) and incubation was performed: 5% glutaraldehyde (Sigma-Aldrich) in PBS (48 h, 4 °C), PBS (3 × 10 min, rt), 1% osmium tetroxide in PBS (90 min, rt), distilled water (2 × 10 min, rt), 35% ethanol (15 min, rt), 50% ethanol (15 min, rt), 75% ethanol (15 min, rt), 95% ethanol (15 min, rt). Subsequently, the glass slides with fixed cells were obtained from the wells and dried. The microscopic glass slides have been coated with gold–palladium alloy (80:20) using a Quorum Q150TS plasma sputter coater (Quorum Technologies, UK). SEM images have been recorded using a Hitachi SU8230 high resolution field-emission scanning electron microscope (Hitachi High-Technologies, Japan). Samples have been attached to aluminum SEM stubs with the use of conductive adhesive tape and inserted into the microscope chamber. Images have been recorded using secondary electron detectors at the accelerating voltage of 5.0 kV, working distance of 8–13 mm, and magnification of 1000–10,000×.

### 4.19 The Adhesion Inhibition Assays

10 mL of YEPD liquid medium was inoculated with a single colony of *C. albicans* strain SC5314 and incubated for 18 h at 37 °C. Then, the yeast suspension was diluted to a density of 1 × 10^6^ cells/mL and 10-fold dilutions in PBS (10^–1^ to 10^–5^) were prepared. To a 24-well plate with 1780 μL of supplemented MEM the 200 μL of 10^4^ yeast suspension was added and 20 μL of 100-times concentrated photosensitizer solution prepared in DMSO in three repetitions. Each plate was duplicated to determine photoinactivation of cells as well as dark anti-*Candida* activity. The plates were incubated for 1 h at 37 °C in the absence of light and then one of the duplicated plates was irradiated using a homemade device for 1 h at 37 °C, while the second was incubated under the same conditions without light. Then, the yeast suspensions were transferred to Eppendorf tubes and centrifuged for 10 min at 12,000 rpm (17700*g*; Centrifuge 5910 Ri, rotor FA-20 × 5, Eppendorf, Hamburg, Germany) at 19 °C. The pellet was washed twice with sterile PBS and resuspended in a fresh portion of MEM. Afterward, the pretreated yeast suspensions were transferred to the L929 cell line monolayer. The monolayer was prepared as described above. The plates were incubated for 90 min at 37 °C. After washing twice with PBS to remove the nonadherent cells, 1000 μL of H_2_O was added to each well and the plates were placed on ice for 30 min. The adherent fungal cells were recovered as described above. After the centrifugation, the cells were resuspended in PBS and inoculated on YEPD plates. The Petri dishes were incubated for 18 h at 37 °C and colonies were counted. The adhesion of the untreated cells was assessed as described above. Adherence was estimated as a percentage of the total number of added cells.^121^

### 4.20 Cytotoxicity Assay

Cytotoxicity of **Bf-BDP-ZWIT**, **Bf-BDP-CAT**, **O-BDP-CAT**, **NMe-BDP-CAT**, **CH2-BDP-CAT**, and **BF2-BDP-CAT** was assessed against the L929 cell line (Merck, Darmstadt, Germany) using MTS test (3-(4.5-dimethylthiazol-2-yl)-5-(3-carboxymethoxyphenyl)-2-(4-sulfophenyl)-2H-tetrazolium, MTS, Promega, USA). The cells were seeded (10^5^ cells·mL^–1^) into 96-well plates and maintained in culture medium MEM (10% fetal bovine serum, 1% Penicillin/Streptomycin) for 24 h at 37 °C and 5% CO_2_. Subsequently, the obtained monolayer of L929 cells was exposed to different concentrations of the studied photosensitizers for 24 h at 37 °C and 5% of CO_2_. Simultaneously, the 96-well plates without cells (photosensitizers solutions in medium) were prepared and incubated. 10 μL of MTS solution was added to each well, and after 2 h of incubation without the presence of light, the optical measurements (490 nm) using Spark Control M10 (Tecan Group Ltd., Mannedorf, Switzerland) were performed. Cell viability was determined as the ratio of the difference between the absorbance of treated cells and the absorbance of the photosensitizer solution without cells to the absorbance of untreated cells.

### 4.21 *G. mellonella* Treatment

*G. mellonella* larvae in the last instar stage were used. The larvae weighing between 150 and 250 mg were selected. For the aPDT assay, the larvae were divided into 6 different groups (10 larvae per group) and placed into sterile Petri dishes. The larvae from 4 groups were injected with 10 μL of 5 × 10^7^ cells·mL^–1^ *C. albicans* SC5314 inoculum in PBS into the last left pro-leg. Instantly, 10 μL of the solution of the dye in PBS (or DMSO in PBS) was injected into the last right pro-leg. The remaining two groups were injected with 10 μL of PBS into both pro-legs. As a result, two identical sets of larvae (*C. albicans* + the dye) and two sets of control groups (*C. albicans* + PBS and PBS + PBS) were prepared. After the injection, the larvae were incubated at 35 °C in the absence of light for 30 min. Then, one set of the treated and control groups was irradiated for 1 h at 35 °C with the device used in antimicrobial photoinactivation experiments (neutral-white light, 40 mW·cm^–2^), and the other set was incubated at 35 °C in the dark. After treatment, the larvae were incubated at 22 °C in the absence of light for 7 days. They were examined daily (first day after 18 h, and for each subsequent day after 24 h). The larvae were considered dead if they showed no response to touch.

### 4.22 Statistical Analysis

The statistical analysis of the obtained results was performed using two-way analysis of variance (ANOVA) using GraphPad Prism 8.0.2. software.^122^ The photoinactivation of planktonic cells was performed in one biological replicate with four technical replicates, whereas the photoinactivation of the biofilm, as well as cell adhesion assays, was conducted in three biological replicates, each with three technical replicates. The cytotoxicity effect on the L929 cell line was performed in one biological repetition with three technical repetitions. Data are represented as the means of the obtained results. Error bars represent the standard deviation (SD).

## Supplementary Material









## Data Availability

The data supporting this article have been included as part of the Supporting Information (SI). Crystallographic data are provided free of charge by the Cambridge Crystallographic Data Centre (CCDC) service (CCDC code for **NMe-BDP-CAT**: 2525435; CCDC code for **BF2-BDP-CAT**: 2525436).

## Notes

Synthetic procedures, data collection, processing and refinement parameters for a single crystal structures, UV–vis absorption and emission spectra, fluorescence decays, results of transient absorption experiments, results of stability studies in CHCl_3_ and 20% (v/v) serum in water, singlet oxygen emission spectra, absorption and emission spectra from the experiments involving ROS scavengers, EPR spectra for **X-BDP-CAT**, CV, and DPV curves for **X-BDP**, MO, and NTO orbital pictures, energy diagrams and Hirshfeld atomic charges derived from DFT calculations, fluorescence microscopy images and CLSM measurements ^1^H, ^13^C, ^11^B NMR spectra and HR-MS data, and HPLC chromatograms (PDF)
The crystallographic information file for **NMe-BDP-CAT** and **BF2-BDP-CAT** (CIF)
The atom coordinates for DFT-optimized geometries (XYZ)
SMILES, MFC in dark, MFC under irradiation, log10 reduction of photoinactivated biofilm and IC_50_ for final compounds (CSV)

## Abbreviations

ABDA: 9,10-anthracenediylbis­(methylene)­dimalonic acid
ADPs: atomic displacement parameters
aPDT: antimicrobial photodynamic therapy
BF_2_-BODIPY: difluoroboron BODIPY
BODIPY: 4,4-difluoro-4-bora-3a,4a-diaza-s-indacene
C-BODIPY: diorganicboron functionalized BODIPY
CFU: colony-forming unit
CIE: Comission Internationale de l’Eclairage
CLSM: confocal laser scanning microscopy
CT: charge transfer excited state
DAPI: 4′,6-diamidino-2-phenylindole
DCFH: 2′,7′-dichlorodihydrofluorescein
DPBF: diphenylisobenzofuran
DPV: differential pulse voltammetry
2-FA2: furoic acid
FBS: fetal bovine serum
HONTO: highest occupied natural transition orbitals
IAM: independent atom model
ISC: intersystem crossing
LE: local excited state
LED: light-emitting diode
LFP: laser flash photolysis
LUNTO: lowest unoccupied natural transition orbitals
MFC: minimum fungicidal concentrations
MTS: (3-(4.5-dimethylthiazol-2-yl)-5-(3-carboxymethoxyphenyl)-2-(4-sulfophenyl)-2H-tetrazolium)
NBT: nitro blue tetrazolium
NIR: near-infrared
NTO: natural transition orbitals
PDT: photodynamic therapy
PET: photoinduced electron transfer
PI: propidium iodine
PMT-EXT: enhanced range photomultiplier detector
PS: photosensitizer
QY^F^: fluorescence quantum yield
QY^O^: singlet oxygen quantum yield
SOCT-ISC: spin–orbit charge-transfer intersystem crossing
SOCME: spin–orbit coupling matrix elements
NaTA: disodium terephthalate
TCSPC: time correlated single photon counting
TEMP: 2,2,6,6-tetramethylpiperidine
TEMPO: (2,2,6,6-tetramethylpiperidin-1-yl)­oxyl
THF: tetrahydrofuran
TOF: turnover frequencies
YEPD: yeast extract peptone dextrose
