# Supplementary material for: C‑BODIPY Dyads Based on Fused-Ring Organoboron Scaffolds for Efficient Photodynamic Therapy Targeting Candida albicans Yeast and Biofilms
Source: J Med Chem. 2026 Jun 1;69(11):13320–44. doi: 10.1021/acs.jmedchem.6c00280 (PMC13308890; doi:10.1021/acs.jmedchem.6c00280)
Supplement: Supplementary file 1 [file jm6c00280_si_001.pdf]

Supporting Information

for

C-BODIPY dyads based on fused-ring organoboron scaffolds for efficient photodynamic therapy targeting *Candida albicans* yeast and biofilm

Karolina A. Urbanowicz,[a] Paulina H. Marek-Urban,[a]* Karolina Wrochna,[a] Marta Rogalska,[a] Anna Lewandowska-Andrałojć,[b] Kamil Kotwica,[a] Sandra Pluczyk-Małek,[c,d] Agata Blacha-Grzechnik,[c,d] Izabela Nasiłowska,[a] Maciej Trzaskowski,[e,f] Julia Gdesz,[a] Jolanta Mierzejewska,[a]* Monika Staniszewska,[e]* Krzysztof Durka[a]*

[a]*Faculty of Chemistry, Warsaw University of Technology, Noakowskiego 3, 00-664 Warsaw, Poland.*

[b]*Faculty of Chemistry, Adam Mickiewicz University, Uniwersytetu Poznańskiego 8, Poznań, Poland*

[c]*Faculty of Chemistry, Silesian University of Technology, Strzody 9, 44-100 Gliwice, Poland*

[d]*Centre for Organic and Nanohybrid Electronics, Silesian University of Technology, Konarskiego 22B, 44-100 Gliwice, Poland*

[e]*Centre for Advanced Materials and Technologies, Warsaw University of Technology, Poleczki 19, 02-822 Warsaw, Poland*

[f]*Centre of Excellence for Health Technologies (HealthTEC), Warsaw University of Technology, Pl. Politechniki 1, 00-661 Warsaw, Poland.*

Corresponding authors:
Paulina H. Marek-Urban: paulina.urban@pw.edu.pl
Jolanta Mierzejewska: jolanta.mierzejewska@pw.edu.pl
Monika Staniszewska: monika.staniszewska@pw.edu.pl
Krzysztof Durka: krzysztof.durka@pw.edu.pl

Table of content

1. Synthesis S3
1.1 Organoboron precursors and (*N*,*N*) chelating dipyrromethene proligands S3
1.2 Synthesis of cationic **X-BDP-CAT** S6
1.3 Synthesis of zwitterionic **X-BDP-ZWIT** S10
1.4 Synthesis of unsubstituted **X-BDP** S17
1.5 Synthesis of **BF2-BDP-R** reference BODIPY complexes S19
2. X-ray crystallography S22
3. Standy-state and time-resolved spectroscopy S24
3.1 Absorption and emission spectra S24
3.2 Transient absorption experiments S28
3.3. Fluorescence decays S30
3.4. Photostability studies S34
3.5. NIR detection of singlet oxygen phosphorescence S43
4. EPR spectroscopy S44
5. Electrochemistry S45
6. ROS photogeneration S48
7. Theoretical calculations S54
8. Fluorescence microscopy images and Confocal Laser Scanning Microscopy measurements S66
9. NMR spectra of new compounds S73
9.1 NMR spectra of **X-BDP-NEt2** S73
9.2 NMR spectra of **X-BDP-CAT** S78
9.3 NMR spectra of **X-BDP-I** S87
9.4 NMR spectra of **X-BDP-CCCH2NMe2** S94
9.5 NMR spectra of **X-BDP-ZWIT** S99
9.6 NMR spectra of **X-BDP** S103
9.7 NMR spectra of **BF2-BDP-R** references S109
9.8 NMR spectra of dipyrromethene ligand and organoboron precursors S116
10. HR-MS S120
10.1 HR-MS data for **X-BDP-NEt2** S120
10.2 HR-MS data for **X-BDP-CAT** S122
10.3 HR-MS data for **X-BDP-I** S125
10.4 HR-MS data for **X-BDP-CCCH2NMe2** S127
10.5 HR-MS data for **X-BDP-ZWIT** S129
10.6 HR-MS data for **X-BDP** S132
11. HPLC traces of target compounds S134
12. References for Supporting Information S140

1. Synthesis

1.1 Organoboron precursors and (*N*,*N*) chelating dipyrromethene proligands

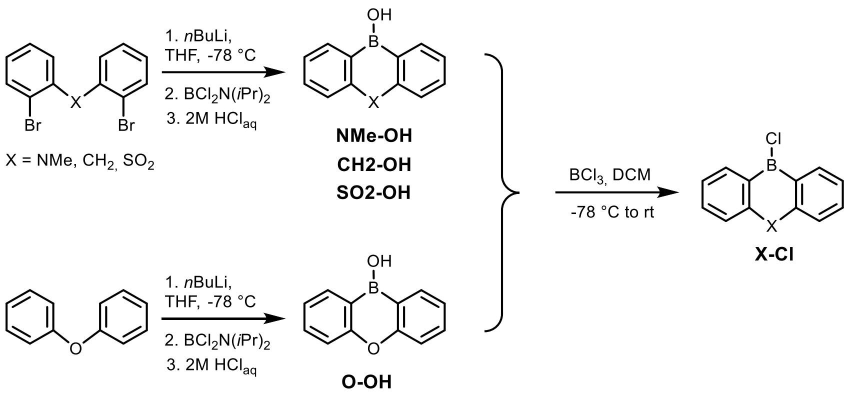

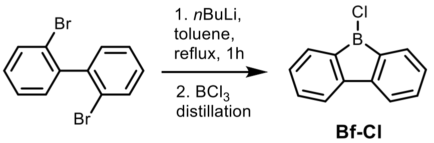

10*H*-dibenzo[*b*,*e*][1,4]oxaborinin-10-ol (O-OH).

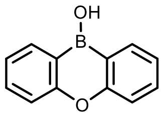

To a solution of diphenyl ether (15.24 g, 89.5 mmol) in anhydrous THF (70 ml), under an inert gas atmosphere (–78 °C), a solution of *n*BuLi (2.5 M in hexane, 76.0 ml, 190.6 mmol) was added dropwise over 20 minutes. The solution was stirred for 1 h at –78 °C and then slowly warmed to room temperature. The clear, colorless solution was stirred overnight, during which it turned dark green. The mixture was cooled to –78 °C, and a solution of $BCl_2N(iPr)_2$ (16.7 g; 92.0 mmol) in anhydrous hexane (25 ml) was added dropwise over 30 minutes, maintaining the same temperature. The solution was slowly warmed to room temperature and stirred for 2.5 h. at 30 °C, turning yellow-green. Then, 1 M HCl solution (~200 ml) was added until a pH reach 3. Et_2O (40 ml) was added and the phases were separated. The aqueous phase was washed with Et_2O (2 x 50 ml) and DCM (2 x 50 ml). The combined organic phases were dried over $MgSO_4$, and the solution was concentrated to 1/5 of the volume. Hexane (60 ml) was added to the mixture, precipitating a white solid, which was then filtered. Due to the greasy nature of the precipitate, it was mixed with dichloromethane (3 ml) and hexane (50 ml) and stirred overnight. The crude product was purified by column chromatography on silica gel with DCM eluent. The reaction yielded a white solid (7.7 g, 44 %). ^{1}H NMR (400 MHz, $CDCl_3$) δ 7.94 (ddd, *J* = 7.5, 1.8, 0.5 Hz, 2H),7.64 (ddd, *J* = 8.4, 7.1, 1.7 Hz, 2H), 7.43 (ddd, *J* = 8.4, 1.0, 0.5 Hz, 2H), 7.26 (ddd, *J* = 7.5, 7.1, 1.0 Hz, 2H) ppm. In the ^{1}H NMR spectrum, an additional set of signals with low intensity was visible, originating from the anhydride of the final compound. The ^{1}H NMR spectrum is in agreement with literature data.[1]

5-Methyldibenzo[*b*,*e*][1,4]azaborinin-10(5*H*)-ol (NMe-OH).

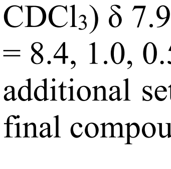

The reaction was carried out under an argon atmosphere. To a solution of bis(2-bromophenyl)methylamine (7.209 g, 21.14 mmol) in anhydrous THF (100 mL), 2.5 M *n*BuLi (16.9 ml, 42.8 mmol) in hexane was added dropwise at –78 °C. After 3 h of stirring, $BCl_2N(iPr)_2$ (5.191 g; 28.54 mmol) was added. The reaction was stirred for 1 h and allowed to slowly warm to 0 °C. Then, distilled water and 1 M HCl were added, adjusting the pH to approximately 5. The mixture was extracted with diethyl ether, and the organic phases were combined and dried over $MgSO_4$. The filtrate was concentrated and the remaining solid was washed with hexane and filtered, yielding a pale yellow solid (3.095 g, 20%). ^{1}H NMR (400 MHz, acetone-d_6+D_2O) δ = 8.25 (dd, *J* = 7.5, 1.8 Hz, 2H), 7.62 (ddd, *J* =

8.7, 6.6, 1.7 Hz, 2H), 7.55 (d, J = 8.7 Hz, 2H), 7.09 (t, J = 7.2 Hz, 2H), 3.83 (s, 3H) ppm. The ^{1}H NMR spectrum is in agreement with literature data.[2]

Dibenzo[*b*,*e*]borinin-5(10*H*)-ol (CH2-OH). The reaction was carried out under an argon atmosphere. To a cooled (–78 °C) solution of bis(2-bromophenyl)methane (3.25 g; 10 mmol) in Et_2O (100 mL), a 2.5 M solution of *n*BuLi in hexane (8.8 ml, 21.9 mmol) was added. After 30 minutes, the reaction was slowly warmed to room temperature. Then it was cooled again to –78 °C, and $BCl_2N(iPr)_2$ (2.37 g; 13 mmol) was added. The mixture was slowly warmed to room temperature and left stirring overnight. Then, 1 M HCl was added. The aqueous phase was extracted with diethyl ether. The organic phases were combined and dried over anhydrous $MgSO_4$. The solution was concentrated to a small volume and then precipitated with hexane. A white, fluffy precipitate (0.87 mg) was obtained, with a yield of 45%. ^{1}H NMR (400 MHz, $CDCl_3$ + acetone-d_6) δ = 7.97 (ddd, J = 7.5, 1.6, 0.6 Hz, 2H), 7.53 (ddd, J = 7.8, 7.1, 1.5 Hz, 2H), 7.46 (m, 2H), 7.38 (m, 2H), 5.85 (s, 1H, B-OH), 4.39 (s, 2H, CH_2) ppm. The ^{1}H NMR spectrum is in agreement with literature data.[3]

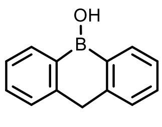

10-Hydroxy-10*H*-dibenzo[*b*,*e*][1,4]thiaborinine 5,5-dioxide (SO2-OH). A solution of 2,2'-dibromodiphenylsulfone (1.88 g, 5 mmol) in THF (50 mL) was slowly added to precooled solution of *n*-BuLi (2.5 M, 4.2 mL, 10.5 mmol) in THF (50 mL). The reaction mixture was red and after 1 h a precipitate was formed whilst the mixture turned yellow. After 3 h the reaction was cooled to –90 °C and a solution of $BCl_2N(iPr)_2$ (0.95 g, 5.2 mmol) in hexane (30 mL) was added dropwise, maintaining the temperature below –90 °C. The mixture was slowly warmed to 10 °C and stirred for 2 h at this temperature, resulting in a clear yellow solution. 1 mL of MeOH was added and the mixture discoloured. After 30 min of stirring, water (20 mL) and 1 M HCl was added to reach pH = 5. Phases were separated, the water phase was extracted with EtOAc (2 × 10 mL) and the combined organic phase was washed with water (30 mL), brine (30 mL), and dried over anhydrous Na_2SO_4. The solution was filtered and concentrated under reduced pressure to leave a solid residue. It was powdered and washed by vigorous stirring with Et_2O (2 × 10 mL), and DCM (2 × 3 mL). Then it was filtered, washed with DCM, hexane and dried under vacuum to give SO2-OH as a white powder (0.66 g, 54%). ^{1}H NMR (400 MHz, $CDCl_3$) δ = 8.20 (ddd, J = 8.0, 1.2, 0.5 Hz, 2H), 7.96 (ddd, J = 7.5, 1.5, 0.5 Hz, 2H), 7.74 (td, J = 8.0, 1.5 Hz, 2H), 7.65 (td, J = 7.5, 1.2 Hz, 2H) ppm. The ^{1}H NMR spectrum is consistent with literature data.[4]

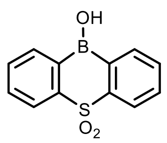

General procedure for the synthesis of dibenzo(X)borinine chlorides (X-Cl).

The synthesis was performed under an inert argon atmosphere in a 100 mL Schlenk tube. To a cooled (–78 °C) solution of borinin-10-ol in dry dichloromethane (20 mL), a solution of 1M BCl_3 in hexane (1.3 equiv.) was added dropwise. The reaction was stirred overnight at room temperature. Then, the solvents and excess of BCl_3 were evaporated under reduced pressure. The crude solid was suspended in new portion of dry dichloromethane and stirred intensively for 10 minutes. Then, after sedimentation of BCl_2OH, the solution of borinine chloride was transferred to a new Schlenk tube. The residue was washed with dry dichloromethane. The combined solutions were evaporated under reduced pressure, yielding a solid product. The product was used in subsequent reaction without further purification. The borinine chlorides are highly moisture-sensitive and undergo instantaneous hydrolysis to borinin-10-ol upon contact with trace amounts of water. Thus, all operations need to be carried out under an inert gas atmosphere.

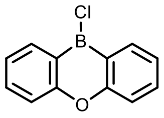

10-chloro-10*H*-dibenzo[*b*,*e*][1,4]oxaborinine (O-Cl). The compound was synthesized following the general procedure **X-Cl** using: 10*H*-dibenzo[*b*,*e*][1,4]oxaborinin-10-ol (1.45 g, 7.4 mmol) and 1M BCl_3 in hexane (9.7 mL, 9.7 mmol). The reaction yielded white solid (1.22 g, 77 %).

5-chloro-5*H*-dibenzo[*b*,*d*]borole10-chloro-5-methyl-5,10-dihydrodibenzo[*b*,*e*][1,4]azaborinine (NMe-Cl). The compound was synthesized following the general procedure **X-Cl** using: 5-methyldibenzo[*b*,*e*][1,4]azaborinin-10(5*H*)-ol (1.17 g, 5.6 mmol) and 1M BCl_3 solution in hexane (7.3 ml, 7.3 mmol). The reaction yielded green solid (0.87 g, 68 %).

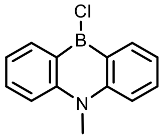

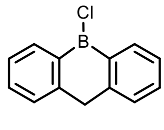

5-chloro-5,10-dihydrodibenzo[*b*,*e*]borinine (CH2-Cl). The compound was synthesized following the general procedure **X-Cl** using dibenzo[*b*,*e*]borinin-5(10*H*)-ol (0.42 g, 2.1 mmol) and 1M BCl_3 in hexane (2.8 ml, 2.8 mmol). The reaction yielded white solid (0.47 g, 72 %).

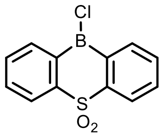

10-chloro-10*H*-dibenzo[*b*,*e*][1,4]thiaborinine 5,5-dioxide (SO2-Cl). The compound was synthesized following the general procedure **X-Cl** using 10-hydroxy-10*H*-dibenzo[*b*,*e*][1,4]thiaborinine 5,5-dioxide (0.40 g, 1.6 mmol) and 1M BCl_3 in hexane (2.1 ml, 2.1 mmol). The reaction yielded white solid (0.28 g, 58 %).

9-chloroborafluorene (Bf-Cl). A solution of *n*BuLi (2.50 M in hexane, 25.7 ml, 64.2 mmol) was added dropwise to a solution of 2,2'-dibromobiphenyl (10.0 g, 32.1 mmol) in anhydrous toluene (180 ml) at 0 °C (inert atmosphere). The resulting mixture was stirred for 1 h at 120 °C; white precipitate of the 2,2'-dilithiobiphenyl formed. The reaction mixture was slowly cooled to 0 °C, and then a 1 M solution of BCl_3 (1 M in hexane, 32.1 ml, 32.1 mmol) was added dropwise over 20 minutes. The mixture turned yellow, it was warmed to room temperature and stirred for additional 42 h. Stirring was then stopped and the mixture was allowed to stand until the lithium chloride settled. The clear, yellow solution of the product was transferred to another flask, also under an inert gas atmosphere. The lithium chloride precipitate was washed with anhydrous pentane (3 x 40 ml). The product solution was concentrated under reduced pressure, yielding an oily yellow solution. Anhydrous pentane (20 ml) was added, and the solution was filtered with the syringe filter to a smaller distillation flask. The mixture was again concentrated, leaving an oily yellow residue. Vacuum distillation was carried out at a pressure of 4.0×10^{-3} mbar and temperature of 96 °C to collect the main fraction of the product, a yellow, low-melting solid with (4.71 g, 74%). The product is stored in a Schlenk vessel with a Rotaflo stopcock under an inert gas atmosphere.

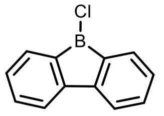

General procedure for the synthesis of Lig-R

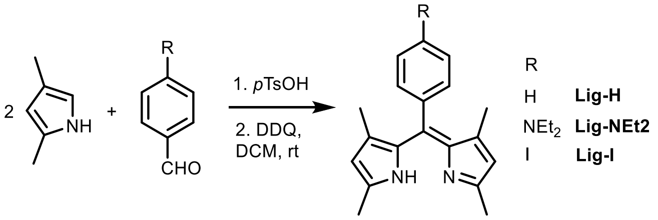

The procedure was performed under an inert gas atmosphere of argon in a 100 mL Schlenk tube. To a solution of benzaldehyde or its derivative (1 equiv.; scale ~15-20 mmol) in dry dichloromethane (30 mL) was added 2,4-dimethylpyrrole (2 equiv.). Next, the catalytic amount of *p*-toluenesulfonic acid (~50 mg) was added. The reaction was stirred overnight at room

temperature. Then, a suspension of a 2,3-dichloro-5,6-dicyano-1,4-benzoquinone – DDQ (1 equiv.) in a dry dichloromethane (20 mL) was added to the reaction. The mixture was stirred for 1 h and then evaporated to dryness yielding a dark brow solid, which was washed with hexane (10 mL) and Et_2O (10 mL). The crude product was filtered through a short Al_2O_3 column with MeOH in DCM (0% to 1% gradient). The product was used in a subsequent reaction without further purification. Dipyrromethene proligands are characterized by limited stability and should be stored under an inert atmosphere in darkness.

(Z)-2-((3,5-dimethyl-2H-pyrrol-2-ylidene)(phenyl)methyl)-3,5-dimethyl-1H-pyrrole (Lig-H). Compound was synthesized following the general procedure **Lig-R** using: benzaldehyde (1.50 g, 14.1 mmol), 2,4-dimethylpyrrole (2.69 g, 28.3 mmol) and DDQ (3.26 g, 14.1 mmol). The reaction yielded a dark brow solid (0.87 g, 32 %). ^{1}H NMR (300 MHz, $CDCl_3$): δ = 7.43−7.38 (m, 3H), 7.33−7.28 (m, 2H), 5.89 (s, 2H), 2.35 (s, 6H), 1.29 (d, J = 1.0 Hz, 6H) ppm.

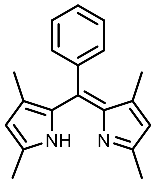

(Z)-2-((3,5-dimethyl-2H-pyrrol-2-ylidene)(4-iodophenyl)methyl)-3,5-dimethyl-1H-pyrrole (Lig-I). Compound was synthesized following the general procedure **Lig-R** using: *p*-iodobenzaldehyde (2.27 g, 9.77 mmol), 2,4-dimethylpyrrole (1.86 g, 19.5 mmol), 2,3-dichloro-5,6-dicyano-1,4-benzoquinone – DDQ (2.22 g, 9.77 mmol). The reaction yielded a dark brown solid (0.92 g, 23 %). ^{1}H NMR (400 MHz, $CDCl_3$) δ = 7.82–7.68 (m, 2H), 7.17–6.96 (m, 2H), 5.91 (s, 2H), 2.35 (s, 6H), 1.35 (s, 6H) ppm.

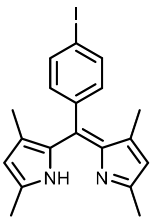

(Z)-4-((3,5-dimethyl-1H-pyrrol-2-yl)(3,5-dimethyl-2H-pyrrol-2-ylidene)methyl)-N,N-diethylaniline (Lig-NEt2). Compound was synthesized following the general procedure **Lig-R** using: *p*-(diethyloamino)benzaldehyde (1.98 g, 11.2 mmol), 2,4-dimethylpyrrole (2.13 g, 22.4 mmol), 2,3-dichloro-5,6-dicyano-1,4-benzoquinone – DDQ (2.54 g, 11.2 mmol). The reaction yielded a dark green solid (0.95 g, 25 %). ^{1}H NMR (400 MHz, $CDCl_3$) δ = 7.19-7.08 (b, 2H), 6.64 (d, J = 8.6 Hz, 2H), 6.12 (s, 2H), 3.43 (q, J = 7,1 Hz, 4H), 2.47 (b, 6H), 1.66 (b, 6H), 1.22 (t, J = 7.0 Hz, 6H) ppm.

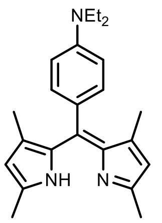

1.2 Synthesis of cationic X-BDP-CAT.

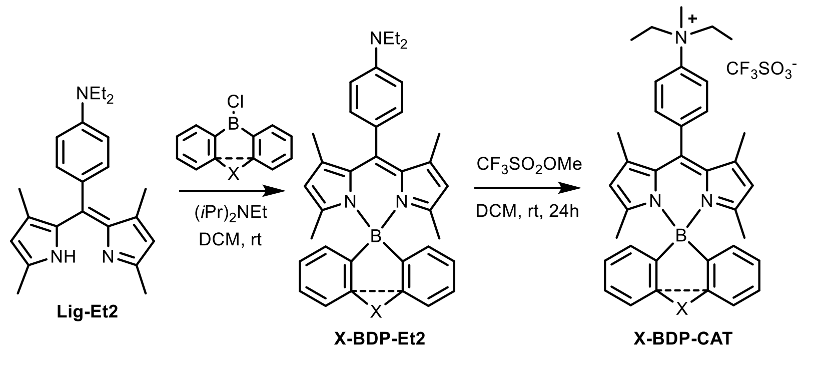

General procedure for the synthesis of X-BDP-NEt2

Lig-Et2 was dissolved in dry DCM (10 mL) and *N*,*N*-diisopropylethylamine (1 equiv. with respect to the amount of proligand) was added. The resulting solution was added to the solution of dibenzo(X)borinine chloride or 9-chloroborafluorene (1 equiv. with respect to the amount of proligand) in 10 mL DCM. The reaction was stirred overnight at room temperature. Then, the distilled water (20 mL) was added. The phases were separated, water phase was extracted three times with dichloromethane (3 × 20 mL). Combined organic phases were dried with anhydrous $MgSO_4$ and concentrated on a rotary evaporator. The crude product was purified by column chromatography on silica gel, yielding an orange solid.

***N*,*N*-diethyl-4-(1',3',7',9'-tetramethyl-4'λ^4,5λ^4-spiro[dibenzo[*b*,*d*]borole-5,5'-dipyrrolo[1,2-*c*:2',1'-*f*][1,3,2]diazaborinin]-10'-yl)aniline (Bf-BDP-NEt2).** Compound was synthesized following general procedure **X-BDP-NEt2** using: 5-chloro-5*H*-dibenzo[*b*,*d*]borole (**Bf-Cl**) (140 mg, 0.71 mmol), *N*,*N*-diisopropylethylamine (92 mg, 0.71 mmol) and (*N*,*N*)-chelating proligand (247 mg, 0.71 mmol) freshly obtained from *p*-(diethyloamino)benzaldehyde (315 mg, 1.78 mmol), 2,4-dimethylpyrrole (338 mg, 3.56 mmol) and DDQ (403 mg, 1.78 mmol). The crude **Bf-BDP-NEt2** was purified by column chromatography on silica gel (hexane:chloroform 1:1). The reaction yielded an orange solid (156 mg, 43 %).

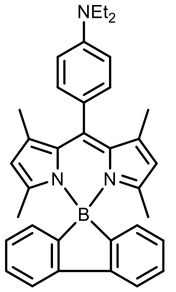

^{1}H NMR (400 MHz, $CDCl_3$) δ = 7.63 (dt, *J* = 7.2, 1.1 Hz, 2H), 7.29 – 7.20 (m, 4H), 7.22 – 7.14 (m, 2H), 7.11 (td, *J* = 7.1, 1.1 Hz, 2H), 6.86 – 6.78 (m, 2H), 5.82 (s, 2H), 3.44 (q, *J* = 7.1 Hz, 4H), 1.57 (s, 6H), 1.49 (s, 6H), 1.23 (t, *J* = 7.0 Hz, 6H) ppm. ^{13}C{^{1}H} NMR(151 MHz, $CDCl_3$) δ =153.6, 150.5, 148.2, 143.8, 140.3, 132.30, 130.3, 129.4, 127.2, 127.1, 122.7, 121.4, 118.7, 112.3, 44.5, 15.1, 14.7, 12.5 ppm. Anal. Calcd for $C_{35}H_{36}BN_3$: C, 82.51; H, 7.12; N, 8.25. Found: C, 82.35; H, 7.28; N, 8.12. HR-MS (ESI) *m*/*z* calculated for $C_{35}H_{37}BN_3$ $[M+H]^+$: 510.3075. Found: 510.3077.

***N*,*N*-diethyl-4-(1',3',7',9'-tetramethyl-4'λ^4,10λ^4-spiro[dibenzo[*b*,*e*][1,4]oxaborinine-10,5'-dipyrrolo[1,2-*c*:2',1'-*f*][1,3,2]diazaborinin]-10'-yl)aniline (O-BDP-NEt2).** Compound was synthesized following general procedure **X-BDP-NEt2** using: 10-chloro-10*H*-dibenzo[*b*,*e*][1,4]oxaborinine (**O-Cl**) (115 mg, 0.54 mmol), *N*,*N*-diisopropylethylamine (69 mg, 0.54 mmol) and (*N*,*N*)-chelating proligand (201 mg, 0.54 mmol). The crude product was purified by column chromatography on silica gel (hexane:toluene 2:3), yielding an orange solid (121 mg, 43 %). ^{1}H NMR (400 MHz, $CDCl_3$) δ = 7.27 – 7.17 (m, 4H), 7.14 (dd, *J* = 7.3, 1.8 Hz, 2H), 7.08 (dd, *J* = 8.2, 1.1 Hz, 2H), 6.94 (td, *J* = 7.2, 1.2 Hz, 2H), 6.86 – 6.78 (m, 2H), 5.81 (s, 2H), 3.44 (q, *J* = 7.1 Hz, 4H), 1.57 (s, 6H), 1.52 (s, 6H), 1.23 (t, *J* = 7.0 Hz, 6H) ppm. ^{13}C{^{1}H} NMR (101 MHz, $CDCl_3$) δ = 157.8, 154.1, 148.3, 144.0, 140.3, 133.1, 132.0, 129.4, 127.8, 122.5, 122.4, 121.4, 114.9, 112.2, 44.5, 15.4, 15.1, 12.6 ppm. Anal. Calcd for $C_{35}H_{36}BN_3O$: C, 80.00; H, 6.91; N, 8.00. Found: C, 79.84; H, 7.14; N, 7.84. HR-MS (ESI) *m*/*z* calculated for $C_{35}H_{37}BN_3O$ $[M+H]^+$: 526.3024. Found: 526.3027.

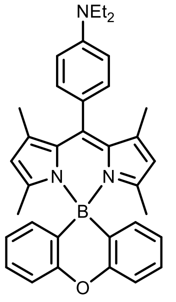

***N*,*N*-diethyl-4-(1',3',5,7',9'-pentamethyl-5*H*-4'λ^4,10λ^4-spiro[dibenzo[*b*,*e*][1,4]azaborinine-10,5'-dipyrrolo[1,2-*c*:2',1'-*f*][1,3,2]diazaborinin]-10'-yl)aniline (NMe-BDP-NEt2).**

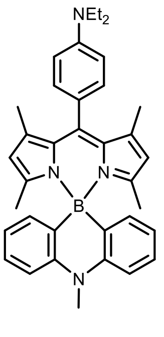

Compound was synthesized following the general procedure **X-BDP-NEt2** using: 10-chloro-5-methyl-5,10-dihydrodibenzo[*b*,*e*][1,4]azaborinine (**NMe-Cl**) (488 mg, 3.78 mmol), *N*,*N*-diisopropylethylamine (488 mg, 3.78 mmol) and (*N*,*N*)-chelating proligand (1.31 g, 3.78 mmol). The crude product was purified by column chromatography on silica gel using dichloromethane as eluent. The reaction yielded an orange solid (160 mg, 15 %). ^{1}H NMR (400 MHz, $CDCl_3$) δ = 7.26 – 7.16 (m, 4H), 7.11 (dd, *J* = 7.2, 1.7 Hz, 2H), 6.97 (d, *J* = 8.1 Hz, 2H), 6.86 – 6.74 (m, 4H), 5.78 (s, 2H), 3.65 (s, 3H), 3.44 (q, *J* = 7.1 Hz, 4H), 1.56 (s, 6H), 1.44 (s, 6H), 1.23 (t, *J* = 7.0 Hz, 6H) ppm. $^{13}C\{^1H\}$ NMR (101 MHz, $CDCl_3$) δ = 154.3, 148.2, 147.7, 143.7, 139.9, 133.3, 131.8, 129.5, 127.1, 122.9, 121.2, 119.4, 112.3, 111.6, 44.5, 35.9, 15.2, 15.1, 12.6 ppm. Anal. Calcd for $C_{36}H_{39}BN_4$: C, 80.29; H, 7.30; N, 10.40. Found: C, 80.17; H, 7.48; N, 10.30. HR-MS (ESI) *m*/*z* calculated for $C_{36}H_{39}BN_4$ $[M]^+$: 538.3262. Found: 538.3260.

***N*,*N*-diethyl-4-(1',3',7',9'-tetramethyl-10*H*-4'λ^4,5λ^4-spiro[dibenzo[*b*,*e*]borinine-5,5'-dipyrrolo[1,2-*c*:2',1'-*f*][1,3,2]diazaborinin]-10'-yl)aniline (CH2-BDP-NEt2).** Compound

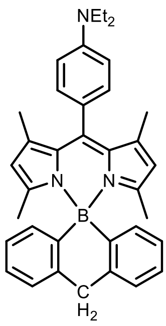

was synthesized following general procedure **X-BDP-NEt2** using: 5-chloro-5,10-dihydrodibenzo[*b*,*e*]borinine (**CH2-Cl**) (279 mg, 1.31 mmol), *N*,*N*-diisopropylethylamine (204 mg, 1.58 mmol) and (*N*,*N*)-chelating proligand (456 mg, 1.31 mmol). The crude product was purified by column chromatography on silica gel (hexane:ethyl acetate 9:1). The reaction yielded an orange solid (305 mg, 44 %). ^{1}H NMR (400 MHz, $CDCl_3$) δ = 7.25 – 7.18 (m, 4H), 7.19 – 7.11 (m, 4H), 7.11 – 7.03 (m, 2H), 6.87 – 6.78 (m, 2H), 5.78 (s, 2H), 4.43 (s, 2H), 3.44 (q, *J* = 7.0 Hz, 4H), 1.57 (s, 6H), 1.46 (s, 6H), 1.23 (t, *J* = 7.0 Hz, 6H) ppm. $^{13}C\{^1H\}$ NMR (101 MHz, $CDCl_3$) δ = 153.6, 148.2, 143.9, 141.9, 140.0, 132.6, 132.0, 129.4, 126.4, 125.9, 125.9, 122.8, 121.2, 112.3, 44.5, 37.6, 15.2, 15.1, 12.6 ppm. Anal. Calcd for $C_{36}H_{38}BN_3$: C, 82.59; H, 7.32; N, 8.03. Found: C, 82.65; H, 7.30; N, 7.97. HR-MS (ESI) *m*/*z* calculated for $C_{36}H_{39}BN_3$: $[M+H]^+$: 524.3232. Found: 524.3230.

10'-(4-(diethylamino)phenyl)-1',3',7',9'-tetramethyl-4'λ^4,10λ^4-spiro[dibenzo[*b*,*e*][1,4]thiaborinine-10,5'-dipyrrolo[1,2-*c*:2',1'-*f*][1,3,2]diazaborinine]

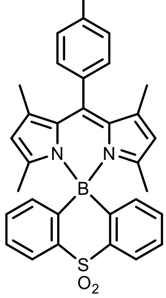

5,5-dioxide (SO2-BDP-NEt2). Compound was synthesized following general procedure **X-BDP-NEt2** using: 10-chloro-10*H*-dibenzo[*b*,*e*][1,4]thiaborinine 5,5-dioxide (**SO2-Cl**) (248 mg, 0.95 mmol), *N*,*N*-diisopropylethylamine (122 mg, 0.95 mmol) and (N,N)-chelating proligand (327 mg, 0.95 mmol). The crude product was purified by column chromatography on silica gel (hexane:toluene 3:2). The reaction yielded an orange solid (172 mg, 32 %). ^{1}H NMR (400 MHz, $CDCl_3$) δ = 8.07 (dd, *J* = 7.9, 1.3 Hz, 2H), 7.45 (td, *J* = 7.6, 1.5 Hz, 2H), 7.38 (td, *J* = 7.4, 1.3 Hz, 2H), 7.25 – 7.15 (m, 4H), 6.87 – 6.79 (m, 2H), 5.84 (s, 2H), 3.45 (q, *J* = 7.1 Hz, 4H), 1.66 (s, 6H), 1.59 (s, 6H), 1.24 (t, *J* = 7.0 Hz, 6H) ppm. $^{13}C\{^1H\}$ NMR (101 MHz, $CDCl_3$) δ = 154.3, 148.4, 144.8, 144.2, 141.6, 133.0, 131.9, 131.9, 129.3, 128.3, 122.2, 121.9, 121.7, 112.2, 44.5, 29.8, 15.8, 15.3, 12.5 ppm. Anal. Calcd for $C_{35}H_{36}BN_3O_2S$: C, 73.29; H, 6.33; N, 7.33. Found: C, 73.05; H, 6.51; N, 7.09. HR-MS (ESI) *m*/*z* calculated for $C_{35}H_{37}BN_3O_2S$ $[M+H]^+$: 574.2694. Found 574.2693.

General procedure for the synthesis of X-BDP-CAT

The synthesis was performed under an inert gas atmosphere of argon in a 25 mL Schlenk tube. To a solution of **X-BDP-NEt2** (1 equiv.; reaction scale ~0.2-0.4 mmol) in dry dichloromethane (10 mL) was added methyl triflate (1 equiv.) at room temperature. The reaction was stirred overnight. The solvent was concentrated to a volume of 1-2 mL and the solid was precipitated using diethyl ether (about 3-4 mL).

***N*,*N*-diethyl-*N*-methyl-4-(1',3',7',9'-tetramethyl-4'λ^{4},5λ^{4}-spiro[dibenzo[*b*,*d*]borole-5,5'-dipyrrolo[1,2-*c*:2',1'-*f*][1,3,2]diazaborinin]-10'-yl)benzenaminium triflate (Bf-BDP-CAT).** The compound was synthesized following the general procedure **X-BDP-CAT** using: **Bf-BDP-NEt2** (96 mg, 0.19 mmol) and methyl triflate (155 mg, 0.94 mmol). The reaction yielded an orange solid (89 mg, 70 %). ^{1}H NMR (400 MHz, $CDCl_3$) δ = 7.91 – 7.88 (m, 2H), 7.80 (m, 2H), 7.65 – 7.61 (dt, *J* = 7.1, 0.9 Hz, 2H), 7.29 – 7.21 (m, 4H), 7.12 (td, *J* = 7.3, 1.1 Hz, 2H), 5.92 – 5.73 (m, 2H), 4.26 (qd, *J* = 13.5, 6.7 Hz, 4H), 3.67 (s, 2H), 1.51 (s, 6H), 1.39 (s, 6H), 1.21 (t, *J* = 7.1 Hz, 6H) ppm. ^{13}C{^{1}H} NMR (101 MHz, $CDCl_3$) δ = 155.6, 150.6, 140.8, 139.4, 138.9, 132.9, 131.9, 130.8, 130.2, 127.6, 127.4, 122.8, 122.5, 119.0, 65.3, 45.6, 15.0, 14.9, 8.8 ppm. ^{19}F NMR (376 MHz, $CDCl_3$) δ = –78.40 ppm. Anal. Calcd for $C_{37}H_{39}BF_3N_3O_3S$: C, 65.97; H, 5.84; N, 6.24. Found: C, 66.19; H, 5.70; N, 5.99. HR-MS (ESI) *m*/*z* calculated for $C_{36}H_{39}BN_3$ $[M]^+$: 524.3232. Found: 524.3237.

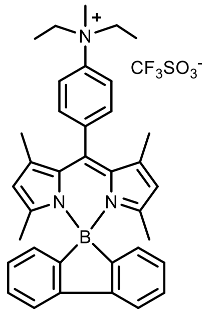

***N*,*N*-diethyl-*N*-methyl-4-(1',3',7',9'-tetramethyl-4'λ^{4},10λ^{4}-spiro[dibenzo[*b*,*e*][1,4]oxaborinine-10,5'-dipyrrolo[1,2-*c*:2',1'-*f*][1,3,2]diazaborinin]-10'-yl)benzenaminium triflate (O-BDP-CAT).** Compound was synthesized following the general procedure **X-BDP-CAT** using: **O-BDP-NEt2** (121 mg, 0.23 mmol) and methyl triflate (189 mg, 1.15 mmol). The reaction yielded an orange solid (41 mg, 26 %). ^{1}H NMR (400 MHz, DMSO-d_6) δ = 8.10 – 8.02 (m, 2H), 7.97 – 7.89 (m, 2H), 7.25 (ddd, *J* = 8.3, 7.0, 1.8 Hz, 2H), 7.11 – 7.04 (m, 4H), 6.95 (td, *J* = 7.2, 1.1 Hz, 2H), 6.01 (d, *J* = 1.0 Hz, 2H), 4.16 (dq, *J* = 14.0, 7.0 Hz, 2H), 3.92 (dq, *J* = 14.0, 7.0 Hz, 2H), 3.58 (s, 3H), 1.44 (s, 6H), 1.39 (s, 6H), 1.06 (t, *J* = 7.0 Hz, 6H) ppm. ^{13}C{^{1}H} NMR (101 MHz, DMSO-d_6) δ = 157.0, 154.2, 141.2, 140.3, 139.2, 136.9, 132.7, 130.6, 130.2, 128.3, 123.3, 122.4, 122.1, 114.6, 63.8, 54.9, 14.7, 14.3, 8.2 ppm. ^{19}F NMR (376 MHz, DMSO-d_6) δ = –77.75 ppm. Anal. Calcd for $C_{37}H_{39}BF_3N_3O_4S$: C, 64.44; H, 5.70; N, 6.09. Found: C, 64.61; H, 5.59; N, 6.15. HR-MS (ESI) *m*/*z* calculated for $C_{36}H_{39}BN_3O$ $[M]^+$: 540.3181. Found: 540.3180.

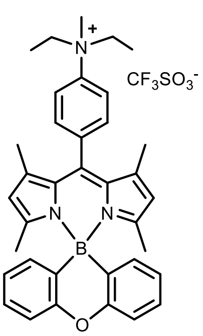

***N*,*N*-diethyl-*N*-methyl-4-(1',3',5,7',9'-pentamethyl-5*H*-4'λ^{4},10λ^{4}-spiro[dibenzo[*b*,*e*][1,4]azaborinine-10,5'-dipyrrolo[1,2-*c*:2',1'-*f*][1,3,2]diazaborinin]-10'-yl)benzenaminium triflate (NMe-BDP-CAT).** Compound was synthesized following the general procedure **X-BDP-CAT** using: **NMe-BDP-NEt2** (198 mg, 0.37 mmol) and methyl triflate (287 mg, 1.75 mmol). The reaction yielded an orange solid (207 mg, 84 %). ^{1}H NMR (400 MHz, $CDCl_3$) δ = 7.89 (m, 2H), 7.82 (m, 2H), 7.29 – 7.20 (m, 2H), 7.09 (dd, *J* = 7.2, 1.8 Hz, 2H), 6.99 (d, *J* = 8.3 Hz, 2H), 6.81 (t, *J* = 7.1 Hz, 2H), 5.82 (d, *J* = 1.0 Hz, 2H), 4.35 – 4.17 (m, 4H), 3.67 (s, 3H), 3.66 (s, 3H), 1.46 (s, 6H), 1.39 (s, 6H), 1.21 (t, *J* = 7.0 Hz, 6H) ppm. ^{13}C{^{1}H} NMR (101 MHz,

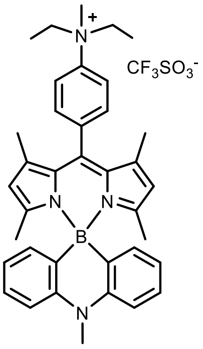

CDCl3) δ = 156.3, 147.7, 140.8, 139.5, 138.6, 138.2, 133.1, 131.9, 130.2, 127.5, 122.6, 122.5, 119.5, 111.9, 65.2, 45.7, 35.9, 15.3, 14.9, 8.8 ppm. ^{19}F NMR (376 MHz, CDCl3) δ = –78.41 ppm. Anal. Calcd for $C_{38}H_{42}BF_3N_4O_3S$: C, 64.96; H, 6.03; N, 7.97. Found: C, 65.08; H, 6.11; N, 7.83. HR-MS (ESI) *m*/*z* calculated for $C_{37}H_{42}BN_4$ [M]$^+$: 553.3497. Found: 553.3498.

***N*,*N*-diethyl-*N*-methyl-4-(1',3',7',9'-tetramethyl-10*H*-4'λ^4,5λ^4-spiro[dibenzo[b,e]borinine-5,5'-dipyrrolo[1,2-*c*:2',1'-*f*][1,3,2]diazaborinin]-10'-yl)benzenaminium triflate (CH2-BDP-CAT).** The compound was synthesized following the general procedure **X-BDP-CAT** using: **CH2-BDP-NEt2** (131 mg, 0.25 mmol) and methyl triflate (205 mg, 1.25 mmol). The reaction yielded an orange solid (147 mg, 86 %). ^{1}H NMR (400 MHz, DMSO-*d*6) δ = 8.07 (m, 2H), 7.94 (m, 2H), 7.25 (d, *J* = 7.7 Hz, 2H), 7.22 – 7.11 (m, 2H), 7.11 – 7.02 (m, 4H), 5.98 (s, 2H), 4.40 (s, 2H), 4.18 (dq, *J* = 14.3, 7.0 Hz, 2H), 3.94 (dq, *J* = 13.7, 6.8 Hz, 2H), 3.60 (s, 3H), 1.40 (s, 12H), 1.07 (t, *J* = 7.0 Hz, 6H) ppm. ^{19}F NMR (376 MHz, DMSO-*d*6) δ = –77.78 ppm. Anal. Calcd for $C_{38}H_{41}BF_3N_3O_3S$: C, 66.38; H, 6.01; N, 6.11. Found: C, 66.18; H, 5.79; N, 5.94. HR-MS (ESI) *m*/*z* calculated for $C_{37}H_{41}BN_3$ [M]$^+$: 538.3388. Found: 538.3386. ^{13}C NMR spectrum could not be obtained due to the insufficient solubility of the compound in the available deuterated solvents.

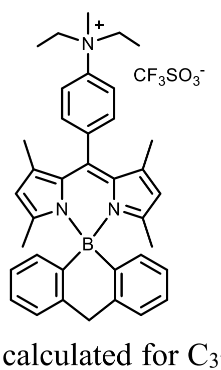

***N*,*N*-diethyl-*N*-methyl-4-(1',3',7',9'-tetramethyl-5,5-dioxido-4'λ^4,10λ^4-spiro[dibenzo[*b*,*e*][1,4]thiaborinine-10,5'-dipyrrolo[1,2-*c*:2',1'-*f*][1,3,2]diazaborinin]-10'-yl)benzenaminium triflate (SO2-BDP-CAT).** The compound was synthesized following the general procedure **X-BDP-CAT** using: **SO2-BDP-NEt2** (172 mg, 0.30 mmol) and methyl triflate (247 mg, 1.50 mmol). The reaction yielded an orange solid (63 mg, 28 %). ^{1}H NMR (400 MHz, acetone-*d*6) δ = 8.30 (m, 2H), 8.08 (m, 2H), 7.98 (d, *J* = 8.0 Hz, 2H), 7.55 (t, *J* = 7.7 Hz, 2H), 7.45 (t, *J* = 7.4 Hz, 2H), 7.29 (d, *J* = 7.6 Hz, 2H), 6.05 (s, 2H), 4.47 (dq, *J* = 14.3, 7.2 Hz, 2H), 4.25 (dq, *J* = 13.7, 7.0 Hz, 2H), 3.92 (s, 3H), 1.65 (s, 6H), 1.51 (s, 6H), 1.34 (t, *J* = 7.1 Hz, 6H) ppm. ^{19}F NMR (376 MHz, acetone-*d*6) δ = –78.86 ppm. Anal. Calcd for $C_{37}H_{39}BF_3N_3O_5S_2$: C, 60.25; H, 5.33; N, 5.70. Found: C, 60.23; H, 5.29; N, 5.61. HR-MS (ESI) *m*/*z* calculated for $C_{36}H_{39}BN_3O_2S$ [M]$^+$: 588.2851 Found: 588.2849. ^{13}C NMR spectrum could not be obtained due to the insufficient solubility of the compound in the available deuterated solvents.

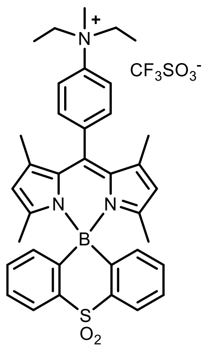

1.3 Synthesis of zwitterionic X-BDP-ZWIT

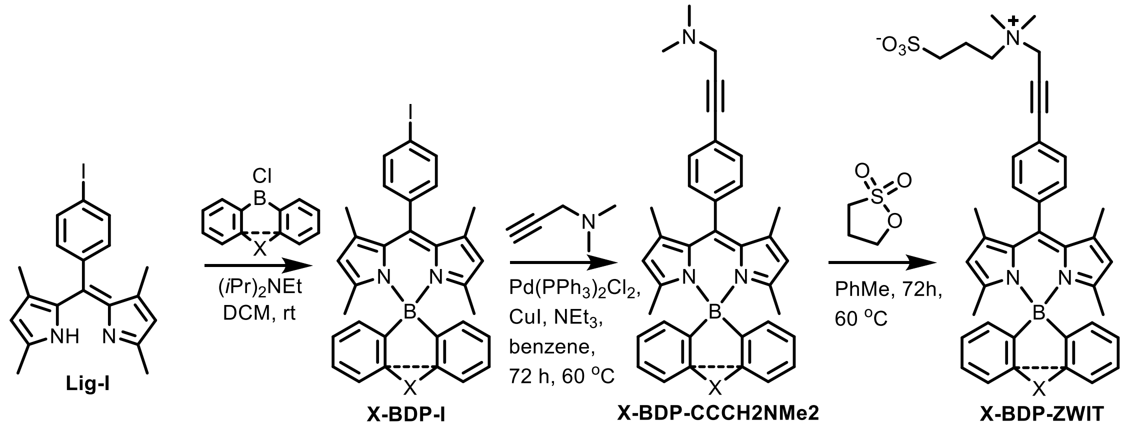

General procedure for the synthesis of X-BDP-I

Lig-I was dissolved in dry DCM (10 mL) and *N*,*N*-diisopropylethylamine (1 equiv. with respect to the amount of proligand) was added. The resulting solution was added to the solution of dibenzo(X)borinine chloride or 9-chloroborafluorene (1 equiv. with respect to the amount of proligand) in 10 mL dry DCM. The reaction was stirred overnight at room temperature. Then, the distilled water (20 mL) was added. The phases were separated, water phase was extracted three times with dichloromethane (3 × 20 mL). Combined organic phases were dried with anhydrous $MgSO_4$ and concentrated on a rotary evaporator. The crude product was purified by column chromatography on silica gel, yielding an orange solid.

10'-(4-iodophenyl)-1',3',7',9'-tetramethyl-4'λ^4,5λ^4-spiro[dibenzo[*b*,*d*]borole-5,5'-dipyrrolo[1,2-*c*:2',1'-*f*][1,3,2]diazaborinine] (Bf-BDP-I). Compound was synthesized following the general procedure **X-BDP-I** using: 5-chloro-5*H*-dibenzo[*b*,*d*]borole (**Bf-Cl**) (123 mg, 0.62 mmol), *N*,*N*-diisopropylamine (80 mg, 0.62 mmol) (*N*,*N*)-chelating proligand (250 mg, 0.62 mmol) freshly obtained from *p*-(diethyloamino)benzaldehyde (315 mg, 1.78 mmol), 2,4-dimethylpyrrole (338 mg, 3.56 mmol) and DDQ (403 mg, 1.78 mmol); the proligand yield was 35% (210 mg, 0.62 mmol). The crude **Bf-BDP-I** was purified by column chromatography on silica gel (hexane:toluene 3:2). The reaction yielded an orange solid (186 mg, 53 %). ^{1}H NMR (400 MHz, $CDCl_3$) δ = 7.92 – 7.88 (m, 2H), 7.65 – 7.60 (m, 2H), 7.28 – 7.19 (m, 6H), 7.10 (td, *J* = 7.1, 1.0 Hz, 2H), 5.83 (s, 2H), 1.49 (s, 6H), 1.46 (s, 6H) ppm. $^{13}C\{^1H\}$ NMR (101 MHz, $CDCl_3$) δ = 154.6, 150.4, 140.5, 139.7, 138.2, 135.6, 132.5, 130.9, 130.4, 129.3, 127.3, 121.9, 118.7, 94.4, 15.0, 14.6 ppm. Anal. Calcd for $C_{31}H_{26}BIN_2$: C, 65.99; H, 4.64; N, 4.96. Found: C, 65.72; H, 4.86; N, 4.67. HR-MS (ESI) *m*/*z* calculated for $C_{31}H_{27}BIN_2$ $[M+H]^+$: 565.1307. Found: 565.1309.

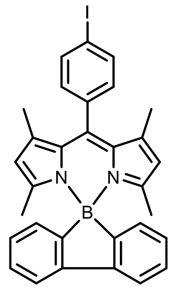

10'-(4-iodophenyl)-1',3',7',9'-tetramethyl-4'λ^4,10λ^4-spiro[dibenzo[*b*,*e*][1,4]oxaborinine-10,5'-dipyrrolo[1,2-*c*:2',1'-*f*][1,3,2]diazaborinine] (O-BDP-I). Compound was synthesized following the general procedure **X-BDP-I** using: 10-chloro-10*H*-dibenzo[*b*,*e*][1,4]oxaborinine (**O-Cl**) (561 mg, 2.61 mmol), *N*,*N*-diisopropylamine (338 mg, 2.61 mmol) and (*N*,*N*)-chelating proligand (1.05 g, 2.61 mmol). The crude product was purified by column chromatography on silica gel (hexane:toluene 1:1). The reaction yielded an orange solid (921 mg, 61 %). ^{1}H NMR (400 MHz, $CDCl_3$) δ = 7.96 – 7.88 (m, 2H), 7.28 – 7.21 (m, 5H), 7.15 – 7.05 (m, 4H), 6.94 (td, *J* = 7.3, 1.0 Hz, 2H), 5.83 (s, 2H), 1.52 (s, 6H), 1.47 (s, 6H) ppm. $^{13}C\{^1H\}$ NMR (101 MHz, $CDCl_3$) δ = 157.8, 155.3, 140.7, 139.9, 138.4, 138.0, 135.7, 132.9, 130.7, 130.6, 129.2, 128.4, 128.1, 125.4, 122.5, 122.1, 115.1, 94.6, 21.6, 15.5, 15.1 ppm. Anal. Calcd for $C_{31}H_{26}BIN_2O$: C, 64.17; H, 4.52; N, 4.83. Found: C, 63.92; H, 4.70; N, 4.63. HR-MS (ESI) *m*/*z* calculated for $C_{31}H_{27}BIN_2O$ $[M+H]^+$: 581.1255. Found 581.1254.

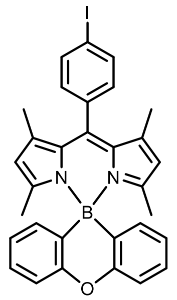

10'-(4-iodophenyl)-1',3',5,7',9'-pentamethyl-5*H*-4'λ⁴,10λ⁴-spiro[dibenzo[*b*,*e*][1,4]azaborinine-10,5'-dipyrrolo[1,2-*c*:2',1'-*f*][1,3,2]diazaborinine]

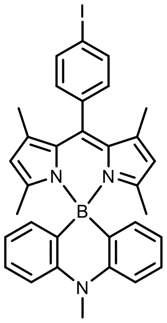

(NMe-BDP-I). Compound was synthesized following the general procedure **X-BDP-I** using: 10-chloro-5-methyl-5,10-dihydrodibenzo[*b*,*e*][1,4]azaborinine (**NMe-Cl**) (340 mg, 1.50 mmol), *N*,*N*-diisopropylethylamine (193 mg, 1.50 mmol) and (*N*,*N*)-chelating proligand (600 mg, 1.50 mmol). The crude product was purified by column chromatography on silica gel (hexane:toluene 3:2). The reaction yielded an orange solid (267 mg, 30 %). ^{1}H NMR (400 MHz, $CDCl_3$) δ = 7.94 – 7.87 (m, 2H), 7.26 – 7.20 (m, 4H), 7.08 (dd, *J* = 7.2, 1.8 Hz, 2H), 6.98 (dd, *J* = 8.4, 1.1 Hz, 2H), 6.79 (td, *J* = 7.2, 1.0 Hz, 2H), 5.80 (d, *J* = 1.0 Hz, 2H), 3.65 (s, 3H), 1.47 (d, *J* = 0.8 Hz, 6H), 1.45 (s, 6H) ppm. ^{13}C{^{1}H} NMR (101 MHz, $CDCl_3$) δ = 155.5, 147.7, 140.5, 139.4, 138.3, 136.0, 133.2, 130.7, 130.5, 127.3, 121.9, 119.4, 111.8, 94.5, 35.9, 15.3, 15.1 ppm. Anal. Calcd for $C_{32}H_{29}BIN_3$: C, 64.78; H, 4.93; N, 7.08. Found: C, 64.61; H, 5.04; N, 6.90. HR-MS (ESI) *m*/*z* calculated for $C_{32}H_{29}BIN_3$ $[M]^+$: 593.1494. Found 593.1489.

10'-(4-iodophenyl)-1',3',7',9'-tetramethyl-10*H*-4'λ⁴,5λ⁴-spiro[dibenzo[*b*,*e*]borinine-5,5'-dipyrrolo[1,2-*c*:2',1'-*f*][1,3,2]diazaborinine] (CH2-BDP-I). Compound was synthesized

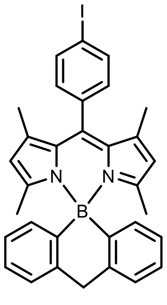

following general procedure **X-BDP-I** using: 5-chloro-5,10-dihydrodibenzo[*b*,*e*]borinine (**CH2-Cl**) (674 mg, 3.17 mmol), and (*N*,*N*)-chelating proligand (471 mg, 3.65 mmol) and *N*,*N*-diisopropylethylamine (471 mg, 3.65 mmol). The crude product was purified by column chromatography on silica gel (hexane:toluene 3:2). The reaction yielded an orange solid (369 mg, 20 %). ^{1}H NMR (400 MHz, $CDCl_3$) δ = 7.96 – 7.89 (m, 2H), 7.31 – 7.24 (m, 2H), 7.27 – 7.20 (m, 2H), 7.21 – 7.12 (m, 3H), 7.15 – 7.04 (m, 3H), 5.80 (s, 2H), 4.44 (s, 2H), 1.48 (s, 6H), 1.48 (s, 6H) ppm. ^{13}C{^{1}H} NMR (101 MHz, $CDCl_3$) δ = 154.7, 141.9, 140.7, 139.6, 138.3, 135.9, 132.5, 130.8, 130.7, 126.6, 126.1, 125.9, 121.9, 94.5, 37.5, 15.2, 15.2 ppm. Anal. Calcd for $C_{32}H_{28}BIN_2$: C, 66.46; H, 4.88; B, N, 4.84. Found: C, 66.28; H, 4.57; B, N, 4.60. HR-MS (ESI) *m*/*z* calculated for $C_{32}H_{29}BIN_2$ $[M+H]^+$: 579.1463. Found: 579.1461.

10'-(4-iodophenyl)-1',3',7',9'-tetramethyl-4'λ⁴,10λ⁴-spiro[dibenzo[*b*,*e*][1,4]thiaborinine-10,5'-dipyrrolo[1,2-*c*:2',1'-*f*][1,3,2]diazaborinine] 5,5-dioxide (SO2-BDP-I). Compound

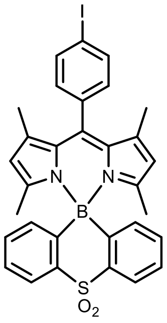

was synthesized following general procedure **X-BDP-I** using: 10-chloro-10*H*-dibenzo[*b*,*e*][1,4]thiaborinine 5,5-dioxide (**SO2-Cl**) (309 mg, 1.12 mmol), *N*,*N*-diisopropylethylamine (152 mg, 1.12 mmol) and (*N*,*N*)-chelating proligand (448 mg, 1.12 mmol). The crude product was purified by column chromatography on silica gel using chloroform as eluent. The reaction yielded an orange solid (256 mg, 35 %). ^{1}H NMR (400 MHz, $CDCl_3$) δ = 8.08 (dd, *J* = 8.0, 1.3 Hz, 2H), 7.99 – 7.91 (m, 2H), 7.47 (ddd, *J* = 8.0, 7.3, 1.4 Hz, 2H), 7.39 (td, *J* = 7.4, 1.3 Hz, 2H), 7.27 – 7.23 (m, 2H), 7.19 (ddd, *J* = 7.4, 1.4, 0.5 Hz, 2H), 5.86 (d, *J* = 1.0 Hz, 2H), 1.66 (s, 6H), 1.49 (d, *J* = 0.9 Hz, 6H) ppm. ^{13}C{^{1}H} NMR (101 MHz, $CDCl_3$) δ = 155.5, 144.9, 141.2, 140.9, 138.6, 135.1, 132.8, 131.9, 130.7, 130.4, 128.5, 122.6, 122.3, 95.0, 31.1, 15.8, 15.2 ppm. Anal. Calcd for $C_{31}H_{26}BIN_2O_2S$: C, 59.26; H, 4.17; N, 4.46. Found: C, 59.60; H, 4.49; N, 4.10. HR-MS (ESI) *m*/*z* calculated for $C_{31}H_{27}BIN_2O_2S$ $[M+H]^+$: 629.0926. Found: 629.0924.

General procedure for the synthesis of X-BDP-CCCH2NMe2

The procedure was performed under an inert gas atmosphere of argon in a 50 mL Schlenk tube. To a solution of **X-BDP-I** (1 equiv.; reaction scale 0.2-0.5 mmol) in benzene (5 mL) and triethylamine (5 mL), CuI (0.05 equiv.) and [Pd(PPh_3)$_2$$Cl_2$] (0.05 equiv.) were added. After stirring for 10 minutes, the 1-dimethylamino-2-propyne (2.3 equiv.) was added. The reaction was stirred for 72 h at 60 ºC. The substrate consumption was monitored by TLC. The reaction was cooled down to room temperature. The mixture was filtered through the celite pad. The solution was washed with distilled water and water phase was then extracted with dichloromethane (3 x 20 mL). The organic phases were combined and dried with anhydrous $MgSO_4$. After filtration, the solvents were evaporated under reduced pressure. The crude product was purified with column chromatography on silica gel, yielding an orange solid of **X-BDP- CCCH2NMe2**.

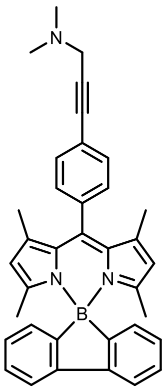

***N*,*N*-dimethyl-3-(4-(1',3',7',9'-tetramethyl-4'λ^4,5λ^4-spiro[dibenzo[*b*,*d*]borole-5,5'-dipyrrolo[1,2-*c*:2',1'-*f*][1,3,2]diazaborinin]-10'-yl)phenyl)prop-2-yn-1-amine (Bf-BDP-CCCH2NMe2).** Compound was synthesized following the general procedure **X-BDP-CCCH2NMe2** using: **Bf-BDP-I** (164 mg, 0.29 mmol), CuI (3 mg, 0.02 mmol), a [Pd(PPh_3)$_2$$Cl_2$] (10 mg, 0.02 mmol) and 1-dimethylamino-2-propyne (458 mg, 0.66 mmol). The crude product was purified with column chromatography on silica gel using 10 % methanol in dichloromethane as eluent. The reaction yielded an orange solid (106 mg, 70 %). ^{1}H NMR (400 MHz, $CDCl_3$) δ = 7.66 – 7.59 (m, 4H), 7.44 – 7.38 (m, 2H), 7.24 (d, *J* = 7.1 Hz, 4H), 7.11 (t, *J* = 7.1 Hz, 2H), 5.83 (s, 2H), 3.53 (s, 2H), 2.43 (s, 6H), 1.49 (s, 6H), 1.45 (s, 6H) ppm. ^{13}C{^{1}H} NMR (151 MHz, $CDCl_3$) δ = 154.6, 150.5, 141.4, 140.0, 135.9, 132.4, 131.2, 130.2, 128.7, 127.4, 127.2, 123.9, 122.0, 118.8, 86.2, 84.8, 48.8, 44.5, 15.0, 14.8 ppm. Anal. Calcd for $C_{36}H_{34}BN_3$: C, 83.23; H, 6.60; N, 8.09. Found: C, 83.00; H, 6.95; N, 7.72. HR-MS (ESI) *m*/*z* calculated for $C_{36}H_{34}BN_3$ $[M]^+$: 518.2762. Found: 518.2768.

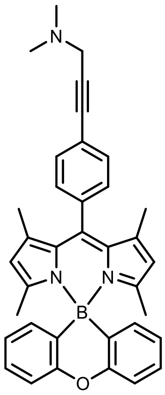

***N*,*N*-dimethyl-3-(4-(1',3',7',9'-tetramethyl-4'λ^4,10λ^4-spiro[dibenzo[*b*,*e*][1,4]oxaborinine-10,5'-dipyrrolo[1,2-*c*:2',1'-*f*][1,3,2]diazaborinin]-10'-yl)phenyl)prop-2-yn-1-amine (O-BDP-CCCH2NMe2).** Compound was synthesized following the general procedure **X-BDP-CCCH2NMe2** using: **O-BDP-I** (603 mg, 1.04 mmol), CuI (10 mg, 0.05 mmol), [Pd(PPh_3)$_2$$Cl_2$] (36 mg, 0.05 mmol) and 1-dimethylamino-2-propyne (194 mg, 2.34 mmol). The crude product was purified with column chromatography on silica gel using 10 % methanol in dichloromethane as eluent. The reaction yielded an orange solid (184 mg, 33 %). ^{1}H NMR (400 MHz, $CDCl_3$) δ = 7.68 – 7.61 (m, 2H), 7.47 – 7.40 (m, 2H), 7.23 (ddd, *J* = 8.6, 7.0, 1.8 Hz, 2H), 7.13 (dd, *J* = 7.3, 1.8 Hz, 2H), 7.09 (dd, *J* = 8.2, 1.1 Hz, 2H), 6.94 (td, *J* = 7.2, 1.1 Hz, 2H), 5.82 (s, 2H), 3.54 (s, 2H), 2.44 (s, 6H), 1.53 (s, 6H), 1.48 – 1.42 (m, 6H) ppm. ^{13}C{^{1}H} NMR (101 MHz, $CDCl_3$) δ = 157.8, 155.1, 141.5, 140.0, 135.9, 133.0, 132.5, 130.8, 128.7, 128.0, 123.9, 122.5, 122.0, 115.0, 86.1, 84.9, 48.8, 44.5, 15.5, 15.0 ppm. Anal. Calcd for $C_{36}H_{34}BN_3O$: C, 80.75; H, 6.40; N, 7.85. Found: C, 80.54; H, 6.69; N, 7.71. HR-MS (ESI) *m*/*z* calculated for $C_{36}H_{34}BN_3O$ $[M]^+$: 535.2795. Found: 535.2789.

***N*,*N*-dimethyl-3-(4-(1',3',5,7',9'-pentamethyl-5*H*-4'λ^4,10λ^4-spiro[dibenzo[*b*,*e*][1,4]azaborinine-10,5'-dipyrrolo[1,2-*c*:2',1'-*f*][1,3,2]diazaborinin]-10'-yl)phenyl)prop-2-yn-1-amine (NMe-BDP-CCCH2NMe2).**

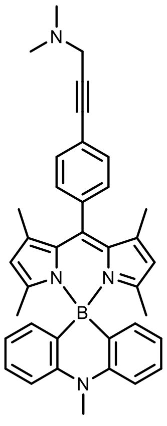

Compound was synthesized following the general procedure **X-BDP-CCCH2NMe2** using: **NMe-BDP-I** (634 mg, 1.07 mmol), CuI (10 mg, 0.05 mmol), $[Pd(PPh_3)_2Cl_2]$ (38 mg, 0.05 mmol) and 1-dimethylamino-2-propyne (200 mg, 2.40 mmol). The crude product was purified with column chromatography on silica gel using 10 % methanol in dichloromethane as eluent. The reaction yielded a red solid (288 mg, 49 %). ^{1}H NMR (400 MHz, $CDCl_3$) δ = 7.68 – 7.60 (m, 2H), 7.48 – 7.40 (m, 2H), 7.23 (ddd, *J* = 8.3, 7.1, 1.8 Hz, 2H), 7.10 (dd, *J* = 7.2, 1.8 Hz, 2H), 6.98 (d, *J* = 8.0 Hz, 2H), 6.79 (td, *J* = 7.1, 1.0 Hz, 2H), 5.80 (d, *J* = 1.0 Hz, 2H), 3.66 (s, 3H), 3.55 (s, 2H), 2.44 (s, 6H), 1.46 (d, *J* = 0.9 Hz, 6H), 1.45 (s, 6H) ppm. ^{13}C{^{1}H} NMR (101 MHz, $CDCl_3$) δ = 155.3, 147.7, 141.3, 139.5, 136.2, 133.2, 132.4, 130.6, 128.8, 127.3, 123.7, 121.8, 119.4, 111.7, 85.8, 85.1, 48.8, 44.5, 35.9, 15.2, 15.0 ppm. Anal. Calcd for $C_{37}H_{37}BN_4$: C, 81.02; H, 6.80; N, 10.21. Found: C, 80.86; H, 7.03; N, 10.08. HR-MS (ESI) *m*/*z* calculated for $C_{37}H_{38}BN_4$ $[M+H]^+$: 549.3184. Found: 549.3180.

***N*,*N*-dimethyl-3-(4-(1',3',7',9'-tetramethyl-10*H*-4'λ^4,5λ^4-spiro[dibenzo[*b*,*e*]borinine-5,5'-dipyrrolo[1,2-*c*:2',1'-*f*][1,3,2]diazaborinin]-10'-yl)phenyl)prop-2-yn-1-amine (CH2-BDP-CCCH2NMe2).**

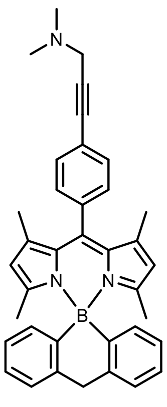

Compound was synthesized following the general procedure **X-BDP-CCCH2NMe2** using: **CH2-BDP-I** (394 mg, 0.68 mmol), CuI (6 mg, 0.03 mmol), $[Pd(PPh_3)_2Cl_2]$ (24 mg, 0.03 mmol) and 1-dimethylamino-2-propyne (127 mg, 1.53 mmol). The crude product was purified with column chromatography on silica gel using 10 % methanol in dichloromethane as eluent. The reaction yielded an orange solid (185 mg, 51 %). ^{1}H NMR (400 MHz, $CDCl_3$) δ = 7.69 – 7.61 (m, 2H), 7.49 – 7.43 (m, 2H), 7.26 – 7.20 (m, 2H), 7.20 – 7.11 (m, 4H), 7.11 – 7.05 (m, 2H), 5.79 (s, 2H), 4.44 (s, 2H), 3.54 (s, 2H), 2.44 (s, 6H), 1.47 (s, 12H) ppm. ^{13}C{^{1}H} NMR (101 MHz, $CDCl_3$) δ = 154.6, 141.9, 141.5, 139.6, 136.2, 132.5, 132.5, 130.8, 128.8, 126.5, 126.1, 125.9, 123.8, 121.8, 86.0, 85.0, 48.8, 44.5, 37.5, 29.8, 15.2, 15.1 ppm. Anal. Calcd for $C_{37}H_{36}BN_3$: C, 83.30; H, 6.80; N, 7.88. Found: C, 83.06; H, 7.13; N, 7.82. HR-MS (ESI) *m*/*z* calculated for $C_{37}H_{37}BN_3$ $[M+H]^+$: 534.3075. Found: 534.3072.

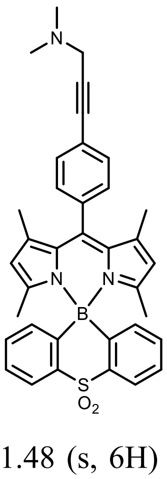

10'-(4-(3-(dimethylamino)prop-1-yn-1-yl)phenyl)-1',3',7',9'-tetramethyl-4'λ^4,10λ^4-spiro[dibenzo[*b*,*e*][1,4]thiaborinine-10,5'-dipyrrolo[1,2-*c*:2',1'-*f*][1,3,2]diazaborinine] 5,5-dioxide (SO2-BDP-CCCH2NMe2). Compound was synthesized following the general procedure **X-BDP-CCCH2NMe2** using: **SO2-BDP-I** (197 mg, 0.31 mmol), CuI (4 mg, 0.02 mmol), $[Pd(PPh_3)_2Cl_2]$ (13 mg, 0.02 mmol) and 1-dimethylamino-2-propyne (59 mg, 0.70 mmol). The crude product was purified with column chromatography on silica gel using 10 % methanol in dichloromethane as eluent. The reaction yielded an orange solid (83 mg, 45 %). ^{1}H NMR (400 MHz, $CDCl_3$) δ = 8.07 (dd, *J* = 8.1, 1.1 Hz, 2H), 7.70 – 7.64 (m, 2H), 7.50 – 7.41 (m, 4H), 7.39 (td, *J* = 7.4, 1.3 Hz, 2H), 7.20 (dd, *J* = 7.5, 1.4 Hz, 2H), 5.85 (s, 2H), 3.57 (s, 2H), 2.45 (s, 6H), 1.66 (s, 6H), 1.48 (s, 6H) ppm. ^{13}C{^{1}H} NMR (101 MHz, $CDCl_3$) δ = 155.3, 144.9, 141.6, 141.3, 135.3, 132.8, 132.7, 131.9, 130.7, 128.5, 128.4, 124.2, 122.5, 122.2, 86.1, 84.9, 48.7, 44.4, 31.0, 15.8, 15.1 ppm. Anal. Calcd for $C_{36}H_{34}BN_3O_2S$: C, 74.10; H, 5.87; N, 7.20. Found: C, 73.95; H, 6.02; N, 7.11. HR-MS (ESI) *m*/*z* calculated for $C_{36}H_{35}BN_3O_2S$ $[M+H]^+$: 584.2538. Found: 584.2535.

General procedure for the synthesis of X-BDP-ZWIT

The procedure was performed under an inert gas atmosphere of argon in 50 mL Schlenk tube. To a solution of **X-BDP-CCCH2NMe2** (1 equiv.; reaction scale 0.2 mmol) in 10 mL of dry toluene, 1,3-propanesultone (2.4 equiv.) was added. The reaction was stirred for 72 h at 60 ºC. Then, the mixture was cooled to room temperature. The orange solid precipitated from the solution. It was centrifuged and washed with toluene (3 x 5 mL) and pentane (3 x 5 mL). The solvents residues were vacuum-evaporated. The orange solid of **X-BDP-ZWIT** was obtained.

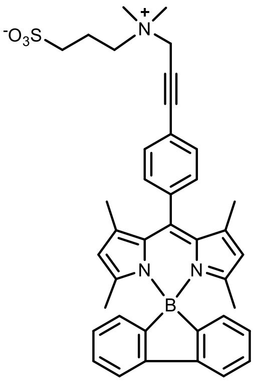

3-(dimethyl(3-(4-(1',3',7',9'-tetramethyl-4'λ^4,5λ^4-spiro[dibenzo[*b*,*d*]borole-5,5'-dipyrrolo[1,2-*c*:2',1'-*f*][1,3,2]diazaborinin]-10'-yl)phenyl)prop-2-yn-1-yl)ammonio)propane-1-sulfonate (Bf-BDP-ZWIT). Compound was synthesized following the general procedure **X-BDP-ZWIT** using: **Bf-BDP-CCCH2NMe2** (106 mg, 0.20 mmol) and 1,3-propanesultone (58 mg, 0.48 mmol). The reaction yielded an orange solid (104 mg, 79 %). ^{1}H NMR (400 MHz, DMSO-d_6) δ = 7.86 (d, J = 8.1 Hz, 2H), 7.67 (d, J = 7.5 Hz, 2H), 7.60 (d, J = 8.0 Hz, 2H), 7.22 (td, J = 7.3, 1.5 Hz, 2H), 7.15 – 7.09 (m, 2H), 7.07 (t, J = 7.0 Hz, 2H), 5.98 (s, 2H), 4.64 (s, 2H), 3.67 – 3.57 (m, 2H), 3.17 (s, 6H), 2.54 (t, J = 7.0 Hz, 2H), 2.15 – 2.04 (m, 2H), 1.38 (s, 12H) ppm. Anal. Calcd for $C_{39}H_{40}BN_3O_3S$: C, 73.01; H, 6.28; N, 6.55. Found: C, 72.74; H, 6.03; N, 6.41. HR-MS (ESI) m/z calculated for $C_{39}H_{41}BN_3O_3S$ [M+H]$^+$: 642.2956. Found: 642.2960. ^{13}C NMR spectrum could not be obtained due to the compound's low solubility in the available deuterated solvents.

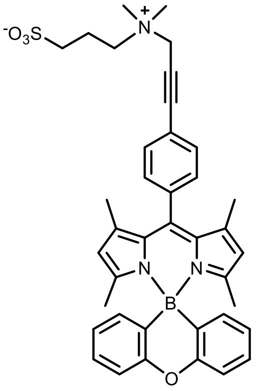

3-(dimethyl(3-(4-(1',3',7',9'-tetramethyl-4'λ^4,10λ^4-spiro[dibenzo[*b*,*e*][1,4]oxaborinine-10,5'-dipyrrolo[1,2-*c*:2',1'-*f*][1,3,2]diazaborinin]-10'-yl)phenyl)prop-2-yn-1-yl)ammonio)propane-1-sulfonate (O-BDP-ZWIT). Compound was synthesized following the general procedure **X-BDP-ZWIT** using: **O-BDP-CCCH2NMe2** (114 mg, 0.21 mmol) and 1,3-propanesultone (31 mg, 0.26 mmol). The reaction yielded an orange solid (103 mg, 74 %). The olubility of the compound was very low in common organic solvents, thus ^{1}H NMR spectrum is of moderate quality. ^{1}H NMR (400 MHz, DMSO-d_6) δ = 7.87 (m, 2H), 7.67 (d, J = 7.8 Hz, 2H), 7.28 (d, J = 8.3 Hz, 2H), 7.09 (d, J = 8.5 Hz, 4H), 6.98 (d, J = 7.4 Hz, 2H), 6.02 (s, 2H), 4.69 (s, 2H), 3.71 – 3.62 (m, 2H), 3.21 (s, 6H), 2.17 – 2.07 (m, 2H), 1.45 (s, 6H), 1.43 (s, 6H) ppm. Anal. Calcd for $C_{39}H_{40}BN_3O_4S$: C, 71.23; H, 6.13; N, 6.39. Found: 71.00; H, 6.29; N, 6.19. HR-MS (ESI) *m*/*z* calculated for $C_{39}H_{41}BN_3O_4S$ [M+H]$^+$: 658.2905. Found: 658.2898. ^{13}C NMR spectrum could not be obtained due to the compound's low solubility in the available deuterated solvents.

3-(dimethyl(3-(4-(1',3',5,7',9'-pentamethyl-5*H*-4'λ^4,10λ^4-spiro[dibenzo[*b*,*e*][1,4]azaborinine-10,5'-dipyrrolo[1,2-*c*:2',1'-*f*][1,3,2]diazaborinin]-10'-yl)phenyl)prop-2-yn-1-yl)ammonio)propane-1-sulfonate (NMe-BDP-ZWIT). Compound was synthesized following the general procedure **X-BDP-ZWIT** using: **NMe-BDP-CCCH2NMe2** (230 mg, 0.42 mmol) and 1,3-propanesultone (62 mg, 0.50 mmol). The reaction yielded an orange solid (158 mg, 56 %). ^{1}H NMR (400 MHz, DMSO-*d*$_6$) δ = 7.89 – 7.82 (m, 2H), 7.65 – 7.57 (m, 2H), 7.24 – 7.09 (m, 2H), 7.01 (dd, *J* = 8.4, 0.9 Hz, 2H), 6.94 (dd, *J* = 7.2, 1.8 Hz, 2H), 6.71 (td, *J* = 7.1, 0.9 Hz, 2H), 5.93 (s, 2H), 4.64 (s, 2H), 3.67 – 3.58 (m, 2H), 3.57 (s, 3H), 3.17 (s, 6H), 2.54 – 2.48 (m, 2H), 2.08 (s, 2H), 1.38 (s, 6H), 1.34 (s, 6H) ppm. Anal. Calcd for $C_{40}H_{43}BN_4O_3S$: C, 71.63; H, 6.46; N, 8.35. Found: C, 71.48; H, 6.60; N, 8.22. HR-MS (ESI) *m*/*z* calculated for $C_{40}H_{44}BN_4O_3S$ [M+H]$^+$: 671.3222. Found: 671.3215. ^{13}C NMR spectrum could not be obtained due to the compound's low solubility in the available deuterated solvents.

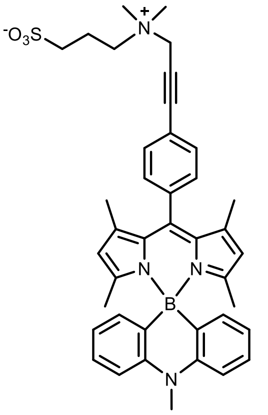

3-(dimethyl(3-(4-(1',3',7',9'-tetramethyl-10*H*-4'λ^4,5λ^4-spiro[dibenzo[*b*,*e*]borinine-5,5'-dipyrrolo[1,2-*c*:2',1'-*f*][1,3,2]diazaborinin]-10'-yl)phenyl)prop-2-yn-1-yl)ammonio)propane-1-sulfonate (**CH2-BDP-ZWIT**). Compound was synthesized following the general procedure **X-BDP-ZWIT** using: **CH2-BDP-CCCH2NMe2** (252 mg, 0.47 mmol) and 1,3-propanesultone (69 mg, 0.57 mmol). The reaction yielded an orange solid (172 mg, 56 %). ^{1}H NMR (400 MHz, (DMSO-*d*$_6$) δ = 7.70 – 7.63 (m, 2H), 7.57 – 7.50 (m, 2H), 7.24 – 7.17 (m, 2H), 7.15 – 7.08 (m, 2H), 7.06 – 6.96 (m, 4H), 5.92 (s, 2H), 4.36 (s, 2H), 3.50 (s, 2H), 2.53 – 2.47 (m, 4H), 2.27 (s, 3H), 2.03 – 1.93 (m, 2H), 1.39 (s, 3H), 1.39 (s, 3H), 1.36 (s, 6H) ppm. Anal. Calcd for $C_{40}H_{42}BN_3O_3S$: C, 73.28; H, 6.46; N, 6.41. Found: C, 73.19; H, 6.66; N, 6.36. HR-MS (ESI) *m*/*z* calculated for $C_{40}H_{43}BN_3O_3S$ [M+H]$^+$: 656.3113. Found: 656.3102. ^{13}C NMR spectrum could not be obtained due to the compound's low solubility in the available deuterated solvents.

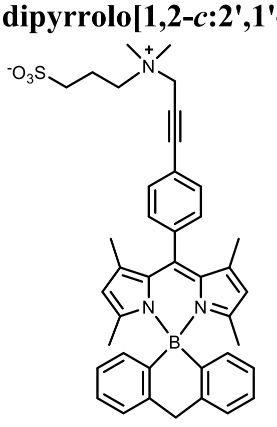

3-(dimethyl(3-(4-(1',3',7',9'-tetramethyl-5,5-dioxido-4'λ^4,10λ^4-spiro[dibenzo[*b*,*e*][1,4]thiaborinine-10,5'-dipyrrolo[1,2-*c*:2',1'-*f*][1,3,2]diazaborinin]-10'-yl)phenyl)prop-2-yn-1-yl)ammonio)propane-1-sulfonate (SO2-BDP-ZWIT). Compound was synthesized following general procedure **X-BDP-ZWIT** using: **SO2-BDP-CCCH2NMe2** (75 mg, 0.13 mmol) and 1,3-propanesultone (19 mg, 0.57 mmol). The reaction yielded an orange solid (173 mg, 30 %). ^{1}H NMR (400 MHz, DMSO-*d*$_6$) δ = 7.94 – 7.86 (m, 4H), 7.73 – 7.65 (m, 2H), 7.54 (ddd, *J* = 7.9, 7.3, 1.5 Hz, 2H), 7.48 (td, *J* = 7.3, 1.4 Hz, 2H), 7.19 (ddd, *J* = 7.4, 1.5, 0.5 Hz, 2H), 6.02 (d, *J* = 0.9 Hz, 2H), 4.65 (s, 2H), 3.69 – 3.59 (m, 2H), 3.17 (s, 6H), 2.54 (t, *J* = 6.9 Hz, 2H), 2.15 – 2.02 (m, 2H), 1.53 (s, 6H), 1.41 (d, *J* = 0.9 Hz, 6H) ppm. Anal. Calcd for $C_{39}H_{40}BN_3O_5S_2$: C, 66.38; H, 5.71; N, 5.95. Found: C, 66.10; H, 5.95; N, 5.76. HR-MS (ESI) *m*/*z* calculated for $C_{39}H_{41}BN_3O_5S_2$ [M+H]$^+$: 706.2575. Found: 706.2566. ^{13}C NMR spectrum could not be obtained due to the compound's low solubility in the available deuterated solvents.

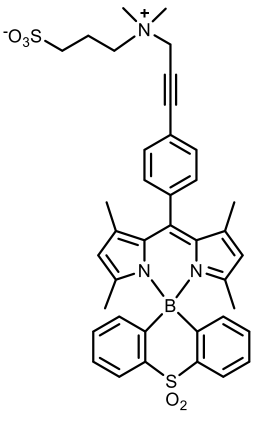

1.4 Synthesis of unsubstituted X-BDP

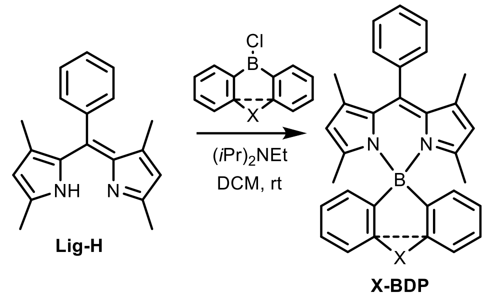

General procedure for the synthesis of X-BDP

Lig-H was dissolved in dry DCM (10 mL) and *N*,*N*-diisopropylethylamine (1 equiv. with respect to the amount of proligand) was added. The resulting solution was added to the solution of dibenzo(X)borinine chloride or 9-chloroborafluorene (1 equiv. with respect to the amount of proligand) in 10 mL dry DCM. The reaction was stirred overnight at room temperature. Then, the distilled water (20 mL) was added. The phases were separated, water phase was extracted three times with dichloromethane (3 × 20 mL). Combined organic phases were dried with anhydrous $MgSO_4$ and concentrated on a rotary evaporator. The crude product was purified by column chromatography on silica gel, yielding an orange solid.

1',3',7',9'-tetramethyl-10'-phenyl-4'λ^4,5λ^4-spiro[dibenzo[*b*,*d*]borole-5,5'-dipyrrolo[1,2-*c*:2',1'-*f*][1,3,2]diazaborinine] (Bf-BDP). Compound was synthesized following the general procedure **X-BDP** using: 5-chloro-5*H*-dibenzo[*b*,*d*]borole (265 mg, 1.34 mmol), *N*,*N*-diisopropylamine (173 mg, 1.34 mmol) and (*N*,*N*)-chelating proligand (388 mg, 1.40 mmol) freshly prepared from benzaldehyde (0.26 g, 2.41 mmol), 2,4-dimethylpyrrole (0.46 g, 4.82 mmol) and 2,3-dichloro-5,6-dicyano-1,4-benzoquinone - DDQ (0.56 g, 2.41 mmol). The crude product was purified by column chromatography on silica gel using hexane:touene (ratio 2:3) as the eluent. The reaction yielded an orange solid (404 mg, 69 %). ^{1}H NMR (400 MHz, $CDCl_3$) δ = 7.66 – 7.59 (m, 2H), 7.59 – 7.48 (m, 3H), 7.48 – 7.41 (m, 2H), 7.29 – 7.21 (m, 4H), 7.11 (ddd, *J* = 7.4, 6.9, 1.1 Hz, 2H), 5.82 (s, 2H), 1.49 (s, 6H), 1.42 (s, 6H) ppm. Anal. Calcd for $C_{31}H_{27}BN_2$: C, 84.94; H, 6.21; N, 6.39. Found: C, 84.91; H, 6.26; N, 6.33. ^{1}H NMR spectrum is consistent with the literature data.[5,6]

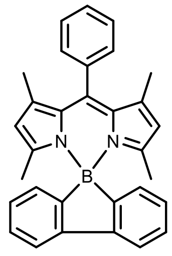

1',3',7',9'-tetramethyl-10'-phenyl-4'λ^4,10λ^4-spiro[dibenzo[*b*,*e*][1,4]oxaborinine-10,5'-dipyrrolo[1,2-*c*:2',1'-*f*][1,3,2]diazaborinine] (O-BDP). Compound was synthesized following the general procedure **X-BDP** using: 10-chloro-10H-dibenzo[b,e][1,4]oxaborinine (**O-Cl**) (166 mg, 0.774 mmol), (*N*,*N*)-chelating proligand (214 mg, 0.774 mmol) and *N*,*N*-diisopropylamine (100 mg, 0.774 mmol). The crude product was purified by column chromatography on silica gel (hexane:ethyl acetate 1:19). The reaction yielded an orange solid (63 mg, 18 %). ^{1}H NMR (400 MHz, $CDCl_3$) δ = 7.59 – 7.51 (m, 3H), 7.48 – 7.44 (m, 2H), 7.23 (ddd, *J* = 8.2, 7.0, 1.8 Hz, 2H), 7.15 (dd, *J* = 7.3, 1.7 Hz, 2H), 7.09 (ddd, *J* = 8.2, 1.1, 0.4 Hz, 2H), 6.95 (td, *J* = 7.2, 1.1 Hz, 2H), 5.82 (s, 1H), 5.81 (s, 1H), 1.54 (s, 6H), 1.53 (s, 6H) ppm. ^{13}C{^{1}H} NMR (101 MHz, $CDCl_3$) δ = 157.8, 154.9, 142.4, 140.2, 136.1, 133.0, 131.1, 129.2, 128.9, 128.6, 128.0, 122.5, 121.8, 115.0, 15.4, 14.8 ppm. Anal.

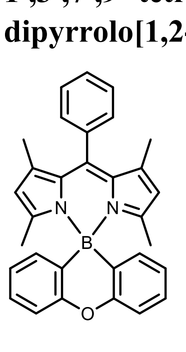

Calcd for $C_{31}H_{27}BN_2O$: C, 81.94; H, 5.99; N, 6.17. Found: C, 82.04; H, 6.12; N, 6.08. HR-MS (ESI) *m*/*z* calculated for $C_{31}H_{28}BN_2O$ $[M+H]^+$: 455.2283. Found: 455.2289.

1',3',5,7',9'-pentamethyl-10'-phenyl-5*H*-4'λ^4,10λ^4-spiro[dibenzo[*b*,*e*][1,4]azaborinine-10,5'-dipyrrolo[1,2-*c*:2',1'-*f*][1,3,2]diazaborinine] (NMe-BDP). Compound was synthesized following the general procedure **X-BDP** using: 10-chloro-5-methyl-5,10-dihydrodibenzo[*b*,*e*][1,4]azaborinine (**NMe-Cl**) (304 mg, 1.34 mmol), (*N*,*N*)-chelating proligand (444 mg, 1.61 mmol) and *N*,*N*-diisopropylamine (173 mg, 1.34 mmol). The crude product was purified by column chromatography on silica gel (hexane:chloroform 1:1). The reaction yielded an orange solid (168 mg, 27 %). ^{1}H NMR (400 MHz, $CDCl_3$) δ = 7.59 – 7.46 (m, 5H), 7.28 – 7.20 (m, 2H), 7.13 (dd, *J* = 7.1, 1.8 Hz, 2H), 6.99 (d, *J* = 8.3 Hz, 2H), 6.81 (t, *J* = 7.1 Hz, 2H), 5.80 (s, 2H), 3.67 (s, 3H), 1.46 (s, 6H), 1.43 (s, 6H) ppm. $^{13}C\{^1H\}$ NMR (101 MHz, $CDCl_3$) δ = 155.0, 147.7, 142.2, 139.7, 136.4, 133.2, 130.9, 129.1, 128.8, 128.6, 127.2, 121.6, 119.4, 111.7, 35.9, 15.2, 14.8 ppm. Anal. Calcd for $C_{32}H_{30}BN_3$: C, 82.23; H, 6.47; N, 8.99. Found: C, 82.40; H, 6.55; N, 8.70. HR-MS (ESI) *m*/*z* calculated for $C_{32}H_{31}BN_3$ $[M+H]^+$: 468.2599. Found: 468.2606.

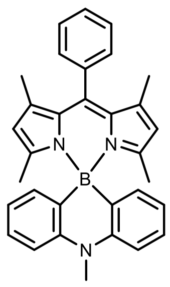

1',3',7',9'-tetramethyl-10'-phenyl-10*H*-4'λ^4,5λ^4-spiro[dibenzo[*b*,*e*]borinine-5,5'-dipyrrolo[1,2-*c*:2',1'-*f*][1,3,2]diazaborinine] (CH2-BDP). Compound was synthesized following the general procedure **X-BDP** using: 5-chloro-5,10-dihydrodibenzo[*b*,*e*]borinine (**CH2-Cl**) (292 mg, 1.37 mmol), (*N*,*N*)-chelating proligand (493 mg, 1.78 mmol) and *N*,*N*-diisopropylamine (212 mg, 1.64 mmol). The crude product was purified by column chromatography on silica gel (hexane:chloroform 1:1). The reaction yielded an orange solid (340 mg, 55 %). ^{1}H NMR (400 MHz, $CDCl_3$) δ = 7.62 – 7.53 (m, 2H), 7.56 – 7.47 (m, 3H), 7.28 – 7.22 (m, 2H), 7.21 – 7.15 (m, 4H), 7.13 – 7.07 (m, 2H), 5.80 (s, 2H), 4.45 (s, 2H), 1.49 (s, 6H), 1.45 (s, 6H) ppm. $^{13}C\{^1H\}$ NMR (100 MHz, $CDCl_3$) δ = 154.3, 142.4, 141.9, 139.8, 136.3, 132.5, 131.1, 129.1, 128.8, 128.6, 126.5, 126.0, 125.9, 121.6, 37.5, 15.2, 14.8 ppm. Anal. Calcd for $C_{32}H_{29}BN_2$: C, 85.96; H, 6.46; N, 6.19. Found: C, 86.03; H, 6.51; N, 6.12. HR-MS (ESI) *m*/*z* calculated for $C_{32}H_{30}BN_2$ $[M+H]^+$: 453.2490. Found: 453.2497.

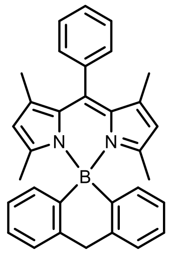

1',3',7',9'-tetramethyl-10'-phenyl-4'λ^4,10λ^4-spiro[dibenzo[*b*,*e*][1,4]thiaborinine-10,5'-dipyrrolo[1,2-*c*:2',1'-*f*][1,3,2]diazaborinine] 5,5-dioxide (SO2-BDP). The compound was synthesized following the general procedure **X-BDP** using: 10-chloro-10*H*-dibenzo[*b*,*e*][1,4]thiaborinine 5,5-dioxide (**SO2-Cl**) (17 mg, 0.065 mmol), ((*N*,*N*)-chelating proligand (18 mg, 0.065 mmol) and *N*,*N*-diisopropylamine (8.4 mg, 0.065 mmol). The crude product was purified by column chromatography on silica gel (hexane:ethyl acetate 4:1). The reaction yielded an orange solid (17 mg, 52 %). ^{1}H NMR (400 MHz, $CDCl_3$) δ = 8.09 (ddd, *J* = 7.9, 1.3, 0.6 Hz, 2H), 7.62 – 7.52 (m, 3H), 7.50 – 7.45 (m, 4H), 7.40 (td, *J* = 7.4, 1.3 Hz, 2H), 7.23 (dd, *J* = 7.5, 1.0 Hz, 2H), 5.85 (d, *J* = 1.0 Hz, 2H), 1.67 (s, 6H), 1.45 (d, *J* = 0.9 Hz, 6H) ppm. $^{13}C\{^1H\}$ NMR (101 MHz, $CDCl_3$) δ = 155.1, 144.9, 142.6, 141.5, 135.6, 132.9, 131.9, 131.0, 129.4, 129.2, 128.41, 128.40, 122.4, 122.3, 15.8, 14.9 ppm. Anal. Calcd for $C_{31}H_{27}BN_2O_2S$: C, 74.11; H, 5.42; N, 5.58. Found: C, 73.93; H, 5.57; N, 5.46. HR-MS (ESI) *m*/*z* calculated for $C_{31}H_{28}BN_2O_2S$ $[M+H]^+$: 503.1953. Found: 503.1959.

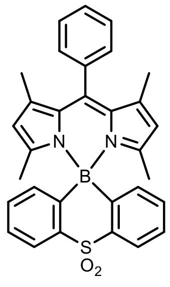

1.5 Synthesis of BF2-BDP reference BODIPY complexes

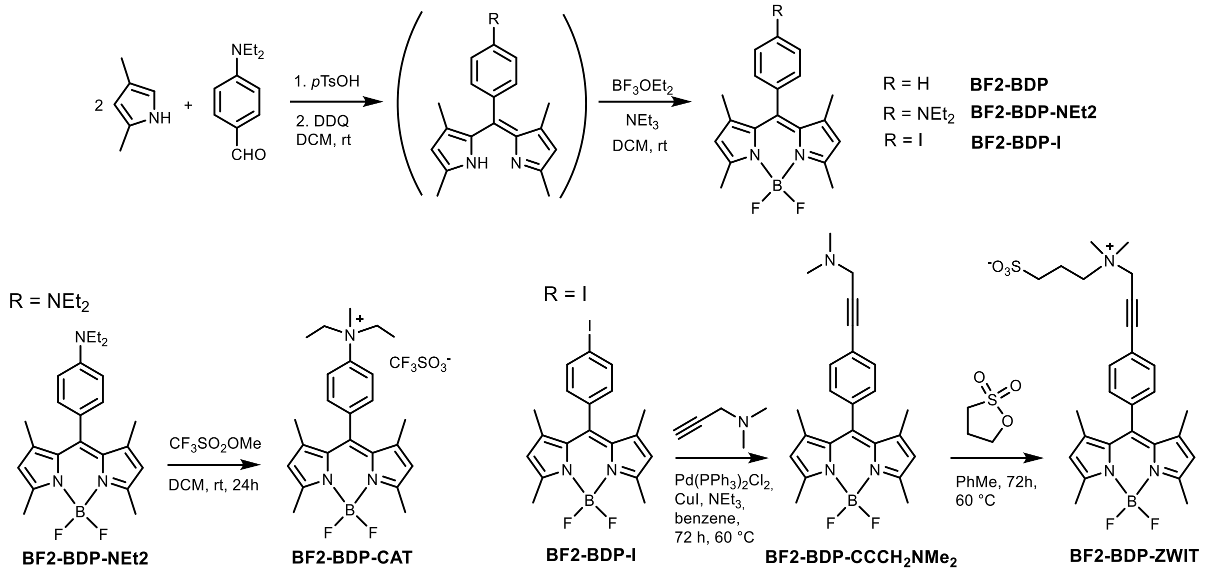

5,5-difluoro-1,3,7,9-tetramethyl-10-phenyl-5*H*-4λ⁴,5λ⁴-dipyrrolo[1,2-*c*:2',1'-*f*][1,3,2]diazaborinine (BF2-BDP). The procedure was performed under an inert gas atmosphere of argon in a 50 mL Schlenk tube. To a solution of benzaldehyde (1.50 g, 14.1 mmol) in dry dichloromethane (20 mL) was added 2,4-dimethylpyrrole (2.69 g, 28.3 mmol). Next, the catalytic amount of *p*-toluenesulfonic acid was added (about 50 mg). The reaction was stirred overnight at room temperature. Then, a suspension of a 2,3-dichloro-5,6-dicyano-1,4-benzoquinone - DDQ (3.26 g, 14.1 mmol) in a dry dichloromethane (10 mL) was added to the reaction. The reaction yielded a dark brow solid of (*N*,*N*)-chelating proligand and was used in the subsequent step without additional purification. It was dissolved in dry DCM (20 mL) and triethylamine (4.02, 40 mmol) was added. After stirring for 1 h, BF_3OEt_2 (6.77 g, 47 mmol) was added. The reaction was stirred overnight at room temperature. Then, the reaction was washed with distilled water and extracted three times with dichloromethane (3 × 20 mL). Combined organic phases were dried with anhydrous $MgSO_4$ and concentrated on a rotary evaporator after filtration. The crude product was purified using column chromatography (silica gel, hexane:toluene 3:2). The reaction yielded an orange solid (1.28 g, 28 %). ^{1}H NMR (400 MHz, $CDCl_3$) δ = 7.53 – 7.43 (m, 3H), 7.31 – 7.25 (m, 2H), 5.98 (s, 2H), 2.56 (s, 6H), 1.37 (s, 6H) ppm. ^{19}F NMR (376 MHz, $CDCl_3$) δ = 146.33 (q, *J* = 33.0 Hz) ppm. ^{1}H NMR spectrum is consistent with the literature data.[7]

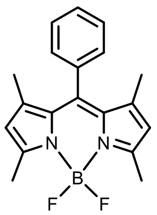

4-(5,5-difluoro-1,3,7,9-tetramethyl-5*H*-4λ⁴,5λ⁴-dipyrrolo[1,2-*c*:2',1'-*f*][1,3,2]diazaborinin-10-yl)-*N*,*N*-diethylaniline (BF2-BDP-NEt2). Compound was synthesized following the procedure described for **BF2-BDP** using *N*,*N*-diisopropylethylamine (124 mg, 0.96 mmol), BF_3OEt_2 (136 mg, 0.96 mmol) and (*N*,*N*)-chelating proligand (333 mg, 0.96 mmol), freshly prepared from *p*-(diethyloamino)benzaldehyde (0.71 g, 4.0 mmol), 2,4-dimethylpyrrole (0.76 g, 8.0 mmol), and DDQ (0.91 g, 4.0 mmol). The crude product was purified using column chromatography (hexane:toluene 2:3). The reaction yielded an orange solid (96 mg, 26 %). ^{1}H NMR (400 MHz, $CDCl_3$) δ = 7.06 – 6.96 (m, 2H), 6.78 – 6.69 (m, 2H), 5.97 (s, 2H), 3.40 (q, *J* = 7.1 Hz, 4H), 2.55 (s, 6H), 1.52 (s, 6H), 1.20 (t, *J* = 7.0 Hz, 6H) ppm. ^{19}F NMR

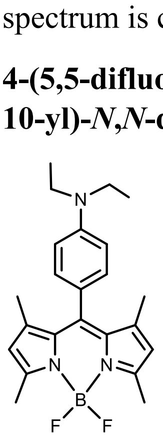

(376 MHz, $CDCl_3$) δ = 146.32 (q, *J* = 33.0 Hz). ^1H NMR spectrum is consistent with the literature data.[8]

4-(5,5-difluoro-1,3,7,9-tetramethyl-5*H*-4λ^4,5λ^4-dipyrrolo[1,2-*c*:2',1'-*f*][1,3,2]diazaborinin-10-yl)-*N*,*N*-diethyl-*N*-methylbenzenaminium triflate (BF2-BDP-CAT).

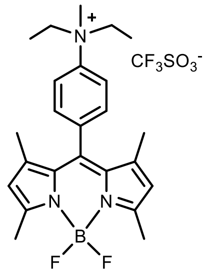

Compound was synthesized following the general procedure **X-BDP-CAT** using **BF2-BDP-NEt2** (92 mg, 0.23 mmol) and methyl triflate (191 mg, 1.16 mmol). The reaction yielded an orange solid (74 mg, 57 %). ^1H NMR (400 MHz, acetone-d_6) δ = 8.24 – 8.15 (m, 2H), 7.89 – 7.80 (m, 2H), 6.16 (s, 2H), 4.41 (dq, *J* = 14.3, 7.2 Hz, 2H), 4.20 (dq, *J* = 13.1, 7.1 Hz, 2H), 3.86 (s, 3H), 2.51 (s, 6H), 1.44 (s, 6H), 1.28 (t, *J* = 7.1 Hz, 6H) ppm. ^{19}F NMR (376 MHz, CDCl3) δ = –78.32, 146.29 (q, *J* = 31.9 Hz) ppm. Anal. Calcd for $C_{25}H_{31}BF_5N_3O_3S$: C, 53.68; H, 5.59; N, 7.51. Found: C, 53.47; H, 5.35; N, 7.70. ^1H NMR spectrum is consistent with the literature data.[9]

5,5-difluoro-10-(4-iodophenyl)-1,3,7,9-tetramethyl-5*H*-4λ^4,5λ^4-dipyrrolo[1,2-*c*:2',1'-*f*][1,3,2]diazaborinine (BF2-BDP-I).

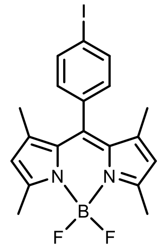

Compound was synthesized following the procedure described for **BF2-BDP** using *N*,*N*-diisopropylethylamine (124 mg, 0.96 mmol), BF_3OEt_2 (136 mg, 0.96 mmol) and (*N*,*N*)-chelating proligand (333 mg, 0.96 mmol), freshly prepared from *p*-iodobenzaldehyde (0.98 g, 4.0 mmol), 2,4-dimethylpyrrole (0.76 g, 8.0 mmol), 2,3-dichloro-5,6-dicyano-1,4-benzoquinone – DDQ (0.91 g, 4.0 mmol). The crude product was purified using column chromatography with dichloromethane as eluent. ^1H NMR (400 MHz, $CDCl_3$) δ = 7.88 – 7.78 (m, 2H), 7.07 – 6.97 (m, 2H), 5.99 (s, 2H), 2.55 (s, 6H), 1.42 (s, 6H) ppm. The reaction yielded an orange solid (340 mg, 29 %). ^{19}F NMR (376 MHz, $CDCl_3$) δ = 146.32 (q, *J* = 32.8 Hz) ppm. ^1H NMR spectrum is consistent with the literature data.[10]

3-(4-(5,5-difluoro-1,3,7,9-tetramethyl-5*H*-4λ^4,5λ^4-dipyrrolo[1,2-*c*:2',1'-*f*][1,3,2]diazaborinin-10-yl)phenyl)-*N*,*N*-dimethylprop-2-yn-1-amine (BF2-BDP-CCCH2NMe2).

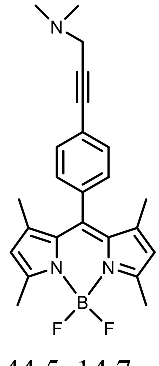

Compound was synthesized following general procedure **X-BDP-CCCH2NMe2** using: **BF2-BDP-I** (301 mg, 0.67 mmol), CuI (8 mg, 0.04 mmol), [Pd(PPh_3)$_2$$Cl_2$] (28 mg, 0.04 mmol) and 1-dimethylamino-2-propyne (127 mg, 1.51 mmol). The crude product was purified with column chromatography on silica gel using 10 % methanol in dichloromethane as eluent. The reaction yielded an orange solid (178 mg, 66 %). ^1H NMR (400 MHz, $CDCl_3$) δ = 7.60 – 7.51 (m, 2H), 7.25 – 7.20 (m, 2H), 5.98 (s, 2H), 3.50 (s, 2H), 2.55 (s, 6H), 2.40 (s, 6H), 1.40 (s, 6H) ppm. $^{13}C\{^1H\}$ NMR (101 MHz, $CDCl_3$) δ = 155.8, 143.1, 141.0, 134.9, 132.5, 128.2, 124.1, 121.5, 121.4, 86.3, 84.7, 48.7, 44.5, 14.7 ppm. ^{19}F NMR (376 MHz, $CDCl_3$) δ = 146.30 (q, *J* = 32.8 Hz) ppm. Anal. Calcd for $C_{24}H_{26}BF_2N_3$: C, 71.12; H, 6.47; N, 10.37. Found: C, 70.93; H, 6.57; N, 10.27.

3-((3-(4-(5,5-difluoro-1,3,7,9-tetramethyl-5*H*-4λ[4],5λ[4]-dipyrrolo[1,2-*c*:2',1'-*f*][1,3,2]diazaborinin-10-yl)phenyl)prop-2-yn-1-yl)dimethylammonio)propane-1-sulfonate (BF2-BDP-ZWIT).

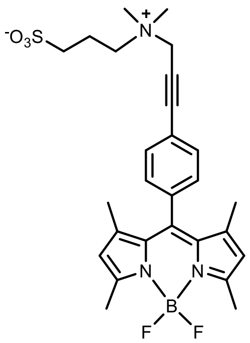

Compound was synthesized following general procedure **X-BDP-ZWIT** using: **BF2-BDP-CCCH2NMe2** (178 mg, 0.43 mmol) and 1,3-propanesultone (64 mg, 0.52 mmol). The reaction yielded an orange solid (216 mg, 93 %). ^{1}H NMR (400 MHz, DMSO-d_6) δ = 7.85 – 7.75 (m, 2H), 7.50 – 7.41 (m, 2H), 6.18 (s, 2H), 4.63 (s, 2H), 3.65 – 3.57 (m, 2H), 3.15 (s, 6H), 2.54 – 2.50 (m, 2H), 2.43 (s, 6H), 2.13 – 2.02 (m, 2H), 1.35 (s, 6H) ppm. $^{13}C\{^{1}H\}$ NMR (100 MHz, DMSO-d_6) δ = 155.68, 143.05, 141.21, 135.72, 133.23, 130.83, 128.92, 122.01, 121.90, 89.93, 79.55, 76.05, 70.28, 63.22, 50.36, 48.16, 19.58, 14.66 ppm. ^{19}F NMR (376 MHz, DMSO-d_6) δ = 143.64 (q, J = 31.5 Hz) ppm. Anal. Calcd for $C_{27}H_{32}BF_2N_3O_3S$: C, 61.49; H, 6.12; N, 7.97. Found: C, 61.17; H, 6.39; N, 7.64.

2. X-ray crystallography

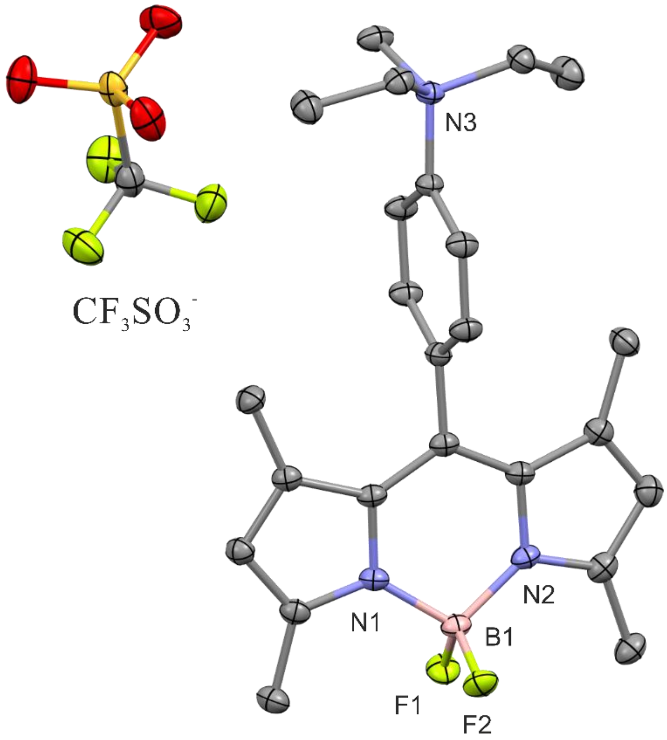

Figure S1. Molecular structure of **BF2-BDP-CAT**. Thermal motions are shown as atomic displacement parameters (ADPs) at the 50% probability level. The hydrogen atoms were omitted for clarity.

Table S1. Selected crystal data, data collection and refinement parameters for **NMe-BDP-CAT** and **BF2-BDP-CAT**.

	**NMe-BDP-CAT**	**BF2-BDP-CAT**
Empirical formula	$C_{81}H_{89}B_2Cl_{15}F_6N_8O_6S_2$	$C_{25}H_{31}BF_5N_3O_3S$
Formula weight	2002.09	559.40
T / K	100.01(10)	100.01(10)
Crystal system	triclinic	monoclinic
Space group	*P*-1	$P2_1/n$
a / Å	14.4444(3)	8.65020(10)
b / Å	17.2400(5)	30.0013(4)
c / Å	19.0349(3)	10.07740(10)
α / °	91.169(2)	90
β / °	90.536(2)	97.398(2)
γ / °	91.305(2)	90
V / Å^3	4737.65(18)	2593.49(5)
Z	2	4
ρ_{calc} / gcm^{-3}	1.403	1.433
μ / mm^{-1}	4.946	1.727
F(000)	2060.0	1168.0
Crystal size/mm^3	0.1254 × 0.0854 × 0.052	0.142 × 0.134 × 0.124
Radiation	CuKα (λ = 1.54184)	CuKα (λ = 1.54184)
2Θ range for data collection/°	4.644 - 153.804	9.328 - 155.486
Index ranges	$-18 \leq h \leq 17$, $-21 \leq k \leq 21$, $-23 \leq 1 \leq 19$	$-10 \leq h \leq 10$, $-37 \leq k \leq 32$, $-12 \leq l \leq 12$
Reflections collected	75571	20955
Independent reflections	19720 R_{int} = 0.1130	5511 R_{int} = 0.0381
Data/restraints/parameters	19720/76/1245	5511/0/350
Goodness-of-fit on F^2	1.315	1.089
Final R indexes [I>=2σ (I)]	R_1 = 0.1168, wR_2 = 0.3207	R_1 = 0.0364, wR_2 = 0.0968
Final R indexes [all data]	R_1 = 0.1463, wR_2 = 0.3603	R_1 = 0.0414, wR_2 = 0.1000
Largest diff. peak/hole / e Å^{-3}	1.97/-0.94	0.35/-0.35

3. Standy-state and time-resolved spectroscopy

3.1 Absorption and emission spectra

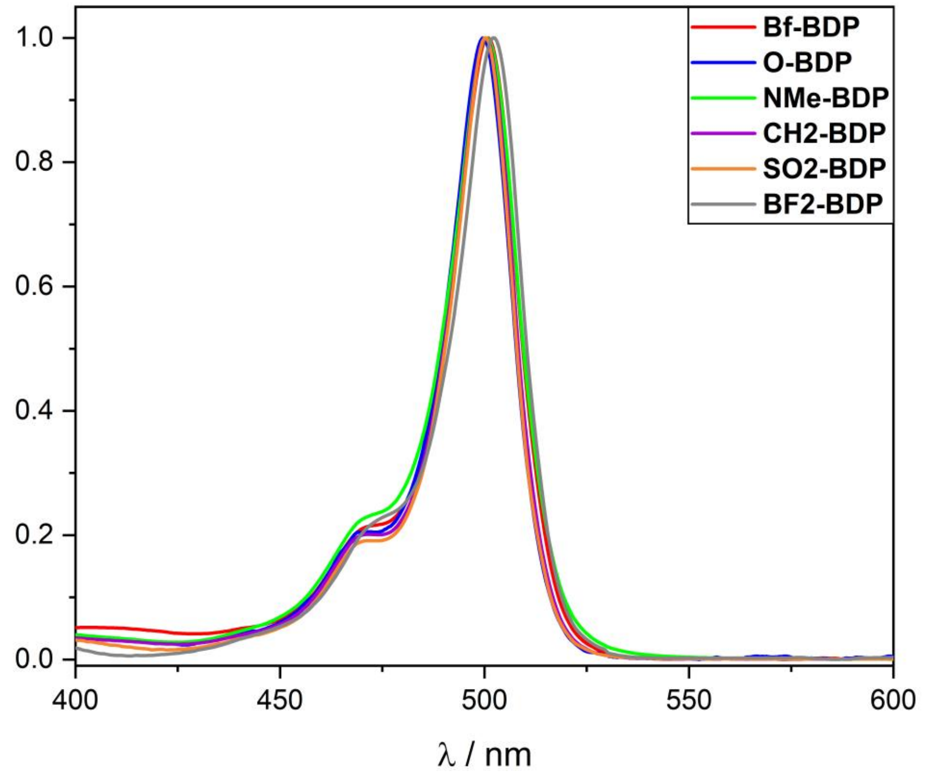

Figure S2. Normalized absorption spectra of **X-BDP** in diluted (c = 10^{-5} M) $CHCl_3$ solutions.

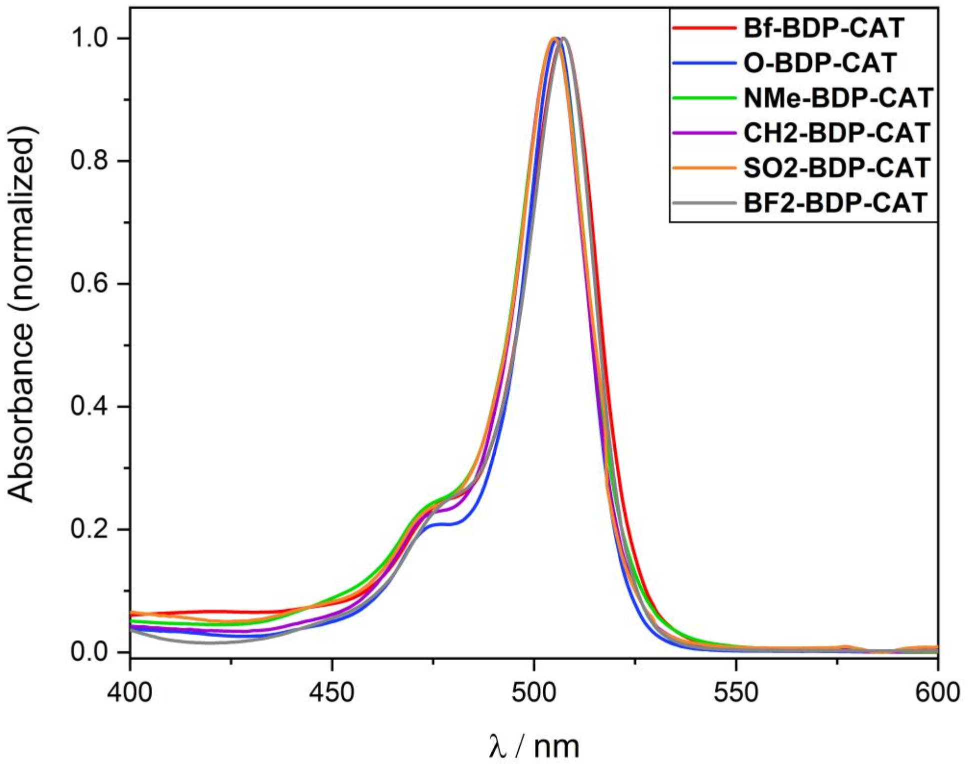

Figure S3. Normalized absorption spectra of **X-BDP-CAT** in diluted (c = 10^{-5} M) $CHCl_3$ solutions.

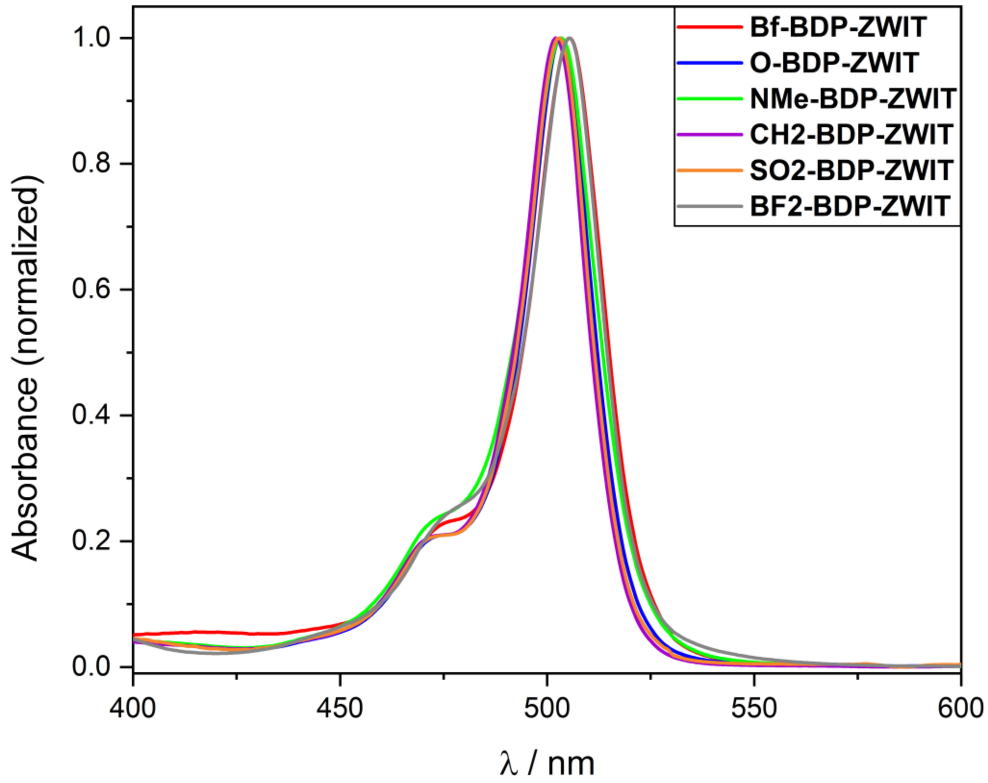

Figure S4. Normalized absorption spectra of **X-BDP-ZWIT** in diluted (c = 10^{-5} M) $CHCl_3$ solutions.

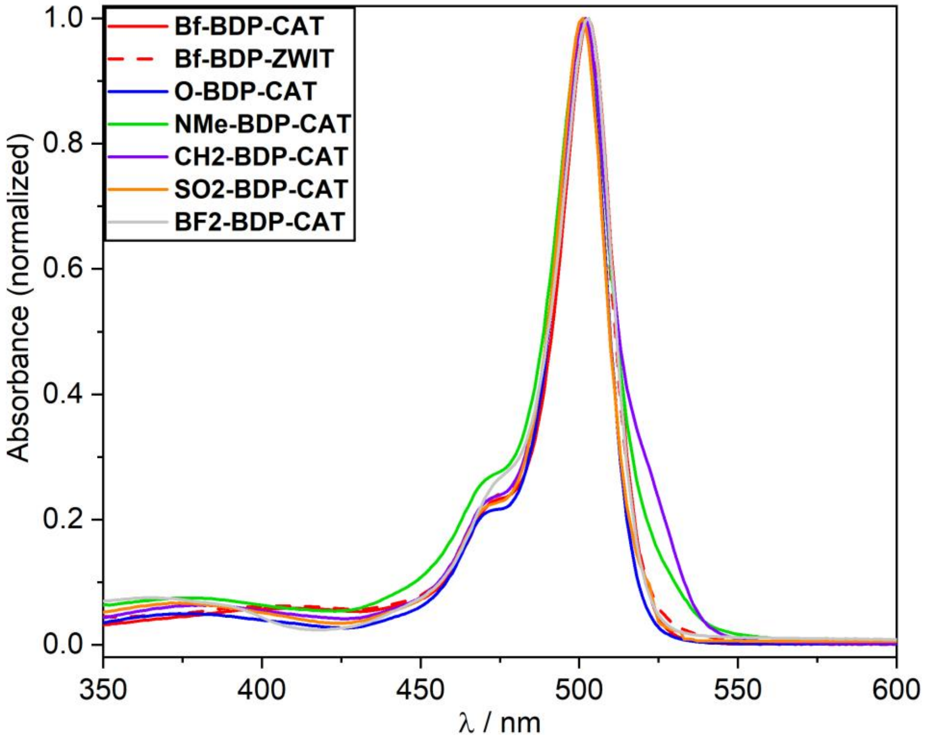

Figure S5. Normalized absorption spectra of **X-BDP-CAT** and **Bf-BDP-ZWIT** in diluted (c = 10^{-5} M) water/DMSO (1/1 v/v) solutions.

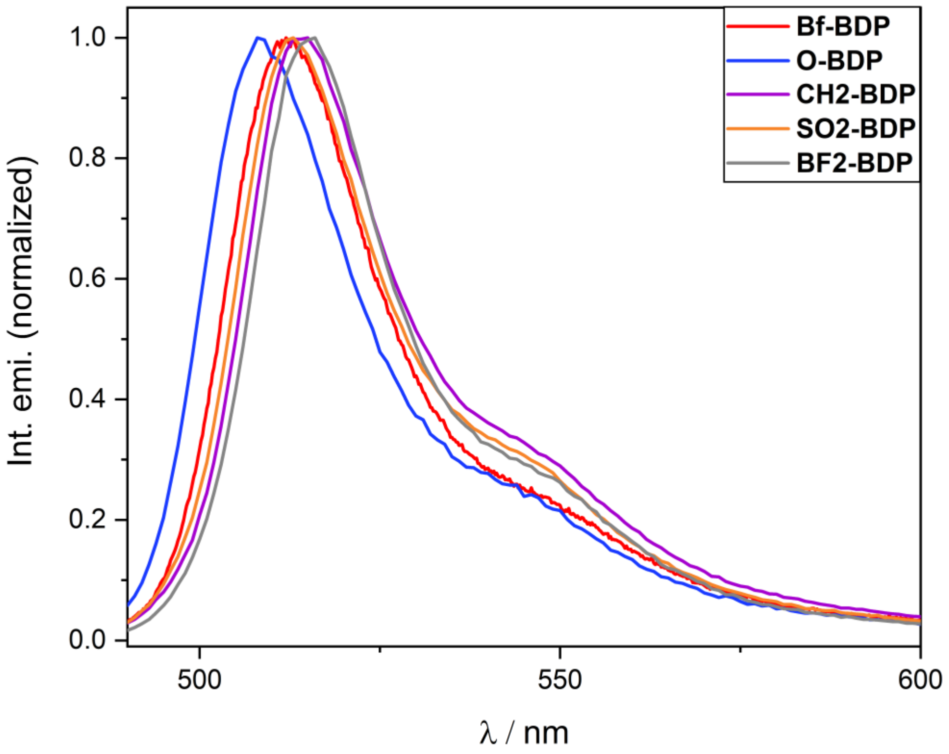

Figure S6. Normalized emission spectra of **X-BDP** in diluted (c = 10^{-5} M) $CHCl_3$ solutions (λ_{ex} = 470 nm).

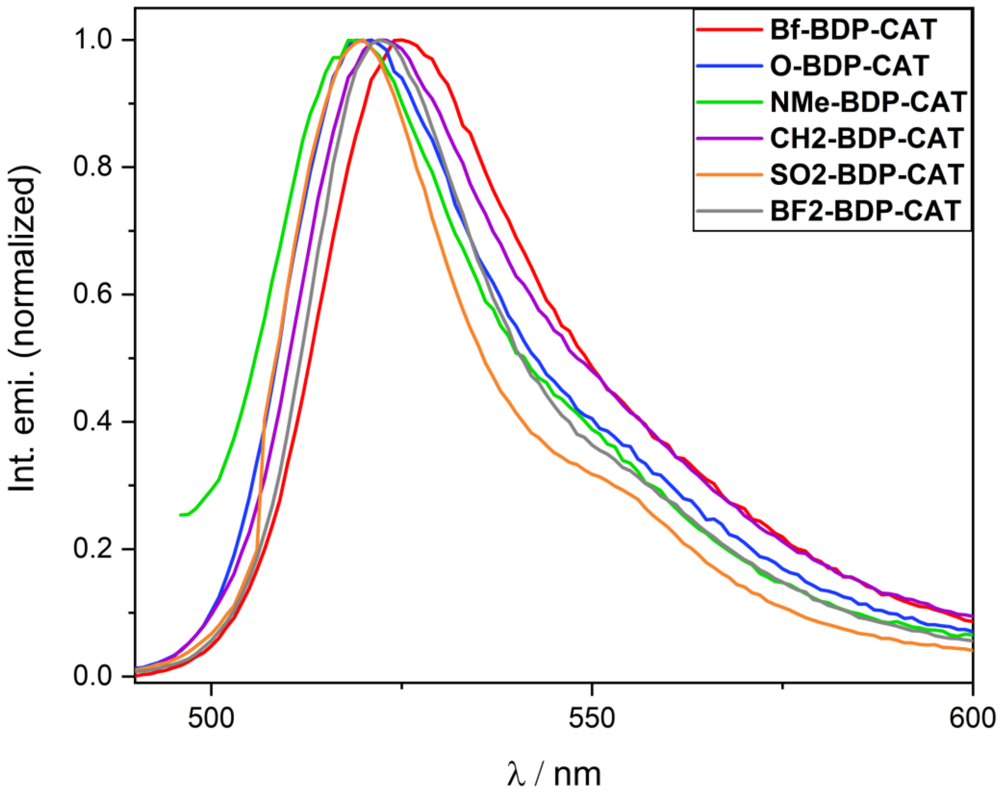

Figure S7. Normalized emission spectra of **X-BDP-CAT** in diluted (c = 10^{-5} M) $CHCl_3$ solutions (λ_{ex} = 470 nm).

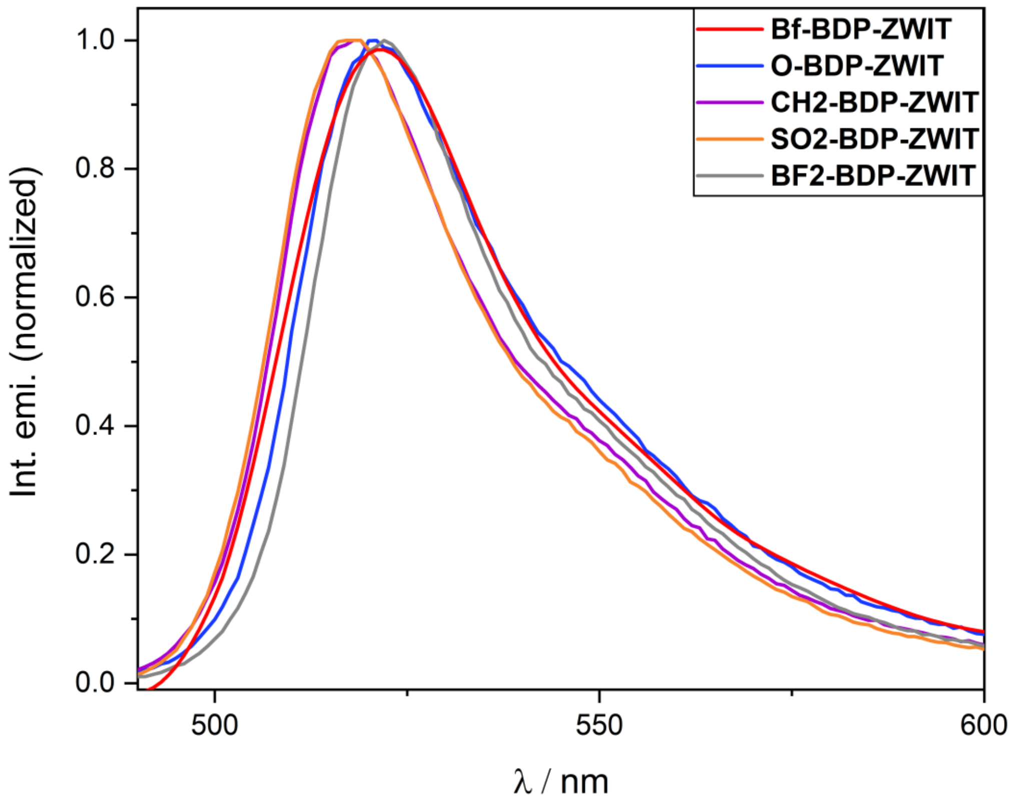

Figure S8. Normalized emission spectra of **X-BDP-ZWIT** in diluted (c = 10^{-5} M) $CHCl_3$ solutions (λ_{ex} = 470 nm).

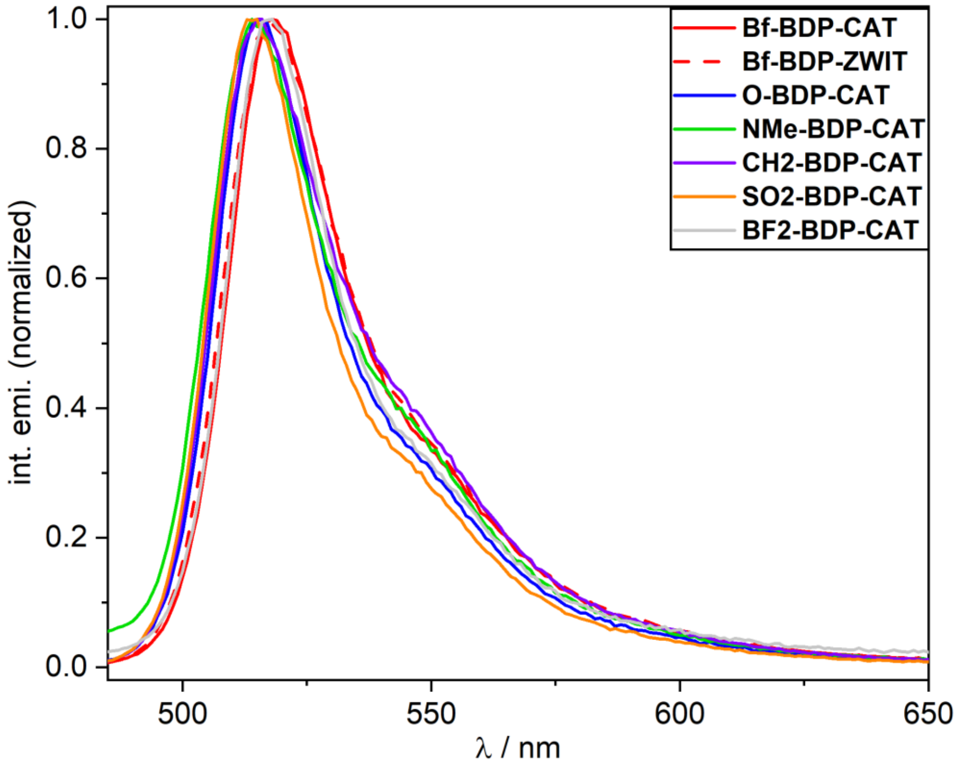

Figure S9. Normalized emission spectra of **X-BDP-CAT** and **Bf-BDP-ZWIT** in dilute (c = 10^{-5} M) water/DMSO (1/1 v/v) solutions (λ_{ex} = 470 nm).

3.2 Transient absorption experiments

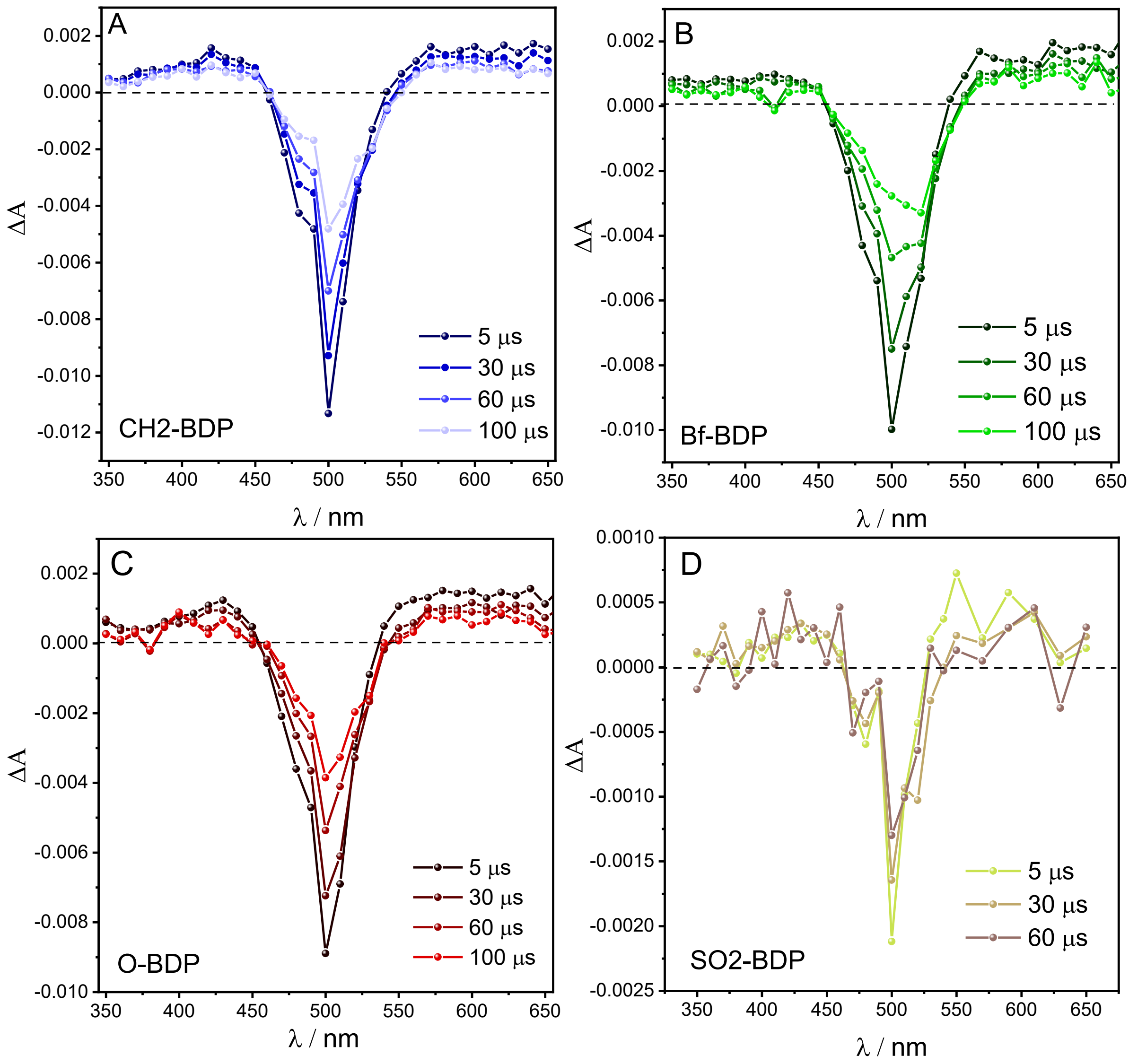

Figure S10. Transient absorption spectra obtained during laser flash photolysis (with excitation at 480 nm) of deoxygenated solutions of A) **CH2-BDP**; B) **Bf-BDP** C) **O-BDP**; D) **SO2-BDP** in chloroform; time delay after flash from 5 μs to 100 μs.

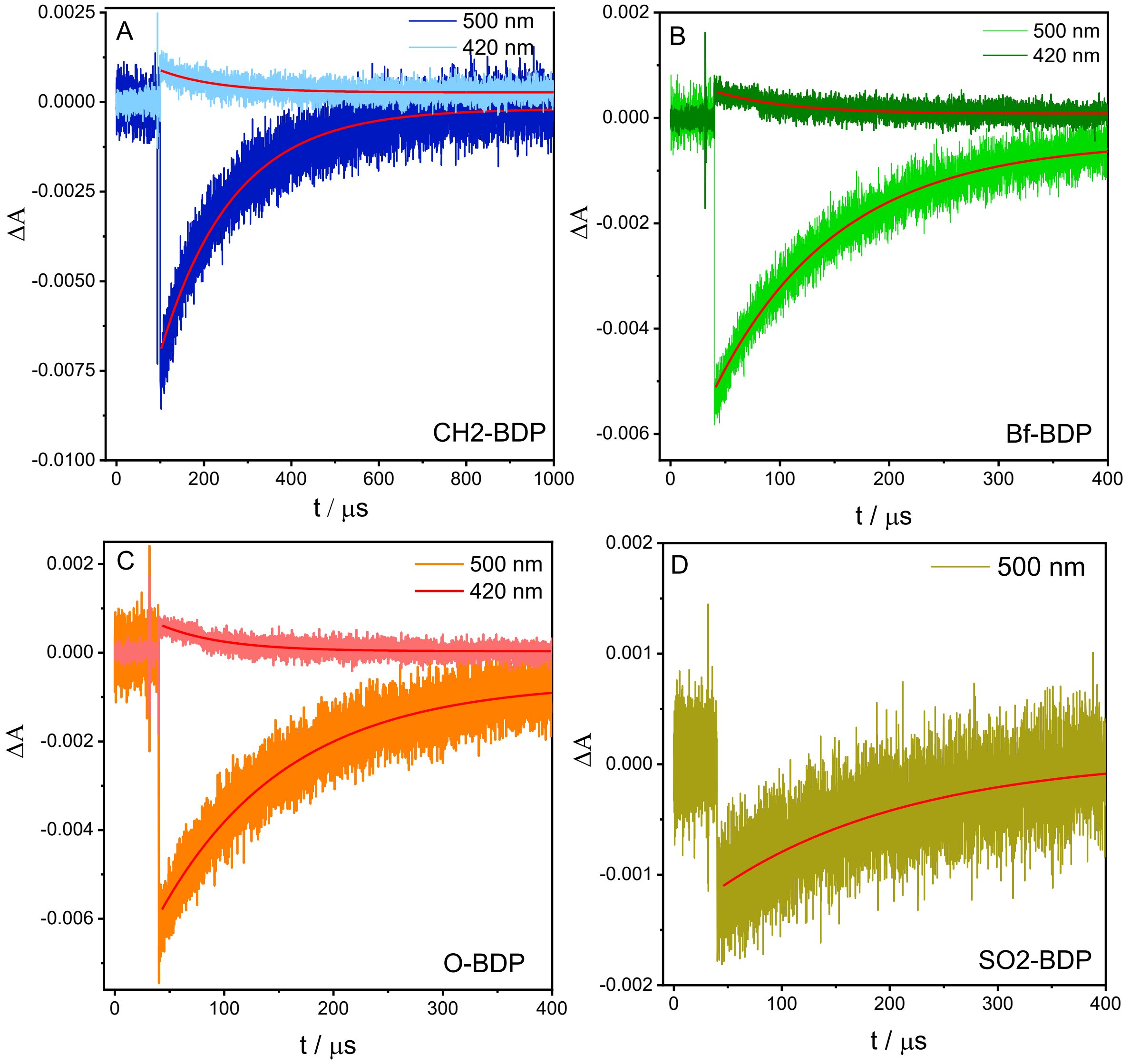

Figure S11. Decay profiles monitored at 500 nm and 420 nm obtained during laser flash photolysis (with excitation at 480 nm) of deoxygenated solutions of A) **CH2-BDP**; B) **Bf-BDP** C) **O-BDP**; D) **SO2-BDP** in chloroform; red lines show monoexponential fits.

3.3. Fluorescence decays

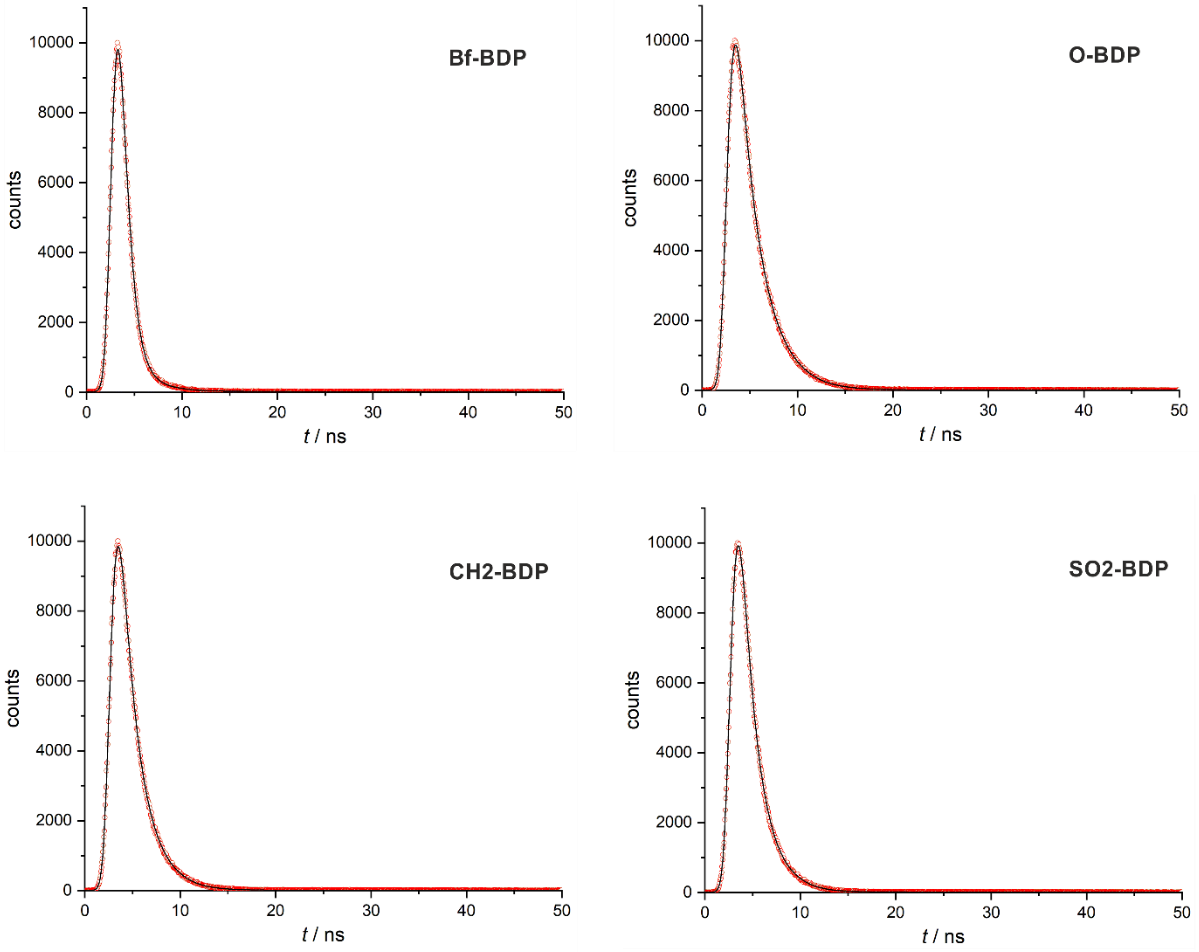

Figure S12. Fluorescence decays of **X-BDP** in $CHCl_3$.

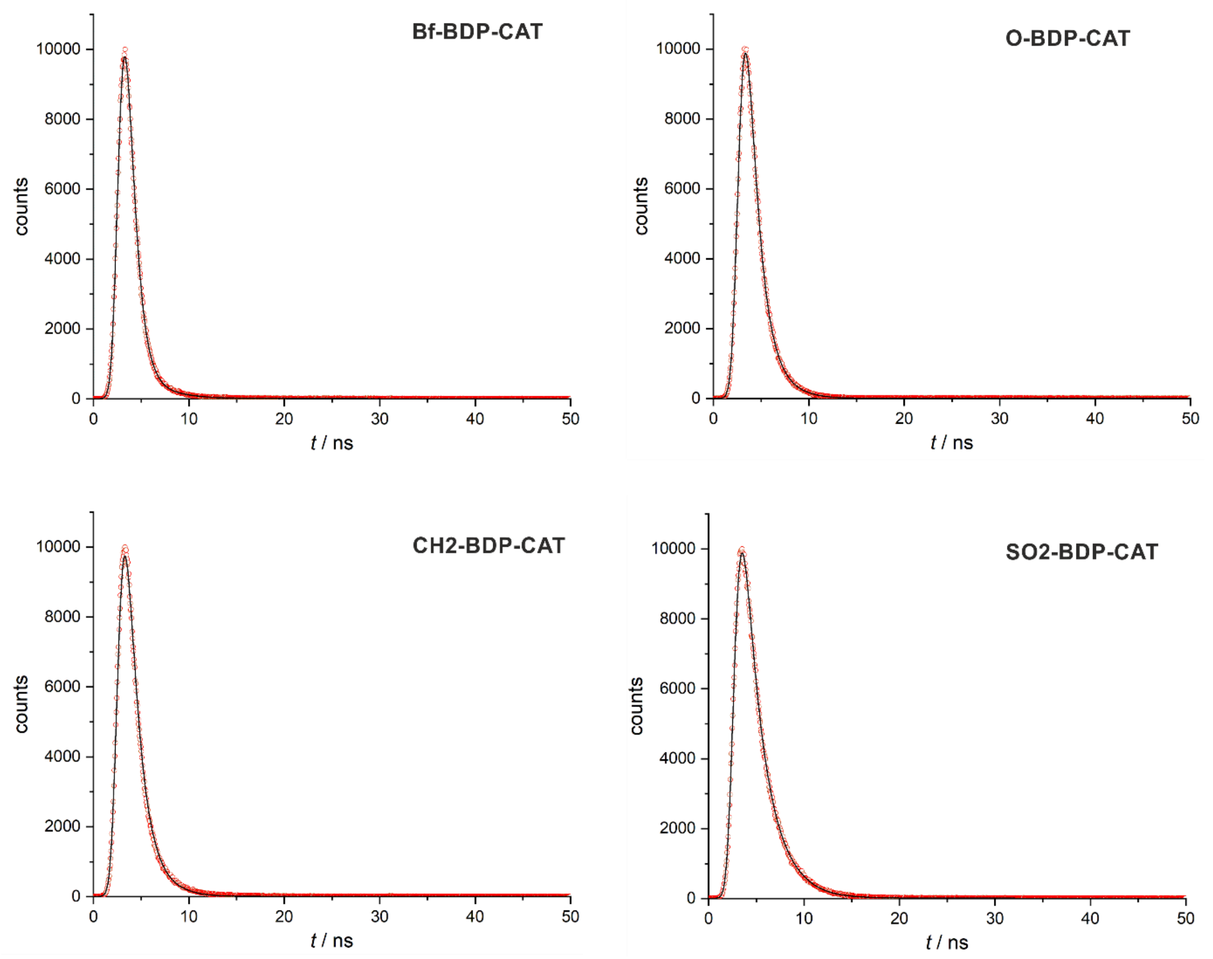

Figure S13. Fluorescence decays of **X-BDP-CAT** in $CHCl_3$.

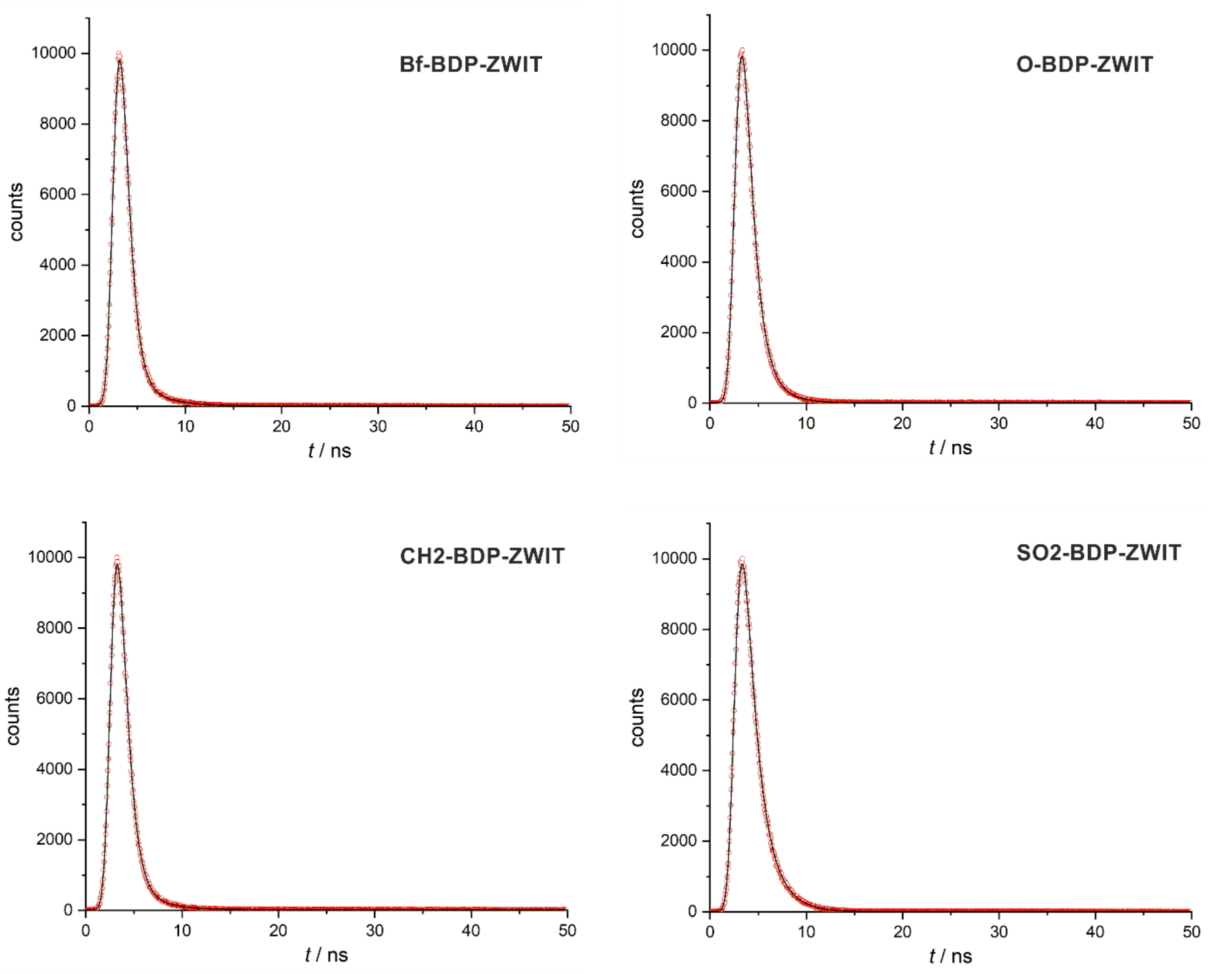

Figure S14. Fluorescence decays of **X-BDP-ZWIT** in $CHCl_3$.

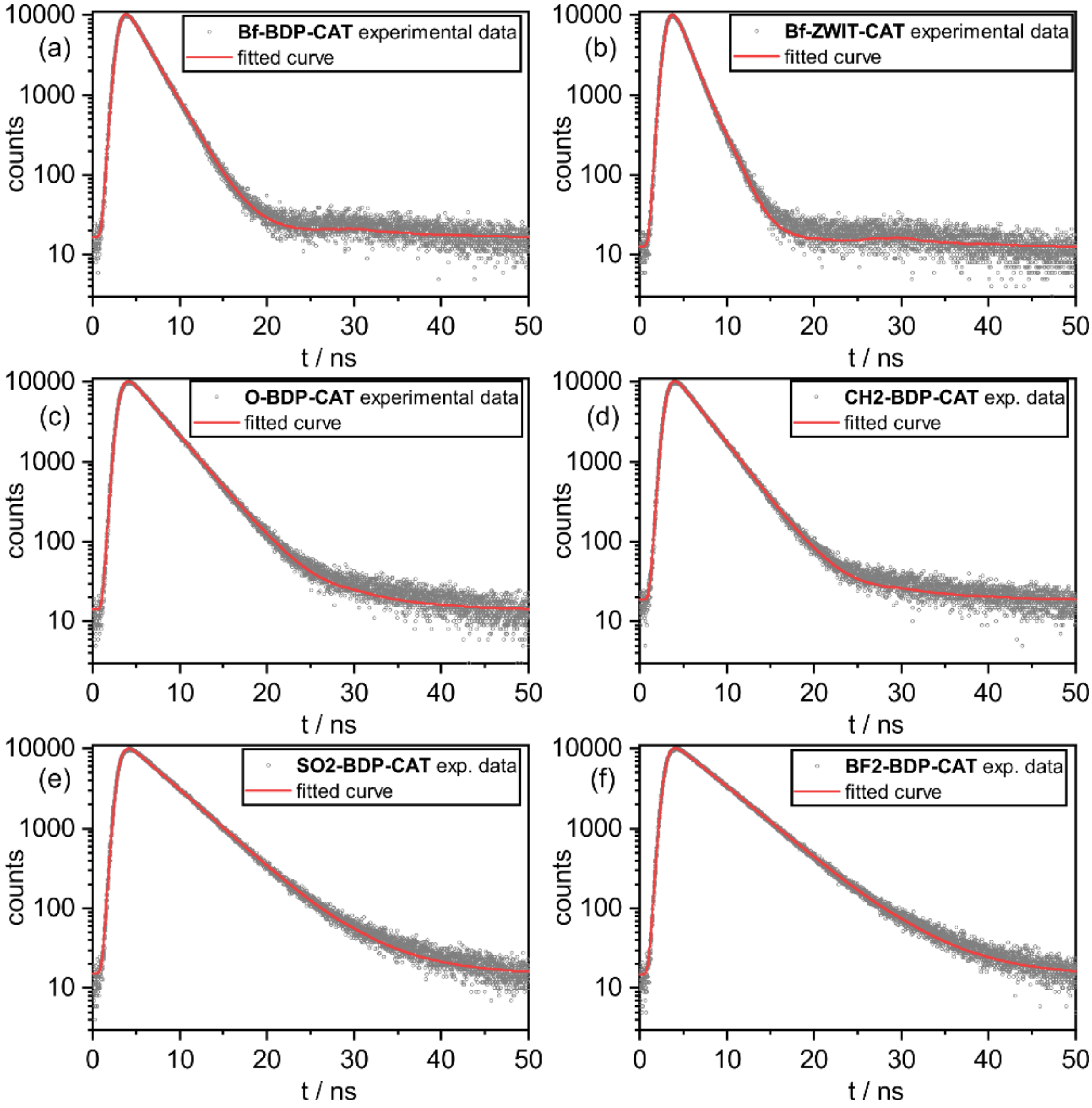

Figure S15. Fluorescence decays of **X-BDP-CAT** and **Bf-BDP-ZWIT** in water/DMSO (1/1 v/v) solutions.

3.4. Photostability studies

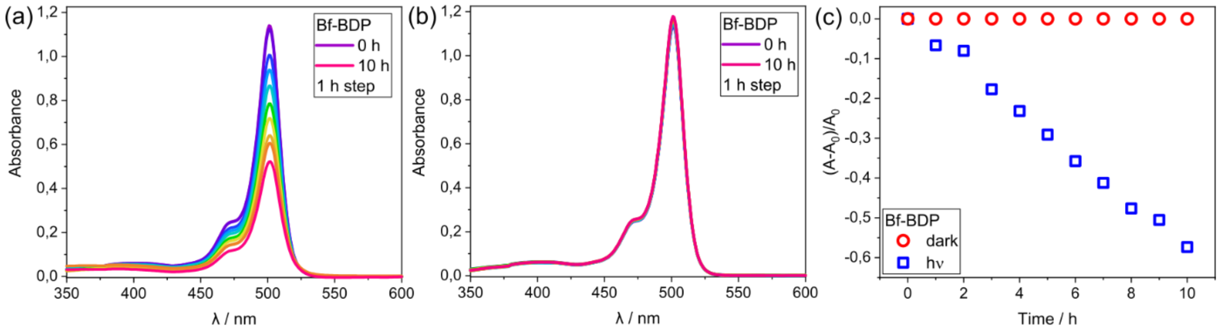

Figure S16. (a) Absorption spectra of **Bf-BDP** recorded at 1 h intervals under irradiation with neutral-white light (26 W) and (b) in the dark. (c) Relative changes in the absorption spectra compared to the initial sample upon irradiation (blue squares) and in the dark (red circles).

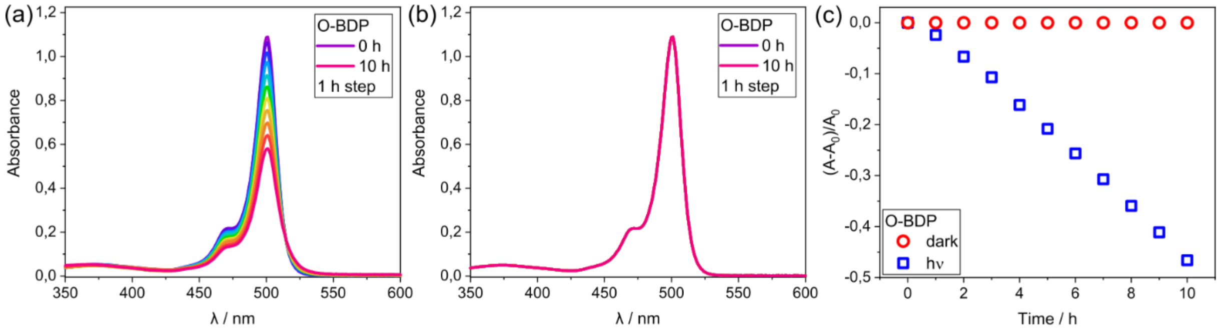

Figure S17. (a) Absorption spectra of **O-BDP** recorded at 1 h intervals under irradiation with neutral-white light (26 W) and (b) in the dark. (c) Relative changes in the absorption spectra compared to the initial sample upon irradiation (blue squares) and in the dark (red circles).

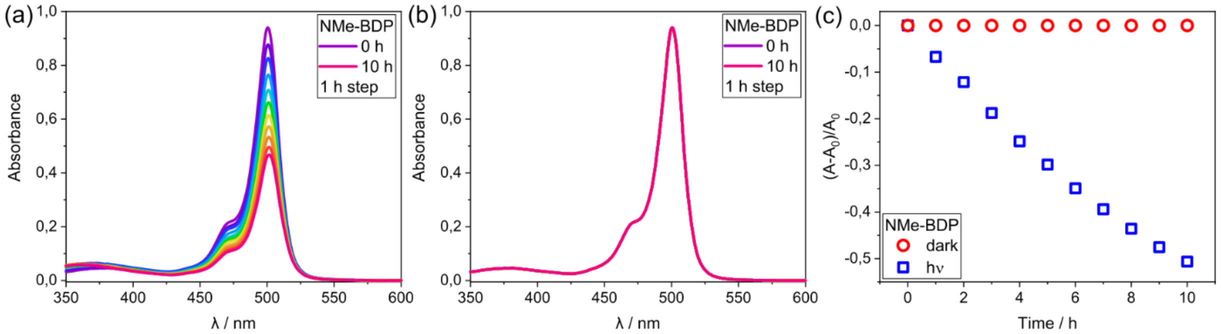

Figure S18. (a) Absorption spectra of **NMe-BDP** recorded at 1 h intervals under irradiation with neutral-white light (26 W) and (b) in the dark. (c) Relative changes in the absorption spectra compared to the initial sample upon irradiation (blue squares) and in the dark (red circles).

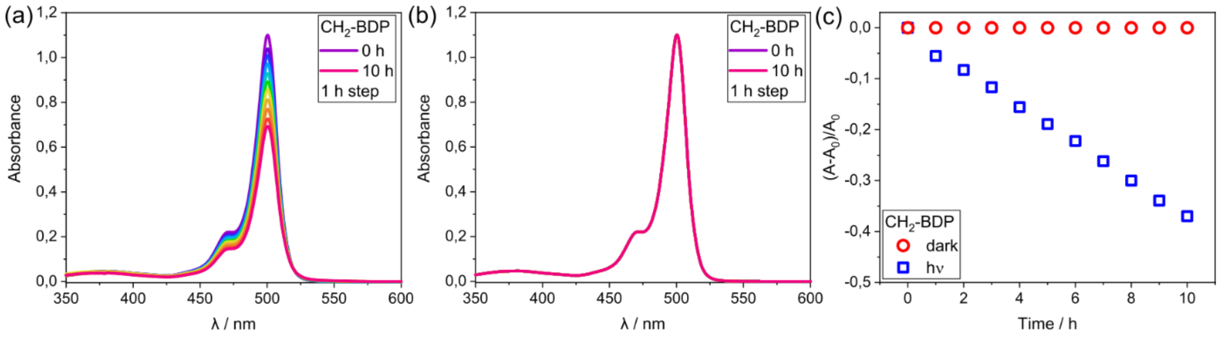

Figure S19. (a) Absorption spectra of **CH2-BDP** recorded at 1 h intervals under irradiation with neutral-white light (26 W) and (b) in the dark. (c) Relative changes in the absorption spectra compared to the initial sample upon irradiation (blue squares) and in the dark (red circles).

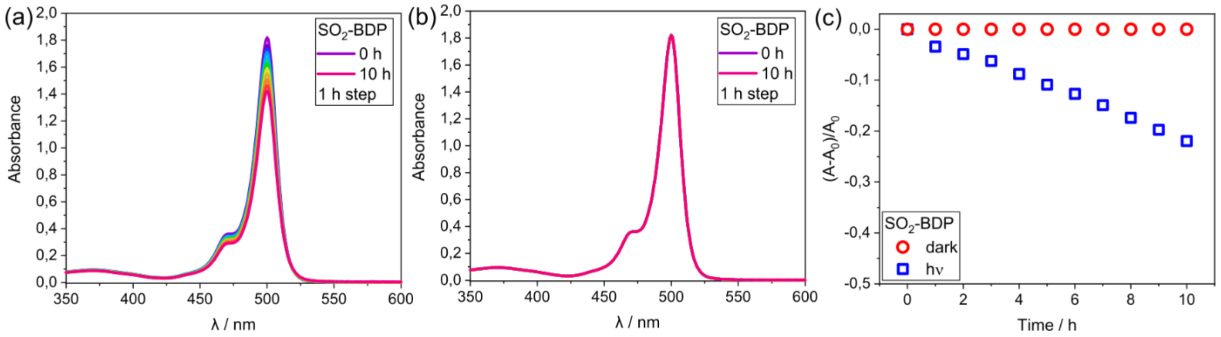

Figure S20. (a) Absorption spectra of **SO2-BDP** recorded at 1 h intervals under irradiation with neutral-white light (26 W) and (b) in the dark. (c) Relative changes in the absorption spectra compared to the initial sample upon irradiation (blue squares) and in the dark (red circles).

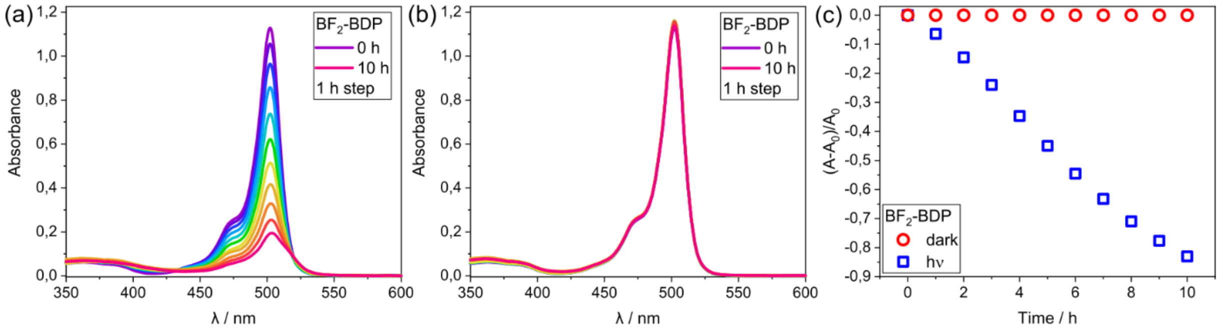

Figure S21. (a) Absorption spectra of **BF2-BDP** recorded at 1 h intervals under irradiation with neutral-white light (26 W) and (b) in the dark. (c) Relative changes in the absorption spectra compared to the initial sample upon irradiation (blue squares) and in the dark (red circles).

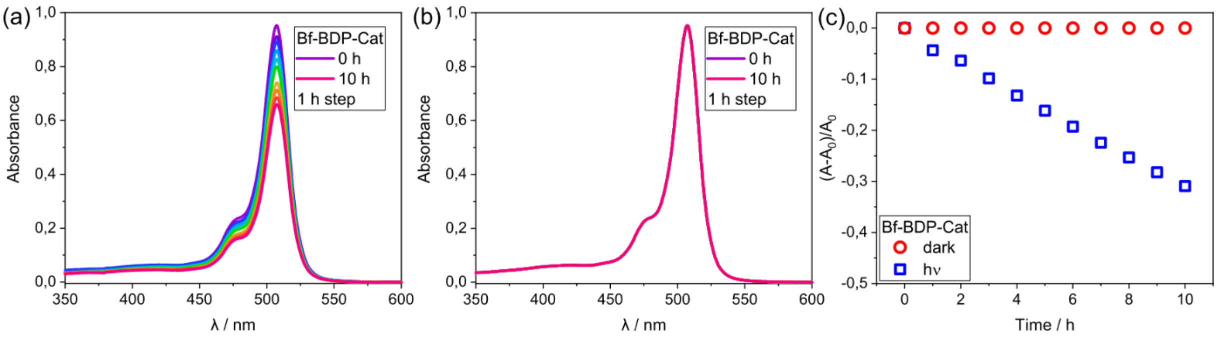

Figure S22. (a) Absorption spectra of **Bf-BDP-CAT** recorded at 1 h intervals under irradiation with neutral-white light (26 W) and (b) in the dark. (c) Relative changes in the absorption spectra compared to the initial sample upon irradiation (blue squares) and in the dark (red circles).

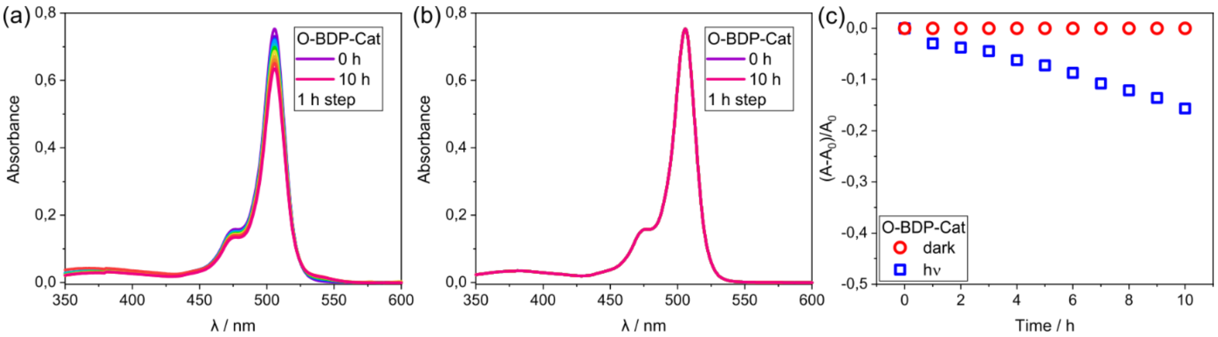

Figure S23. (a) Absorption spectra of **O-BDP-CAT** recorded at 1 h intervals under irradiation with neutral-white light (26 W) and (b) in the dark. (c) Relative changes in the absorption spectra compared to the initial sample upon irradiation (blue squares) and in the dark (red circles).

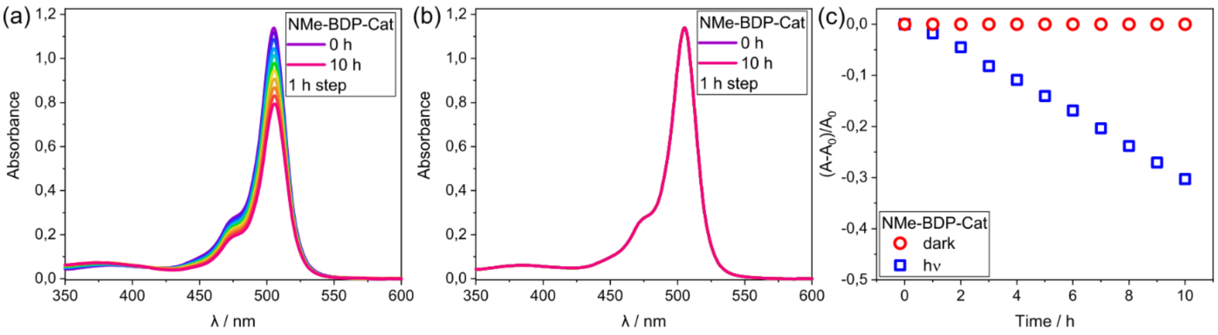

Figure S24. (a) Absorption spectra of **NMe-BDP-CAT** recorded at 1 h intervals under irradiation with neutral-white light (26 W) and (b) in the dark. (c) Relative changes in the absorption spectra compared to the initial sample upon irradiation (blue squares) and in the dark (red circles).

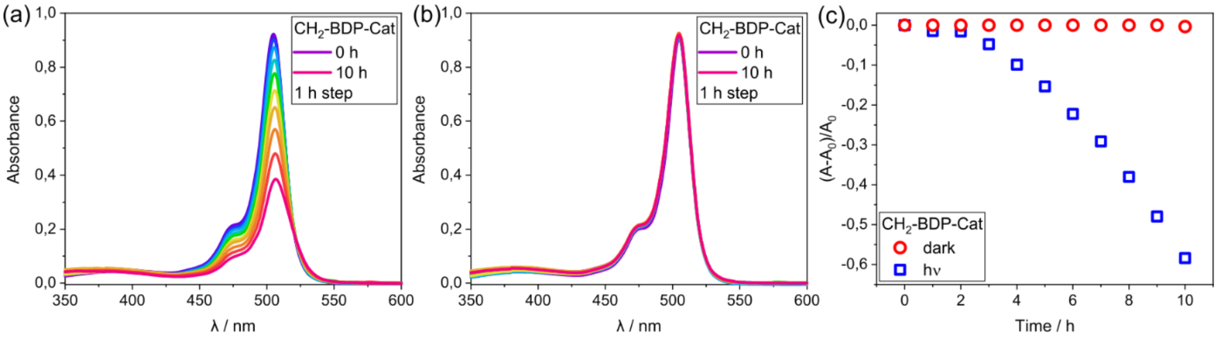

Figure S25. (a) Absorption spectra of **CH2-BDP-CAT** recorded at 1 h intervals under irradiation with neutral-white light (26 W) and (b) in the dark. (c) Relative changes in the absorption spectra compared to the initial sample upon irradiation (blue squares) and in the dark (red circles).

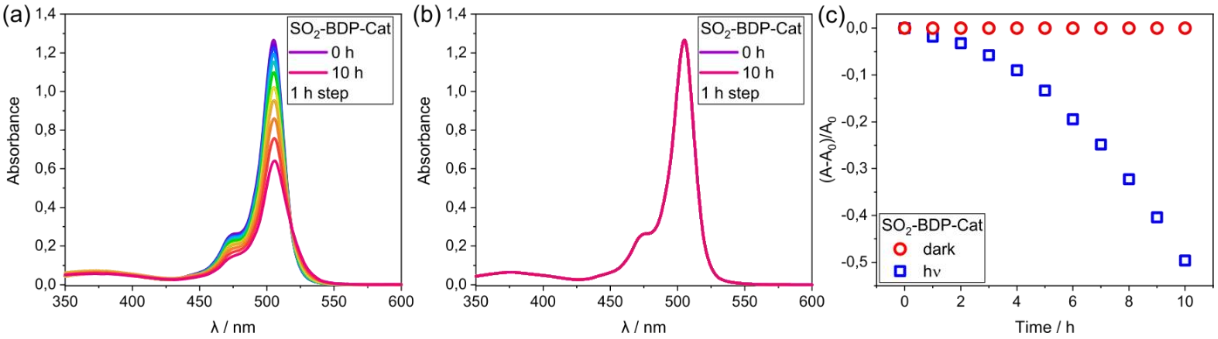

Figure S26. (a) Absorption spectra of **SO2-BDP-CAT** recorded at 1 h intervals under irradiation with neutral-white light (26 W) and (b) in the dark. (c) Relative changes in the absorption spectra compared to the initial sample upon irradiation (blue squares) and in the dark (red circles).

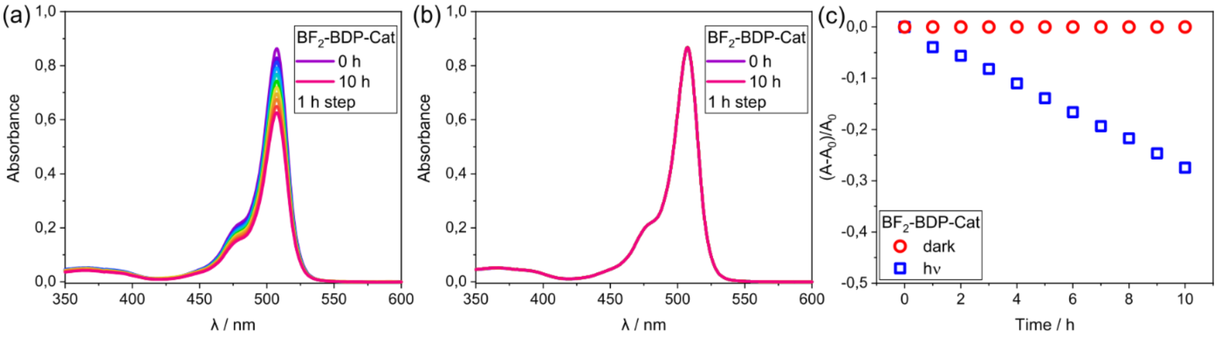

Figure S27. (a) Absorption spectra of **BF2-BDP-CAT** recorded at 1 h intervals under irradiation with neutral-white light (26 W) and (b) in the dark. (c) Relative changes in the absorption spectra compared to the initial sample upon irradiation (blue squares) and in the dark (red circles).

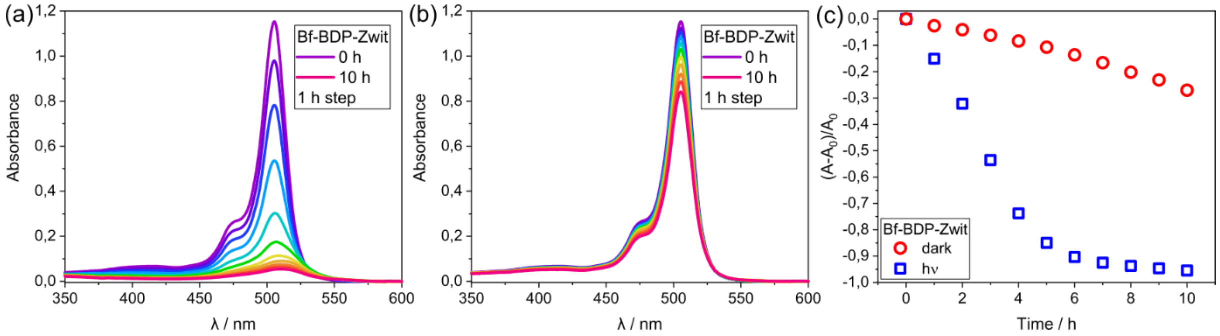

Figure S28. (a) Absorption spectra of **Bf-BDP-ZWIT** recorded at 1 h intervals under irradiation with neutral-white light (26 W) and (b) in the dark. (c) Relative changes in the absorption spectra compared to the initial sample upon irradiation (blue squares) and in the dark (red circles).

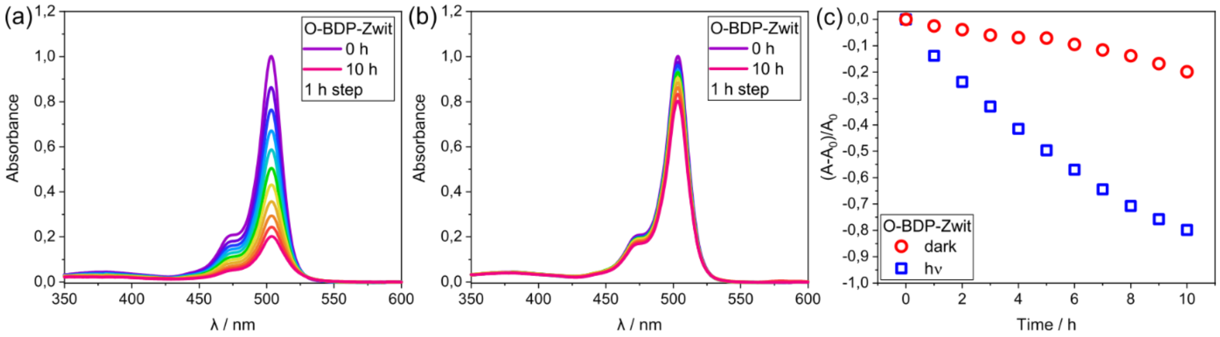

Figure S29. (a) Absorption spectra of **O-BDP-ZWIT** recorded at 1 h intervals under irradiation with neutral-white light (26 W) and (b) in the dark. (c) Relative changes in the absorption spectra compared to the initial sample upon irradiation (blue squares) and in the dark (red circles).

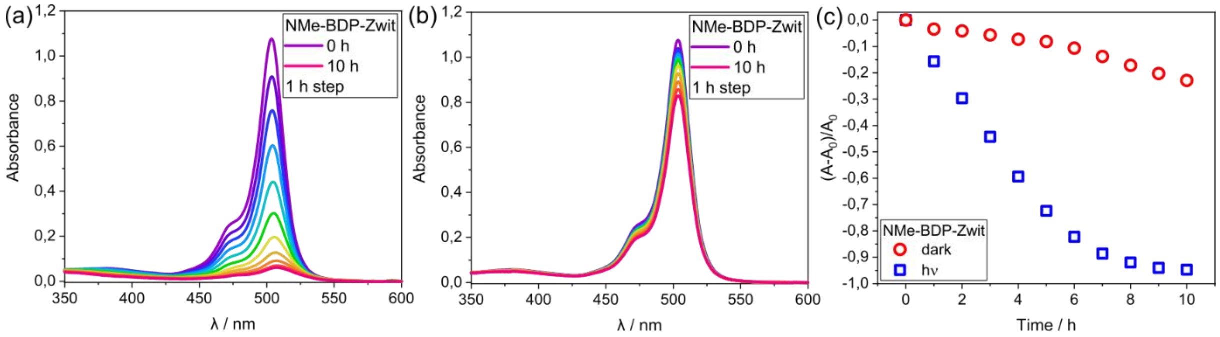

Figure S30. (a) Absorption spectra of **NMe-BDP-ZWIT** recorded at 1 h intervals under irradiation with neutral-white light (26 W) and (b) in the dark. (c) Relative changes in the absorption spectra compared to the initial sample upon irradiation (blue squares) and in the dark (red circles).

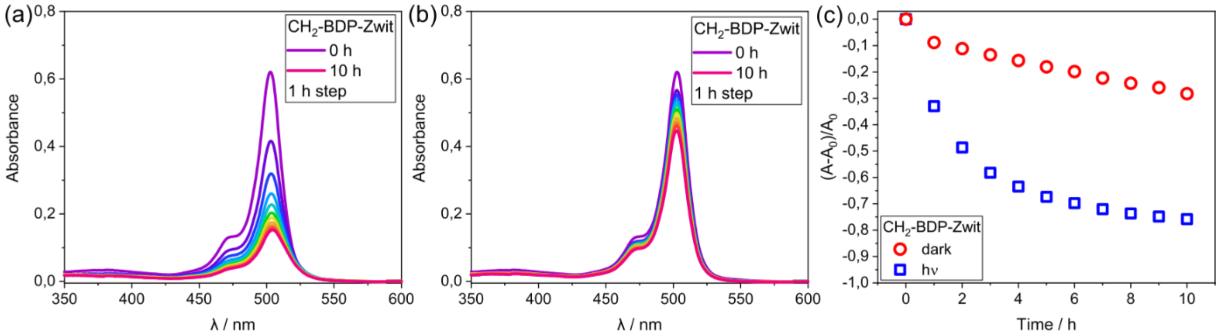

Figure S31. (a) Absorption spectra of **CH2-BDP-ZWIT** recorded at 1 h intervals under irradiation with neutral-white light (26 W) and (b) in the dark. (c) Relative changes in the absorption spectra compared to the initial sample upon irradiation (blue squares) and in the dark (red circles).

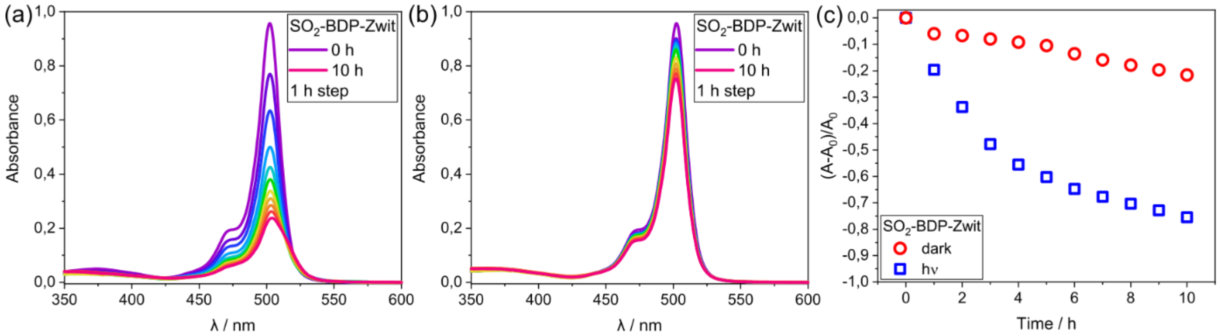

Figure S32. (a) Absorption spectra of **SO2-BDP-ZWIT** recorded at 1 h intervals under irradiation with neutral-white light (26 W) and (b) in the dark. (c) Relative changes in the absorption spectra compared to the initial sample upon irradiation (blue squares) and in the dark (red circles).

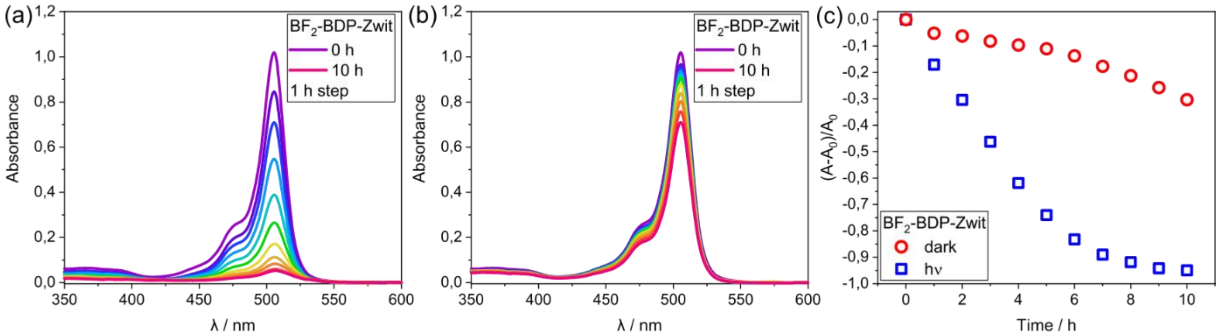

Figure S33. (a) Absorption spectra of **BF2-BDP-ZWIT** recorded at 1 h intervals under irradiation with neutral-white light (26 W) and (b) in the dark. (c) Relative changes in the absorption spectra compared to the initial sample upon irradiation (blue squares) and in the dark (red circles).

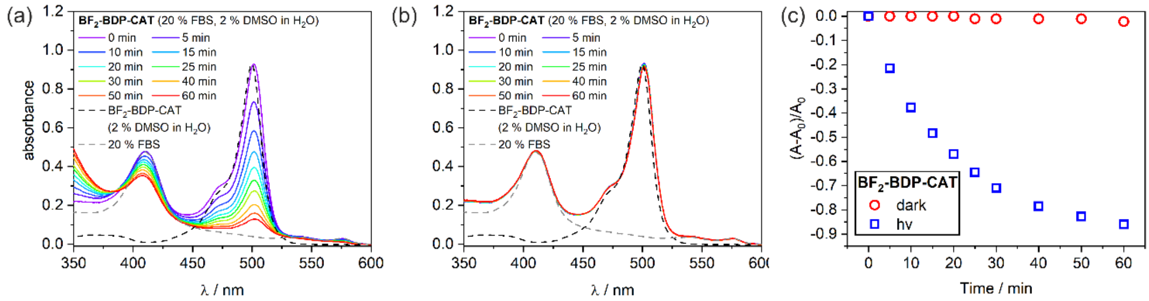

Figure S34. (a) Absorption spectra of **BF2-BDP-CAT** in 20 % (v/v) serum in water under irradiation with neutral-white light (26 W) and (b) in the dark. (c) Relative changes in the absorption spectra compared to the initial sample upon irradiation (blue squares) and in the dark (red circles).

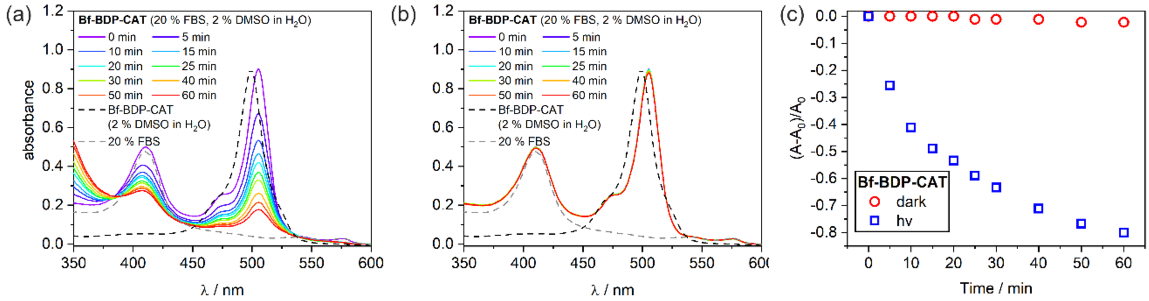

Figure S35. (a) Absorption spectra of **Bf-BDP-CAT** in 20 % (v/v) serum in water under irradiation with neutral-white light (26 W) and (b) in the dark. (c) Relative changes in the absorption spectra compared to the initial sample upon irradiation (blue squares) and in the dark (red circles).

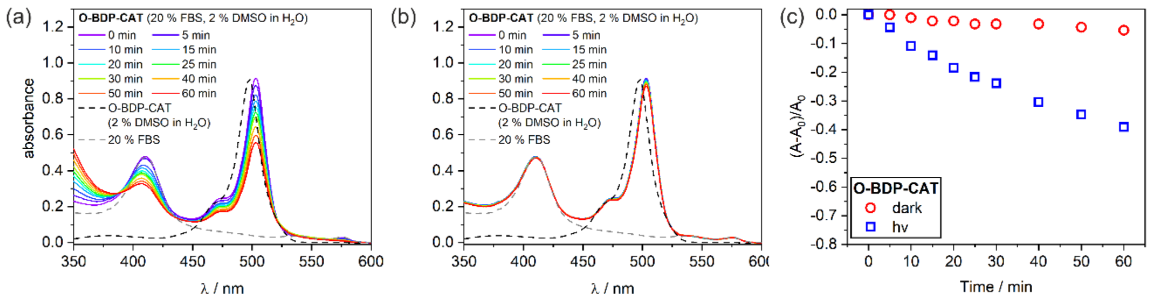

Figure S36. (a) Absorption spectra of **O-BDP-CAT** in 20 % (v/v) serum in water under irradiation with neutral-white light (26 W) and (b) in the dark. (c) Relative changes in the absorption spectra compared to the initial sample upon irradiation (blue squares) and in the dark (red circles).

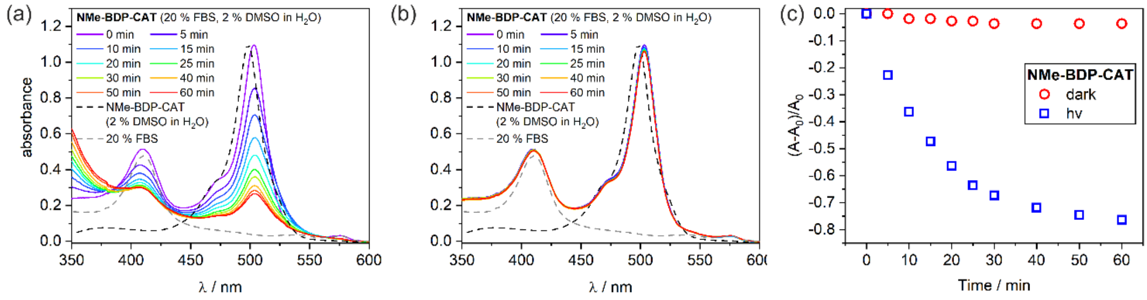

Figure S37. (a) Absorption spectra of **NMe-BDP-CAT** in 20 % (v/v) serum in water under irradiation with neutral-white light (26 W) and (b) in the dark. (c) Relative changes in the absorption spectra compared to the initial sample upon irradiation (blue squares) and in the dark (red circles).

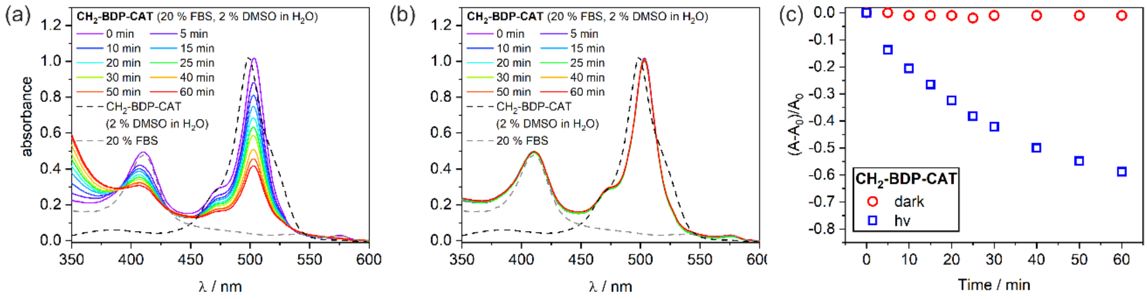

Figure S38. (a) Absorption spectra of **CH2-BDP-CAT** in 20 % (v/v) serum in water under irradiation with neutral-white light (26 W) and (b) in the dark. (c) Relative changes in the absorption spectra compared to the initial sample upon irradiation (blue squares) and in the dark (red circles).

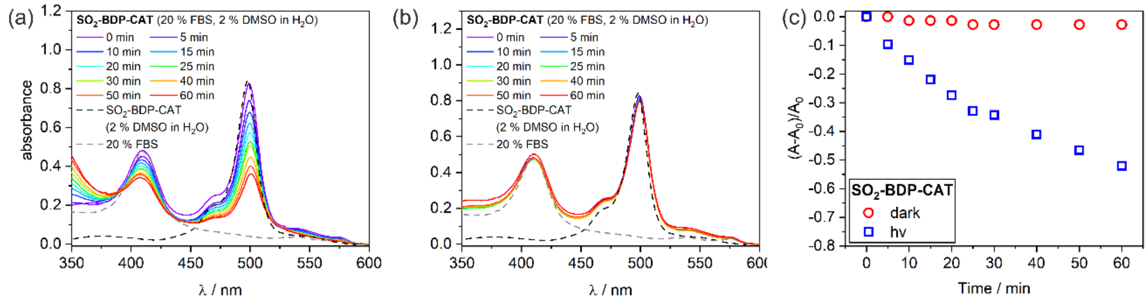

Figure S39. (a) Absorption spectra of **SO2-BDP-CAT** in 20 % (v/v) serum in water under irradiation with neutral-white light (26 W) and (b) in the dark. (c) Relative changes in the absorption spectra compared to the initial sample upon irradiation (blue squares) and in the dark (red circles).

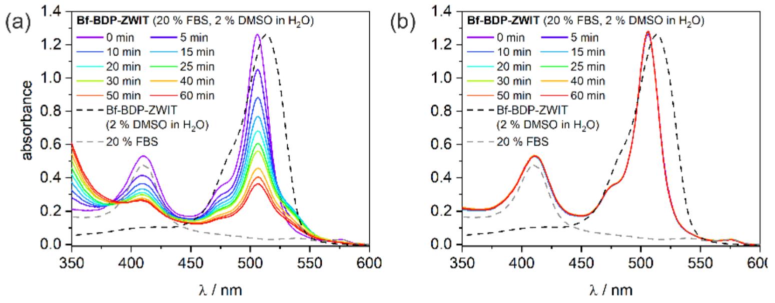

Figure S40. (a) Absorption spectra of **Bf-BDP-ZWIT** in 20 % (v/v) serum in water under irradiation with neutral-white light (26 W) and (b) in the dark. (c) Relative changes in the absorption spectra compared to the initial sample upon irradiation (blue squares) and in the dark (red circles).

3.5. NIR detection of singlet oxygen phosphorescence

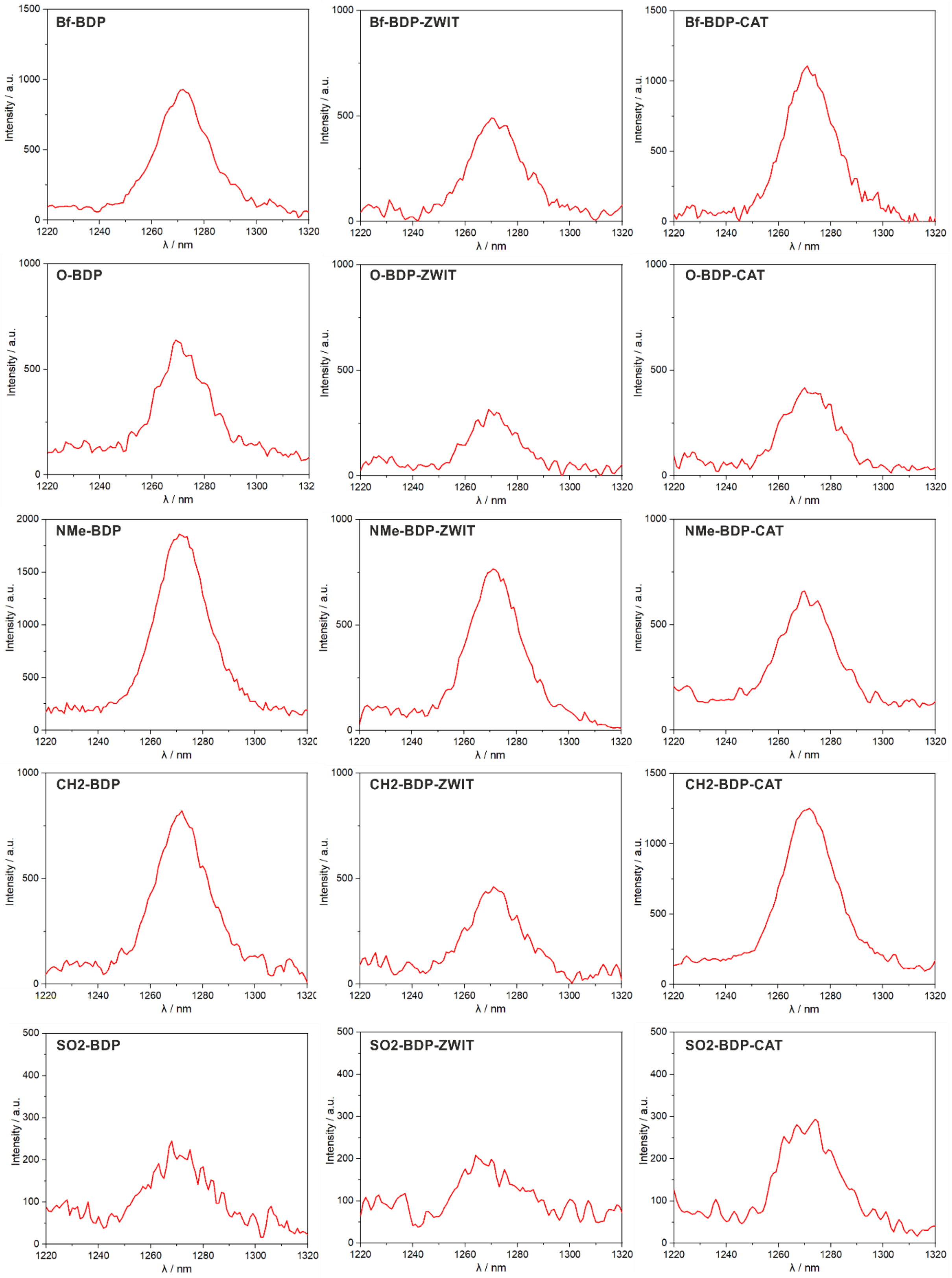

Figure S41. Singlet oxygen phosphorescence observed upon excitation of **X-BDP-R** at maximum absorption wavelength.

4. EPR spectroscopy

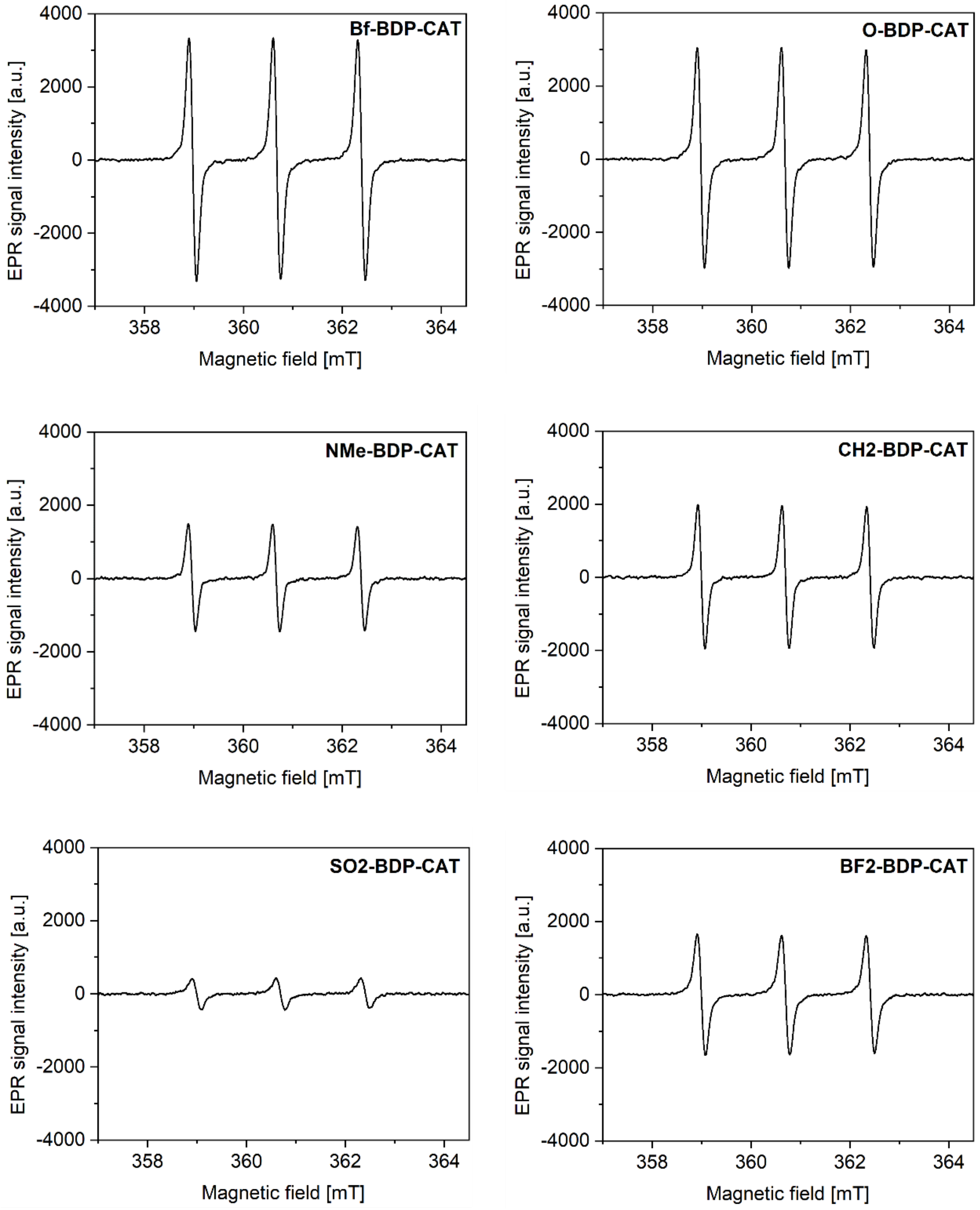

Figure S42. EPR spectra recorded for **X-BDP-CAT** with TEMP as $^1O_2$ trap upon 5 min irradiation with neutral-white light.

5. Electrochemistry

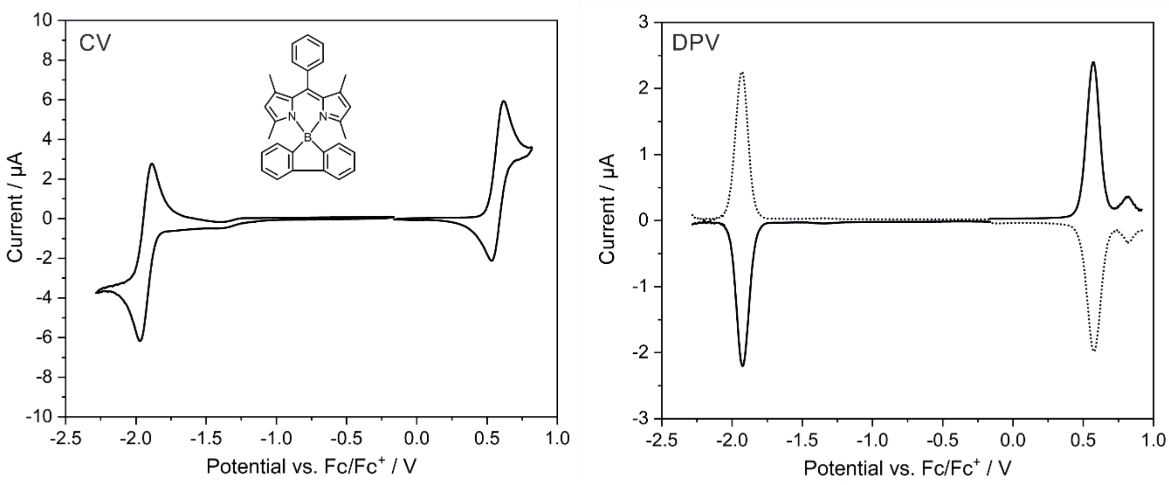

Figure S43. Electrochemical redox processes recorded for **Bf-BDP** with cyclic voltammetry and differential pulse voltammetry (glassy carbon electrode). All potentials are referenced to the half-wave potential of the Fc/Fc^{+} redox pair. Electrolyte: 0.1 M Bu_4NBF_4 CH_2Cl_2, Cyclic voltammetry – scan rate: 50 mV/s. Differential pulse voltammetry – modulation time: 50 ms, modulation amplitude: 10 mV, step potential: 5 mV.

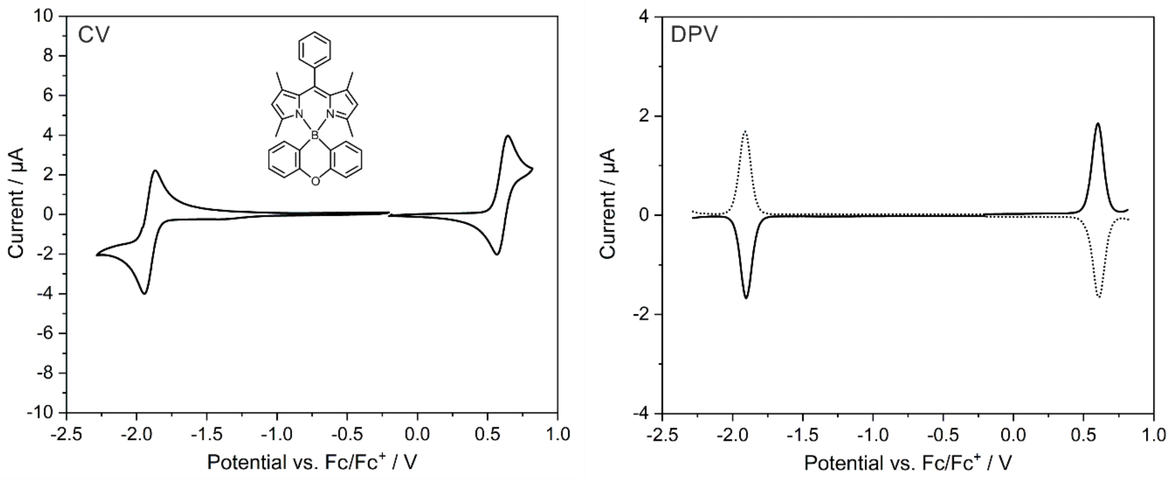

Figure S44. Electrochemical redox processes recorded for **O-BDP** with cyclic voltammetry and differential pulse voltammetry (glassy carbon electrode). All potentials are referenced to the half-wave potential of the Fc/Fc^{+} redox pair. Electrolyte: 0.1 M Bu_4NBF_4 CH_2Cl_2, Cyclic voltammetry – scan rate: 50 mV/s. Differential pulse voltammetry – modulation time: 50 ms, modulation amplitude: 10 mV, step potential: 5 mV.

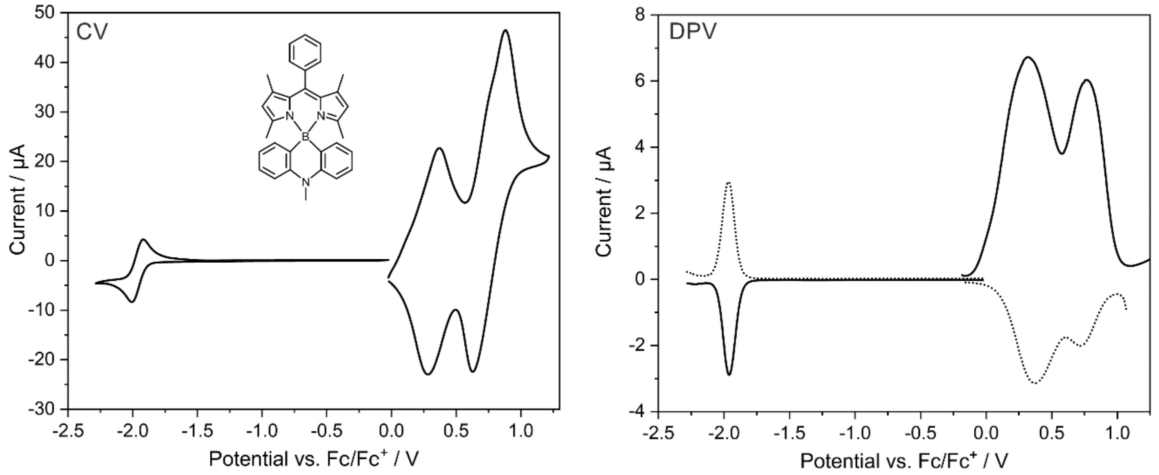

Figure S45. Electrochemical redox processes recorded for **NMe-BDP** with cyclic voltammetry and differential pulse voltammetry (glassy carbon electrode). All potentials are referenced to the half-wave potential of the Fc/Fc^+ redox pair. Large discrepancy between cathodic and anodic currents are clearly visible, most likely, this results from differences in adhesion to the electrode surface, which - in this case - are observed only within the anodic potential range. Confirmation of this hypothesis requires further investigation. Electrolyte: 0.1 M Bu_4NBF_4 CH_2Cl_2, Cyclic voltammetry – scan rate: 50 mV/s. Differential pulse voltammetry – modulation time: 50 ms, modulation amplitude: 10 mV, step potential: 5 mV.

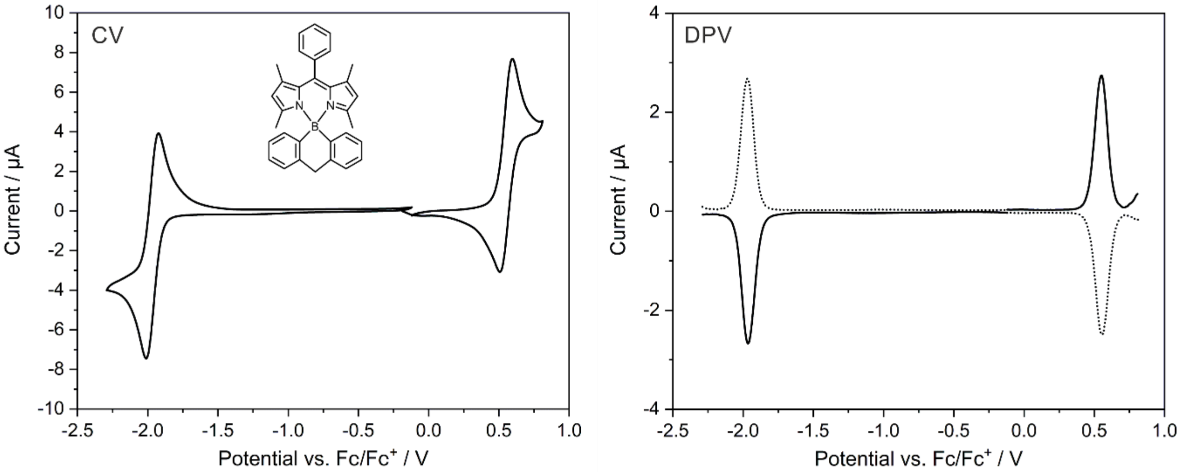

Figure S46. Electrochemical redox processes recorded for **CH2-BDP** with cyclic voltammetry and differential pulse voltammetry (glassy carbon electrode). All potentials are referenced to the half-wave potential of the Fc/Fc^+ redox pair. Electrolyte: 0.1 M Bu_4NBF_4 CH_2Cl_2, Cyclic voltammetry – scan rate: 50 mV/s. Differential pulse voltammetry – modulation time: 50 ms, modulation amplitude: 10 mV, step potential: 5 mV.

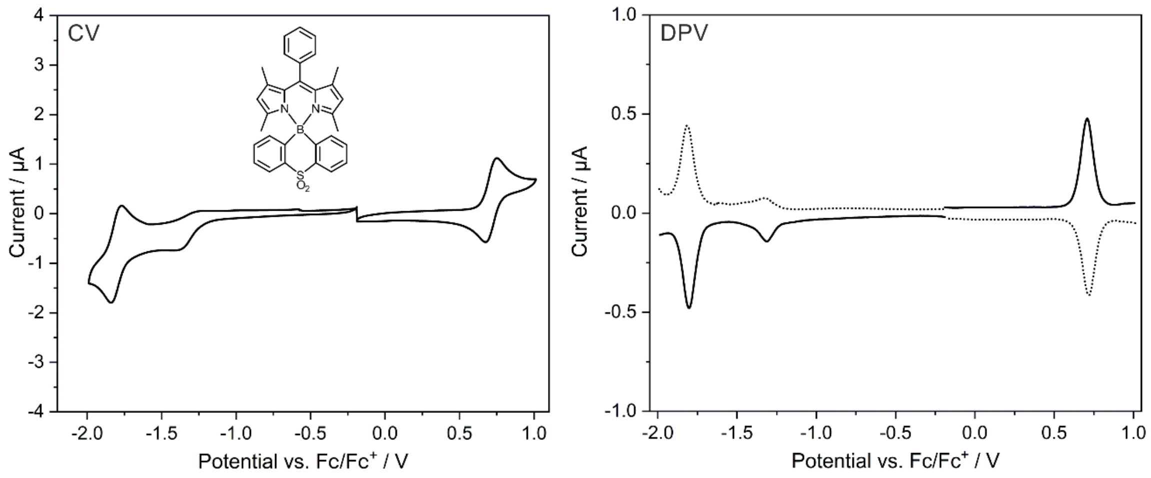

Figure S47. Electrochemical redox processes recorded for **SO2-BDP** with cyclic voltammetry and differential pulse voltammetry (glassy carbon electrode). All potentials are referenced to the half-wave potential of the Fc/Fc$^+$ redox pair. The reduction of oxygen is visible at ca. -1.2 eV vs. Fc/Fc+. Electrolyte: 0.1 M Bu_4NBF_4 CH_2Cl_2, Cyclic voltammetry – scan rate: 50 mV/s. Differential pulse voltammetry – modulation time: 50 ms, modulation amplitude: 10 mV, step potential: 5 mV.

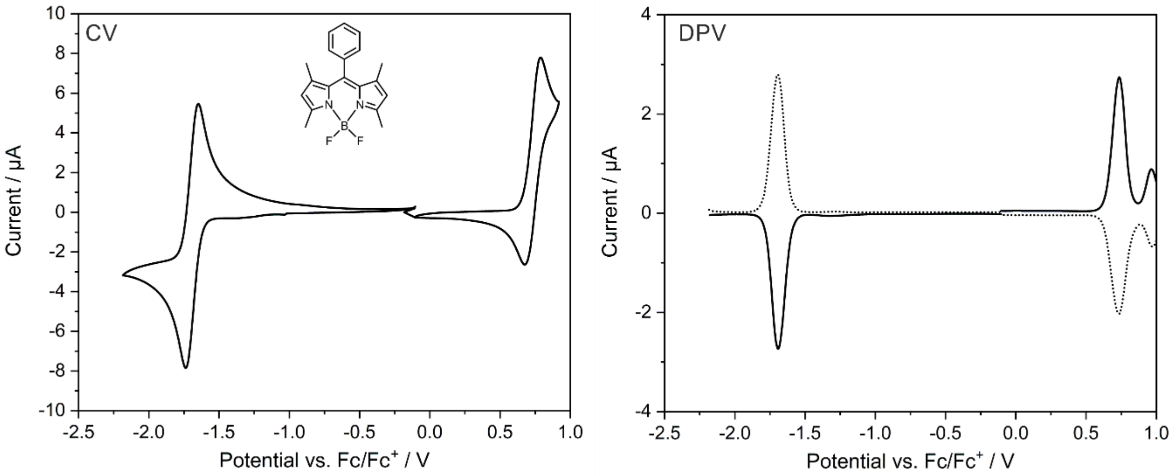

Figure S48. Electrochemical redox processes recorded for **BF2-BDP** with cyclic voltammetry and differential pulse voltammetry (glassy carbon electrode). All potentials are referenced to the half-wave potential of the Fc/Fc$^+$ redox pair. The reduction of oxygen is visible at ca. -1.2 eV vs. Fc/Fc+. Electrolyte: 0.1 M Bu_4NBF_4 CH_2Cl_2, Cyclic voltammetry – scan rate: 50 mV/s. Differential pulse voltammetry – modulation time: 50 ms, modulation amplitude: 10 mV, step potential: 5 mV.

6. ROS photogeneration

Photoreactor for laboratory experiments:

The photoreactor is built of an aluminium tube (Φ = 150) equipped with a neutral-white LED strip with 56 diodes (40 mW·cm^{-2}; CIE 1931 coordinates: 0.38, 0.38; 4000 K) and a plastic cover which allows for the simultaneous conduction of eight reactions in 4 mL vials. The LED strip is attached to the inner wall of the aluminium tube. The cover was equipped with a fan (Ø=60 mm) with air diffusor, while the aluminium tube was cooled by a copper coiled tube heat exchanger sticked to the outside wall of the reactor. Temperature inside reactor was controlled by placing Pt-100 thermometer into one of the reaction vials filled with AcOEt. Under such conditions the temperature inside the reactor was maintained at 25 °C.

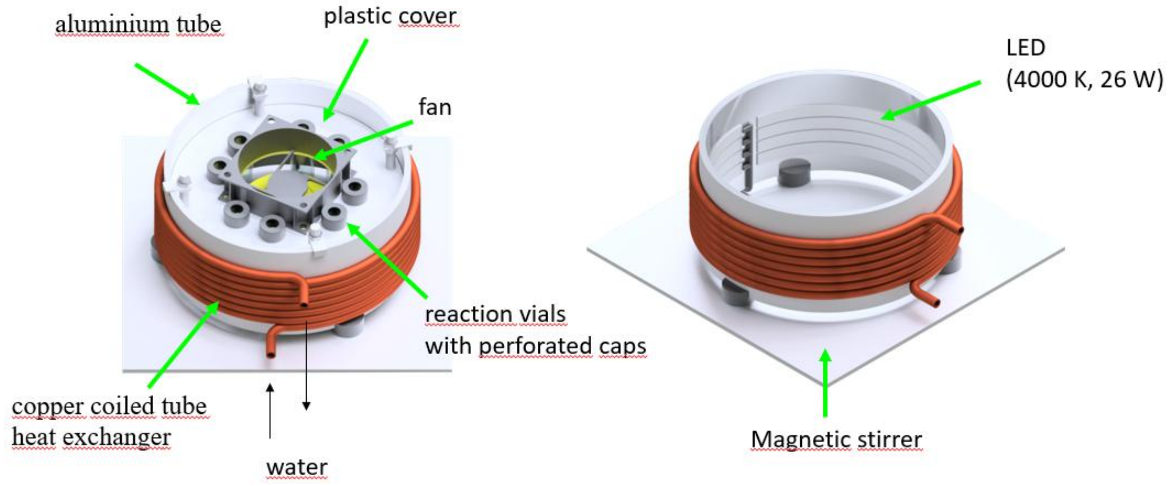

Figure S49. Schematic model of the photoreactor used in photooxidation of 2-furoic acid and photostability studies.

Photoreactor for biological experiments:

The custom-built LED illumination system consisted of four stainless-steel threaded rods forming an adjustable frame for aluminium plate (20 × 23 cm) acting as a mechanical support and thermal radiator for the light source. White LED strips (14 strips × 48 diodes = 672 diodes in total; CIE 1931 coordinates: 0.38, 0.38; 4000 K) was glued on the bottom of the plate. They provide a homogeneous irradiation over the working area corresponding to a standard 96-well plate positioned centrally beneath the LEDs. In all experiments, the distance between the light source and the plate was fixed at 10 cm, ensuring homogeneous light distribution (40 mW·cm^{-2}) without a measurable rise in temperature within the incubator.

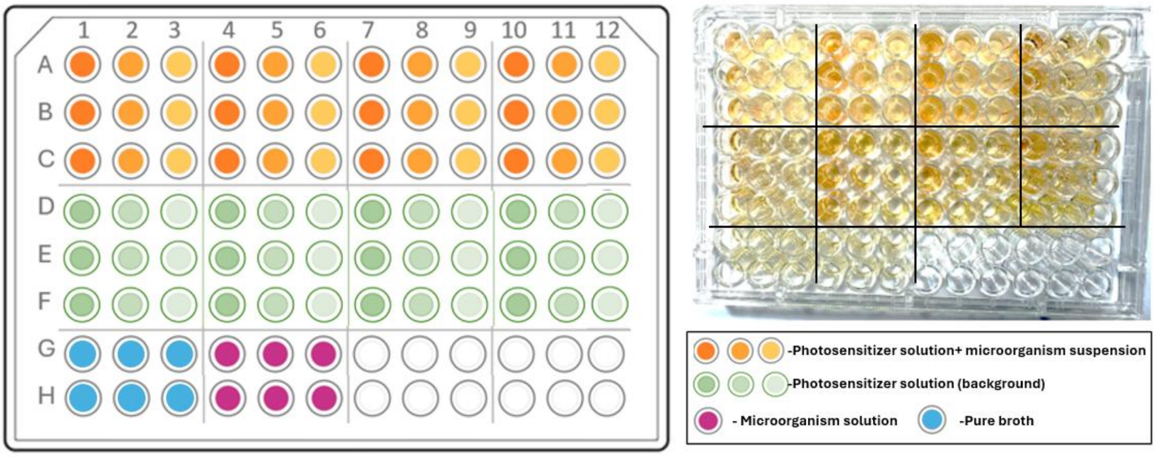

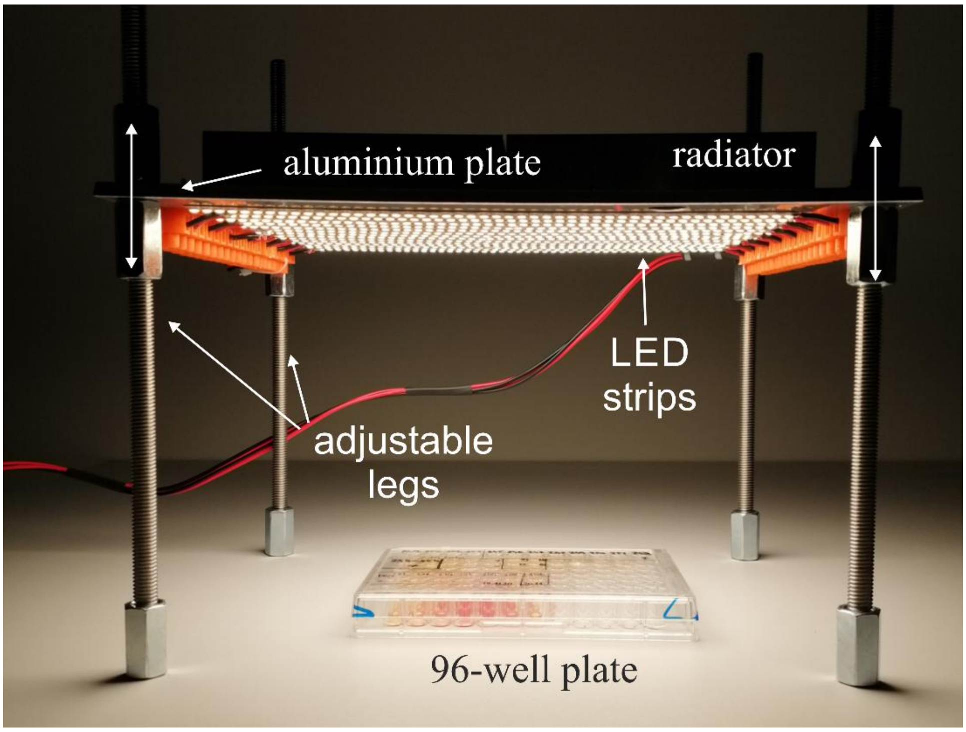

Figure S50. Irradiation device used in photoinactivation studies.

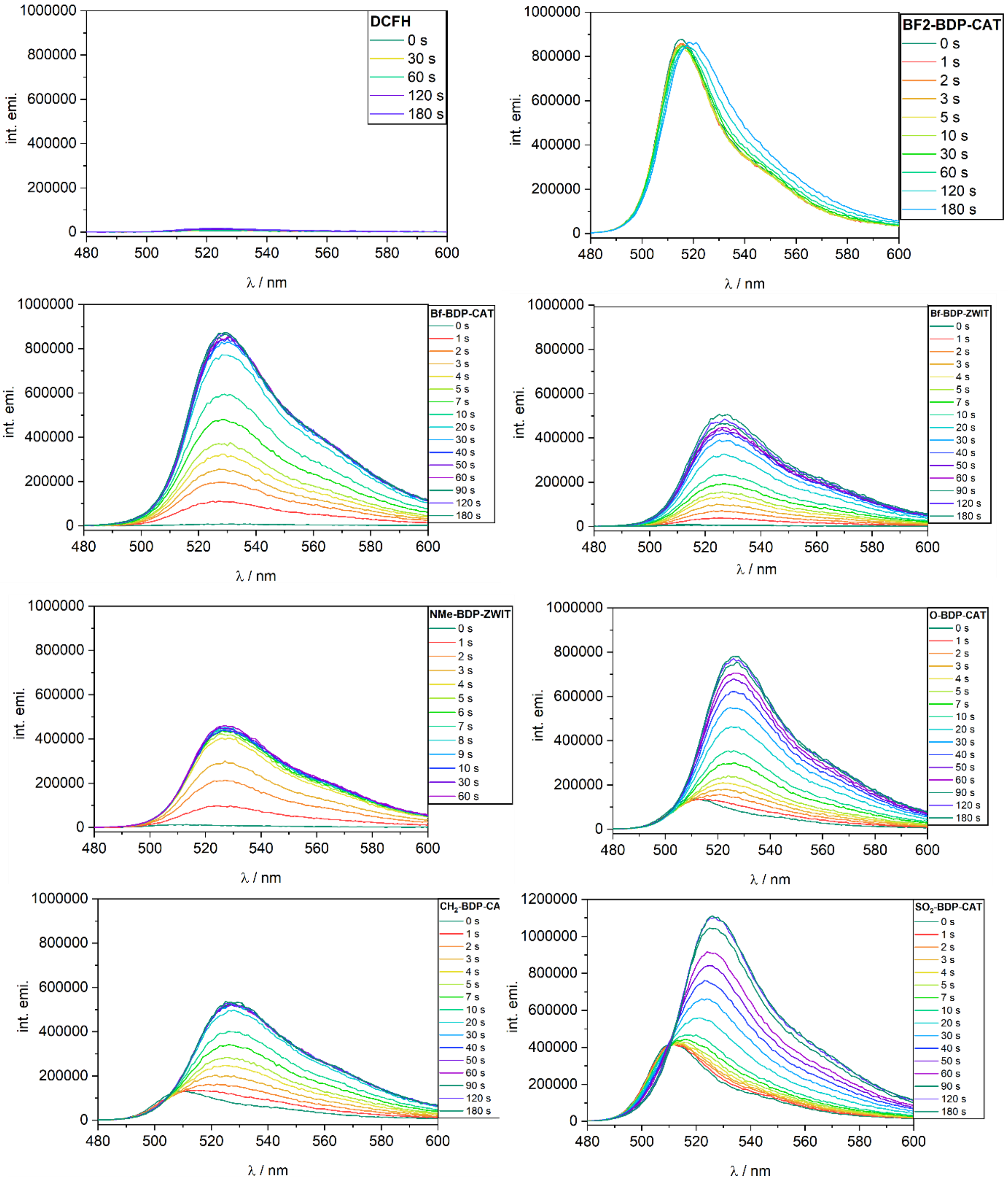

Figure S51. Results of the irradiation studies (40 mW·cm^{-2}) with photosensitizer (c = 10^{-5} M) and DCFH (c = 5·10^{-5} M) in DMSO/water (2% v/v).

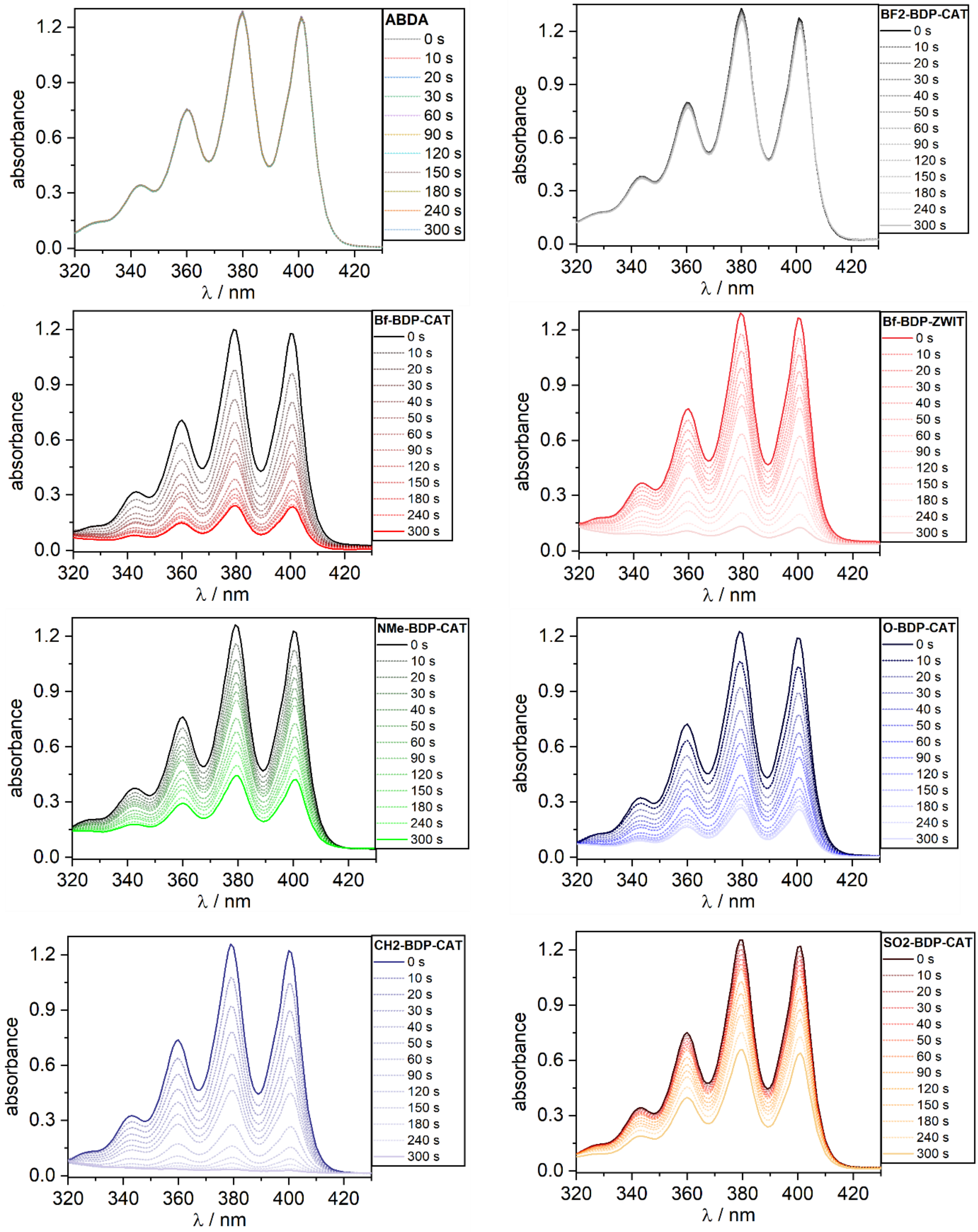

Figure S52. Results of the irradiation studies (40 mW·cm^{-2}) with photosensitizer ($c = 10^{-5}$ M) and ABDA ($c = 10^{-4}$ M) in PBS.

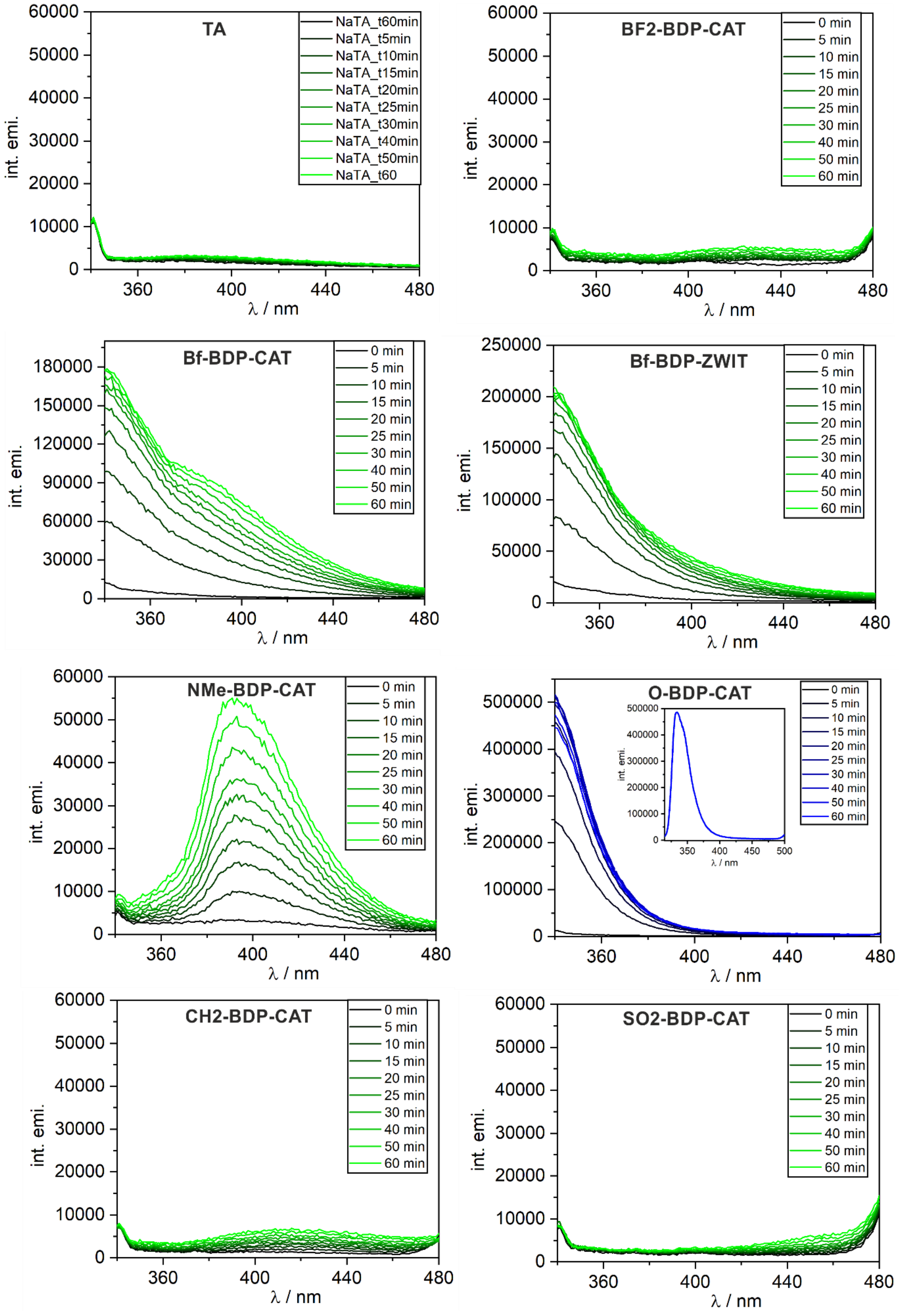

Figure S53. Results of the irradiation studies (40 mW·cm^{-2}) with photosensitizer (c = 2·10^{-5} M) and NaTA (c = 5·10^{-4} M) in PBS.

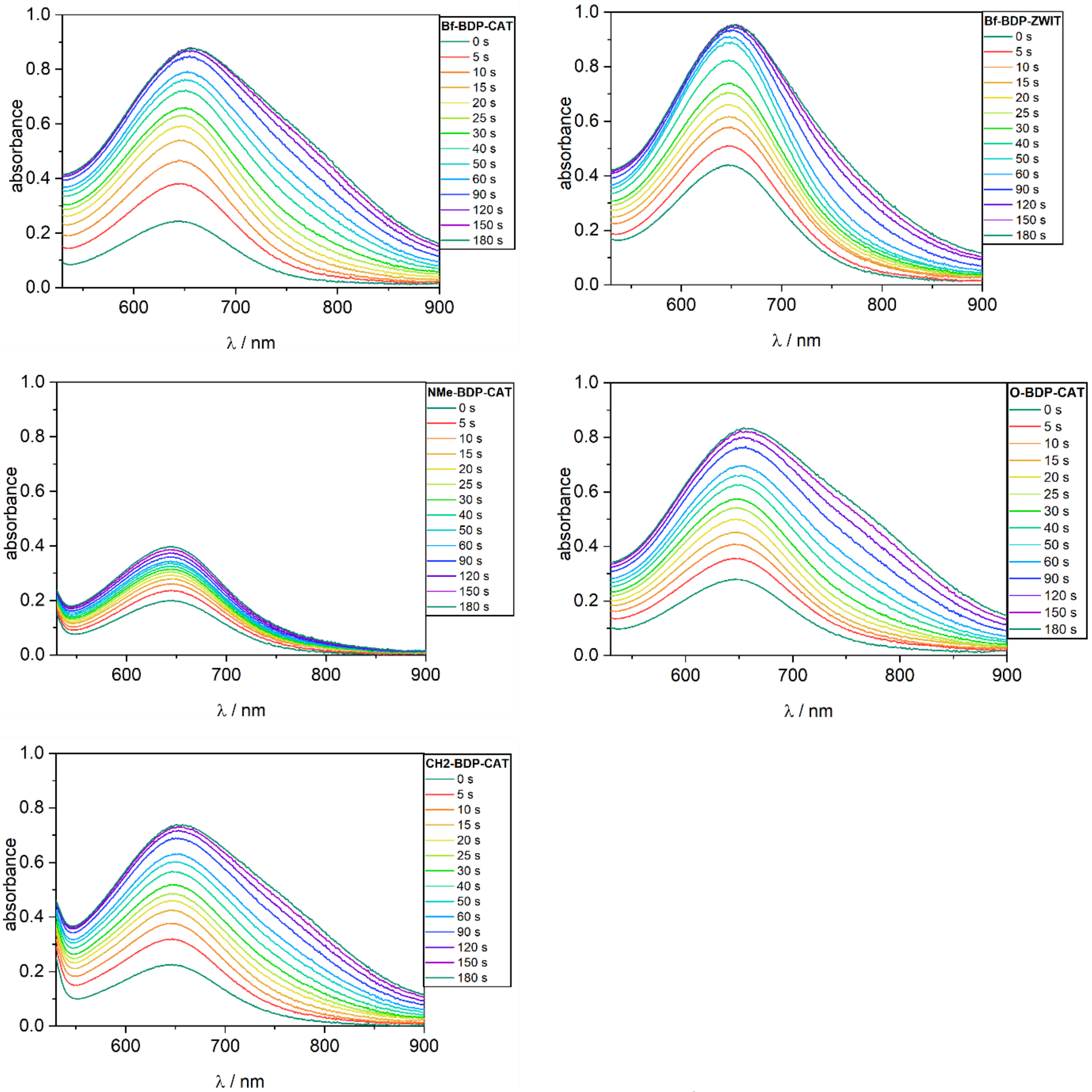

Figure S54. Results of the irradiation studies (40 mW·cm^{-2}) with photosensitizer (c = 2·10^{-5} M) and NBT (c = 5·10^{-5} M) in water/DMSO mixture (2:1 v/v).

7. Theoretical calculations

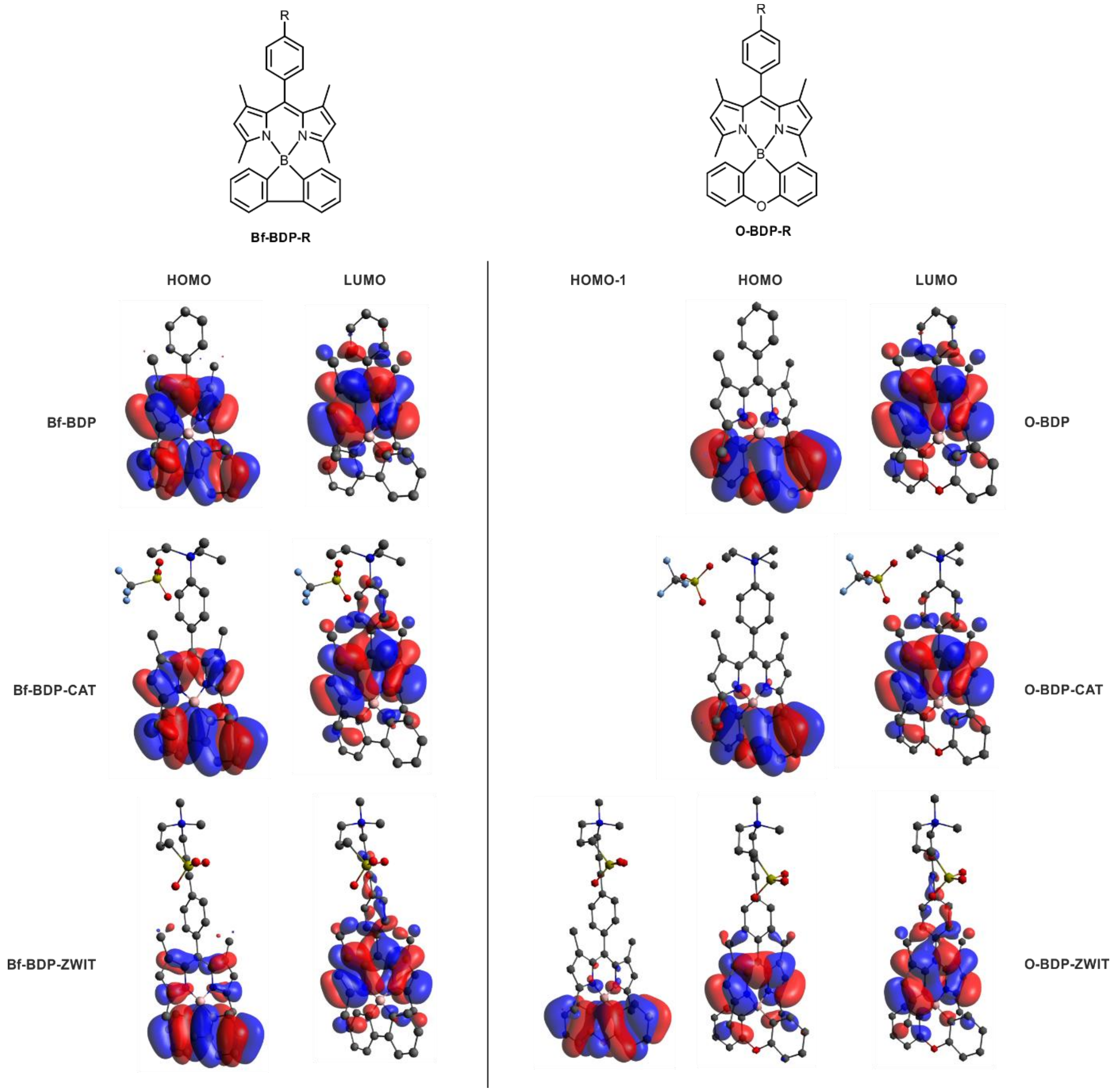

Figure S55. Frontier molecular orbitals in **Bf-BDP-R** and **O-BDP-R**.

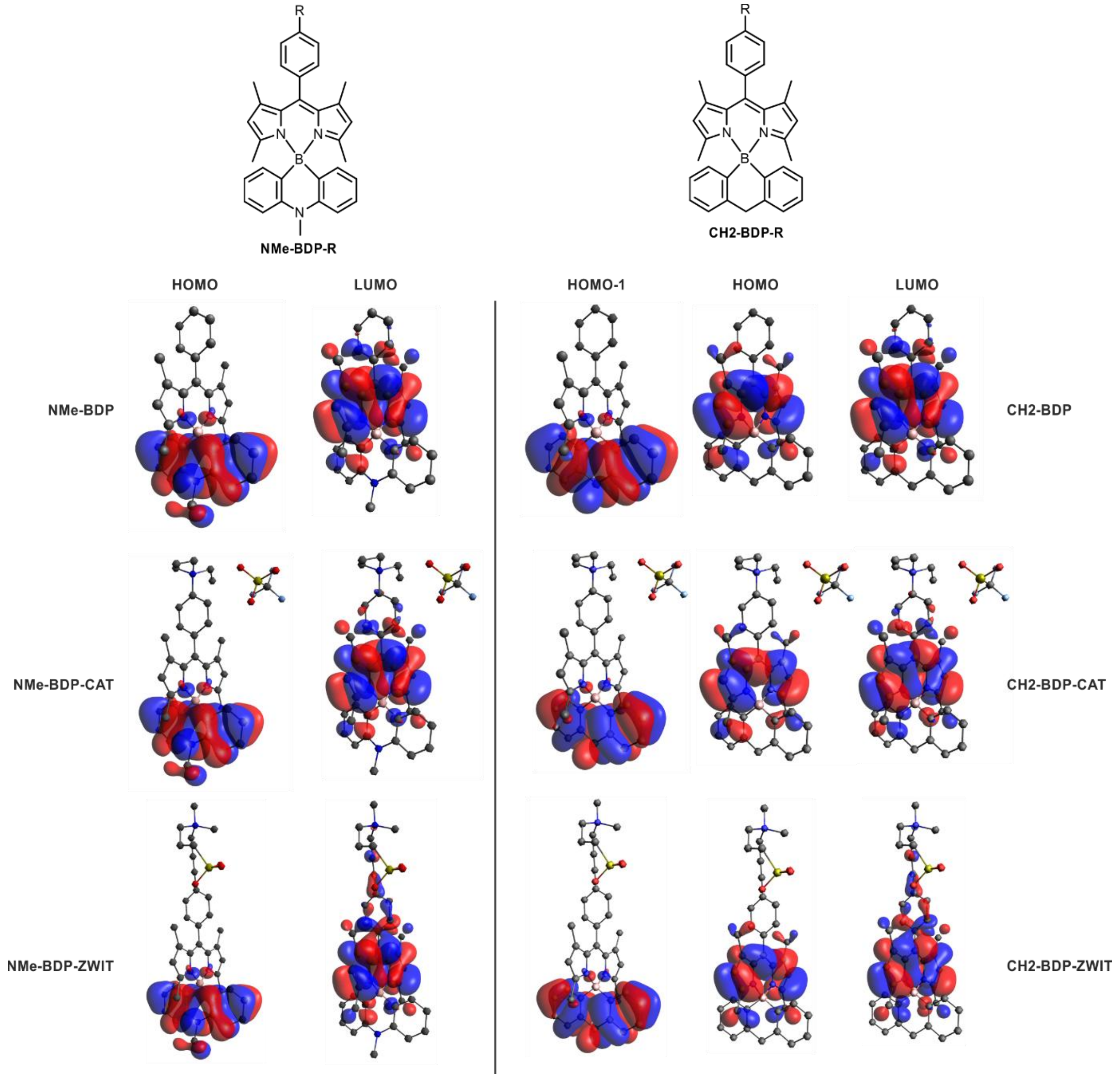

Figure S56. Frontier molecular orbitals in **NMe-BDP-R** and **CH2-BDP-R**.

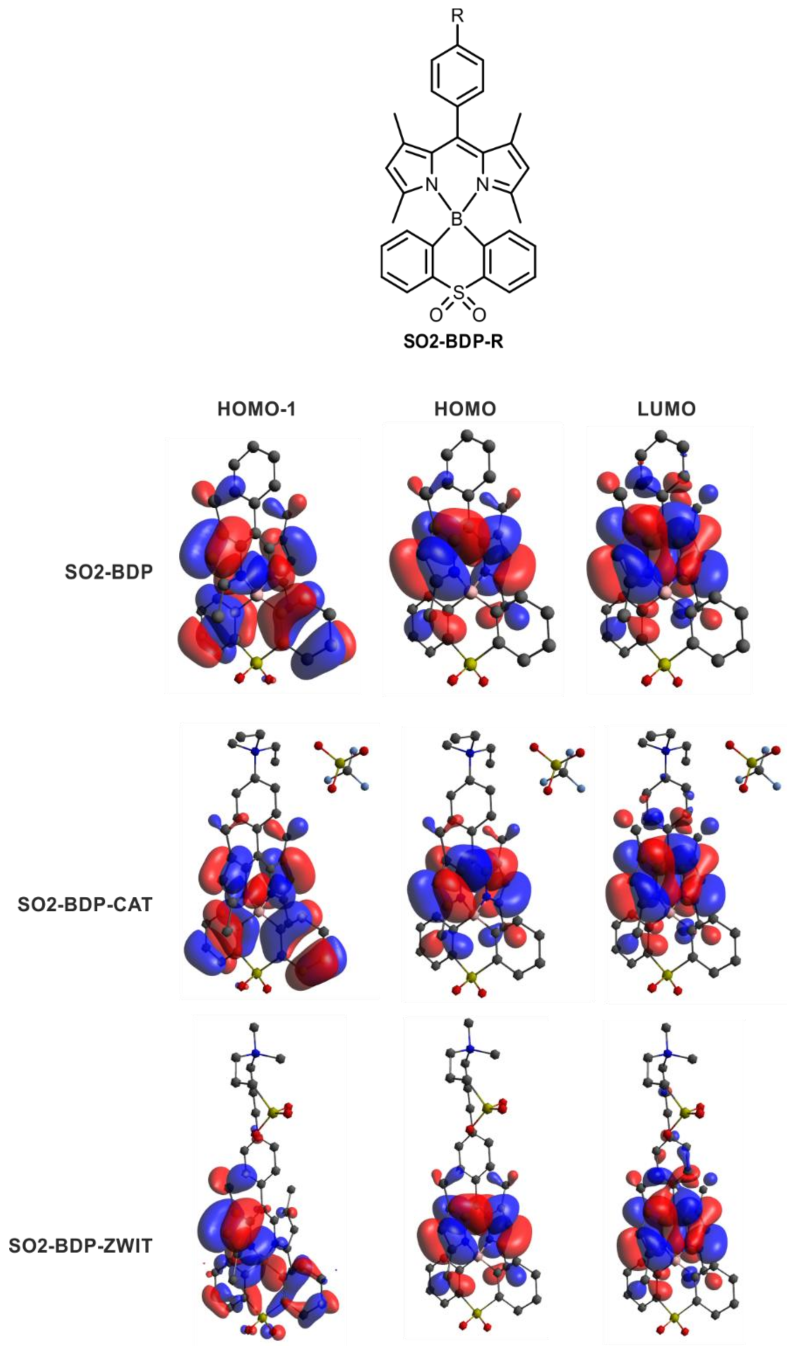

Figure S57. Frontier molecular orbitals in **SO2-BDP-R**.

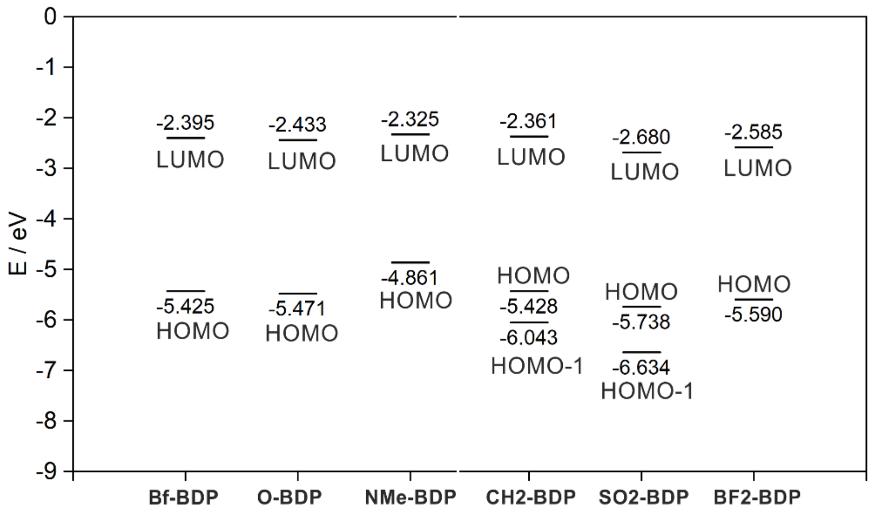

Figure S58. Molecular orbital diagram in **X-BDP.**

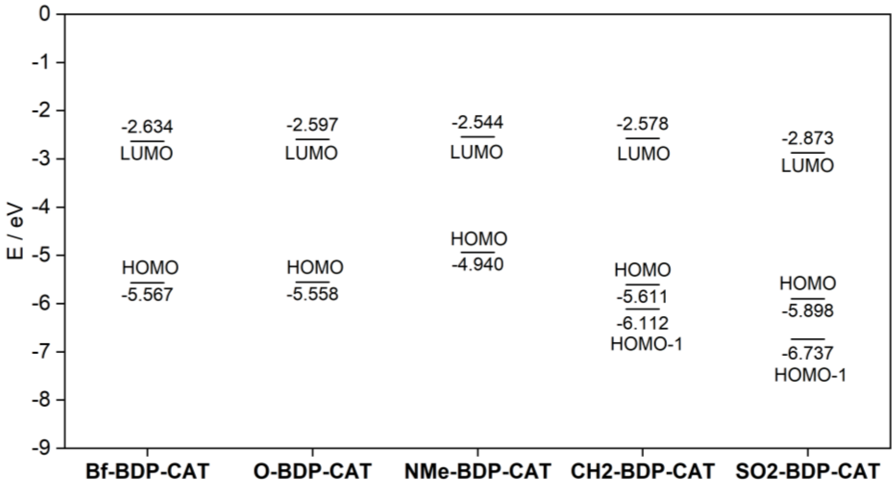

Figure S59. Molecular orbital diagram in **X-BDP-CAT.**

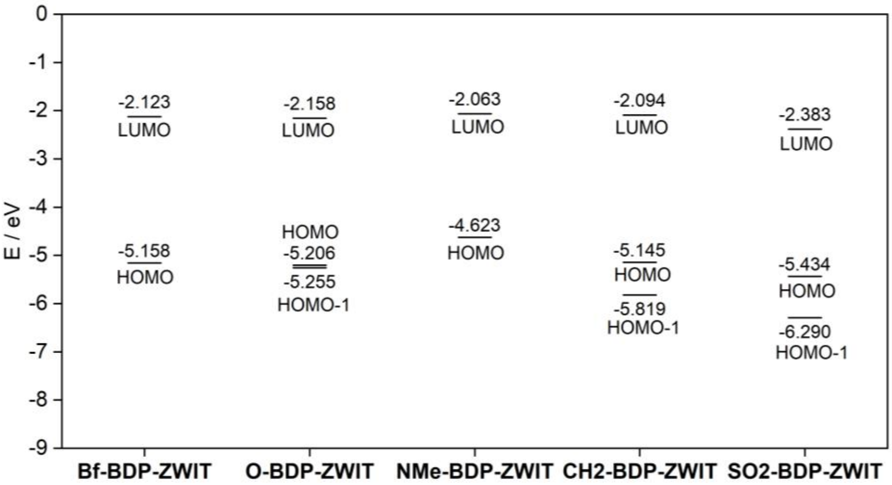

Figure S60. Molecular orbital diagram in **X-BDP-ZWIT.**

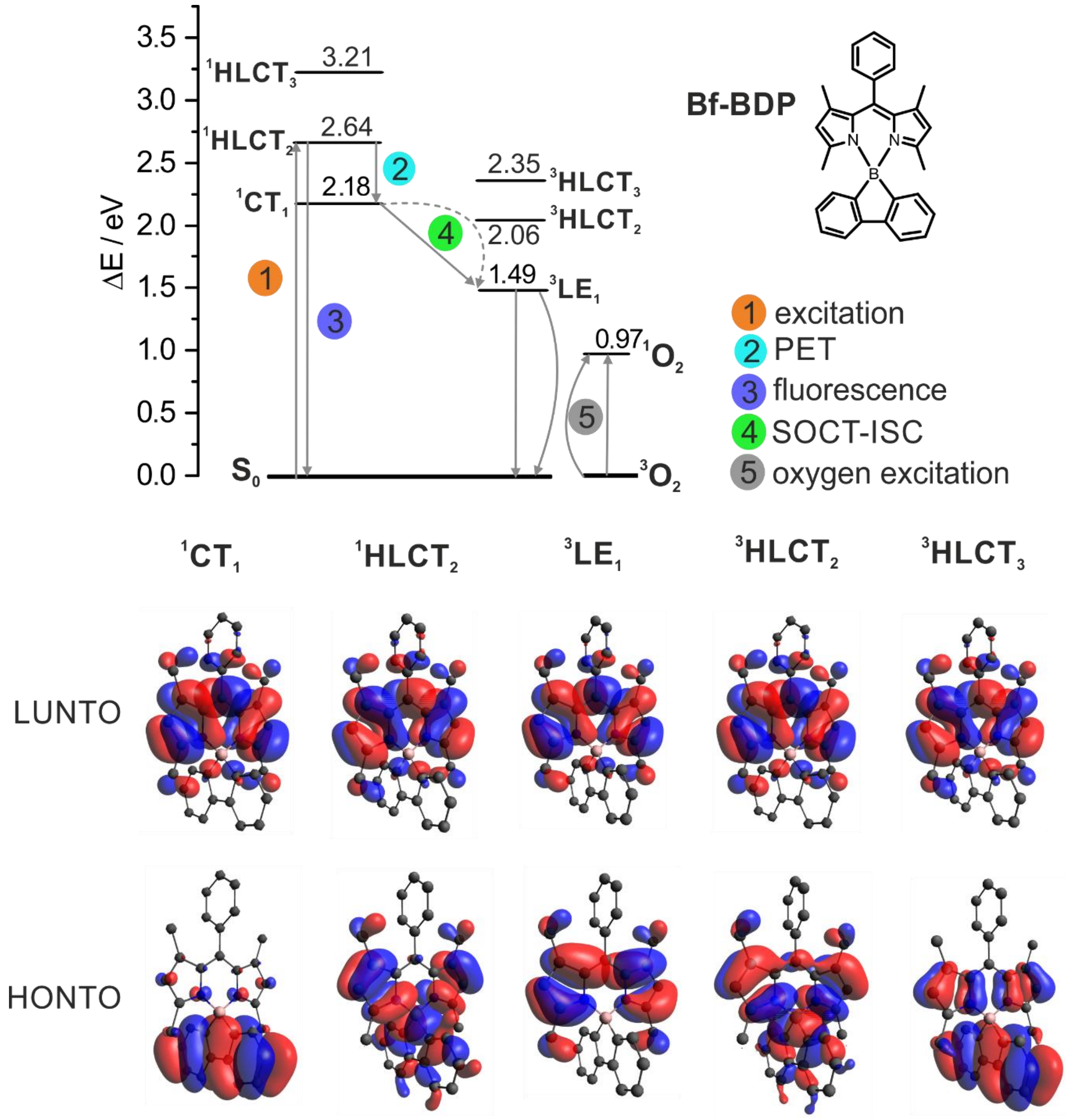

Figure S61. Energy diagram along with NTO orbitals demonstrating the photophysical processes in **Bf-BDP**.

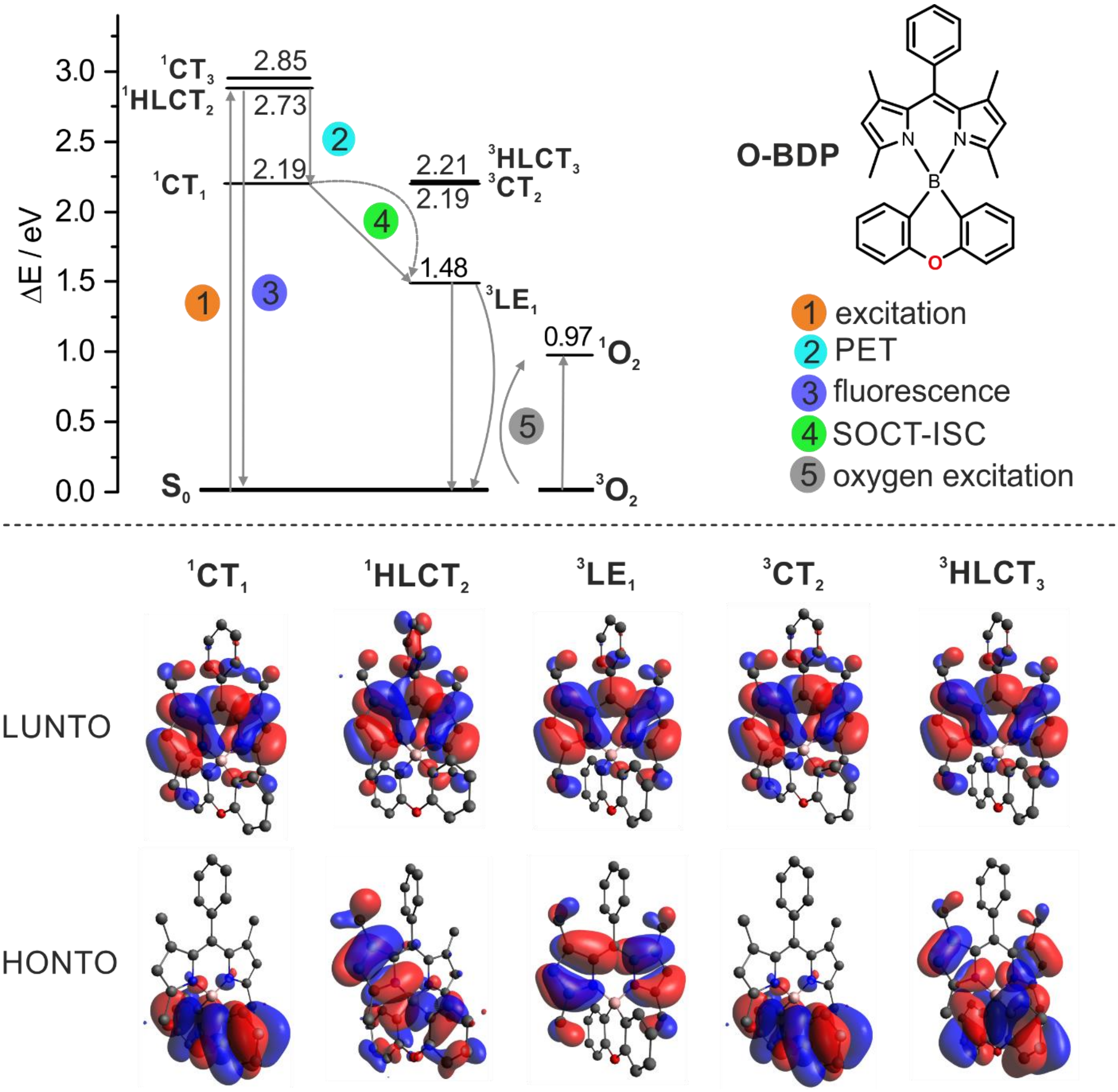

Figure S62. Energy diagram along with NTO orbitals demonstrating the photophysical processes in **O-BDP**.

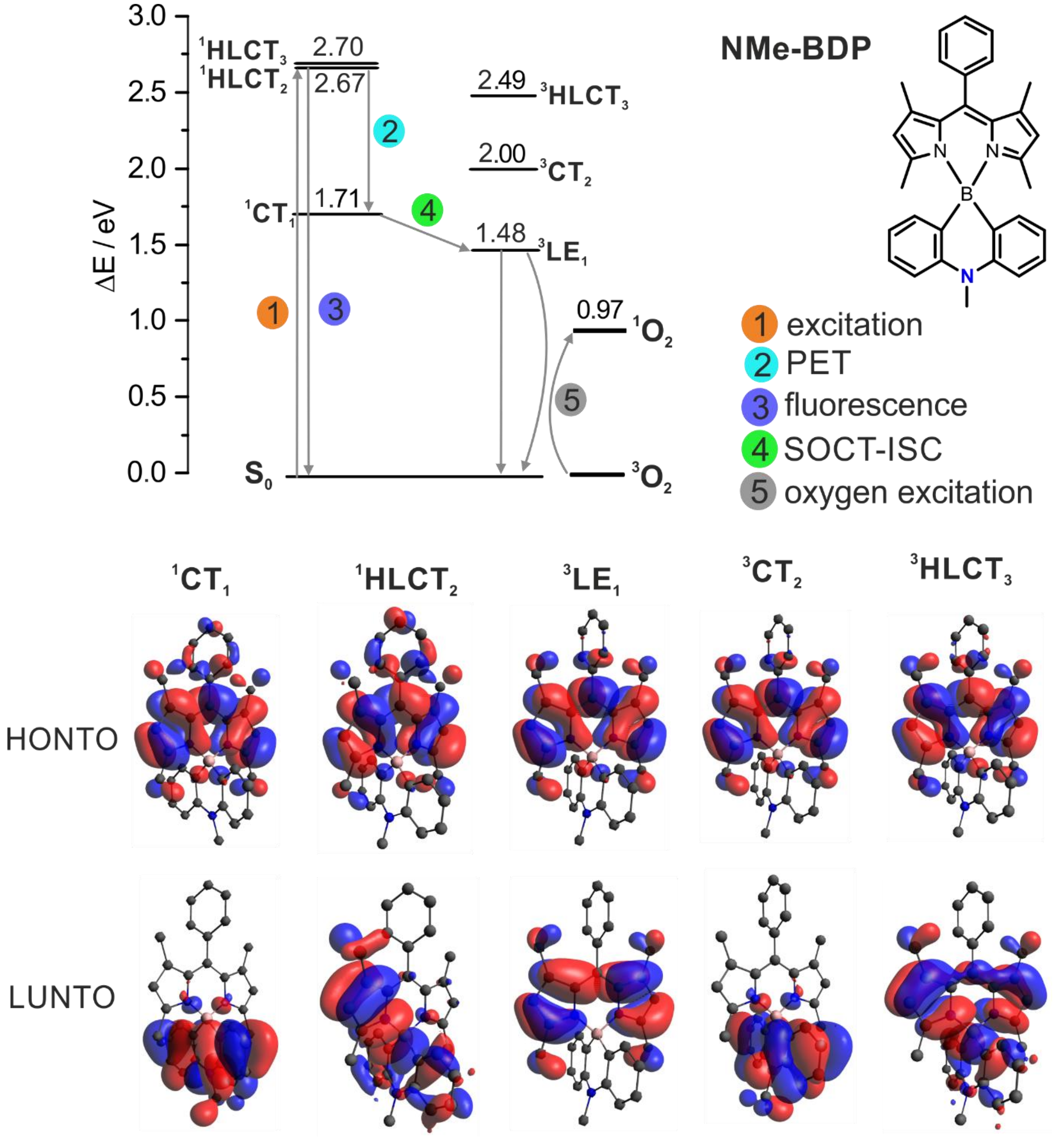

Figure S63. Energy diagram along with NTO orbitals demonstrating the photophysical processes in **NMe-BDP**.

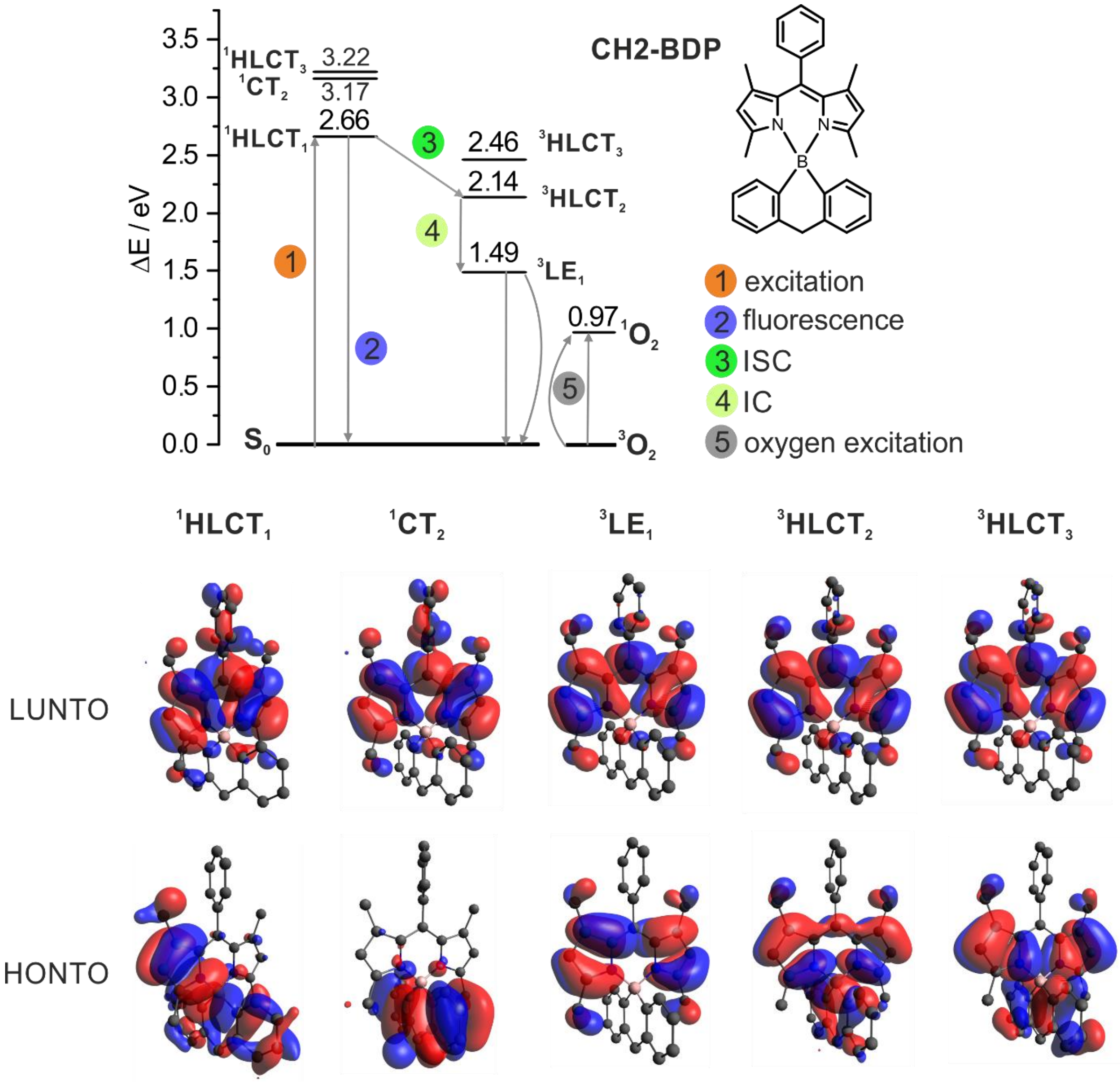

Figure S64. Energy diagram along with NTO orbitals demonstrating the photophysical processes in **CH2-BDP**.

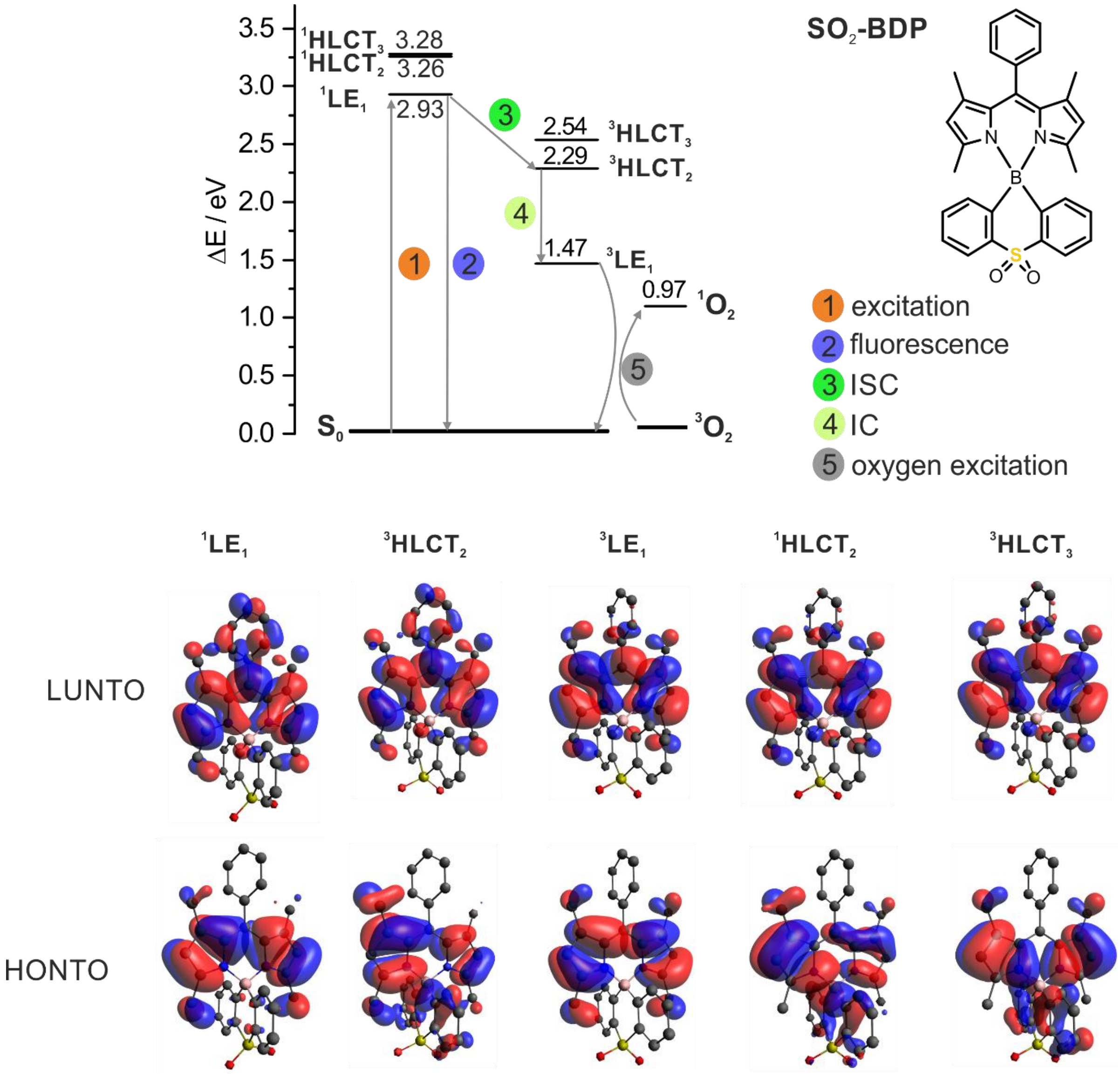

Figure S65. Energy diagram along with NTO orbitals demonstrating the photophysical processes in **SO2-BDP**.

Table S2. Hirshfeld atomic charges (with hydrogens summed into heavy atoms) in **Bf-BDP**.

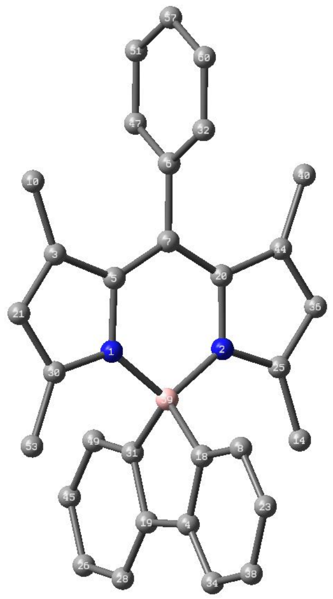

		Q / e			Q / e
1	N	-0.044	28	C	-0.015
2	N	-0.044	30	C	0.074
3	C	-0.004	31	C	-0.069
4	C	-0.006	32	C	0.018
5	C	0.007	34	C	-0.015
6	C	0.016	36	C	-0.046
7	C	0.031	38	C	-0.014
8	C	-0.014	40	C	0.014
10	C	0.013	44	C	-0.004
14	C	0.042	45	C	-0.018
18	C	-0.069	47	C	0.018
19	C	-0.006	49	C	-0.014
20	C	0.007	51	C	0.012
21	C	-0.046	53	C	0.042
23	C	-0.018	57	C	0.008
25	C	0.074	59	B	0.073
26	C	-0.014	60	C	0.012

Table S3. Hirshfeld atomic charges (with hydrogens summed into heavy atoms) in **NMe-BDP**.

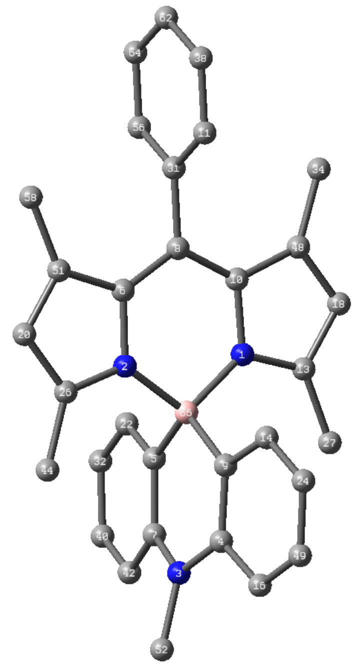

		Q / e			Q / e
1	N	-0.042	26	C	0.074
2	N	-0.044	27	C	0.038
3	N	-0.031	31	C	0.016
4	C	0.042	32	C	-0.033
5	C	-0.086	34	C	0.012
6	C	0.007	38	C	0.011
7	C	0.042	40	C	-0.015
8	C	0.031	42	C	-0.040
9	C	-0.085	44	C	0.036
10	C	0.006	48	C	-0.004
11	C	0.018	49	C	-0.015
13	C	0.075	51	C	-0.004
14	C	-0.015	52	C	0.072
16	C	-0.040	56	C	0.017
18	C	-0.047	58	C	0.012
20	C	-0.047	62	C	0.007
22	C	-0.015	64	C	0.011
24	C	-0.033	66	B	0.071
1	N	-0.042	26	C	0.074

Table S4. Hirshfeld atomic charges (with hydrogens summed into heavy atoms) in **O-BDP**.

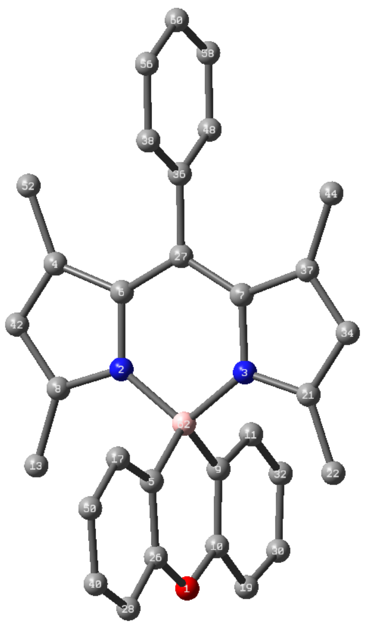

		Q / e			Q / e
1	O	-0.113	28	C	-0.020
2	N	-0.044	30	C	-0.008
3	N	-0.044	32	C	-0.026
4	C	-0.003	34	C	-0.045
5	C	-0.087	36	C	0.016
6	C	0.006	37	C	-0.003
7	C	0.006	38	C	0.017
8	C	0.075	40	C	-0.008
9	C	-0.087	42	C	-0.045
10	C	0.070	44	C	0.014
11	C	-0.011	48	C	0.017
13	C	0.042	50	C	-0.026
17	C	-0.011	52	C	0.014
19	C	-0.020	56	C	0.012
21	C	0.075	58	C	0.012
22	C	0.042	60	C	0.008
26	C	0.070	62	B	0.073
27	C	0.031			

Table S5. Hirshfeld atomic charges (with hydrogens summed into heavy atoms) in **CH2-BDP**.

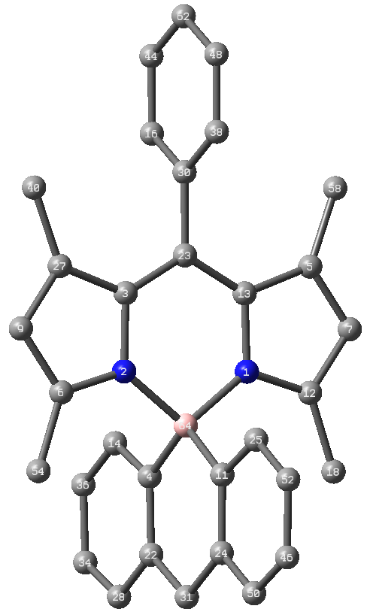

		Q / e			Q / e
1	N	-0.042	27	C	-0.004
2	N	-0.042	28	C	-0.021
3	C	0.007	30	C	0.016
4	C	-0.072	31	C	0.019
5	C	-0.004	34	C	-0.015
6	C	0.074	36	C	-0.020
7	C	-0.046	38	C	0.017
9	C	-0.046	40	C	0.013
11	C	-0.072	44	C	0.011
12	C	0.074	46	C	-0.015
13	C	0.007	48	C	0.011
14	C	-0.018	50	C	-0.021
16	C	0.017	52	C	-0.020
18	C	0.041	54	C	0.041
22	C	0.002	58	C	0.013
23	C	0.031	62	C	0.008
24	C	0.002	64	B	0.074
25	C	-0.018			

Table S6. Hirshfeld atomic charges (with hydrogens summed into heavy atoms) in **SO2-BDP**.

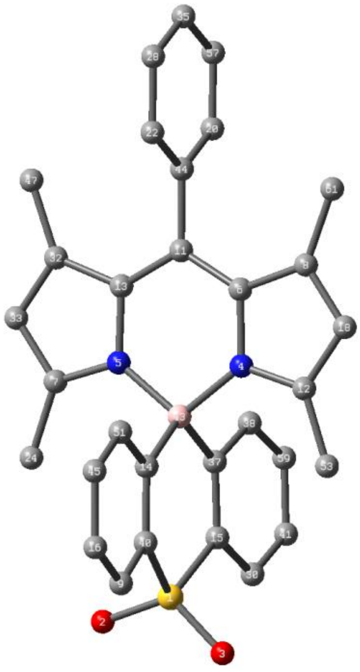

		Q / e			Q / e
1	S	0.485	28	C	0.014
2	O	-0.338	30	C	0.019
3	O	-0.343	32	C	0.000
4	N	-0.046	33	C	-0.040
5	N	-0.047	35	C	0.011
6	C	0.006	37	C	-0.062
7	C	0.077	38	C	-0.004
8	C	-0.001	40	C	-0.025
9	C	0.019	41	C	0.009
11	C	0.030	43	B	0.078
12	C	0.074	44	C	0.015
13	C	0.007	45	C	0.006
14	C	-0.062	47	C	0.018
15	C	-0.025	51	C	-0.004
16	C	0.009	53	C	0.045
18	C	-0.042	57	C	0.014
20	C	0.017	59	C	0.006
22	C	0.017	61	C	0.017
24	C	0.045			

8. Fluorescence microscopy images and Confocal Laser Scanning Microscopy measurements

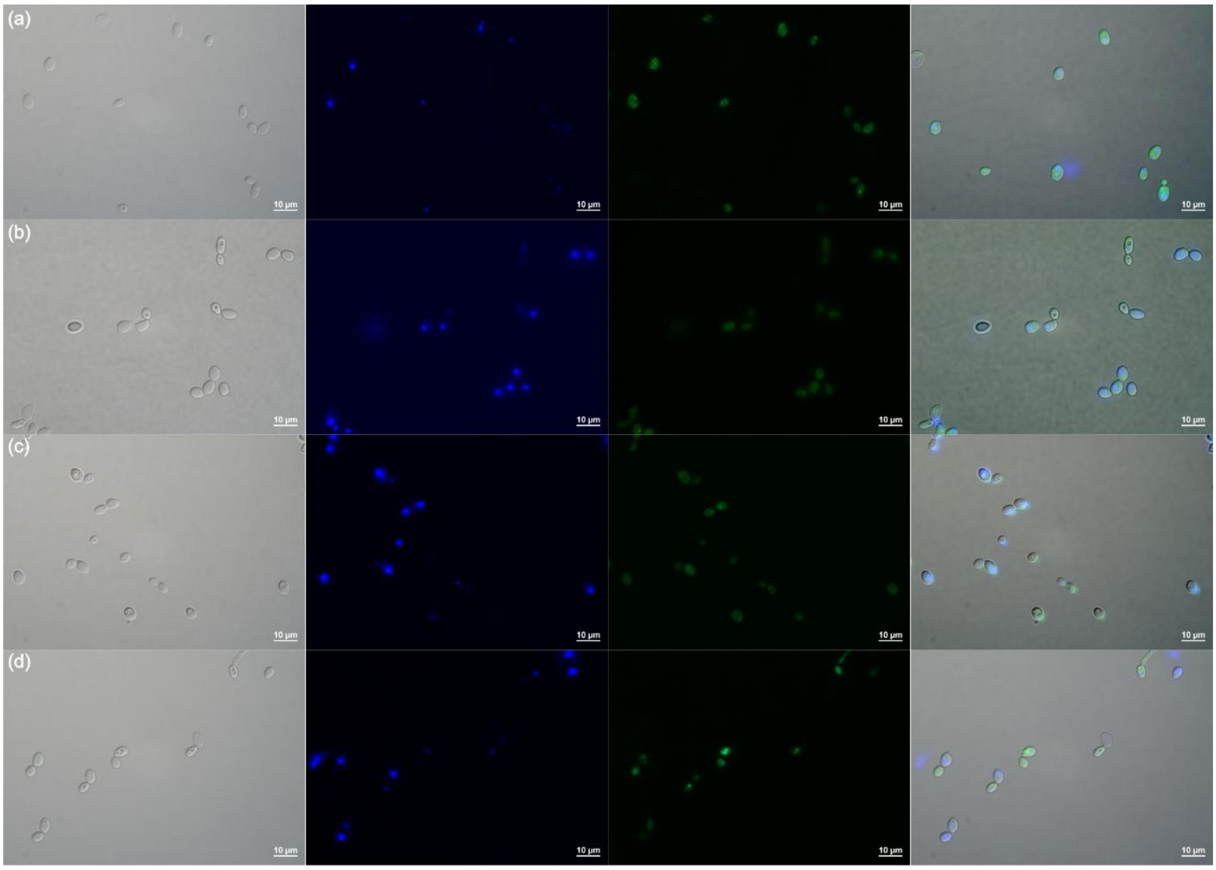

Figure S66. The fluorescence microscopy images of *C. albicans* co-stained with DAPI and BODIPY. From left: images taken in white light, DAPI fluorescence, BODIPY fluorescence and merged images: (*a*) **Bf-BDP-ZWIT** (6.3 µM), (*b*) **Bf-BDP-CAT** (1.6 µM), (*c*) **CH2-BDP-CAT** (1.6 µM), and (*d*) **BF2-BDP-CAT** (12.5 µM).

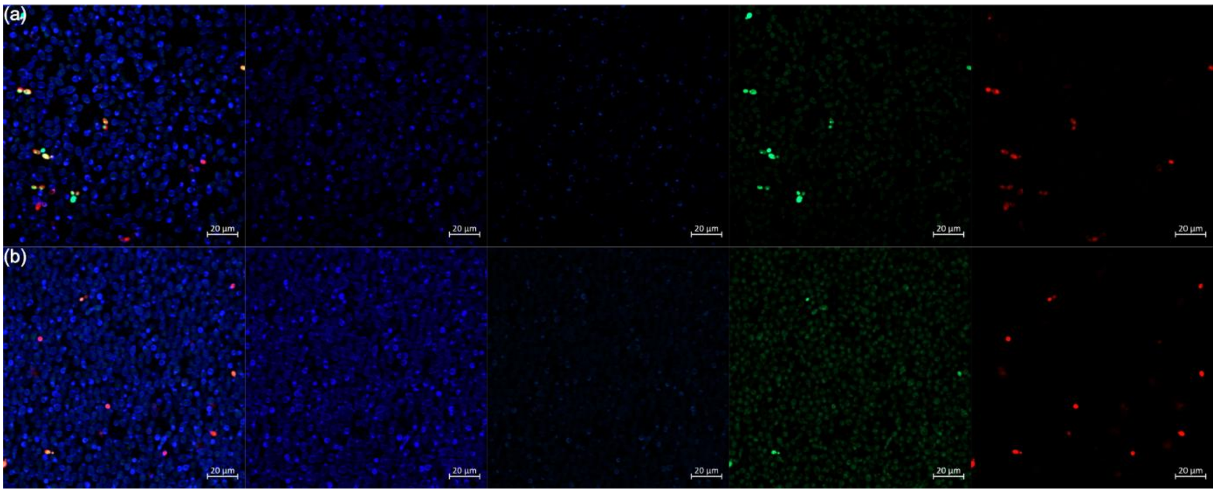

Figure S67. CLSM images of *C. albicans* cells co-stained with Calcofluor White, Hoechst 33342, **Bf-BDP-ZWIT** (3.1 µM), and propidium iodine (PI): (*a*) treated and irradiated cells, (*b*) treated cells incubated in the absence of light. From left: merged images, Calcofluor White fluorescence, Hoechst 33342 fluorescence, BODIPY fluorescence, PI fluorescence.

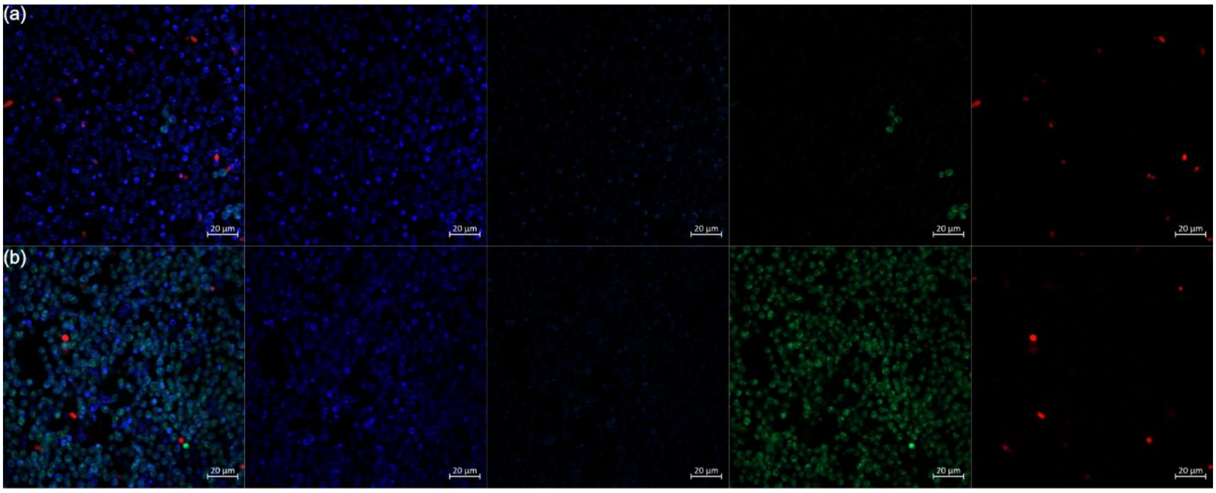

Figure S68. CLSM images of *C. albicans* cells co-stained with Calcofluor White, Hoechst 33342, **Bf-BDP-CAT** (0.8 μM), and propidium iodine (PI): (*a*) treated and irradiated cells, (*b*) treated cells incubated in the absence of light. From left: merged images, Calcofluor White fluorescence, Hoechst 33342 fluorescence, BODIPY fluorescence, PI fluorescence.

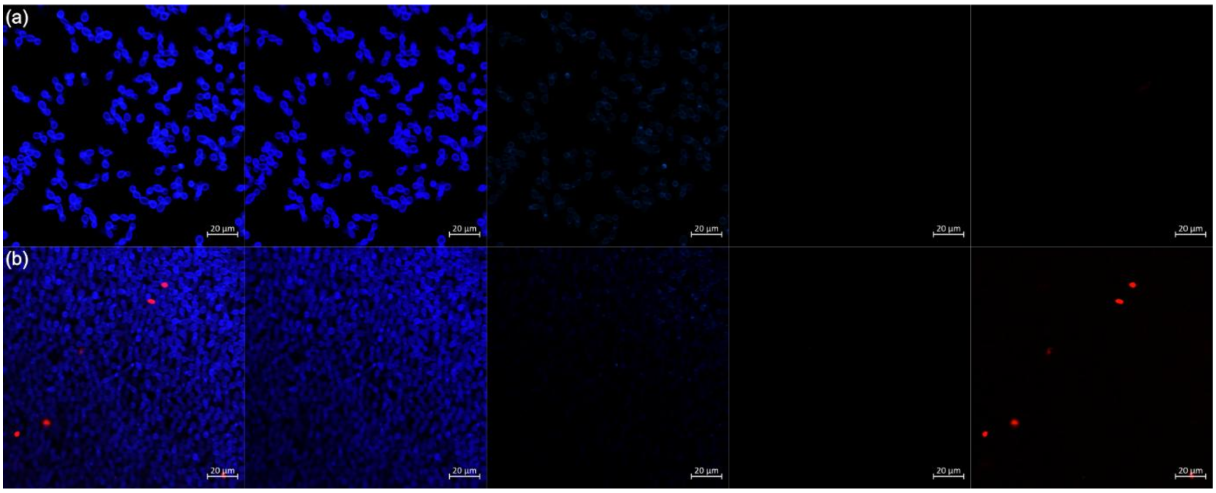

Figure S69. CLSM images of *C. albicans* cells co-stained with Calcofluor White, Hoechst 33342, **NMe-BDP-CAT** (0.8 μM), and propidium iodine (PI): (*a*) treated and irradiated cells, (*b*) treated cells incubated in the absence of light. From left: merged images, Calcofluor White fluorescence, Hoechst 33342 fluorescence, BODIPY fluorescence, PI fluorescence.

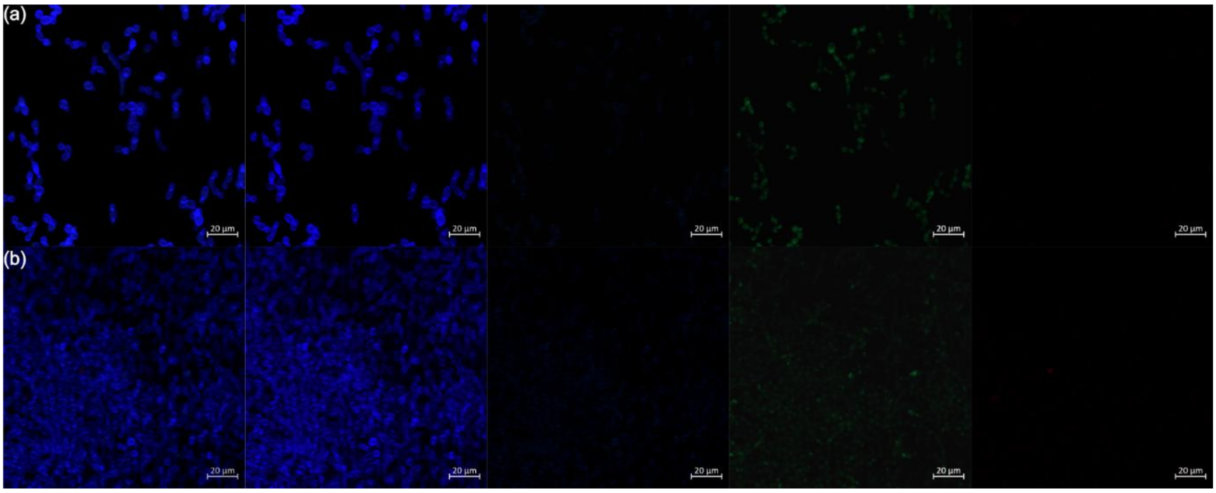

Figure S70. CLSM images of *C. albicans* cells co-stained with Calcofluor White, Hoechst 33342, **CH2-BDP-CAT** (0.8 µM), and propidium iodine (PI): (*a*) treated and irradiated cells, (*b*) treated cells incubated in the absence of light. From left: merged images, Calcofluor White fluorescence, Hoechst 33342 fluorescence, BODIPY fluorescence, PI fluorescence.

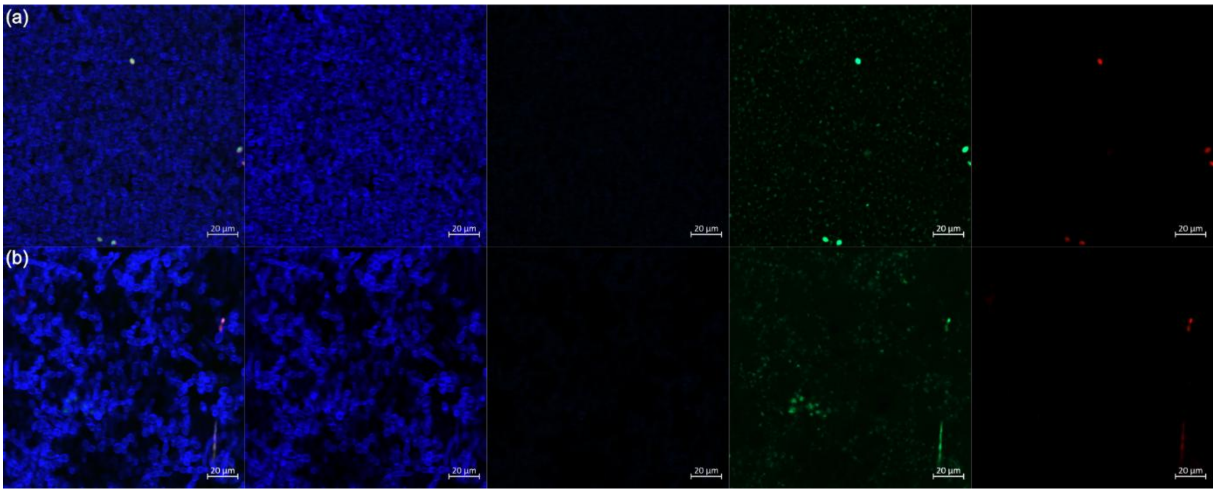

Figure S71. CLSM images of *C. albicans* cells co-stained with Calcofluor White, Hoechst 33342, **SO2-BDP-CAT** (12.5 µM), and propidium iodine (PI): (*a*) treated and irradiated cells, (*b*) treated cells incubated in the absence of light. From left: merged images, Calcofluor White fluorescence, Hoechst 33342 fluorescence, BODIPY fluorescence, PI fluorescence.

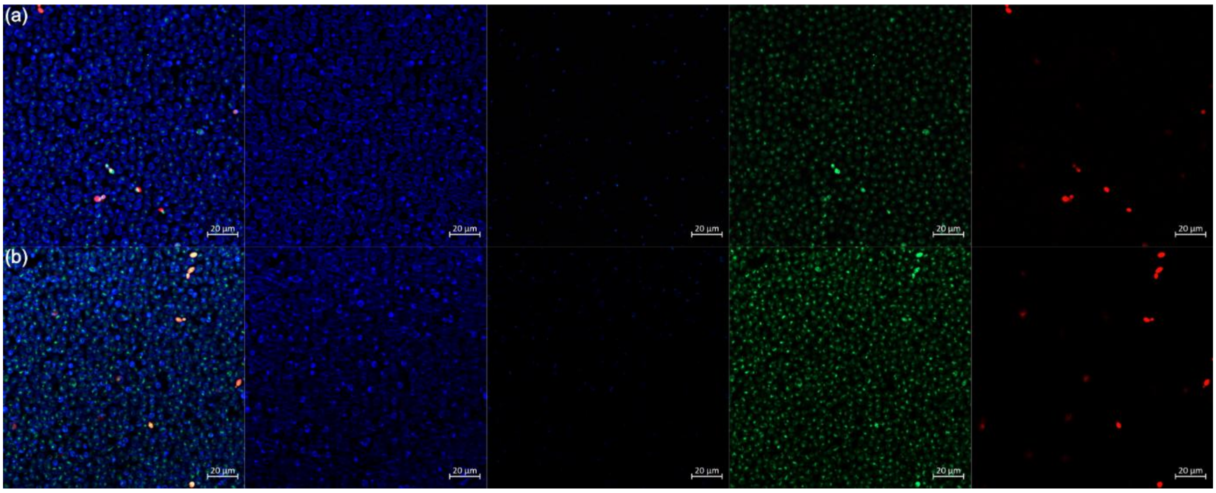

Figure S72. CLSM images of *C. albicans* cells co-stained with Calcofluor White, Hoechst 33342, **BF2-BDP-CAT** (1.6 µM), and propidium iodine (PI): (*a*) treated and irradiated cells, (*b*) treated cells incubated in the absence of light. From left: merged images, Calcofluor White fluorescence, Hoechst 33342 fluorescence, BODIPY fluorescence, PI fluorescence.

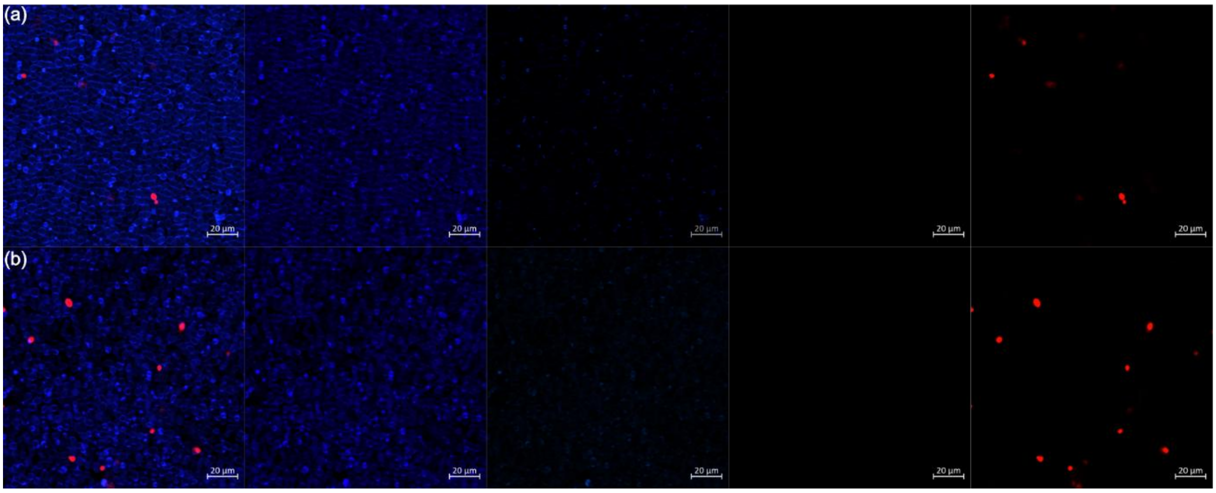

Figure S73. CLSM images of untreated *C. albicans* cells co-stained with Calcofluor White, Hoechst 33342, and propidium iodine (PI): (*a*) irradiated cells, (*b*) cells incubated in the absence of light. From left: merged images, Calcofluor White fluorescence, Hoechst 33342 fluorescence, BODIPY fluorescence, PI fluorescence.

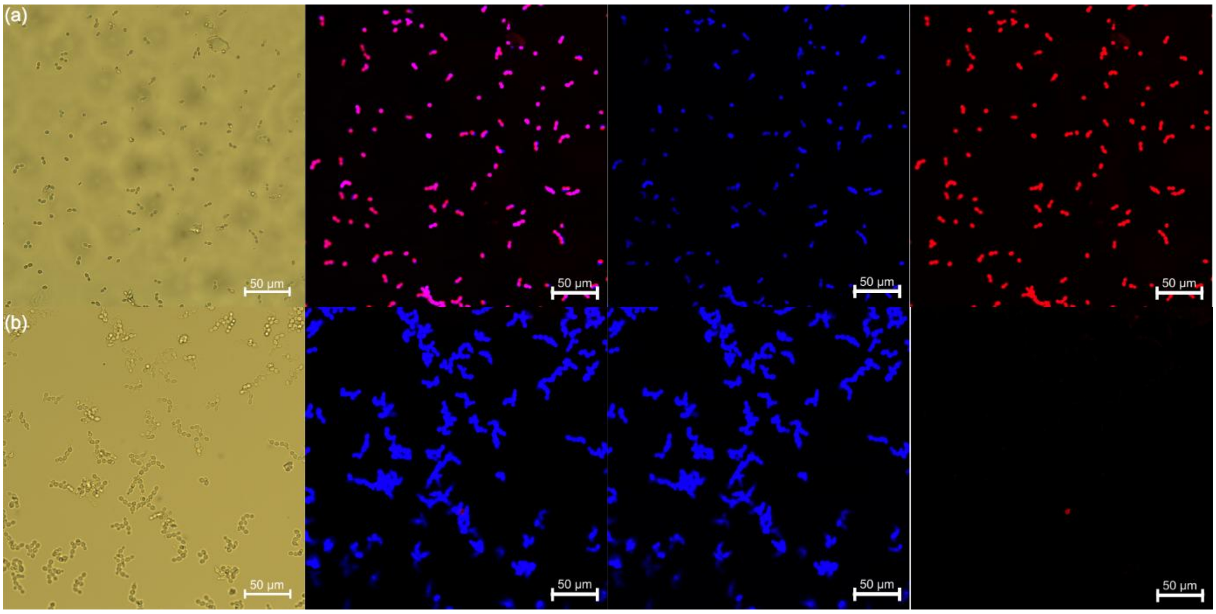

Figure S74. Results of the assay with oxidative stress indicator (CellRox™ Deep Red) after exposure to **Bf-BDP-CAT**: (*a*) treated and irradiated cells, (*b*) treated cells incubated in the absence of light. From the left: cells in the bright field, merged fluorescence images, cells stained with calcofluor white, cells stained with CellRox™ Deep Red.

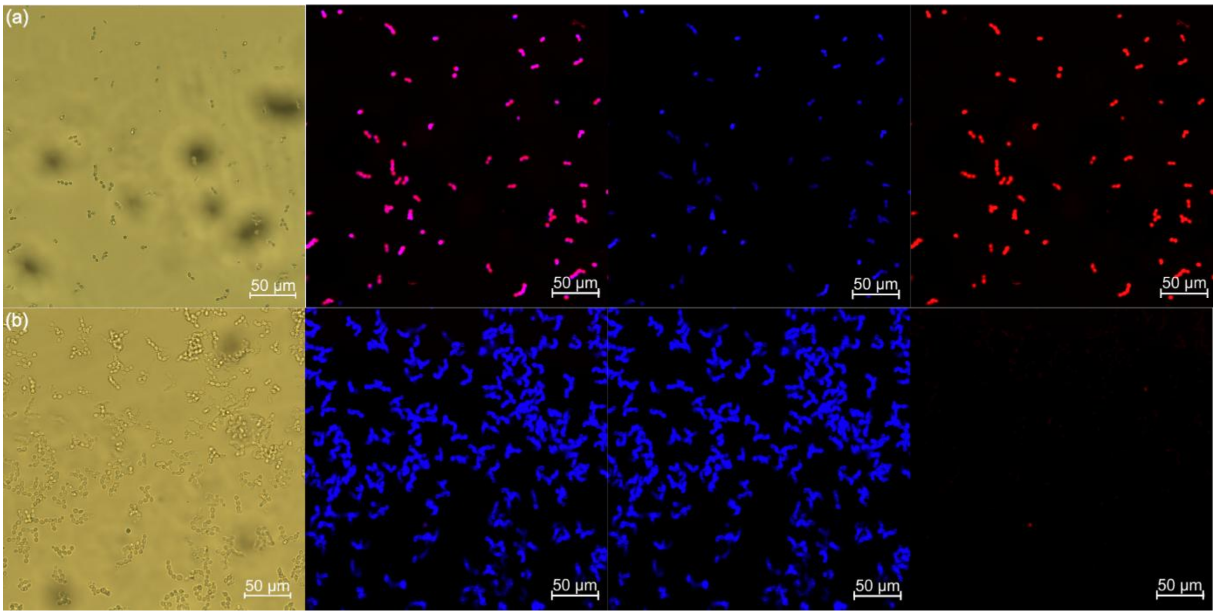

Figure S75. Results of the assay with oxidative stress indicator (CellRox™ Deep Red) after exposure to **O-BDP-CAT**: (*a*) treated and irradiated cells, (*b*) treated cells incubated in the absence of light. From the left: cells in the bright field, merged fluorescence images, cells stained with calcofluor white, cells stained with CellRox™ Deep Red.

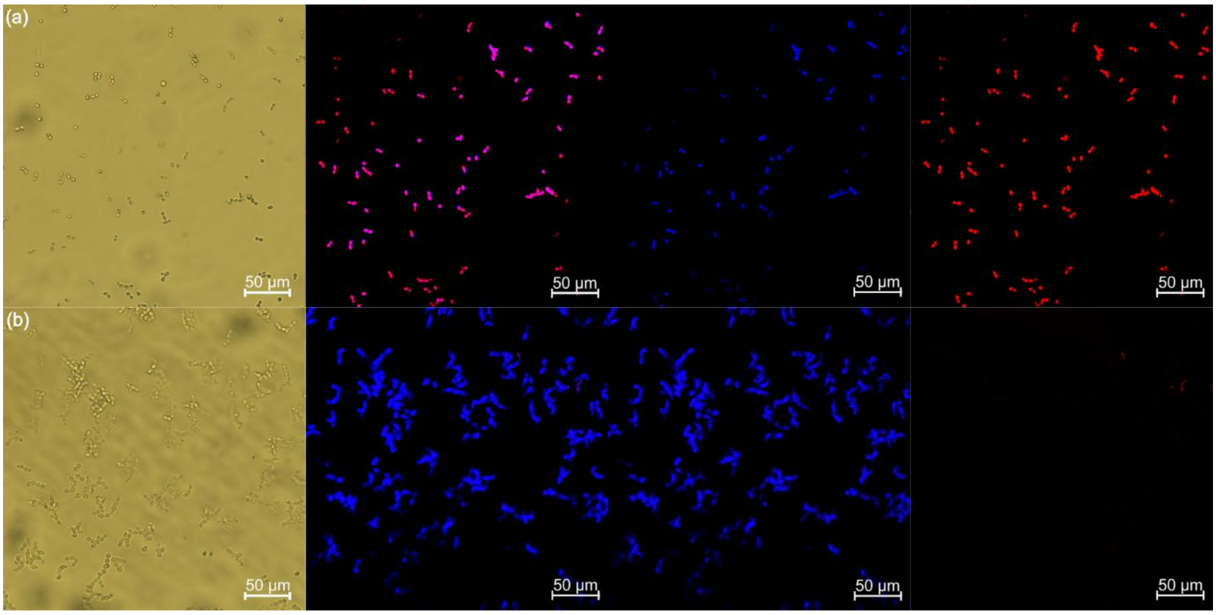

Figure S76. Results of the assay with oxidative stress indicator (CellRox™ Deep Red) after exposure to **NMe-BDP-CAT**: (*a*) treated and irradiated cells, (*b*) treated cells incubated in the absence of light. From the left: cells in the bright field, merged fluorescence images, cells stained with calcofluor white, cells stained with CellRox™ Deep Red.

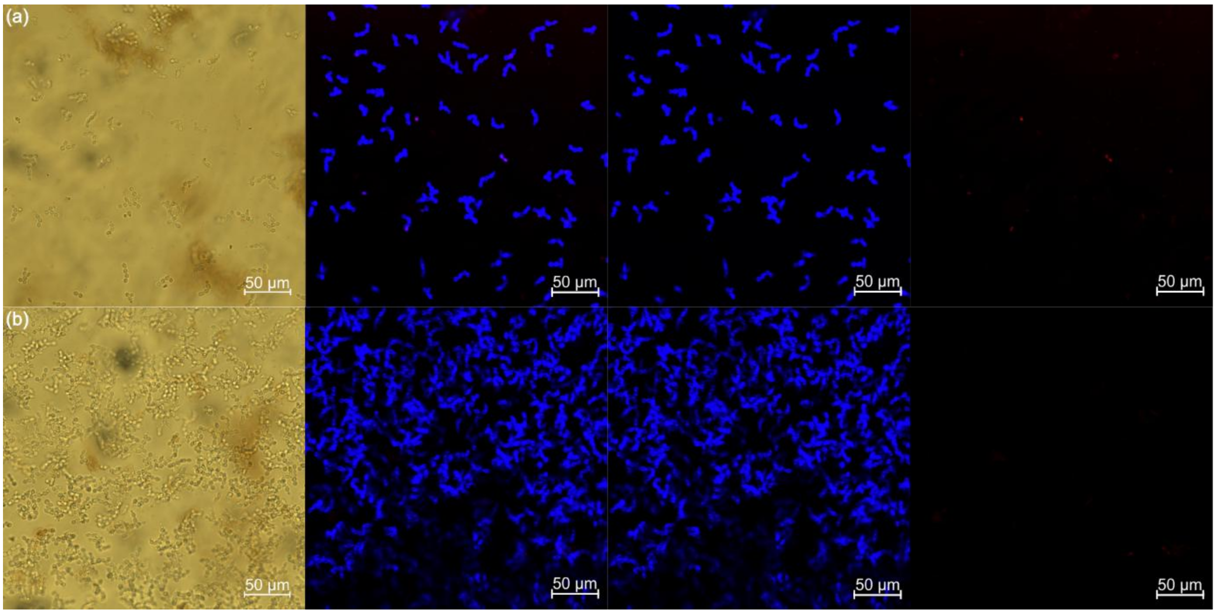

Figure S77. Results of the assay with oxidative stress indicator (CellRox™ Deep Red) after exposure to **SO_2-BDP-CAT**: (*a*) treated and irradiated cells, (*b*) treated cells incubated in the absence of light. From the left: cells in the bright field, merged fluorescence images, cells stained with calcofluor white, cells stained with CellRox™ Deep Red.

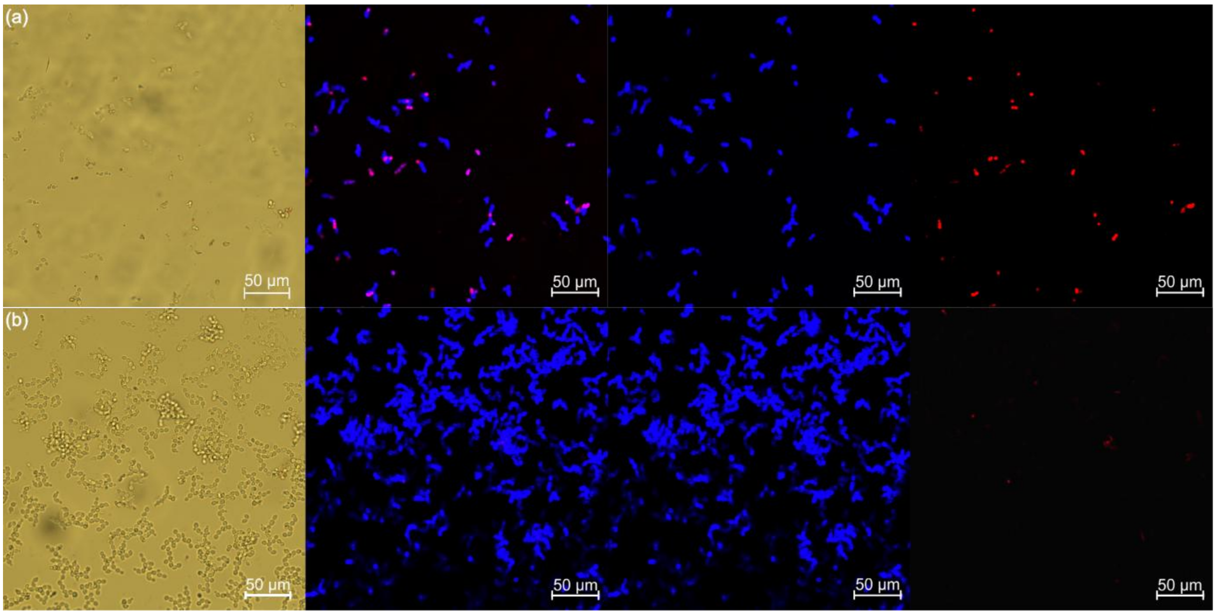

Figure S78. Results of the assay with oxidative stress indicator (CellRox™ Deep Red) after exposure to **BF_2-BDP-CAT**: (*a*) treated and irradiated cells, (*b*) treated cells incubated in the absence of light. From the left: cells in the bright field, merged fluorescence images, cells stained with calcofluor white, cells stained with CellRox™ Deep Red.

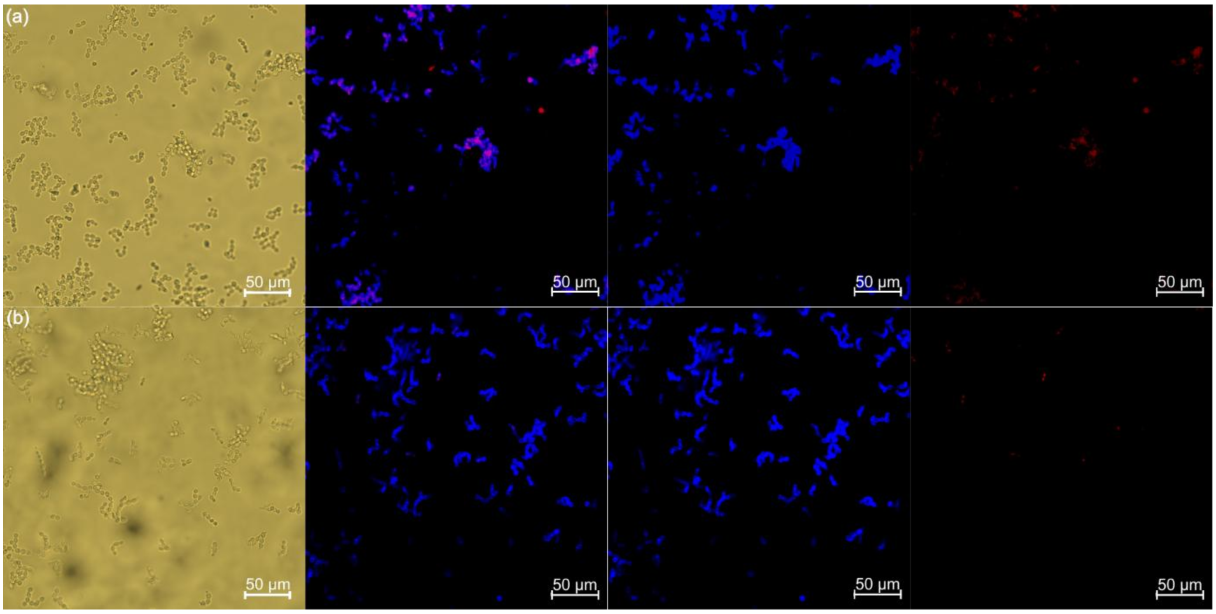

Figure S79. Results of the assay with oxidative stress indicator (CellRox™ Deep Red) for negative control (without exposure to the photosensitizer): (*a*) irradiated cells, (*b*) cells incubated in the absence of light. From the left: cells in the bright field, merged fluorescence images, cells stained with calcofluor white, cells stained with CellRox™ Deep Red.

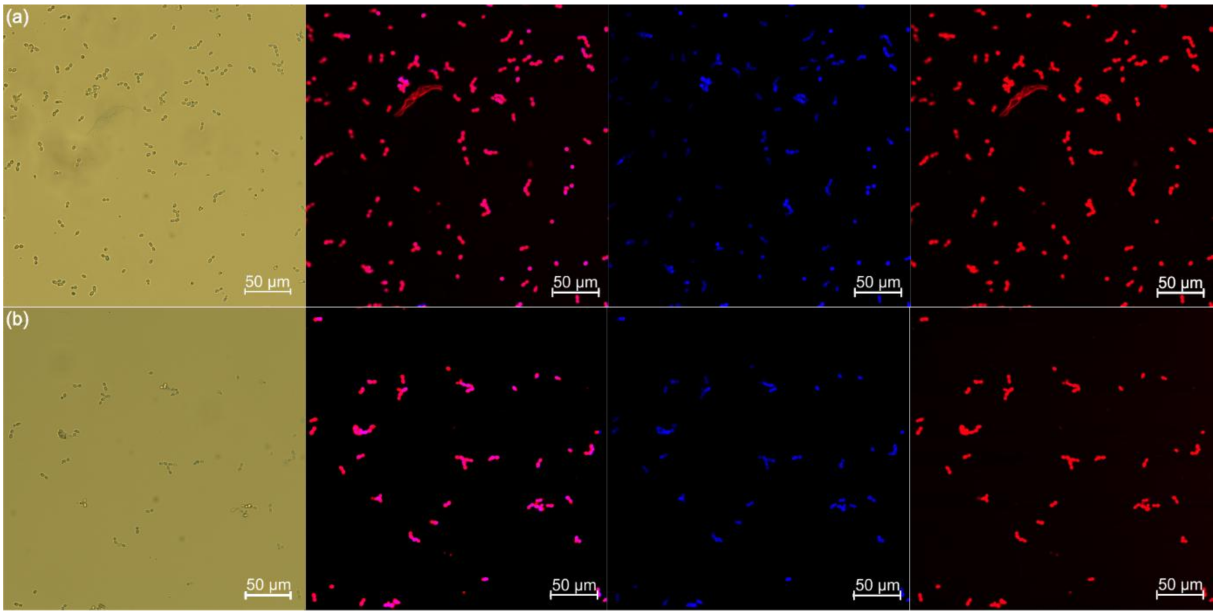

Figure S80. Results of the assay with oxidative stress indicator (CellRox™ Deep Red) after exposure to 3 % solution of H_2O_2 (positive control): (*a*) treated and irradiated cells, (*b*) treated cells incubated in the absence of light. From the left: cells in the bright field, merged fluorescence images, cells stained with calcofluor white, cells stained with CellRox™ Deep Red.

9. NMR spectra of new compounds

9.1 NMR spectra of X-BDP-NEt2

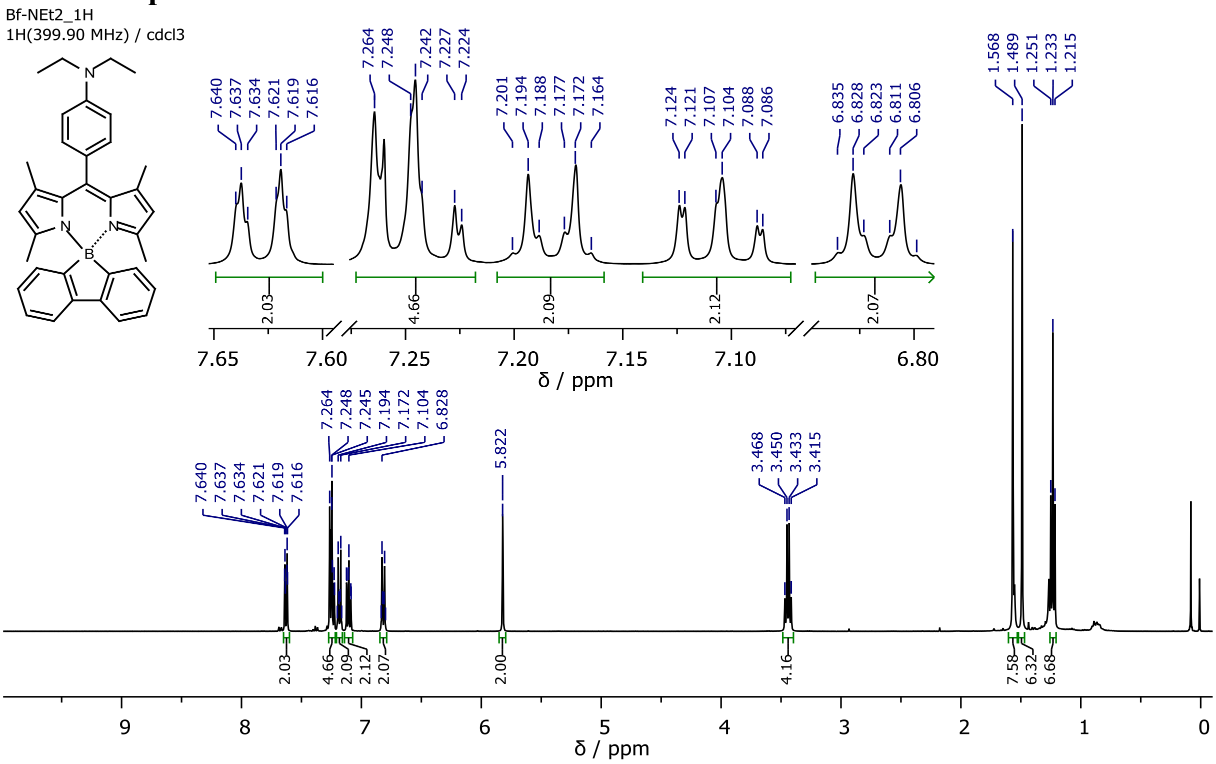

Figure S81. ^{1}H NMR spectrum of **Bf-BDP-NEt2** (400 MHz, $CDCl_3$).

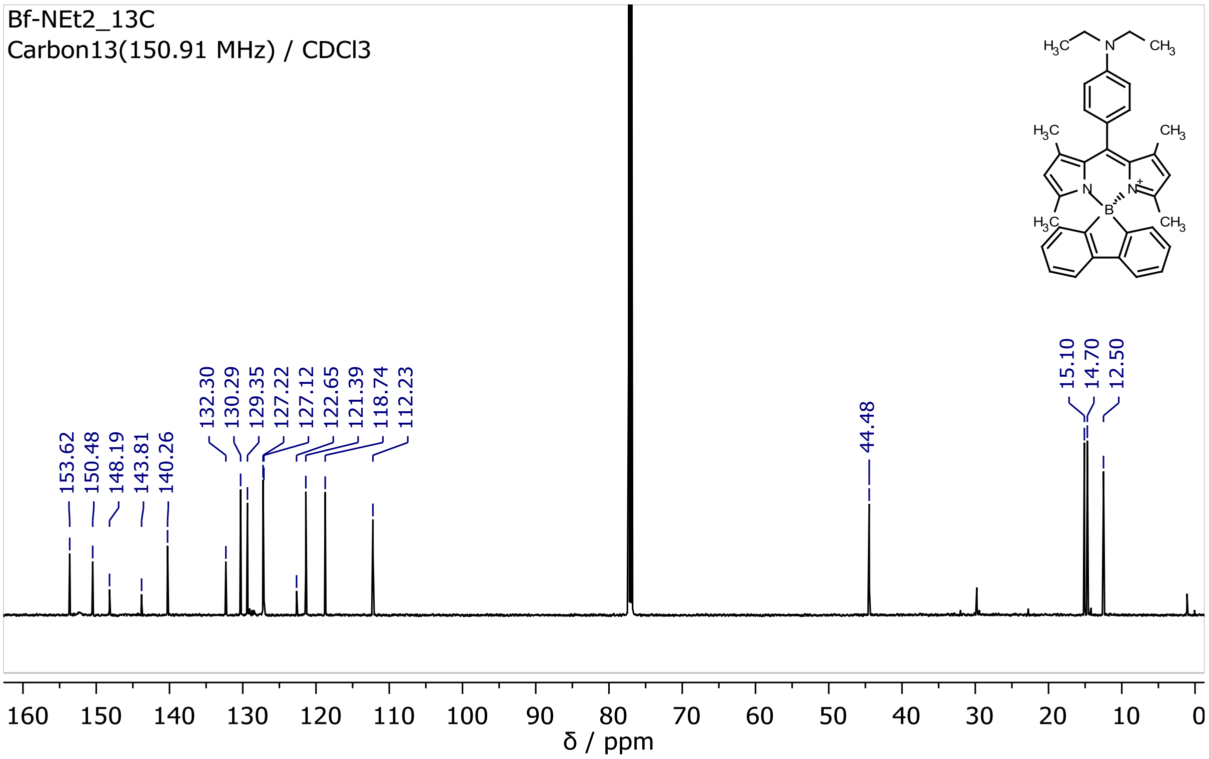

Figure S82. ^{13}C NMR spectrum of **Bf-BDP-NEt2** (151 MHz, $CDCl_3$).

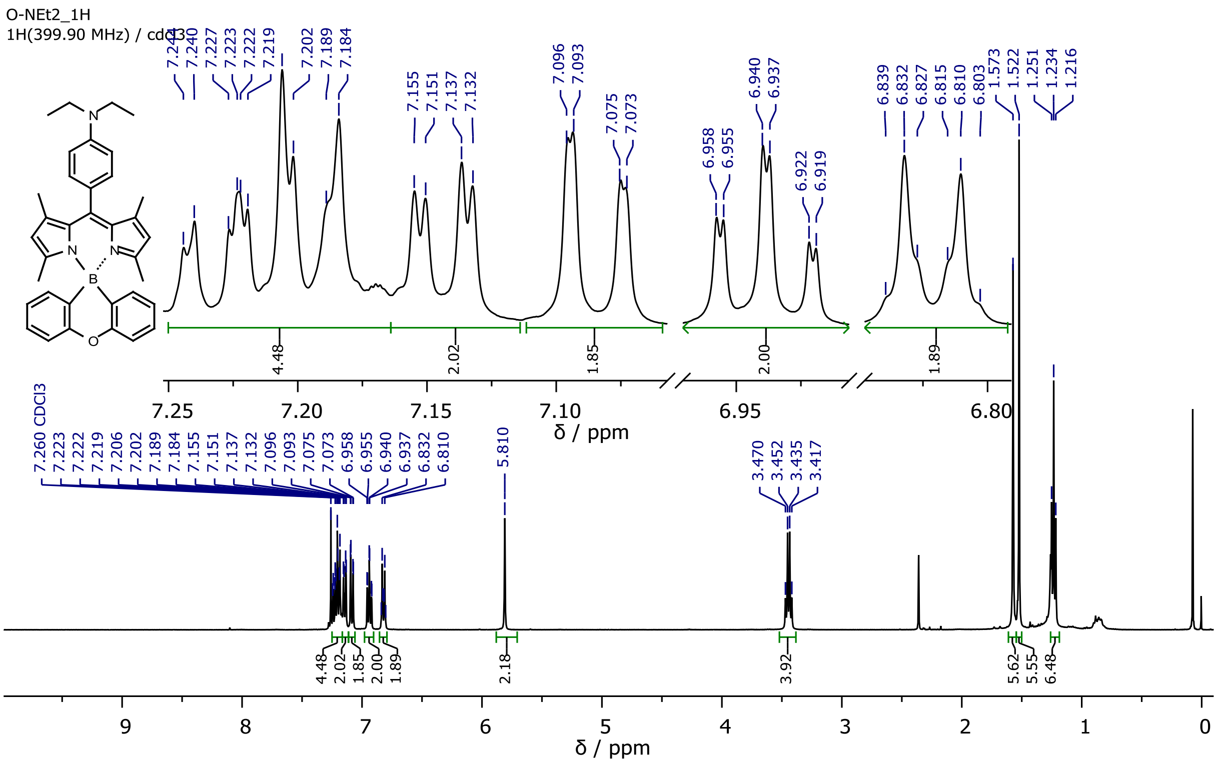

Figure S83. ^{1}H NMR spectrum of **O-BDP-NEt2** (400 MHz, $CDCl_3$).

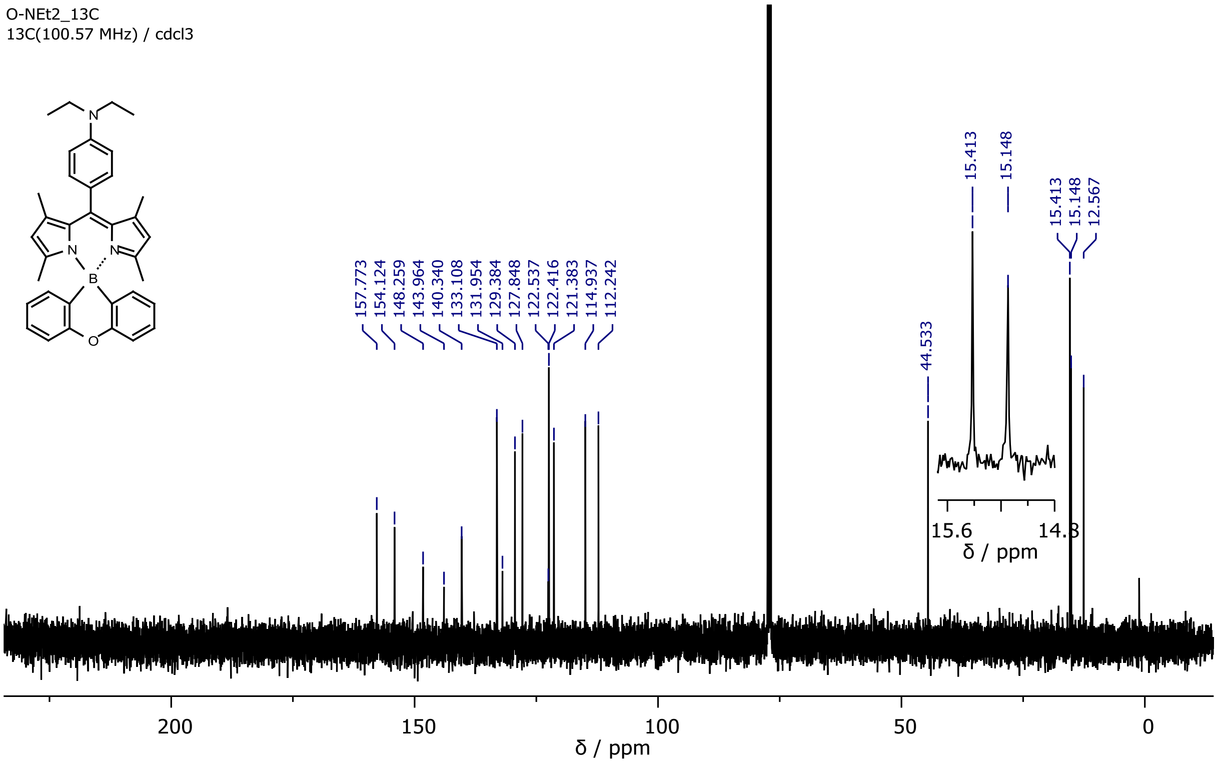

Figure S84. ^{13}C NMR spectrum of **O-BDP-NEt2** (101 MHz, $CDCl_3$).

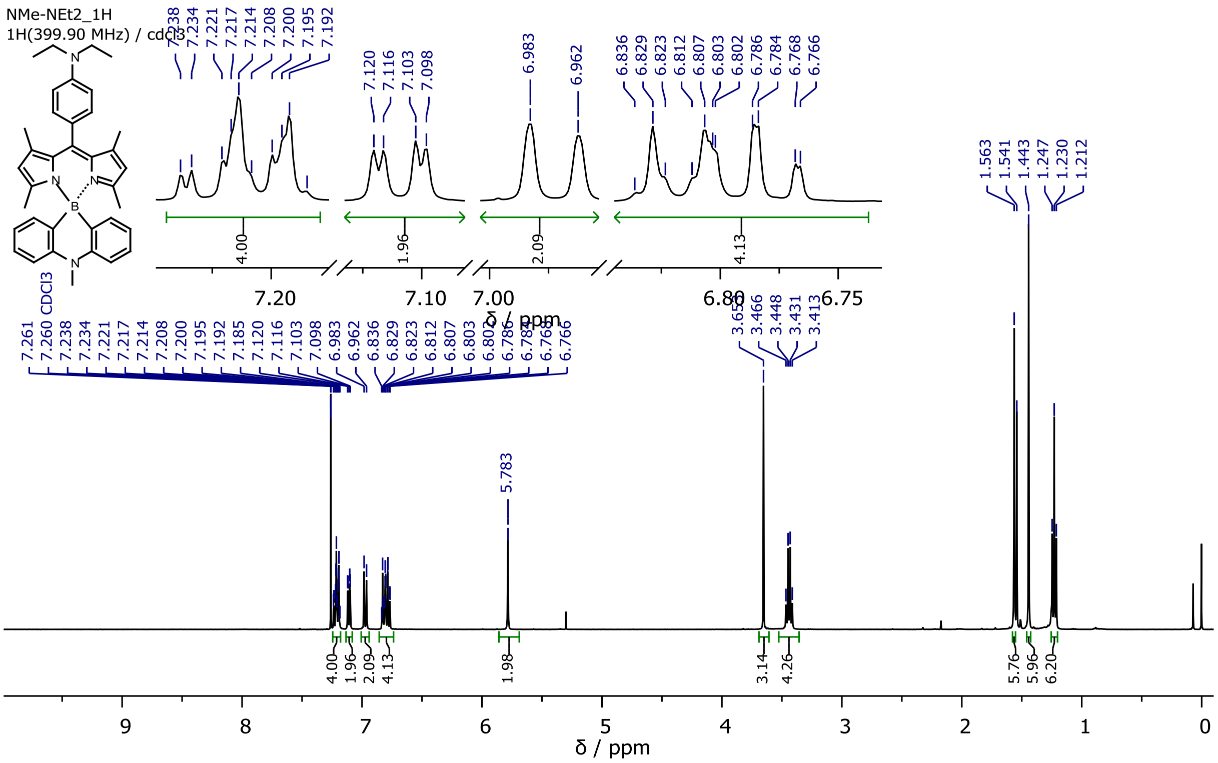

Figure S85. ^{1}H NMR spectrum of **NMe-BDP-NEt2** (400 MHz, $CDCl_3$).

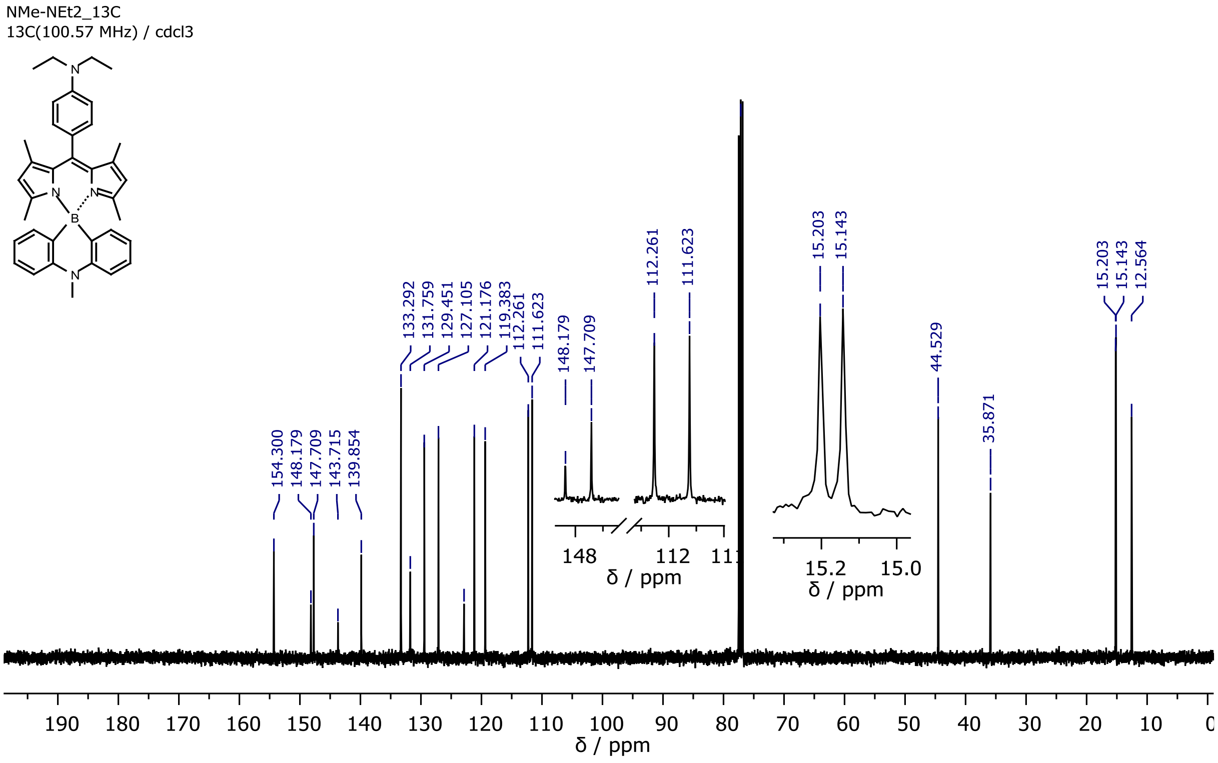

Figure S86. ^{13}C NMR spectrum of **NMe-BDP-NEt2** (101 MHz, $CDCl_3$).

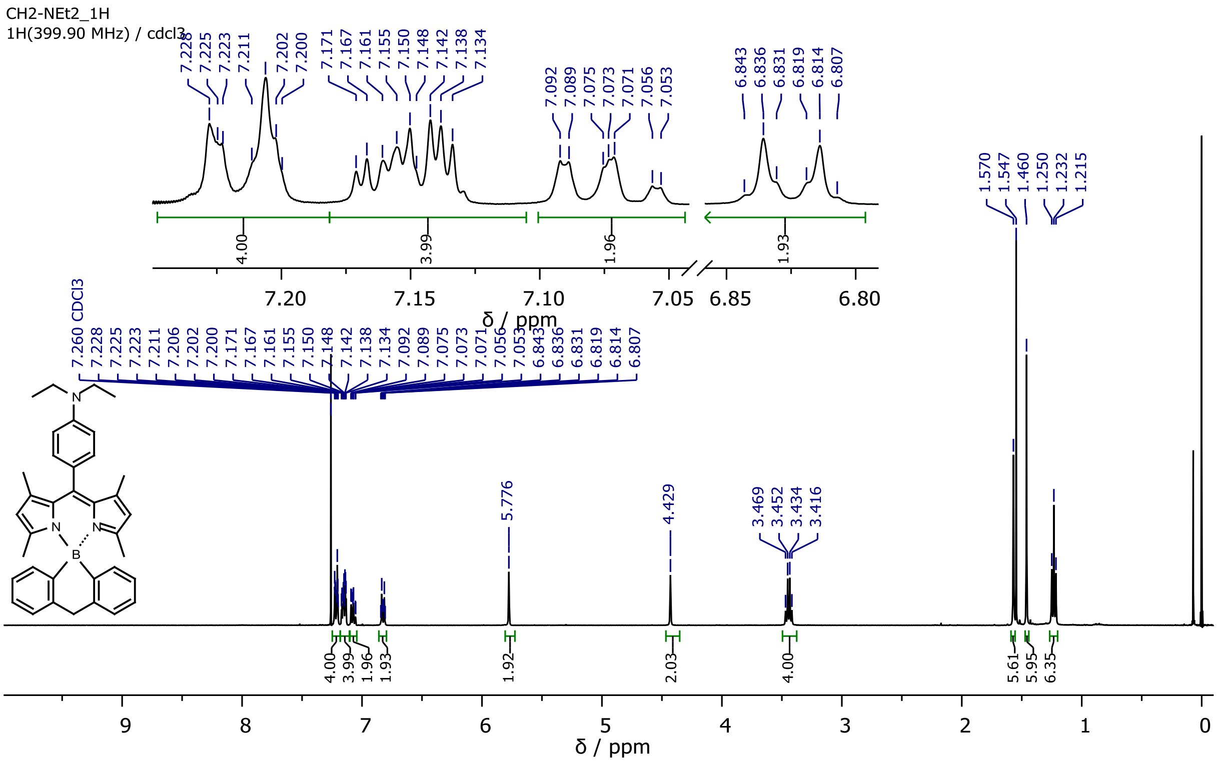

Figure S87. ^{1}H NMR spectrum of **CH2-BDP-NEt2** (400 MHz, $CDCl_3$).

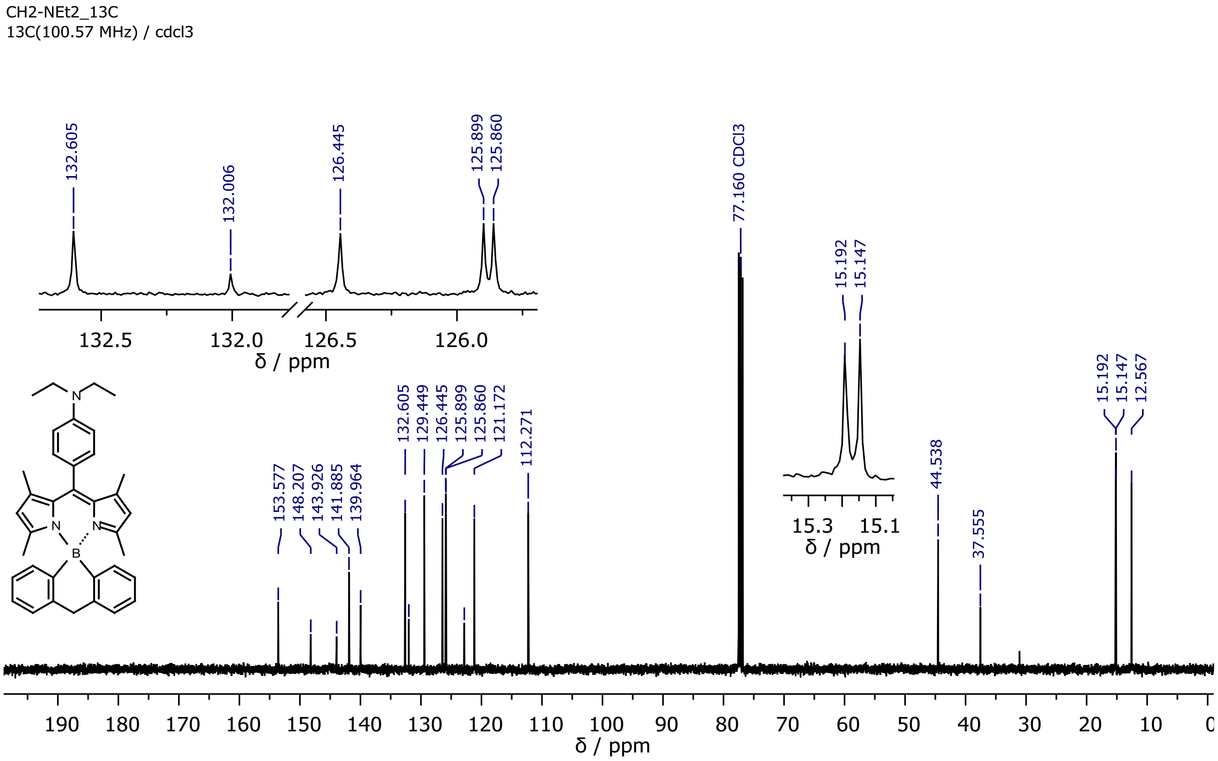

Figure S88. ^{13}C NMR spectrum of **CH2-BDP-NEt2** (101 MHz, $CDCl_3$).

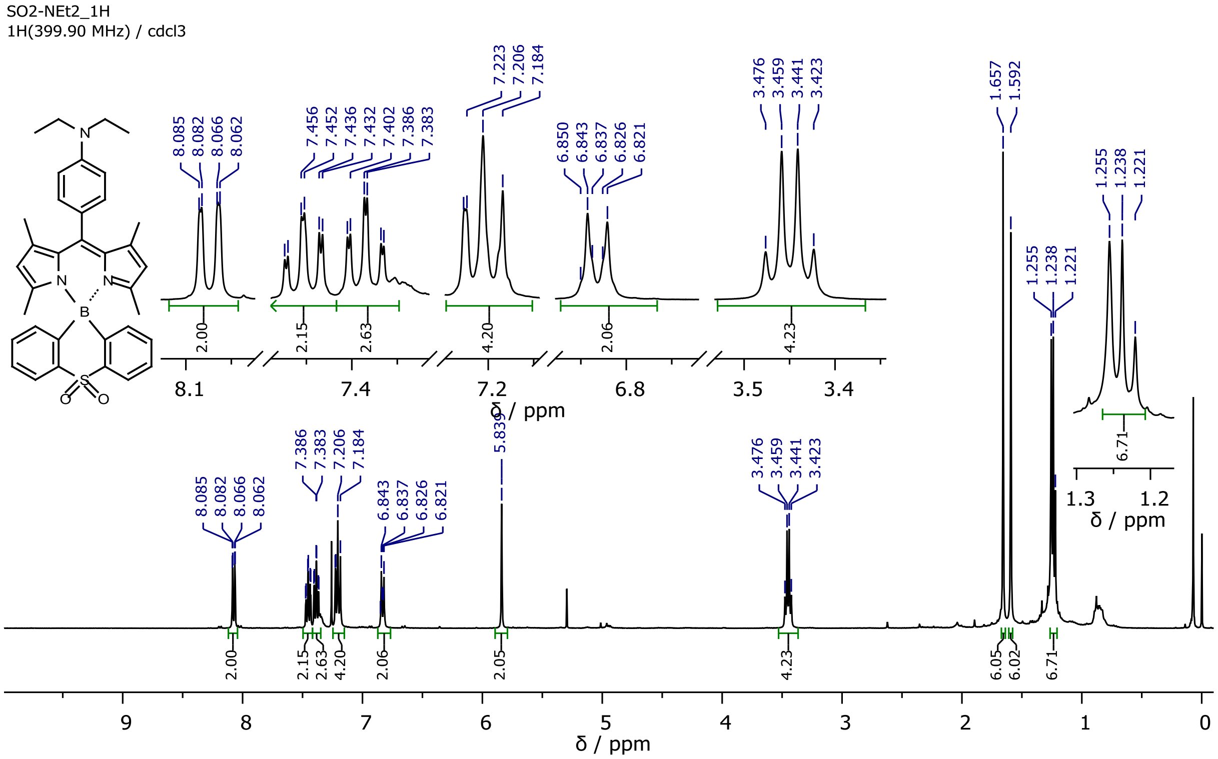

Figure S89. ^{1}H NMR spectrum of **SO2-BDP-NEt2** (400 MHz, $CDCl_3$).

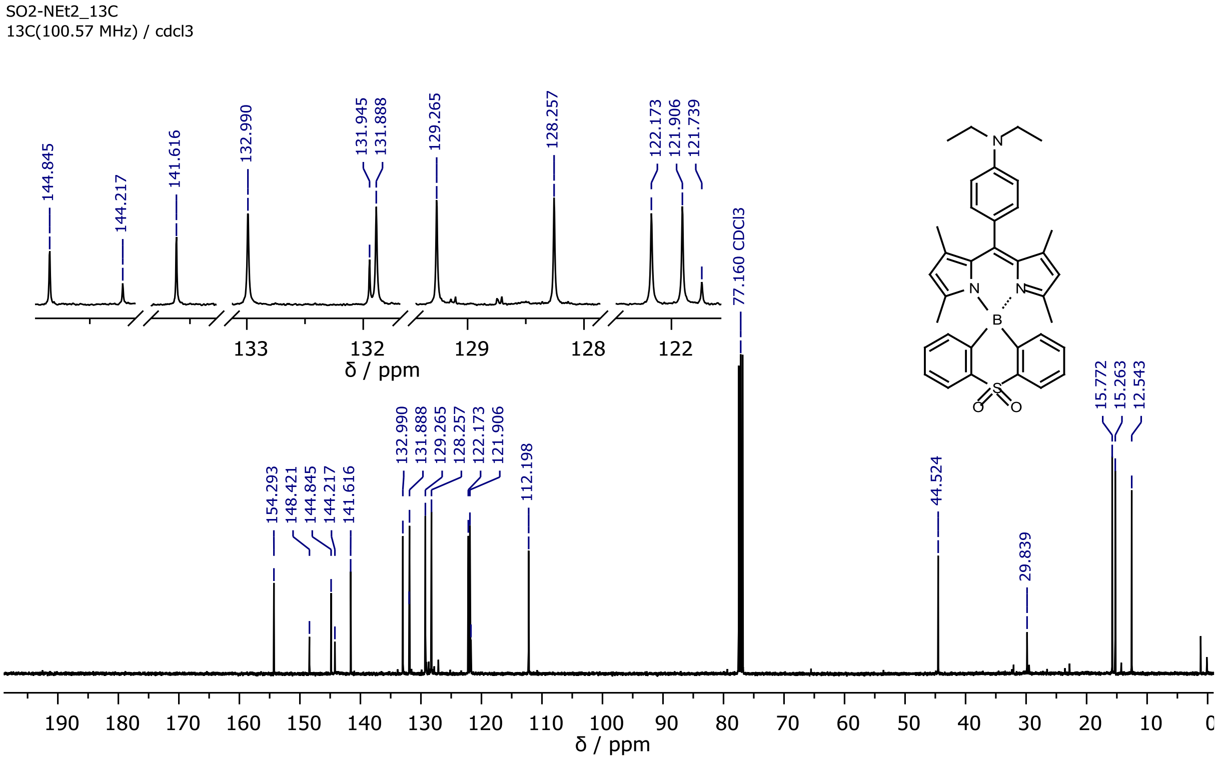

Figure S90. ^{13}C NMR spectrum of **SO2-BDP-NEt2** (101 MHz, $CDCl_3$).

9.2 NMR spectra of X-BDP-CAT

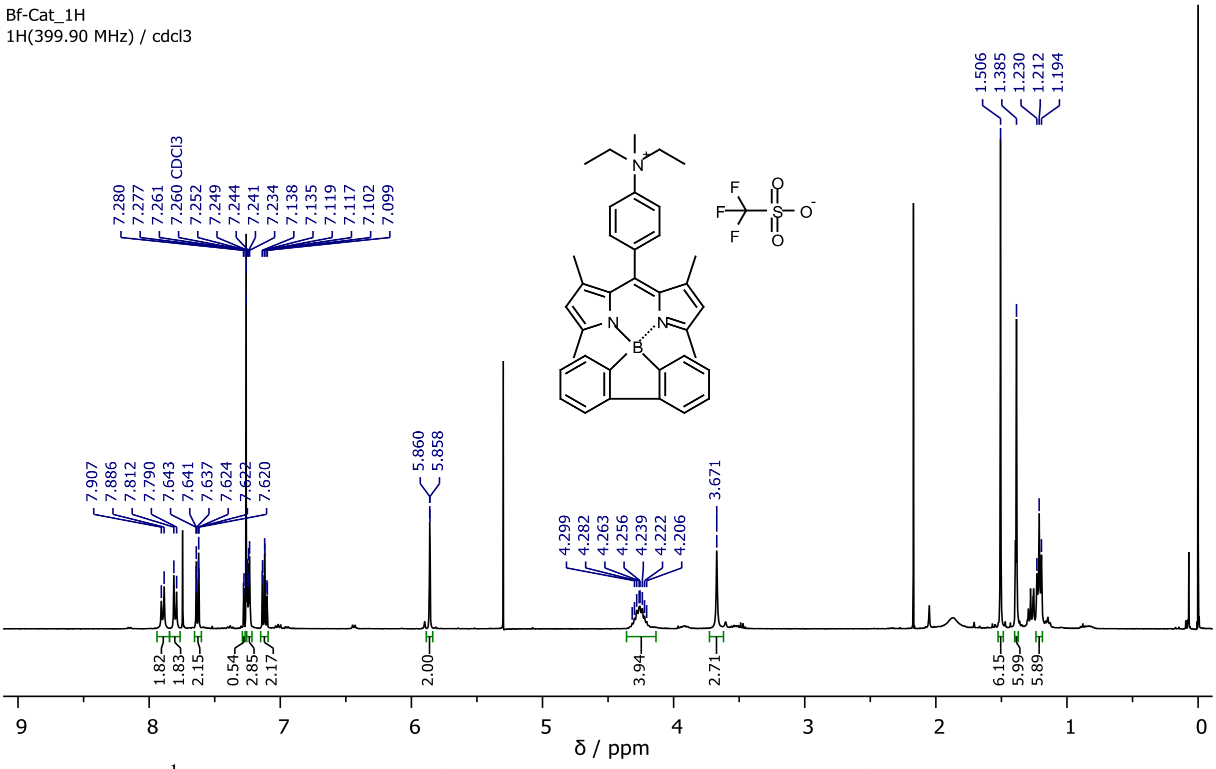

Figure S91. ^{1}H NMR spectrum of **Bf-BDP-CAT** (400 MHz, $CDCl_3$).

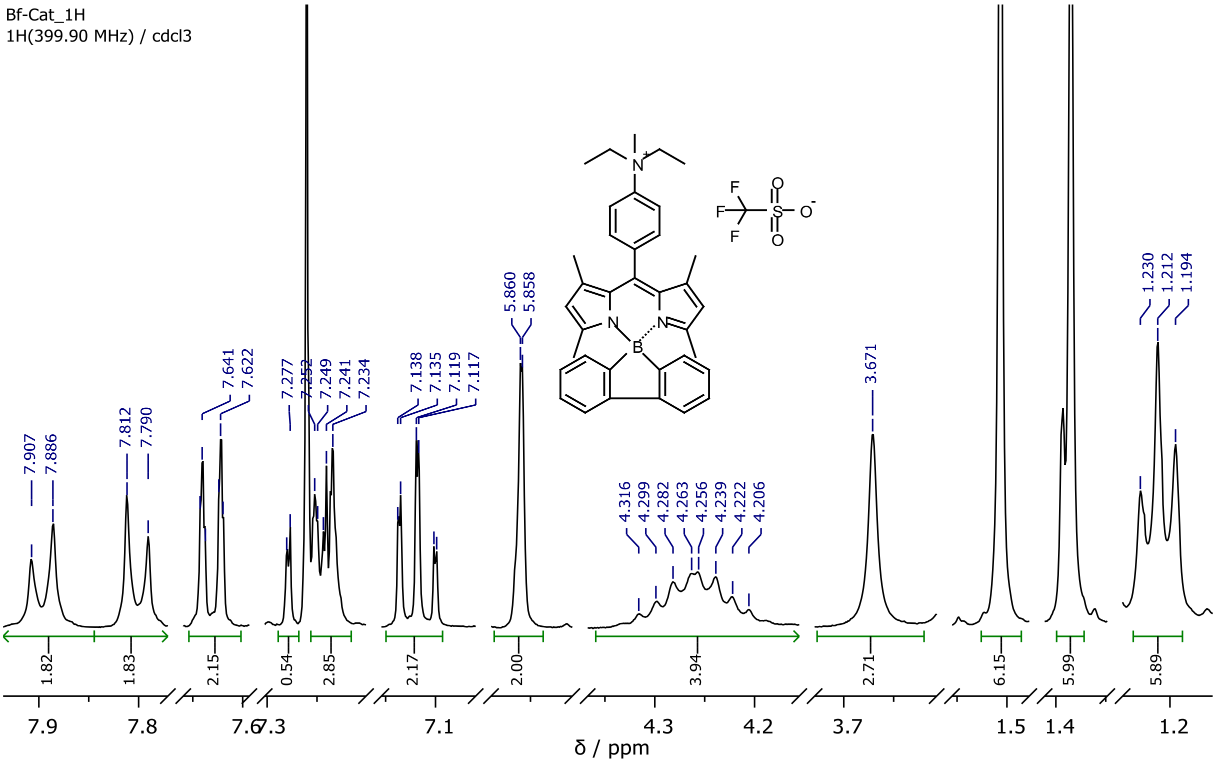

Figure S92. ^{1}H NMR spectrum of **Bf-BDP-CAT** (400 MHz, $CDCl_3$) – zoom.

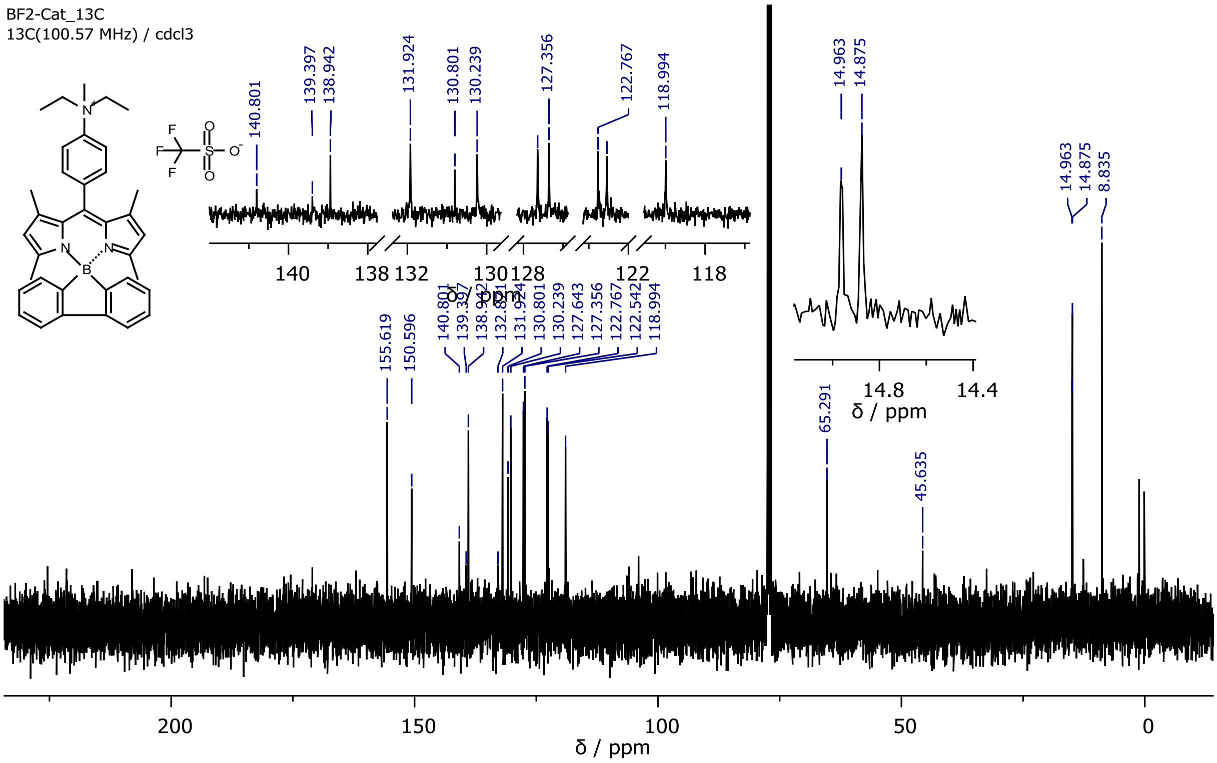

Figure S93. ^{13}C NMR spectrum of **Bf-BDP-CAT** (101 MHz, $CDCl_3$).

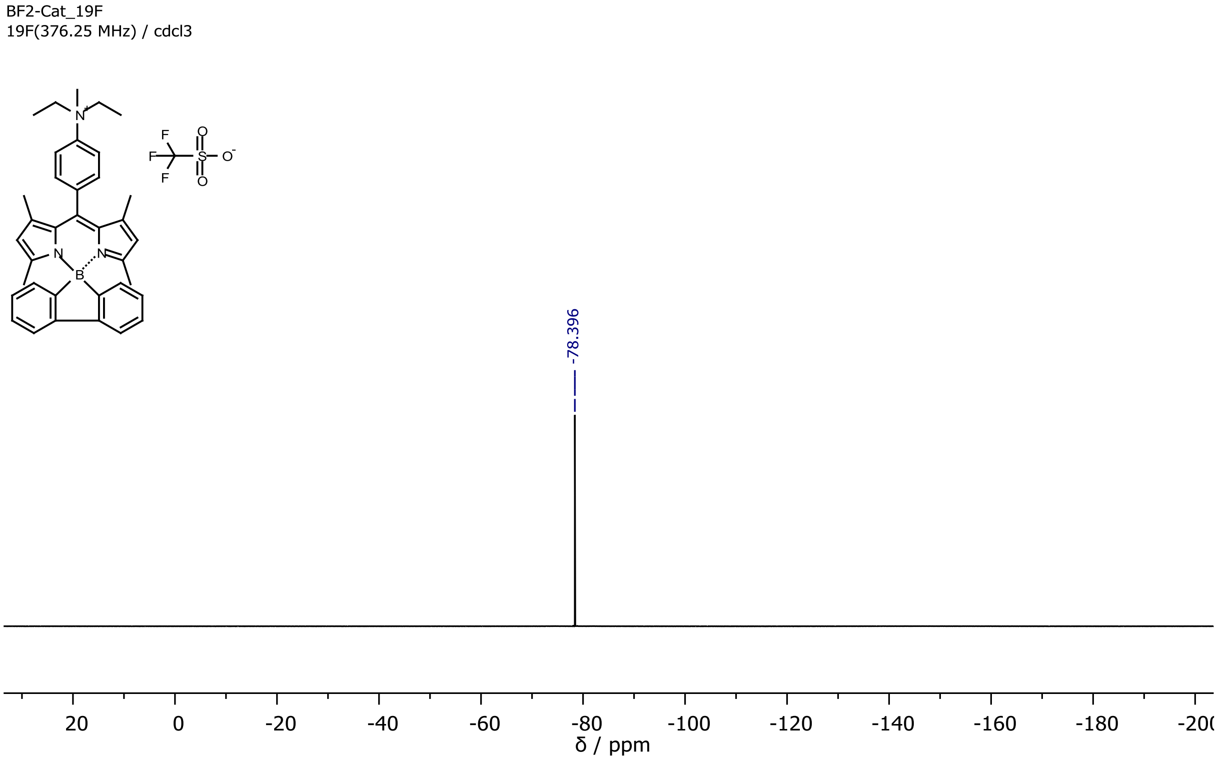

Figure S94. ^{19}F NMR spectrum of **Bf-BDP-CAT** (376 MHz, $CDCl_3$).

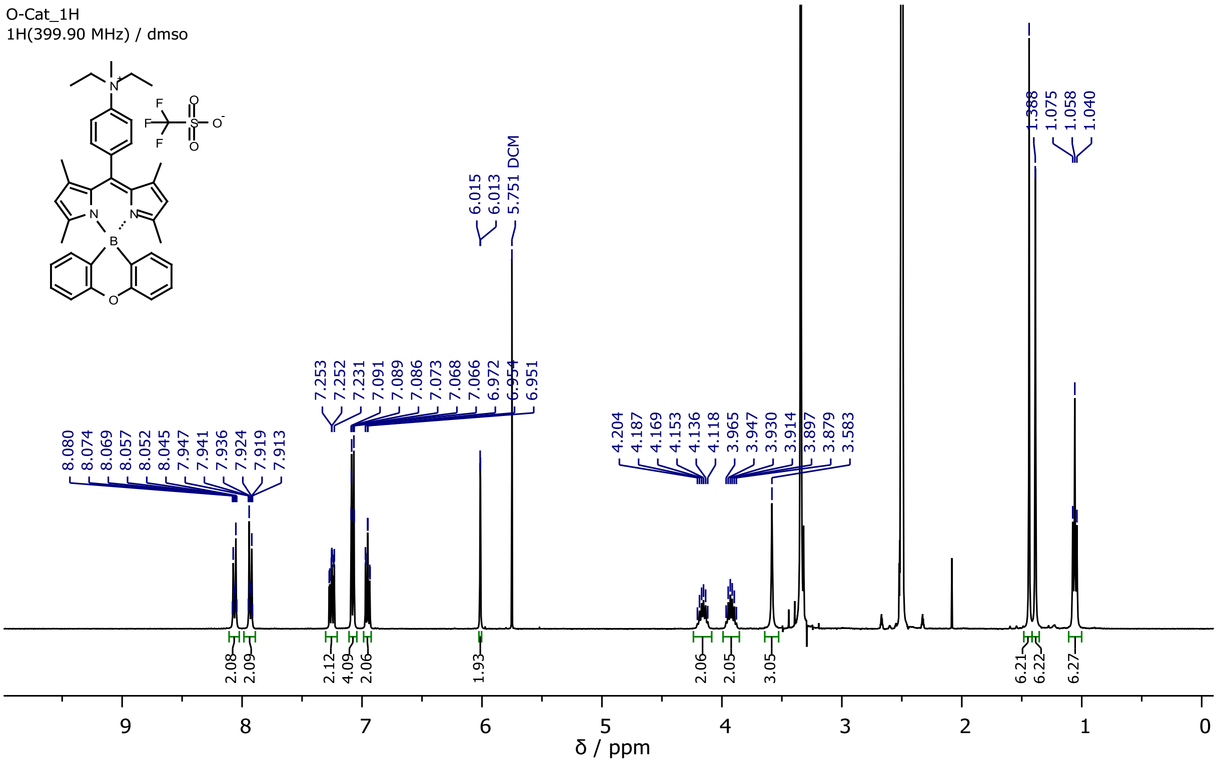

Figure S95. ^{1}H NMR spectrum of **O-BDP-CAT** (400 MHz, DMSO-d_6).

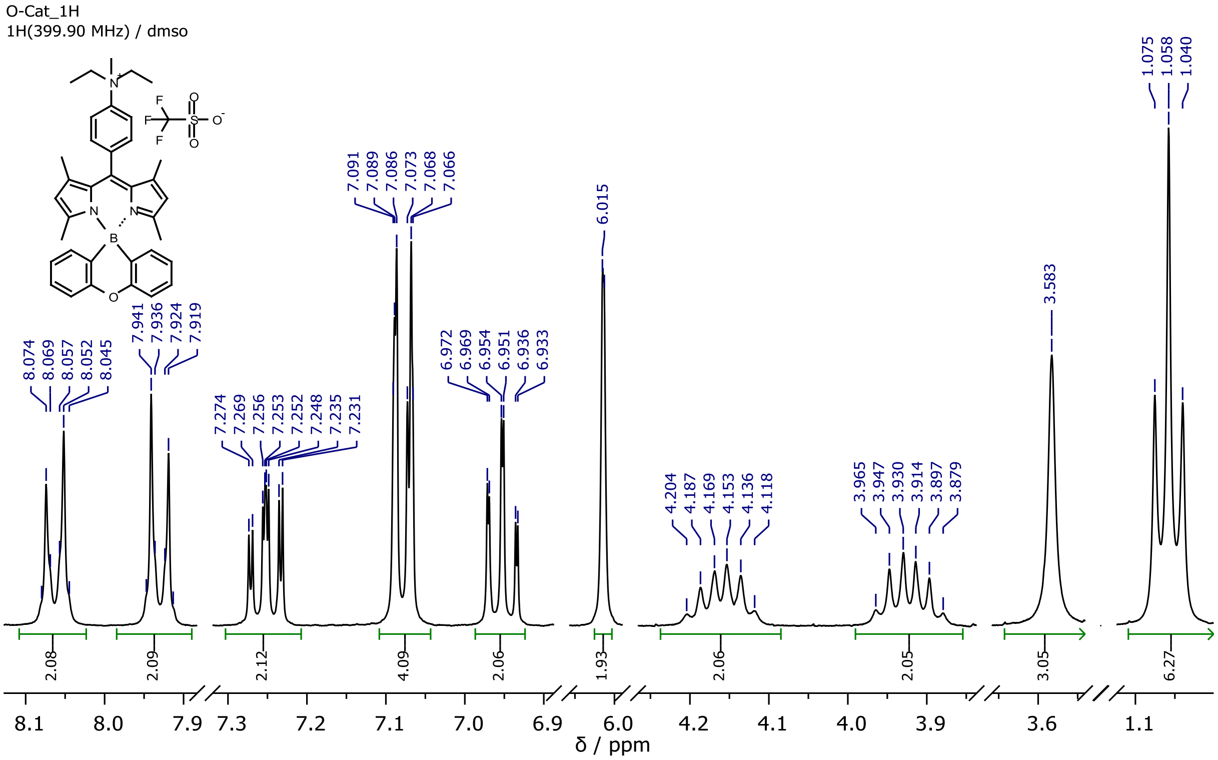

Figure S96. ^{1}H NMR spectrum of **O-BDP-CAT** (400 MHz, DMSO-d_6)- zoom.

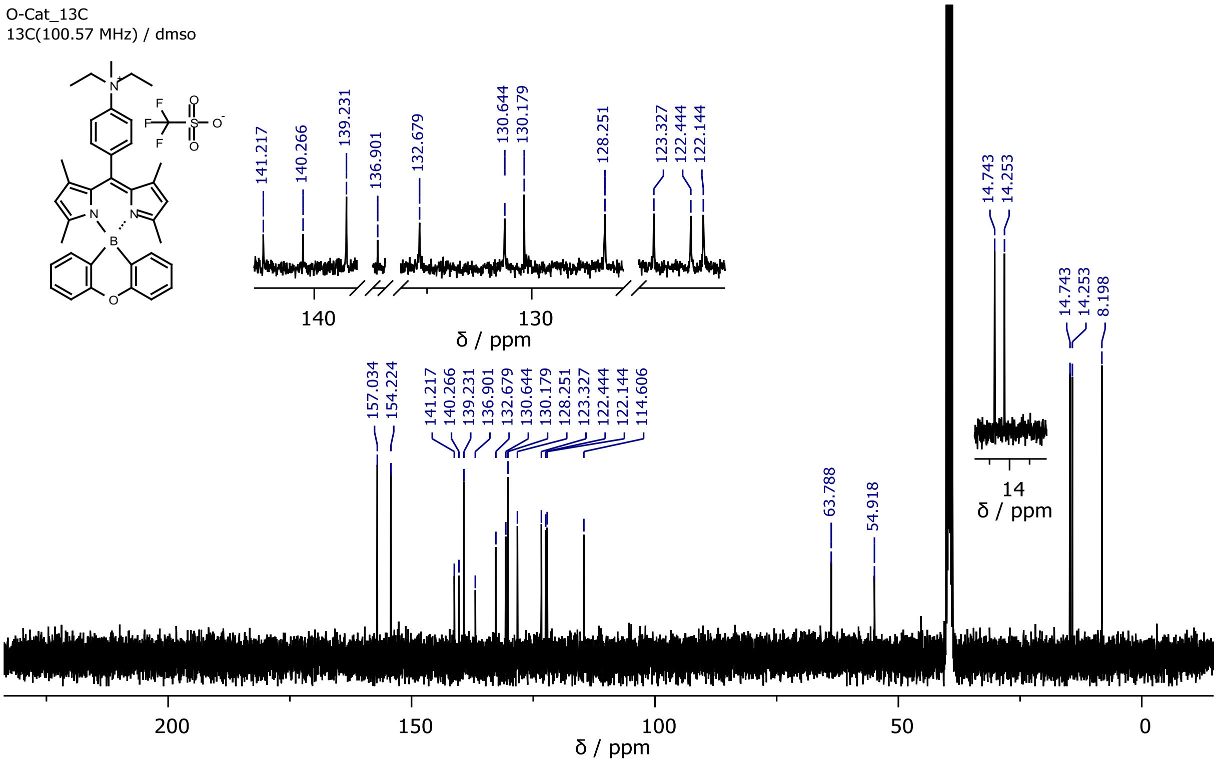

Figure S97. ^{13}C NMR spectrum of **O-BDP-CAT** (101 MHz, DMSO-d_6).

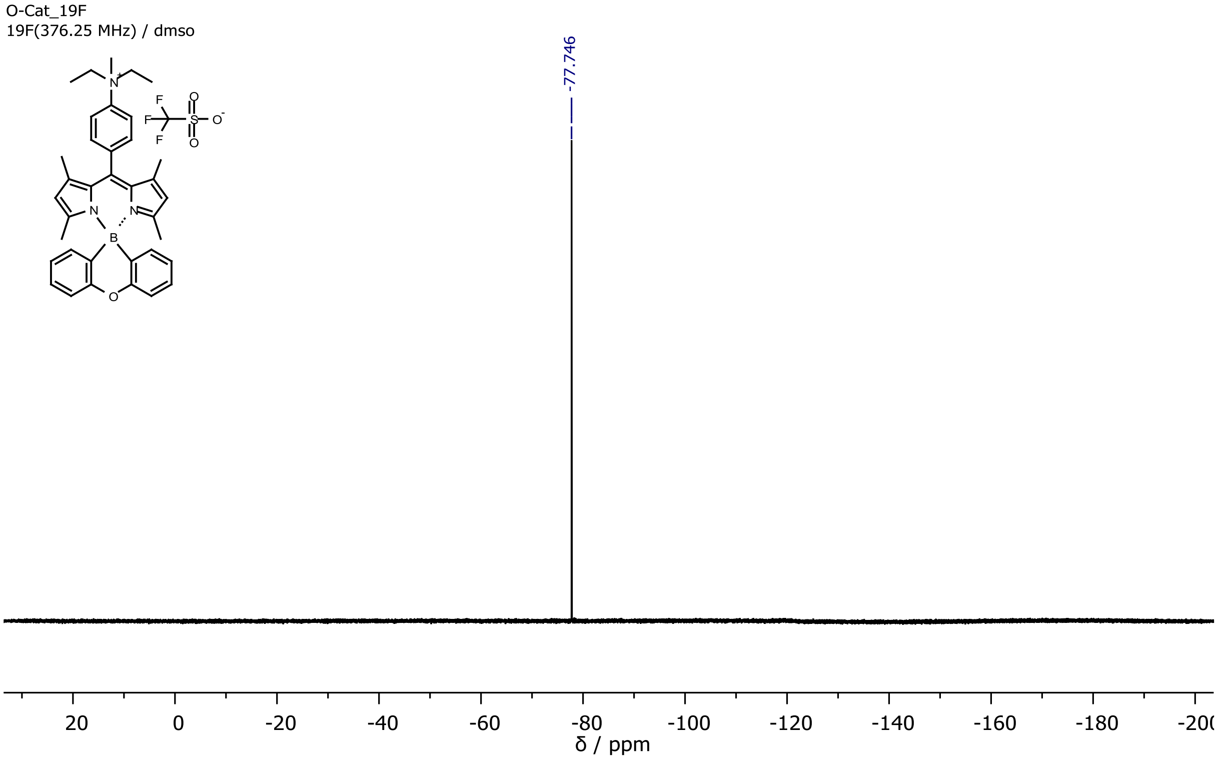

Figure S98. ^{19}F NMR spectrum of **O-BDP-CAT** (376 MHz, DMSO-d_6).

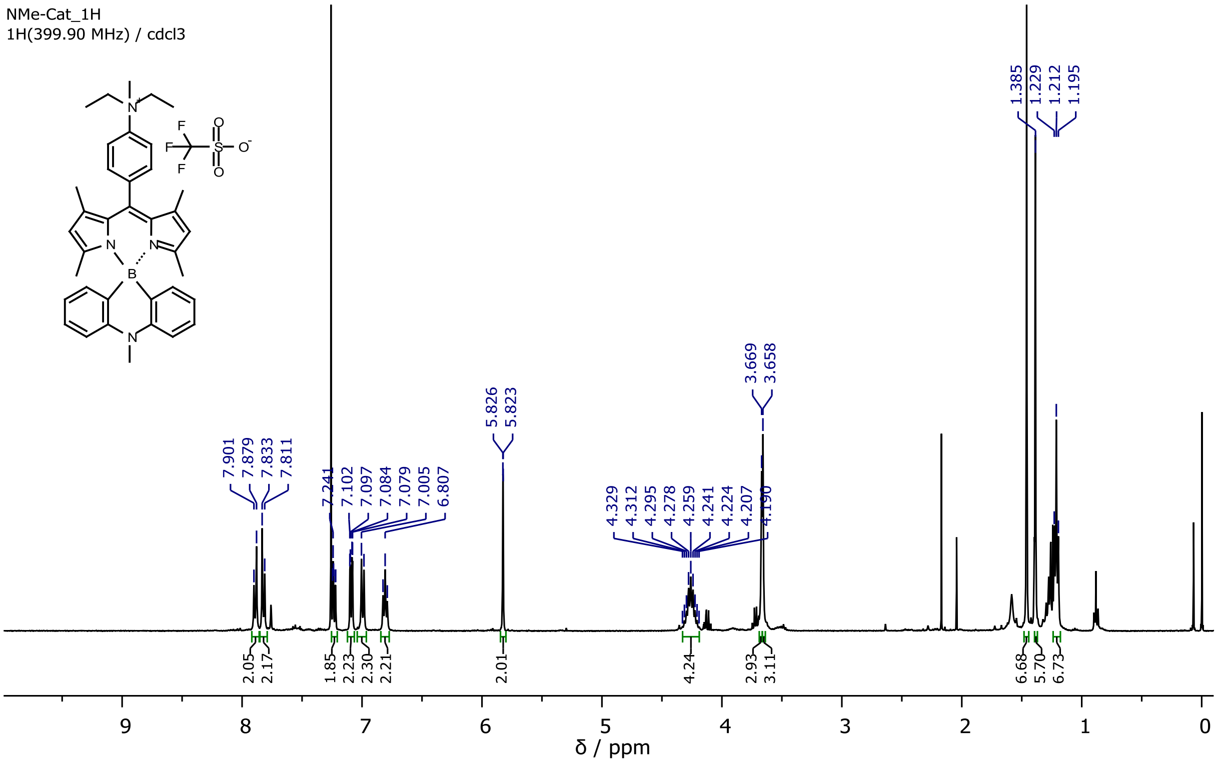

Figure S99. ^{1}H NMR spectrum of **NMe-BDP-CAT** (400 MHz, $CDCl_3$).

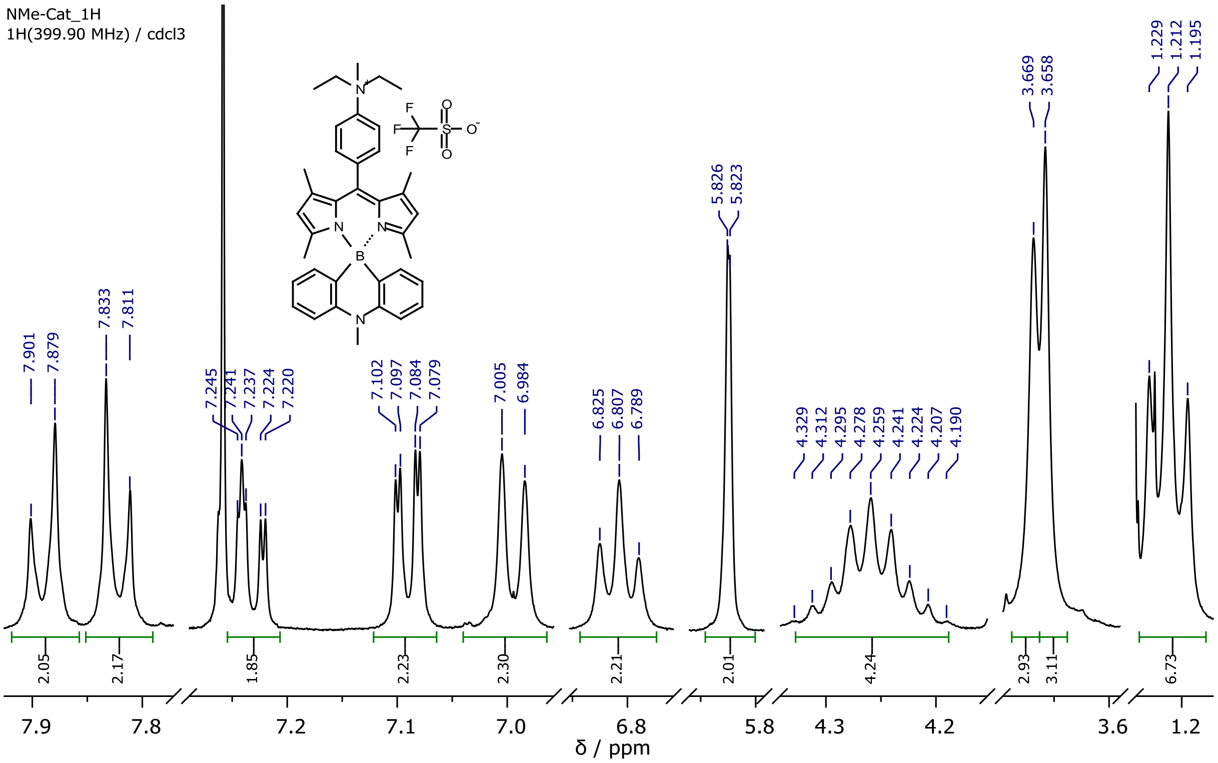

Figure S100. ^{1}H NMR spectrum of **NMe-BDP-CAT** (400 MHz, $CDCl_3$) – zoom.

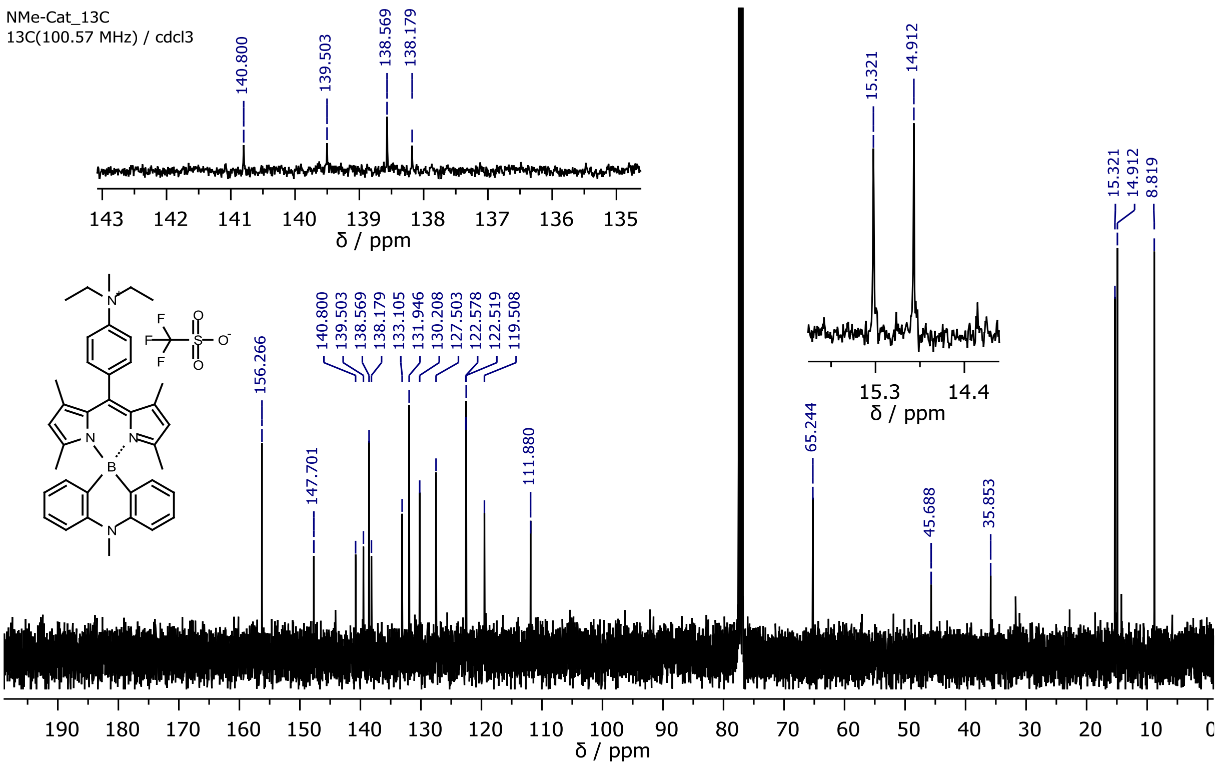

Figure S101. ^{13}C NMR spectrum of **NMe-BDP-CAT** (101 MHz, $CDCl_3$).

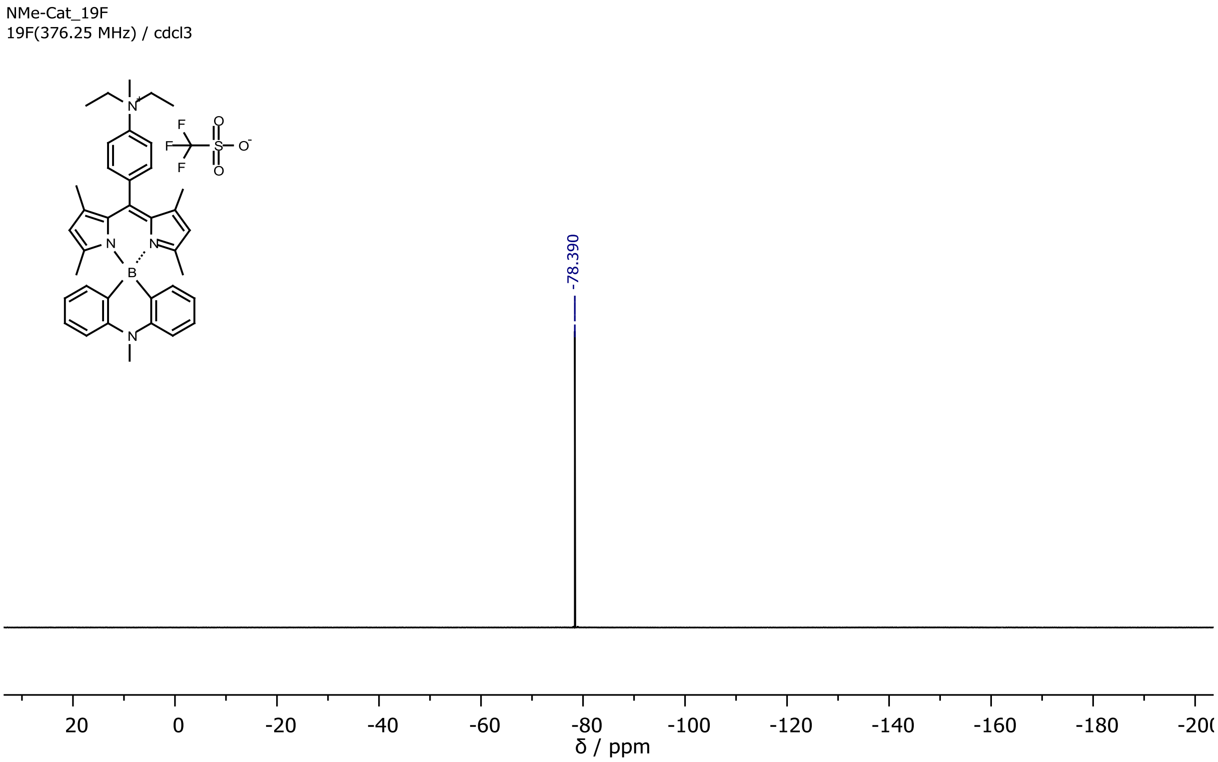

Figure S102. ^{19}F NMR spectrum of **NMe-BDP-CAT** (376 MHz, $CDCl_3$).

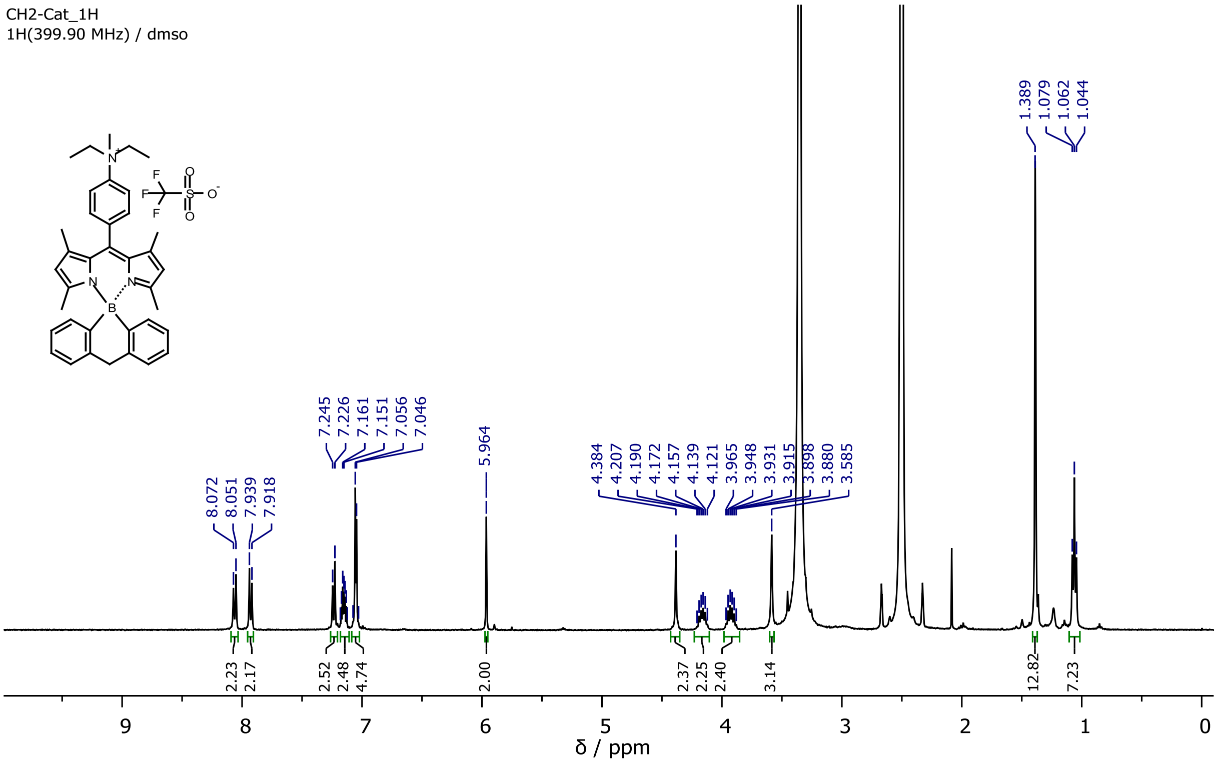

Figure S103. ^{1}H NMR spectrum of **CH$_2$-BDP-CAT** (400 MHz, DMSO-d_6).

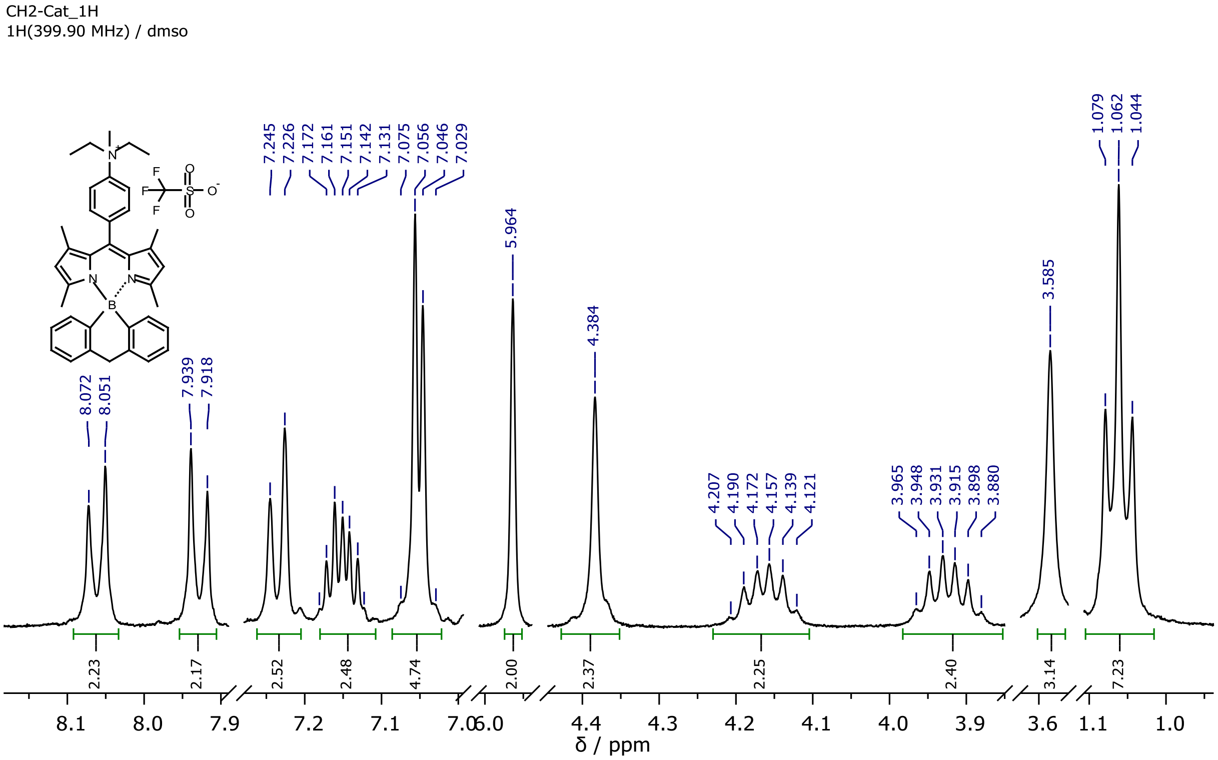

Figure S104. ^{1}H NMR spectrum of **CH$_2$-BDP-CAT** (400 MHz, DMSO-d_6) – zoom.

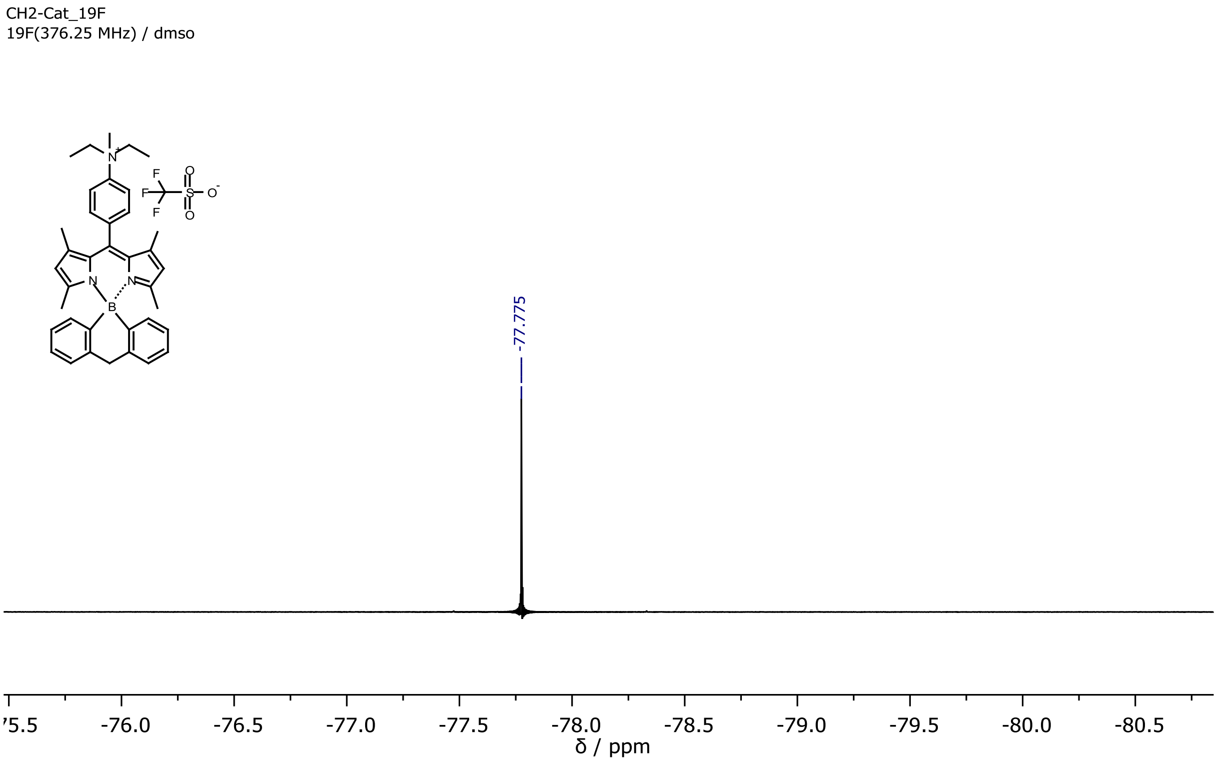

Figure S105. ^{19}F NMR spectrum of **CH$_2$-BDP-CAT** (376 MHz, DMSO-d_6).

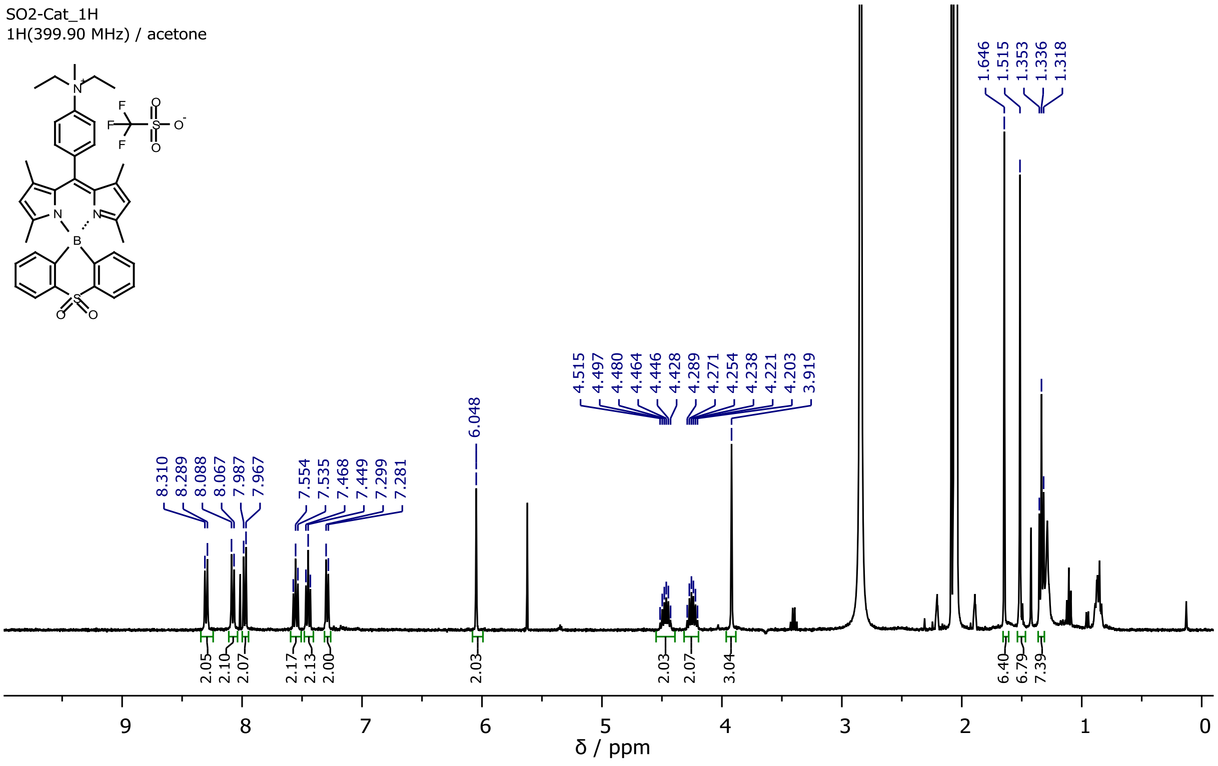

Figure S106. ^{1}H NMR spectrum of **SO$_2$-BDP-CAT** (400 MHz, acetone-d_6).

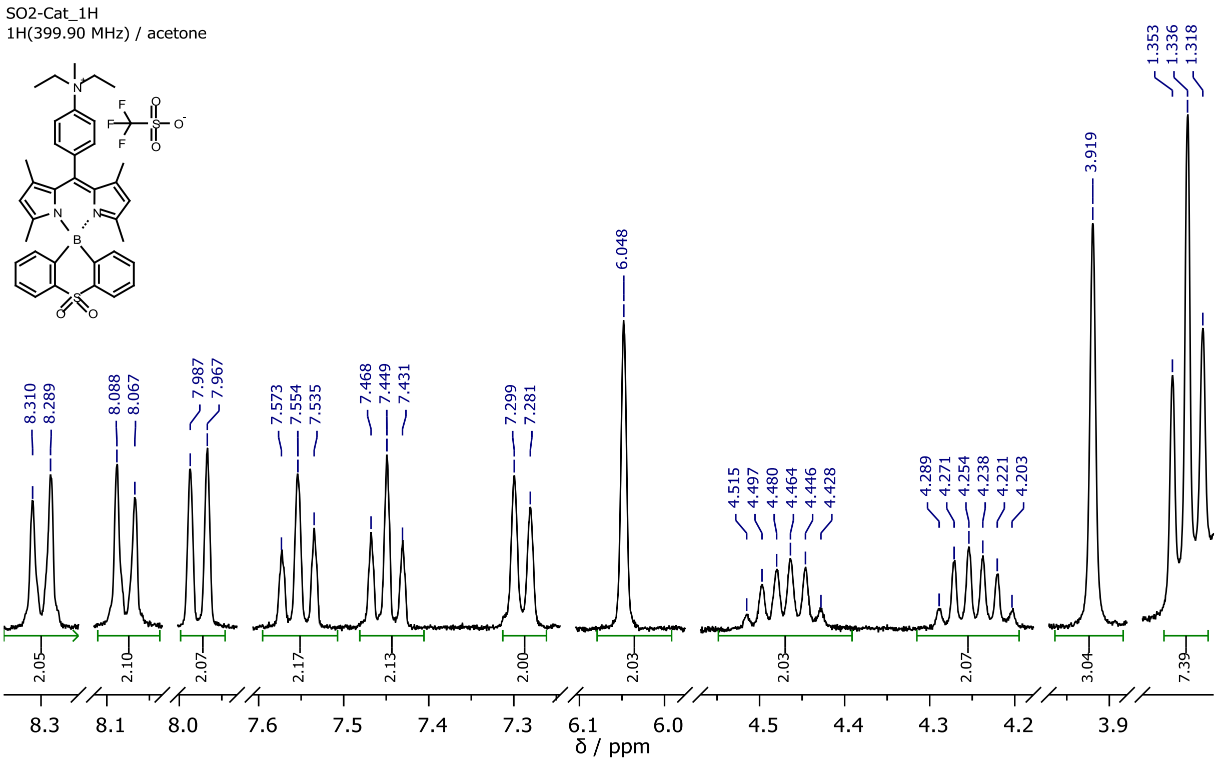

Figure S107. ^{1}H NMR spectrum of **SO$_2$-BDP-CAT** (400 MHz, acetone-d_6) – zoom.

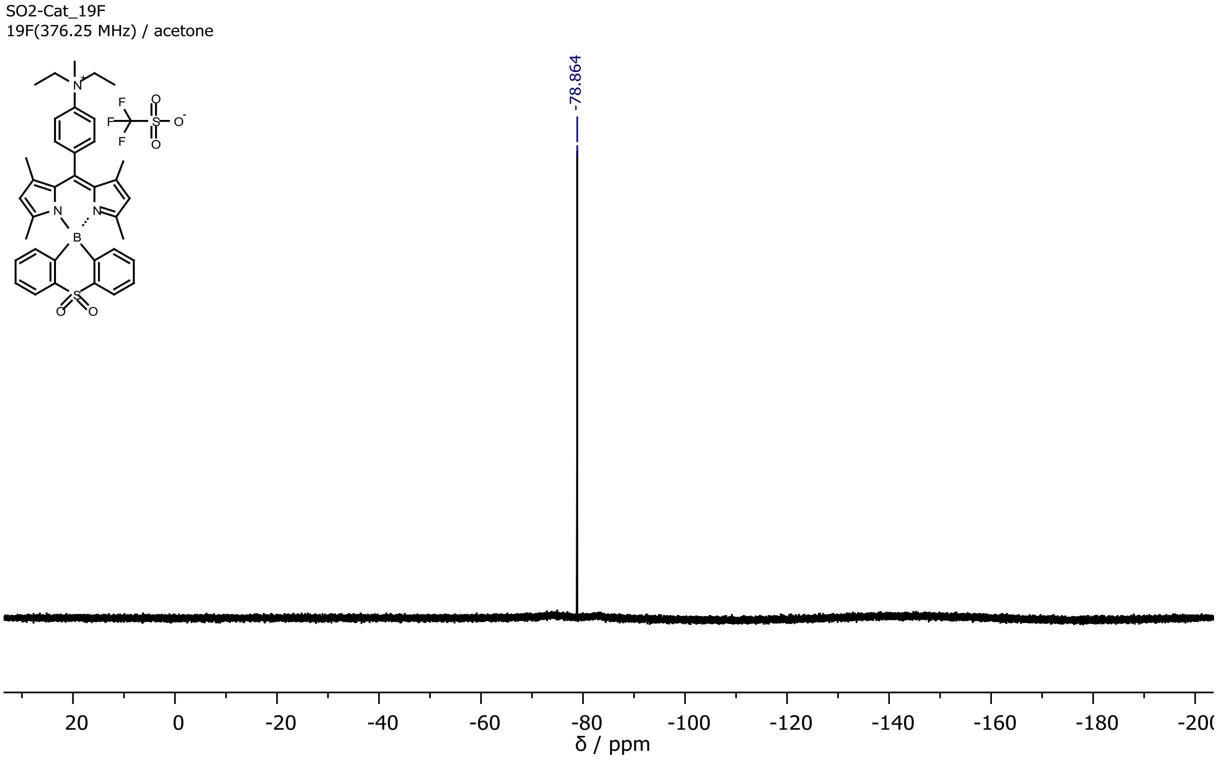

Figure S108. ^{19}F NMR spectrum of **SO$_2$-BDP-CAT** (376 MHz, acetone-d_6).

9.3 NMR spectra of X-BDP-I

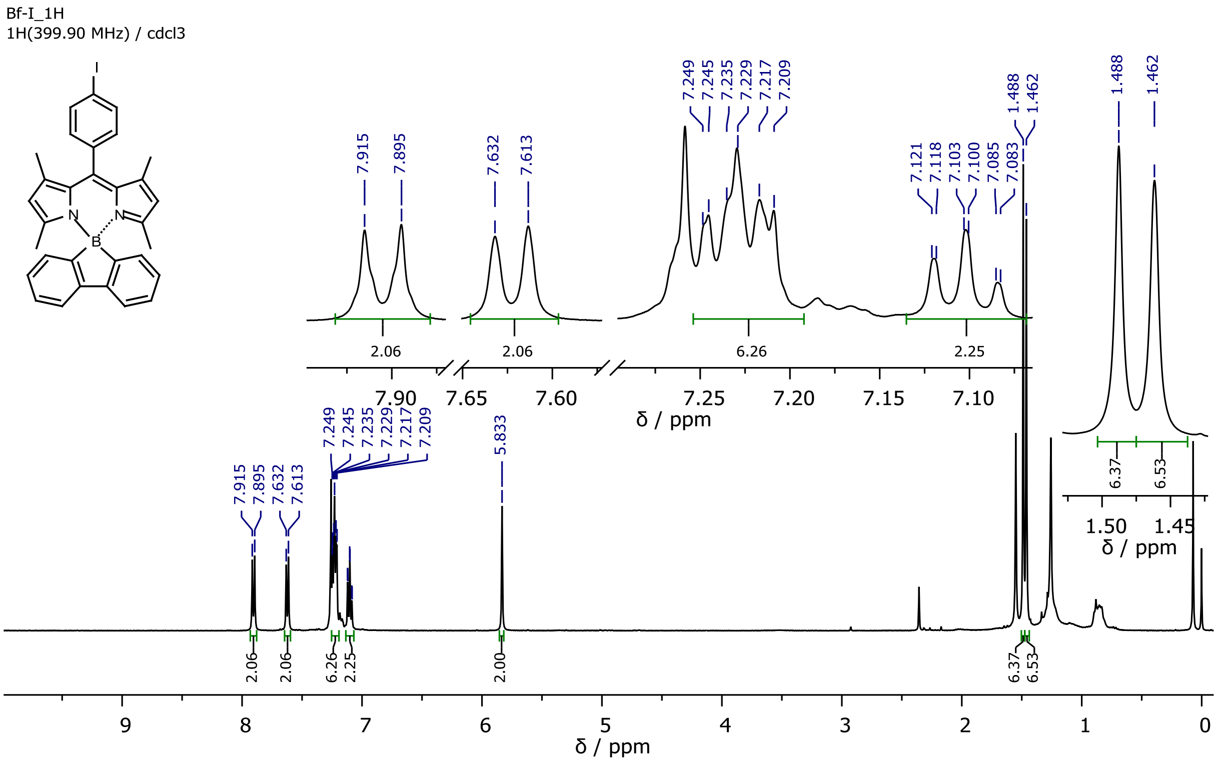

Figure S109. ^{1}H NMR spectrum of **Bf-BDP-I** (400 MHz, $CDCl_3$).

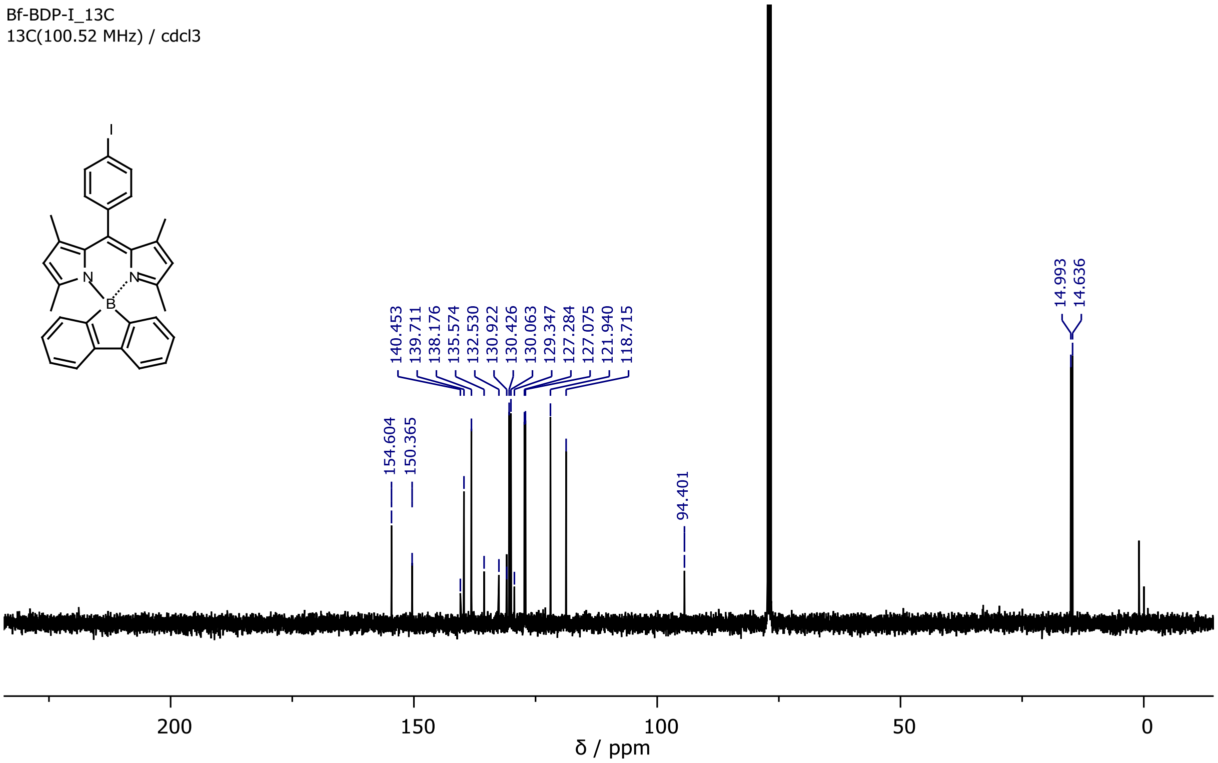

Figure S110. ^{13}C NMR spectrum of **Bf-BDP-I** (101 MHz, $CDCl_3$).

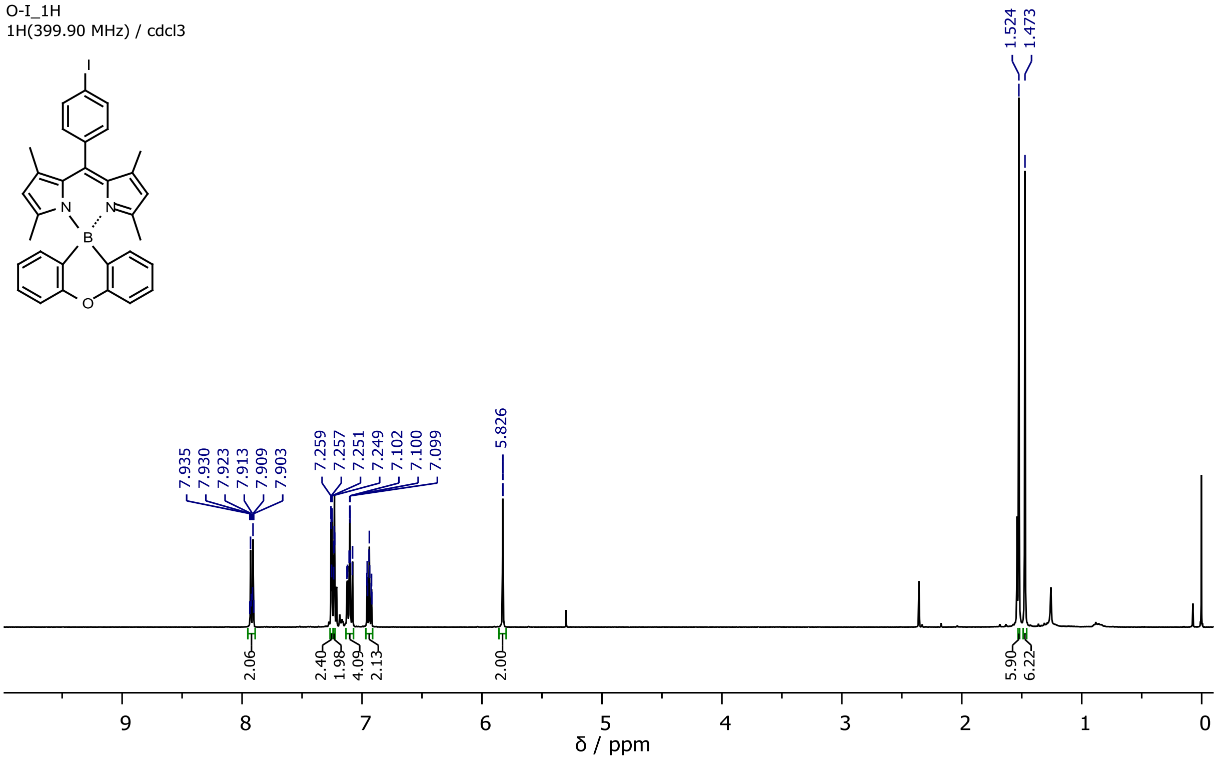

Figure S111. ^{1}H NMR spectrum of **O-BDP-I** (400 MHz, $CDCl_3$).

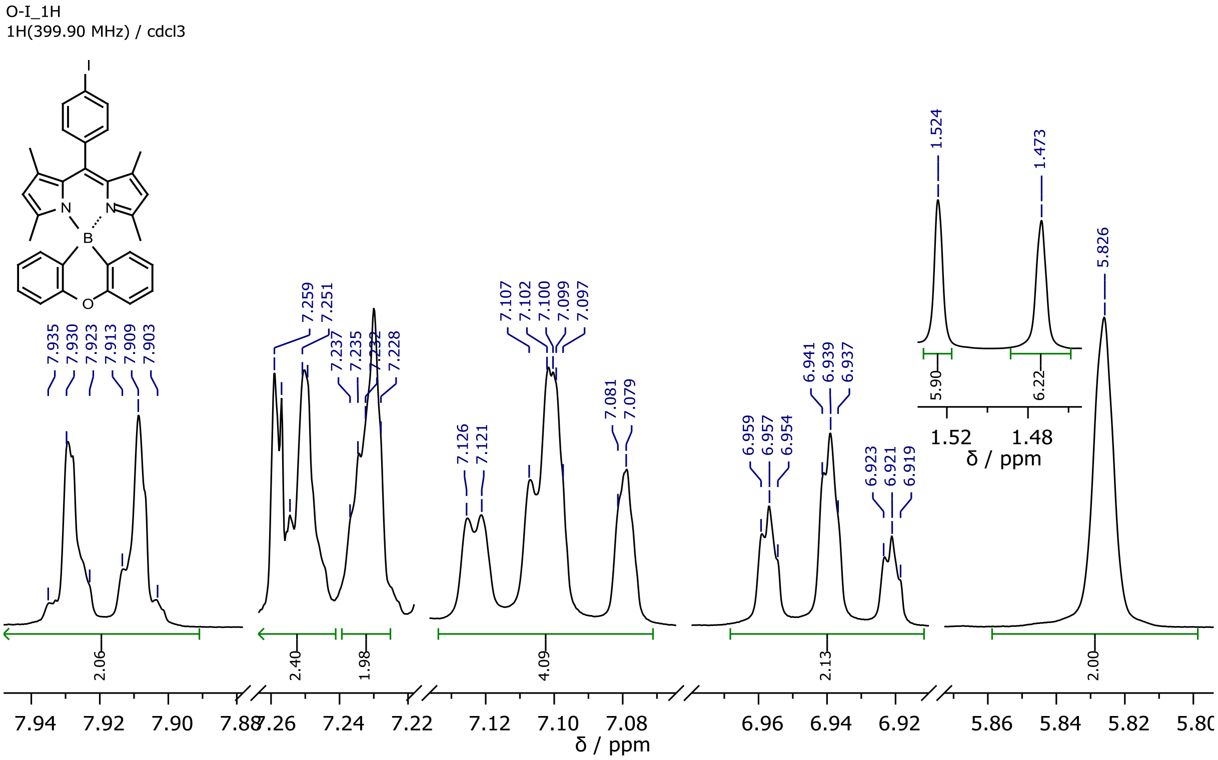

Figure S112. ^{1}H NMR spectrum of **O-BDP-I** (400 MHz, $CDCl_3$) – zoom.

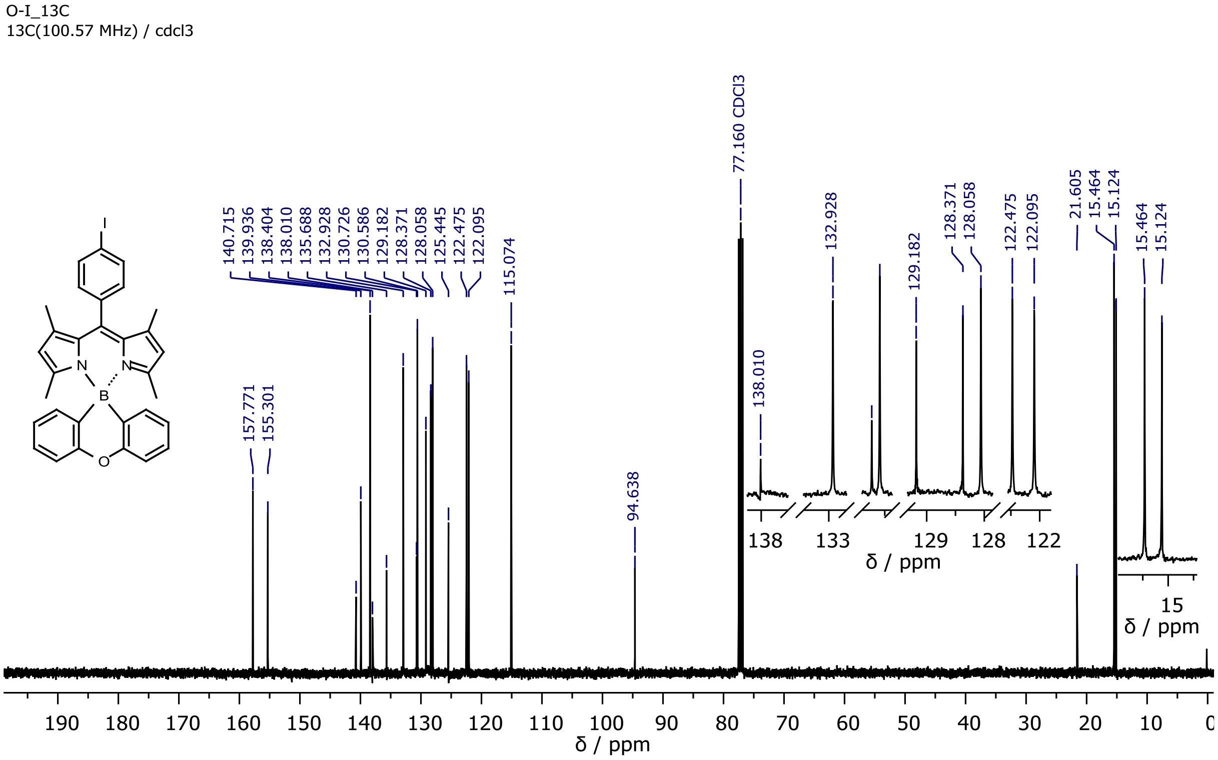

Figure S113. ^{13}C NMR spectrum of **O-BDP-I** (101 MHz, $CDCl_3$).

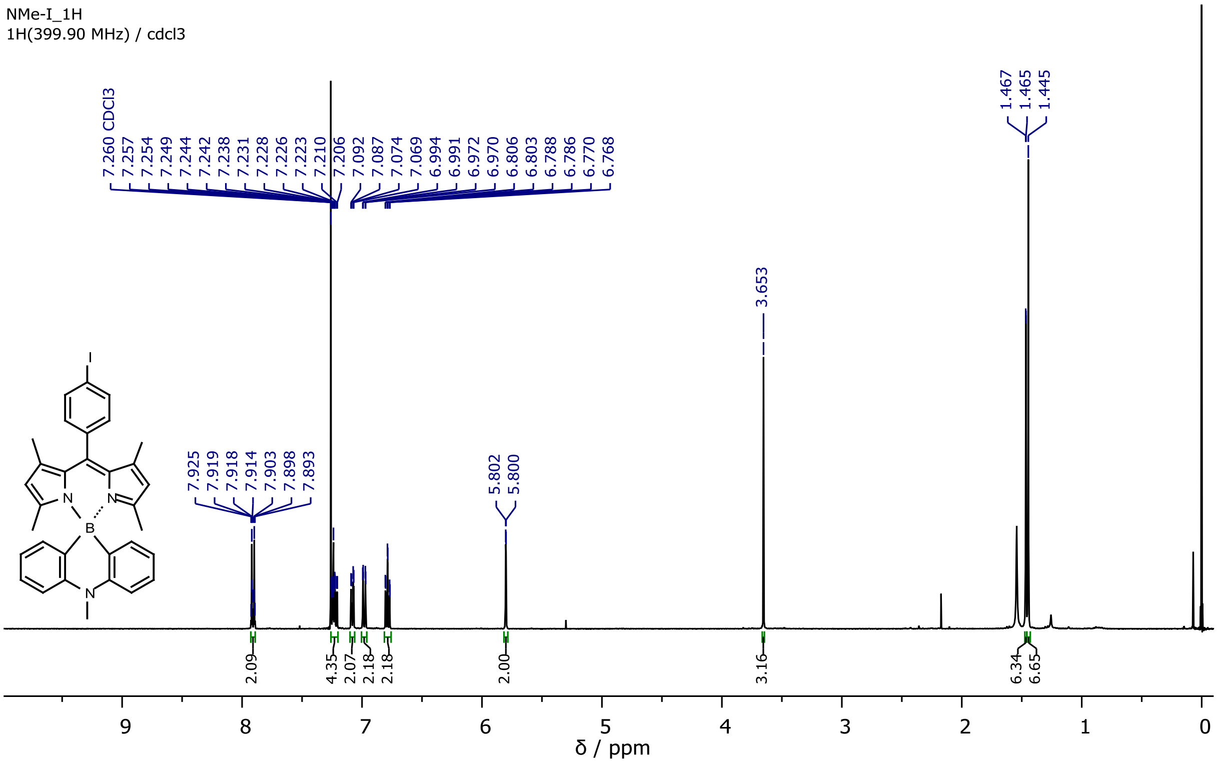

Figure S114. ^{1}H NMR spectrum of **NMe-BDP-I** (400 MHz, $CDCl_3$).

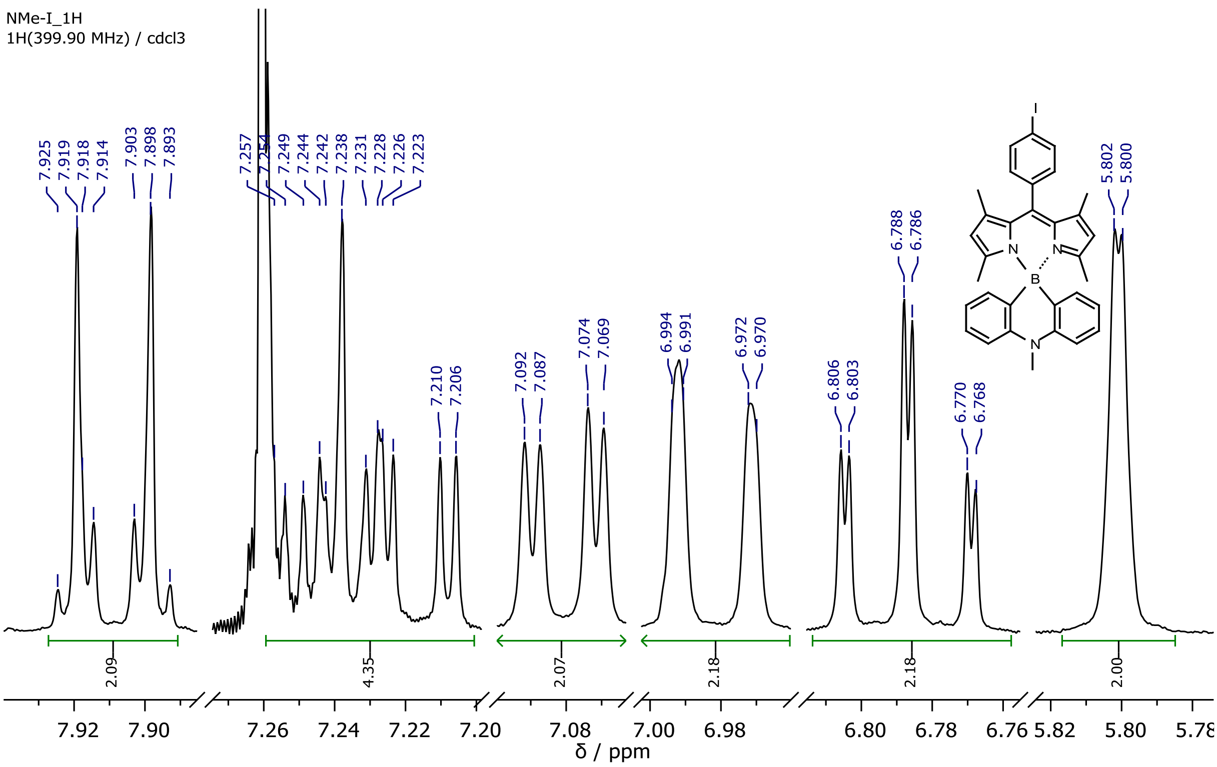

Figure S115. ^{1}H NMR spectrum of **NMe-BDP-I** (400 MHz, $CDCl_3$) -zoom.

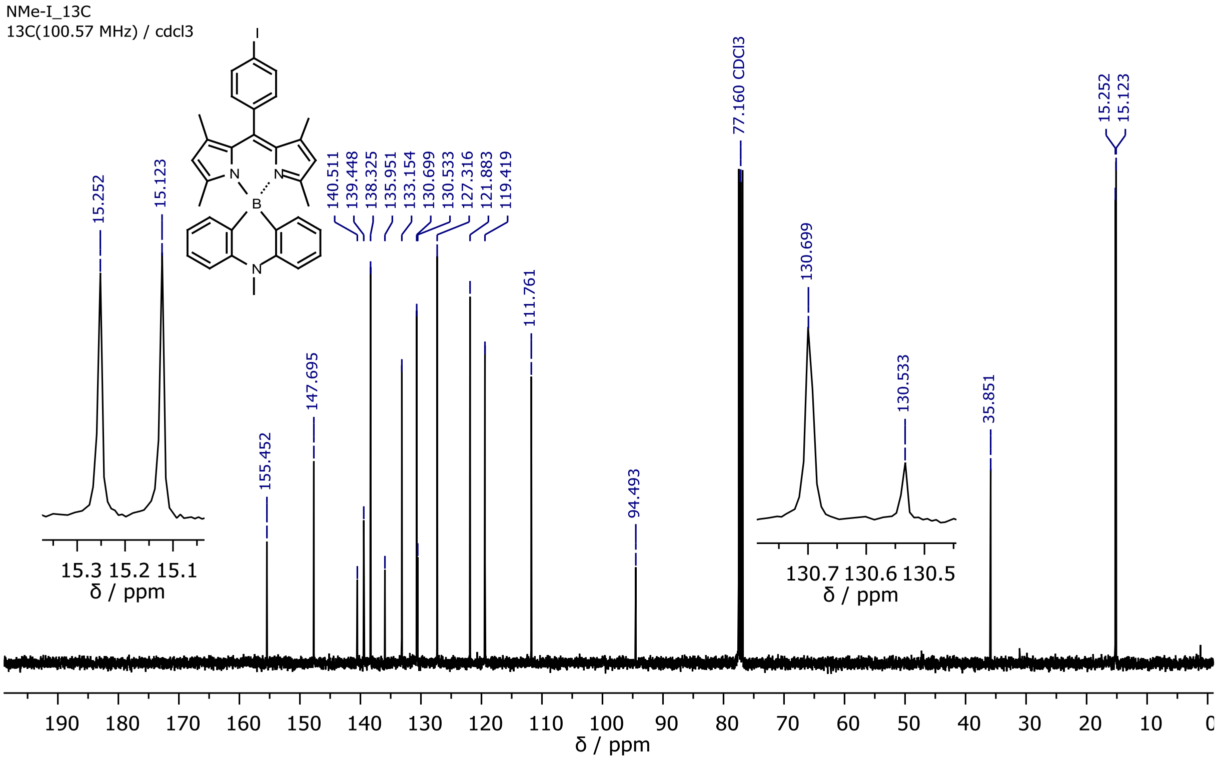

Figure S116. ^{13}C NMR spectrum of **NMe-BDP-I** (101 MHz, $CDCl_3$).

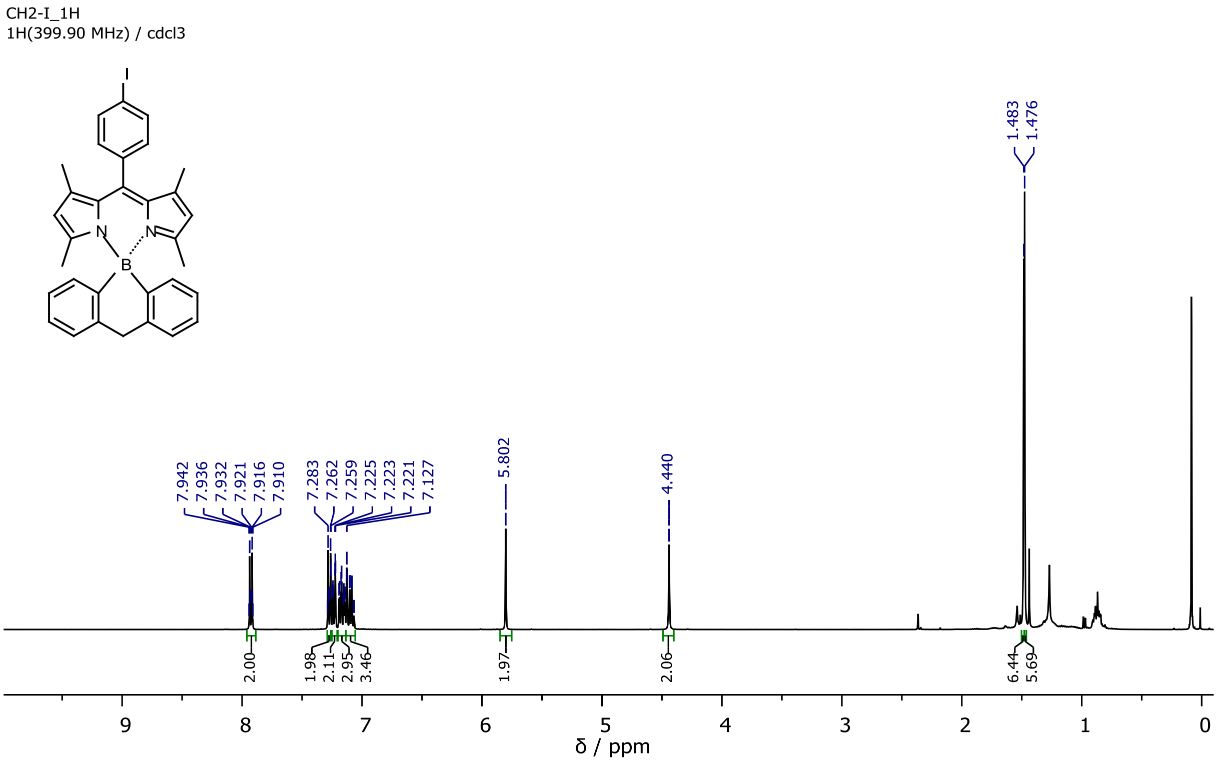

Figure S117. ^{1}H NMR spectrum of **CH2-BDP-I** (400 MHz, $CDCl_3$).

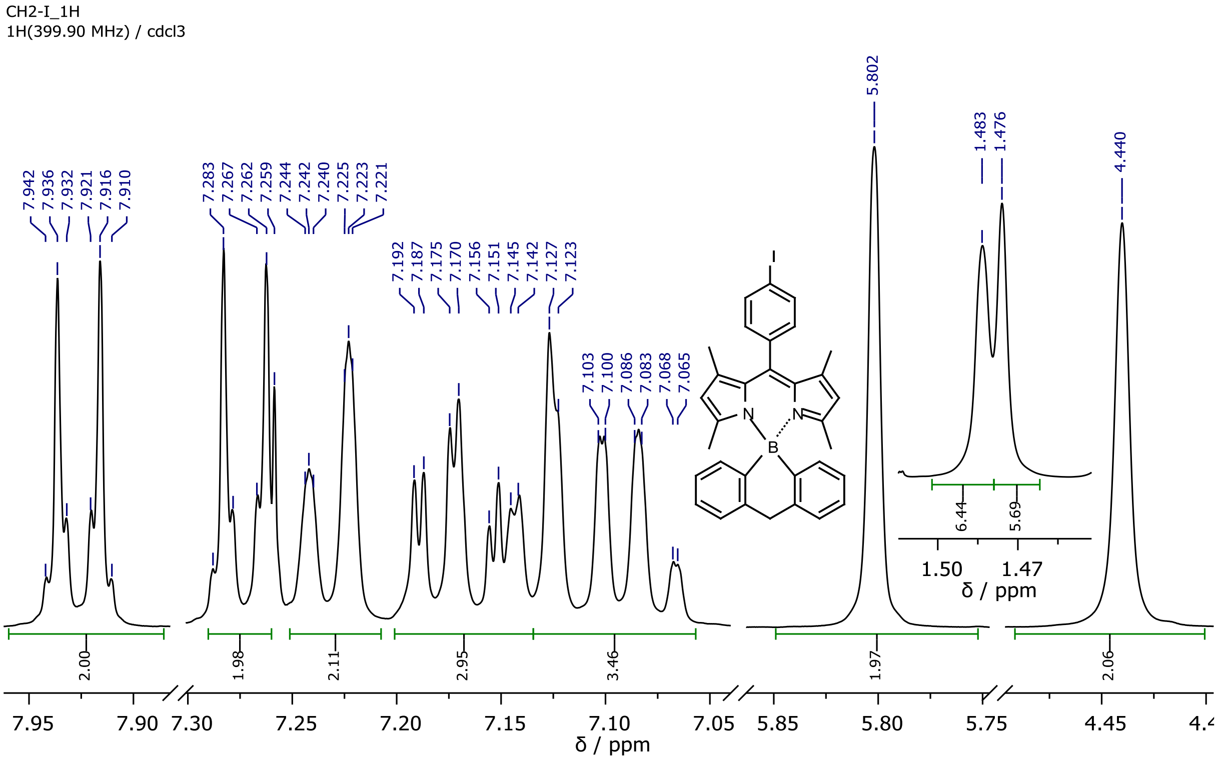

Figure S118. ^{1}H NMR spectrum of **CH2-BDP-I** (400 MHz, $CDCl_3$) – zoom.

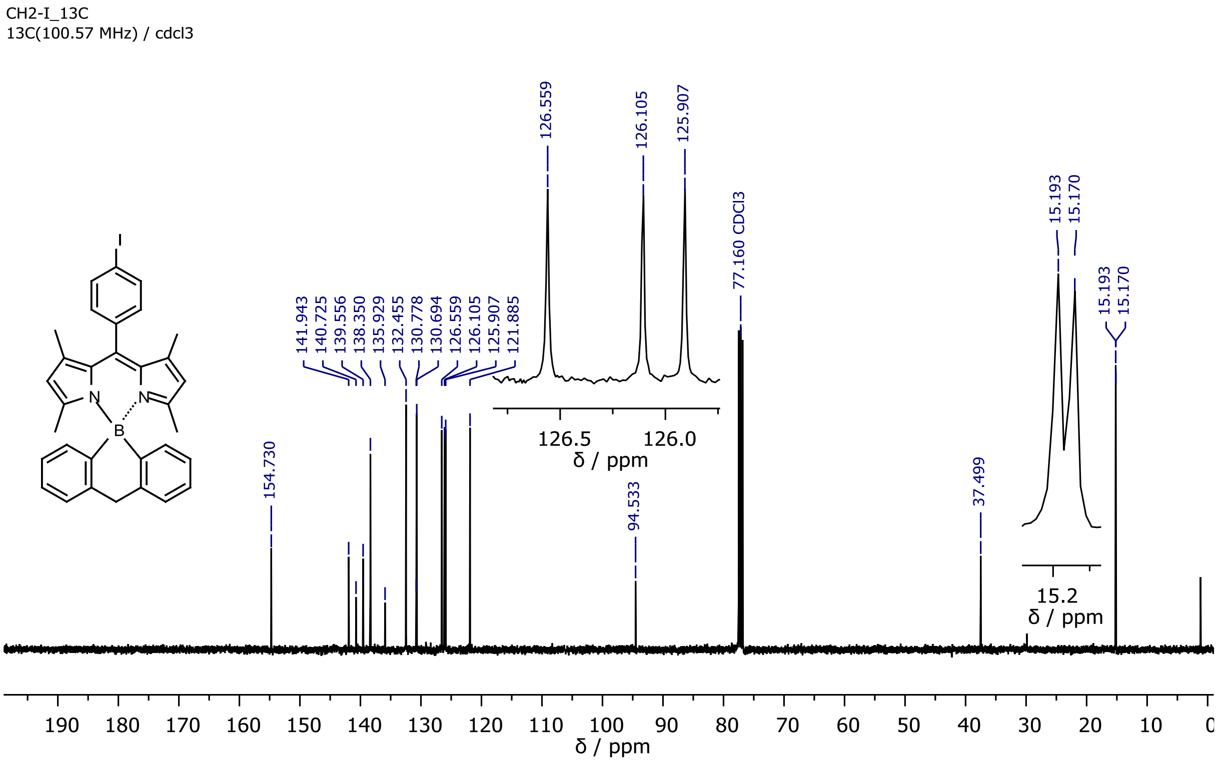

Figure S119. ^{13}C NMR spectrum of **CH2-BDP-I** (101 MHz, $CDCl_3$).

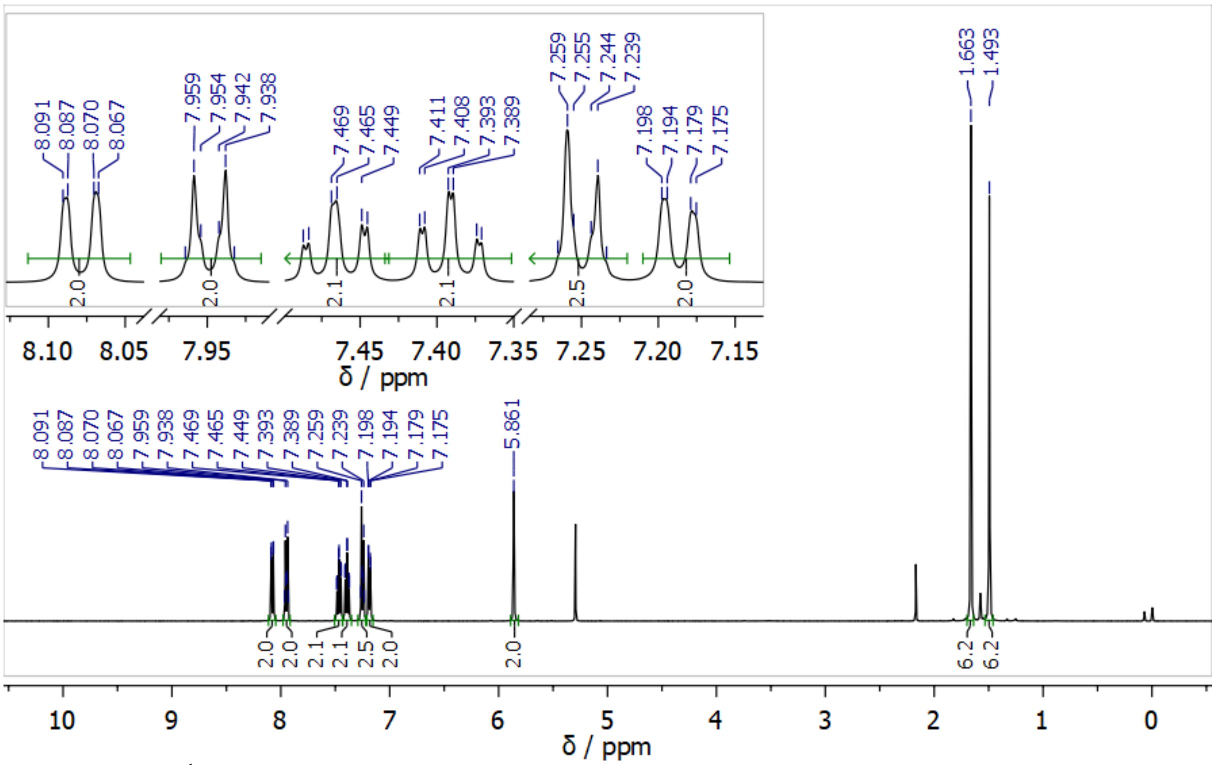

Figure S120. ^{1}H NMR spectrum of **SO2-BDP-I** (400 MHz, $CDCl_3$).

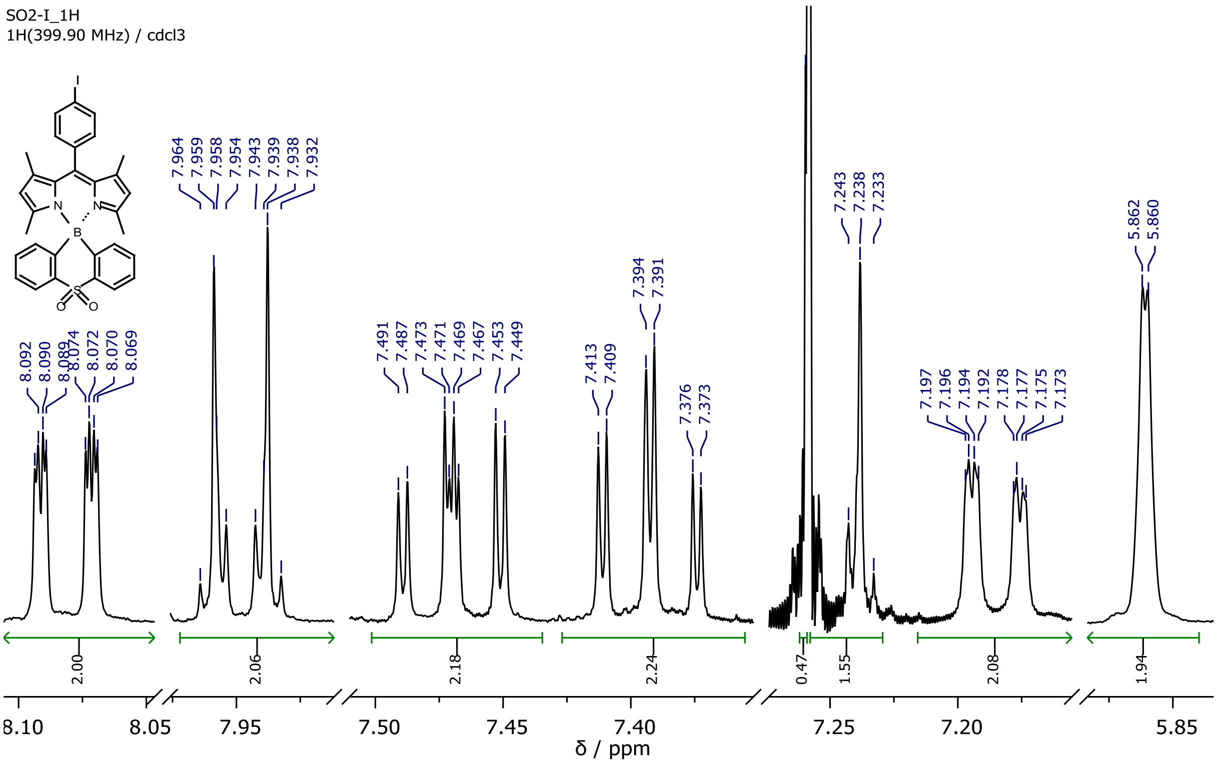

Figure S121. ^{1}H NMR spectrum of **SO2-BDP-I** (400 MHz, $CDCl_3$) – zoom.

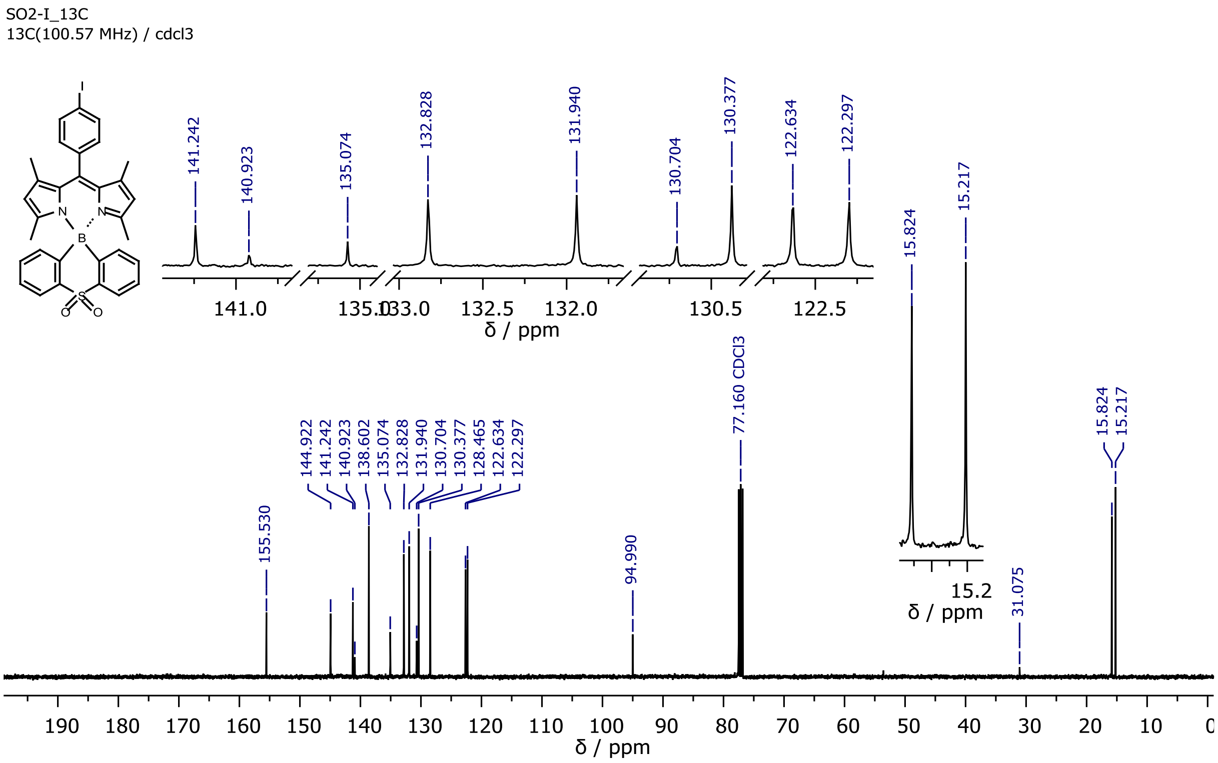

Figure S122. ^{13}C NMR spectrum of **SO2-BDP-I** (101 MHz, $CDCl_3$).

9.4 NMR spectra of X-BDP-CCCH2NMe2

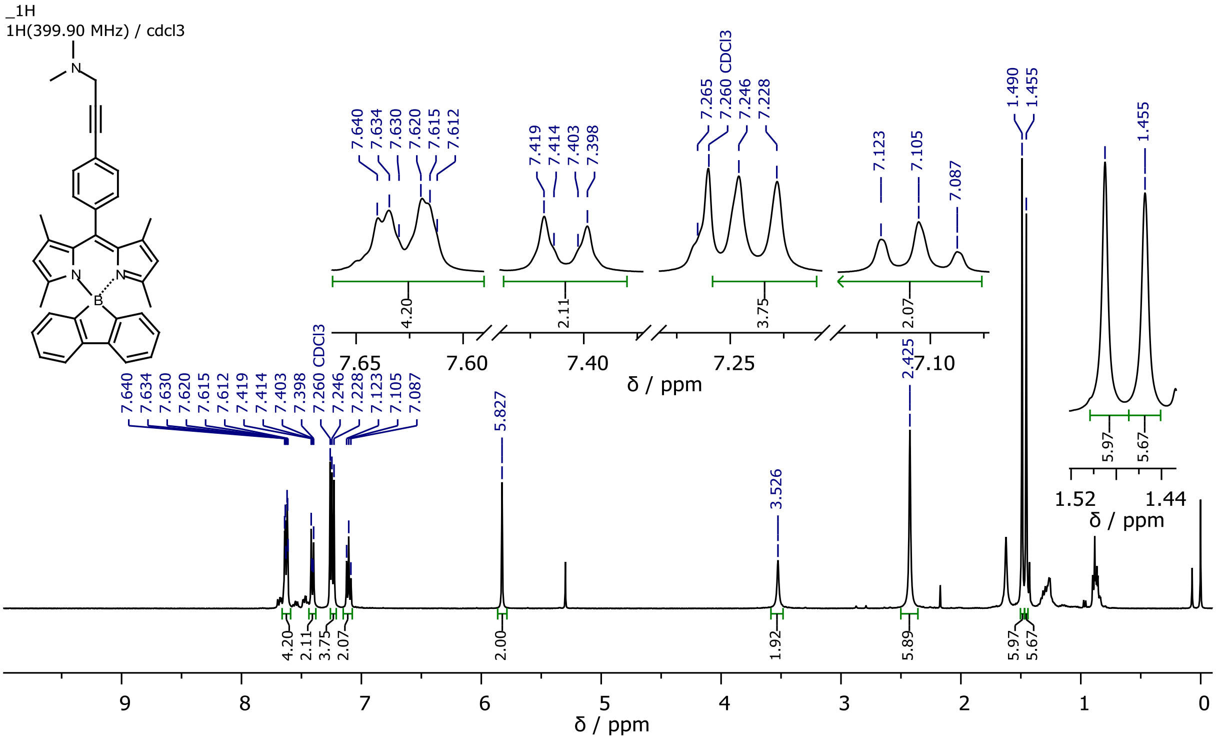

Figure S123. ^{1}H NMR spectrum of **Bf-BDP-CCCH2NMe2** (400 MHz, $CDCl_3$).

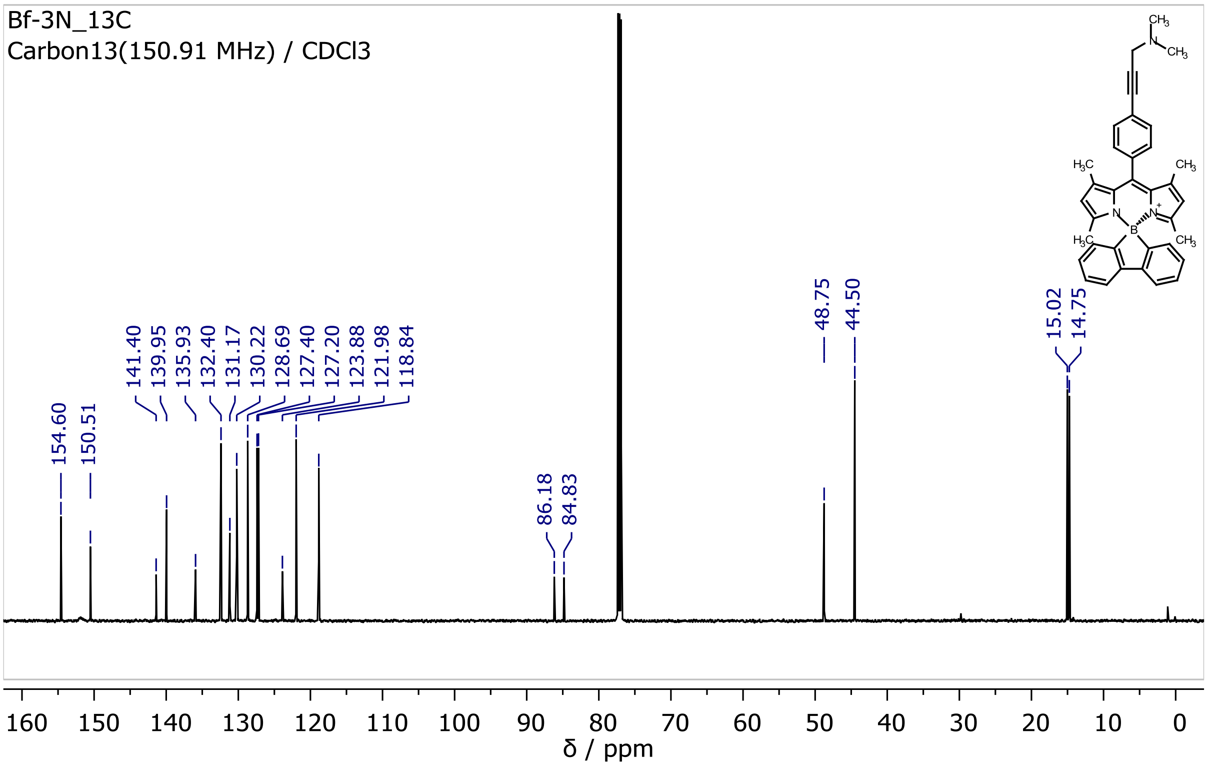

Figure S124. ^{13}C NMR spectrum of **Bf-BDP-CCCH2NMe2** (151 MHz, $CDCl_3$).

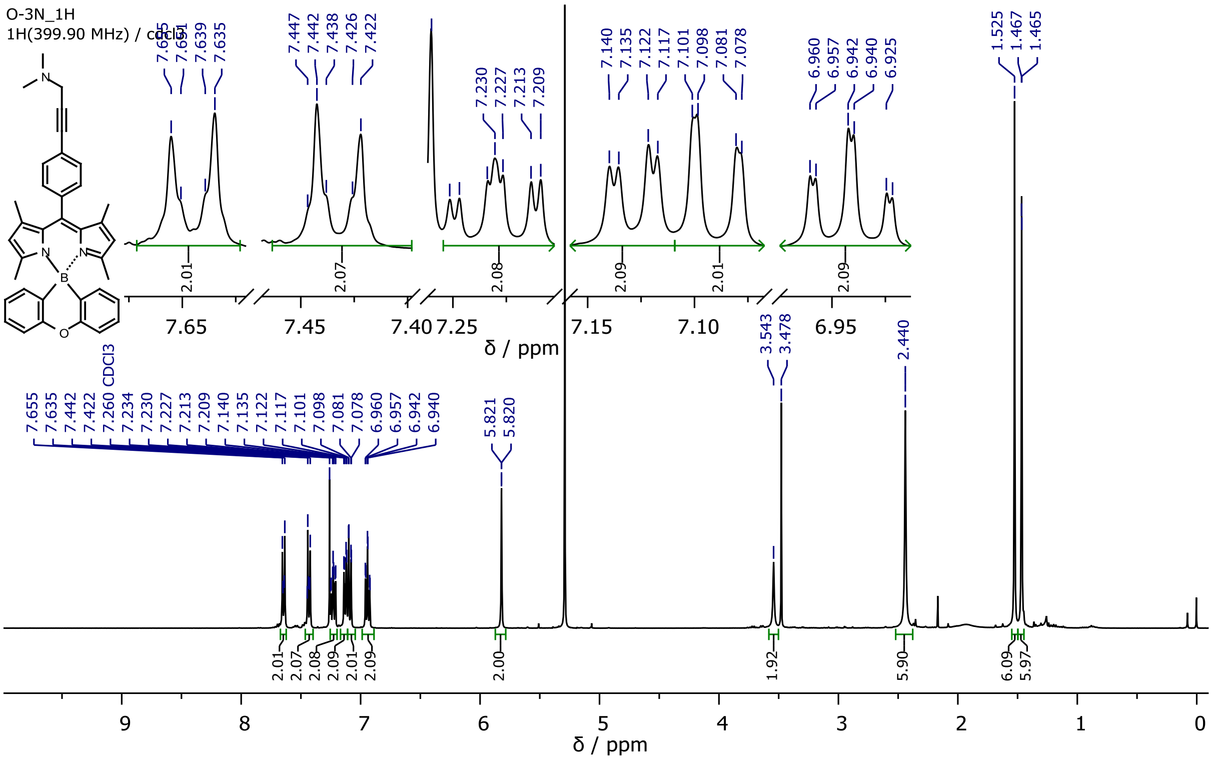

Figure S125. ^{1}H NMR spectrum of **O-BDP-CCCH2NMe2** (400 MHz, $CDCl_3$).

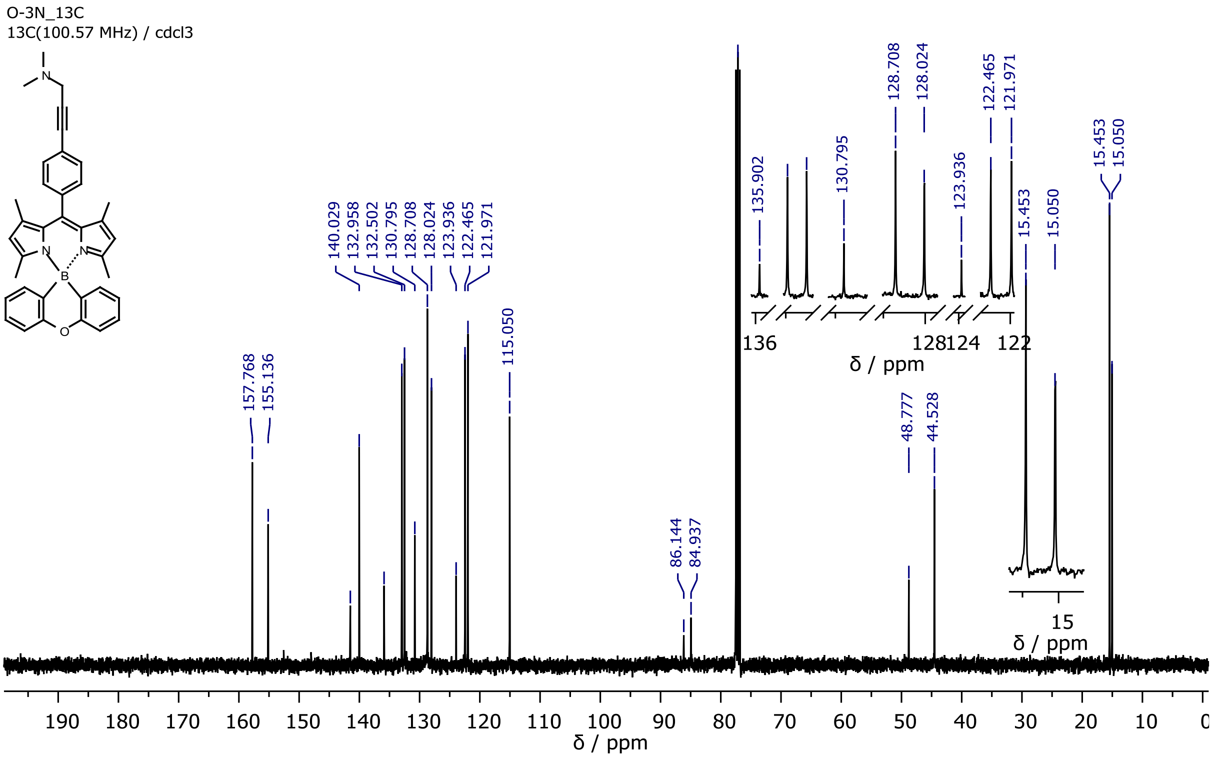

Figure S126. ^{13}C NMR spectrum of **O-BDP-CCCH2NMe2** (101 MHz, $CDCl_3$).

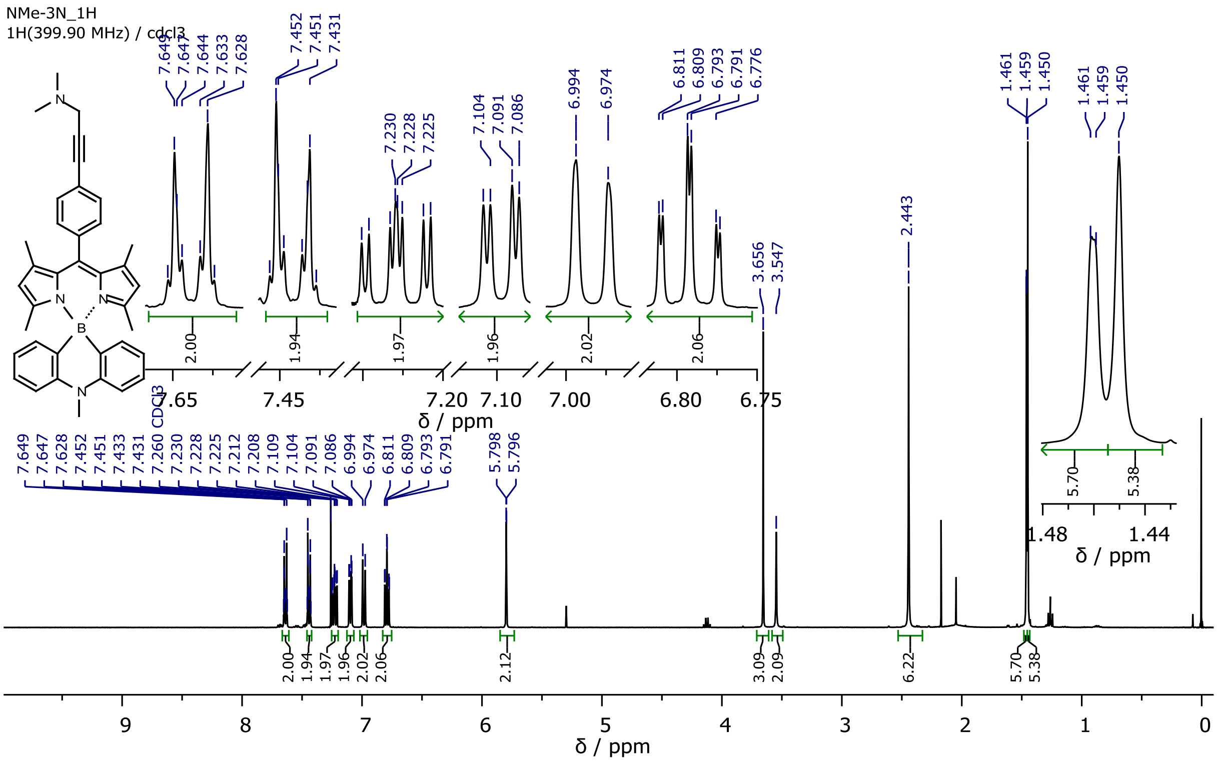

Figure S127. ^{1}H NMR spectrum of **NMe-BDP-CCCH2NMe2** (400 MHz, $CDCl_3$).

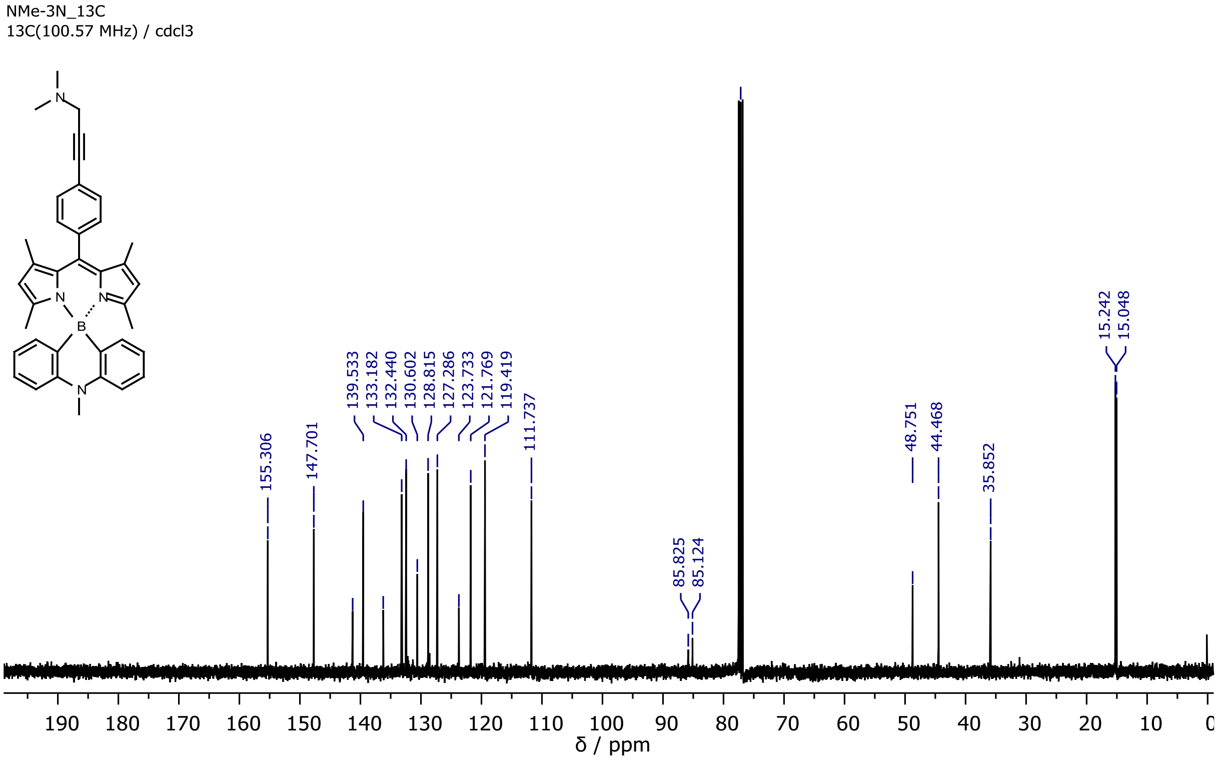

Figure S128. ^{13}C NMR spectrum of **NMe-BDP-CCCH2NMe2** (101 MHz, $CDCl_3$).

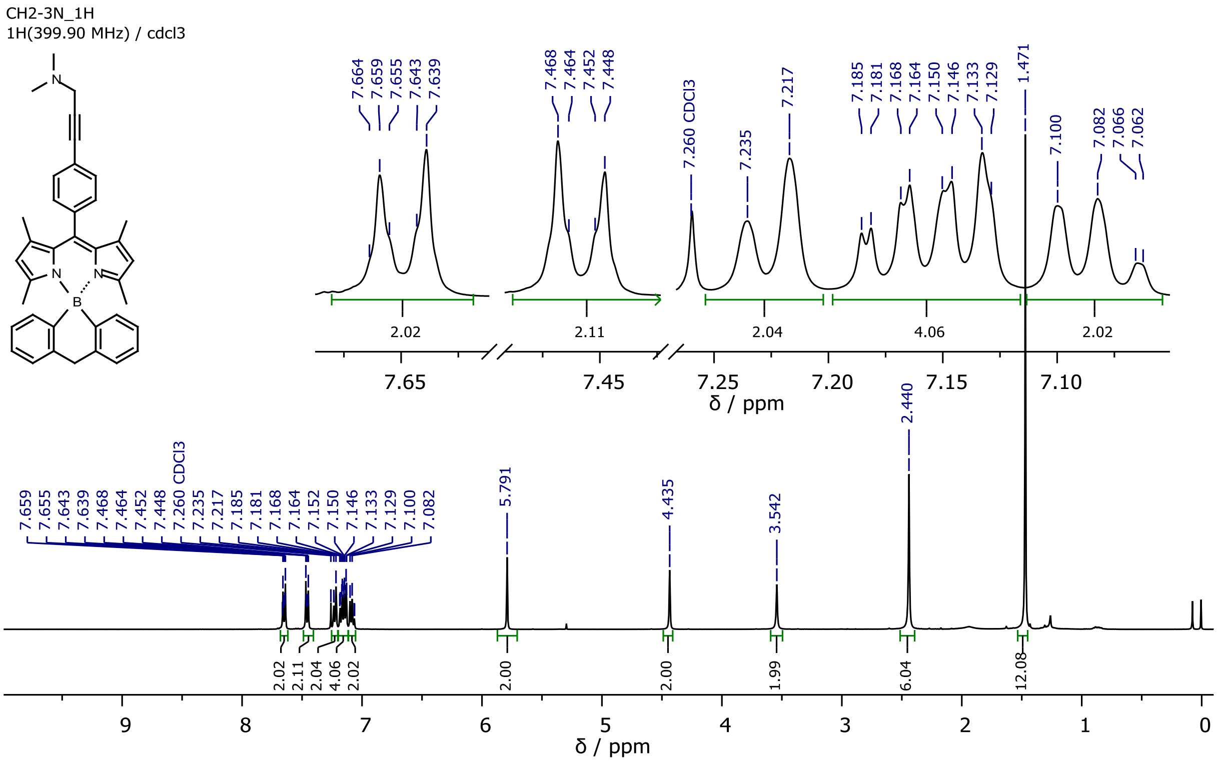

Figure S129. ^{1}H NMR spectrum of **CH2-BDP-CCCH2NMe2** (400 MHz, $CDCl_3$).

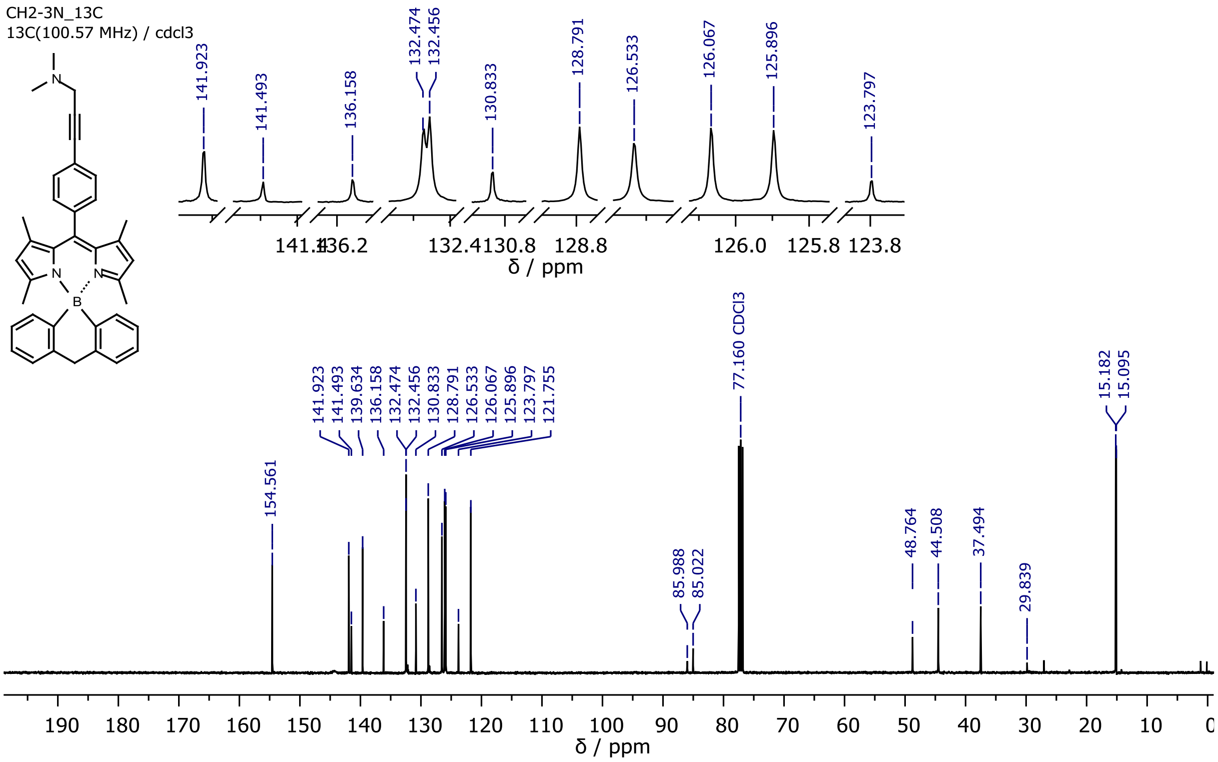

Figure S130. ^{13}C NMR spectrum of **CH2-BDP-CCCH2NMe2** (101 MHz, $CDCl_3$).

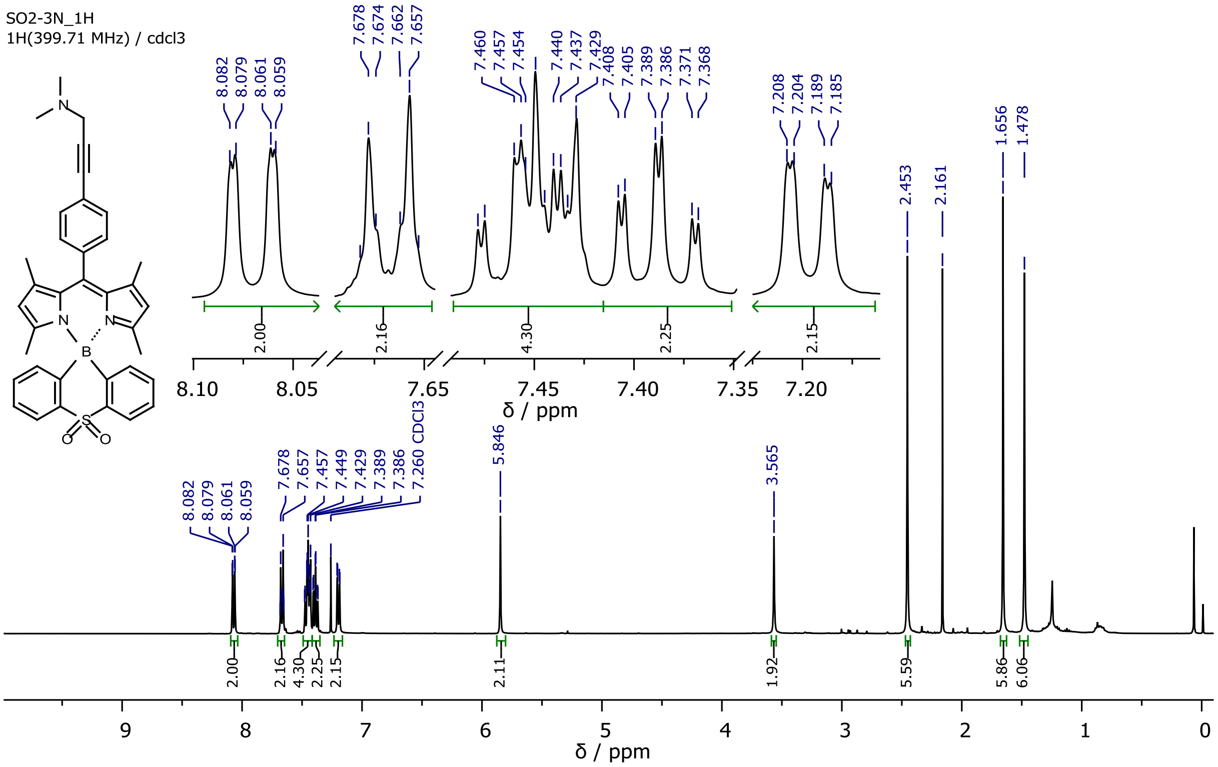

Figure S131. ^{1}H NMR spectrum of **SO2-BDP-CCCH2NMe2** (400 MHz, $CDCl_3$).

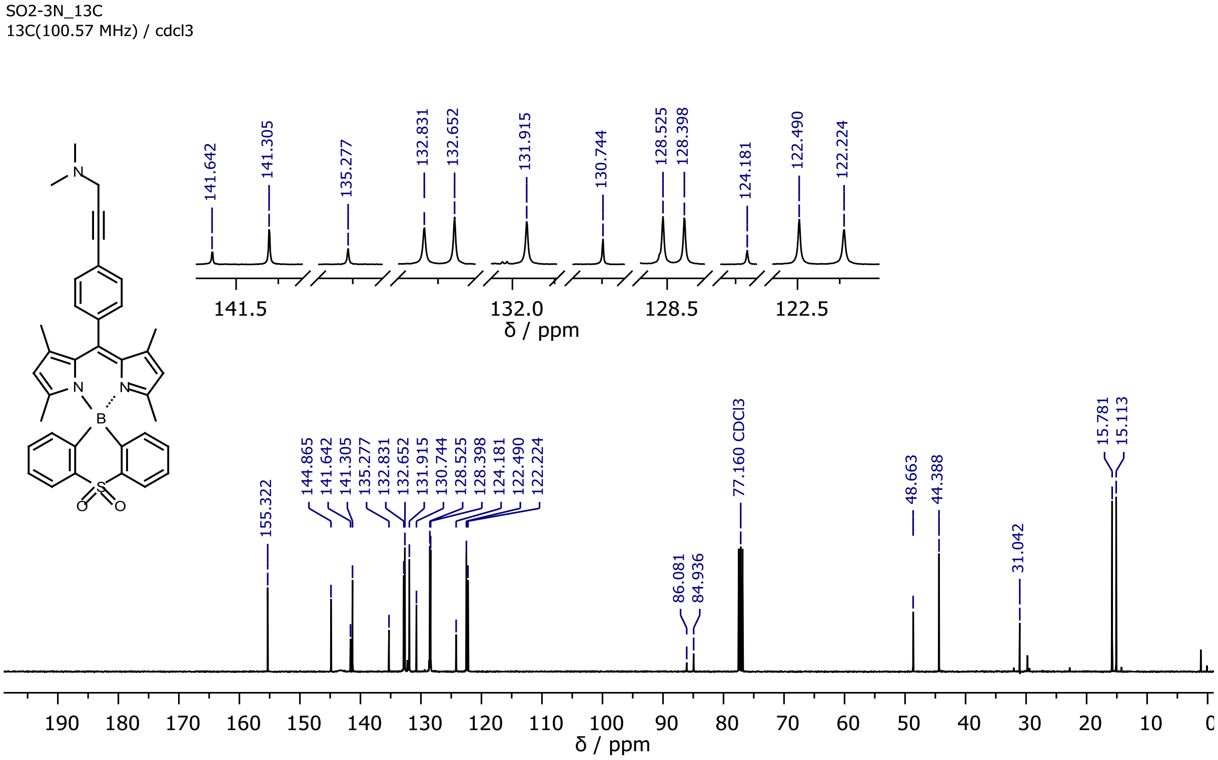

Figure S132. ^{13}C NMR spectrum of **SO2-Bf-BDP-CCCH2NMe2** (101 MHz, $CDCl_3$).

9.5 NMR spectra of X-BDP-ZWIT

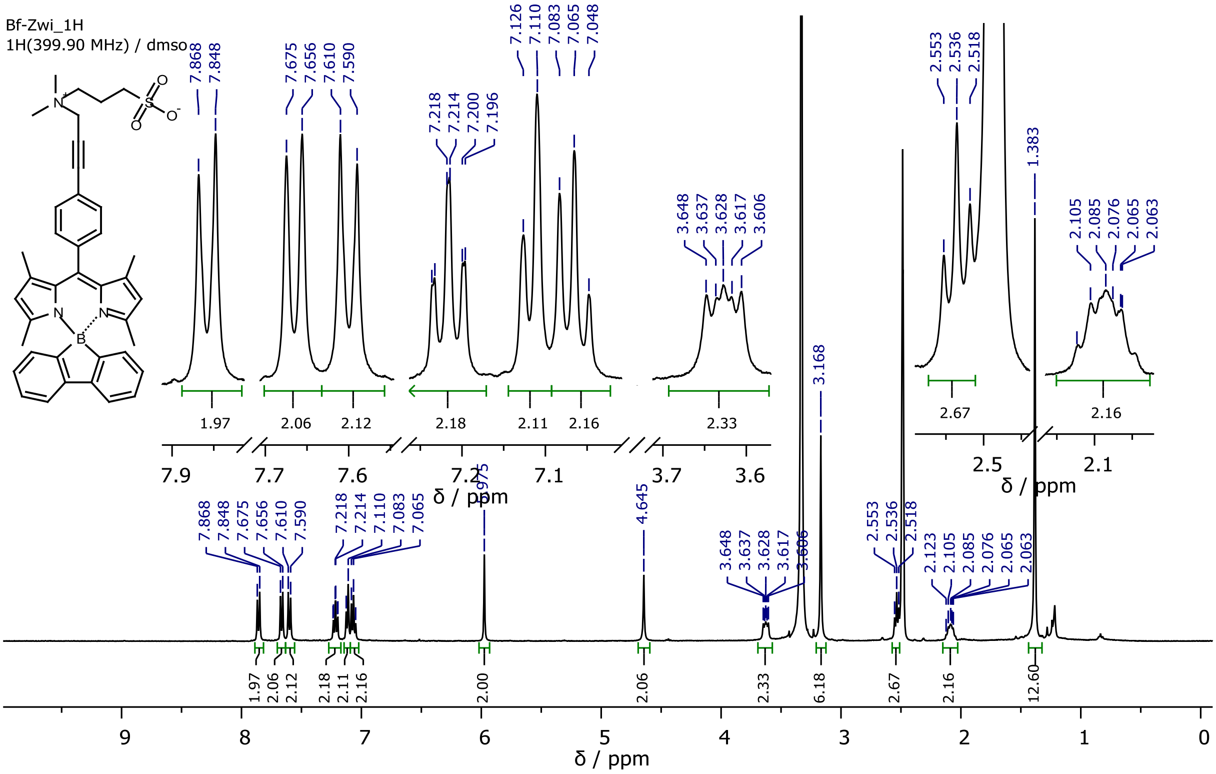

Figure S133. ^{1}H NMR spectrum of **Bf-BDP-ZWIT** (400 MHz, $CDCl_3$).

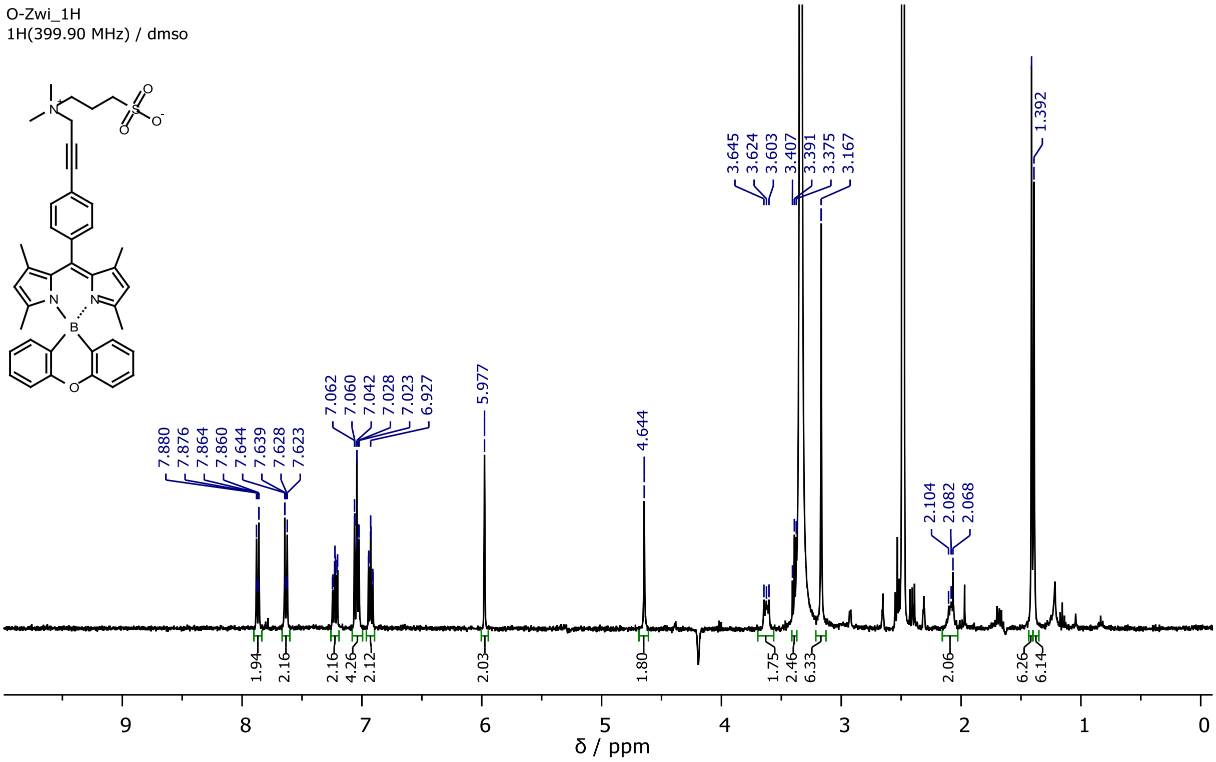

Figure S134. ^{1}H NMR spectrum of **O-BDP-ZWIT** (400 MHz, DMSO-d_6).

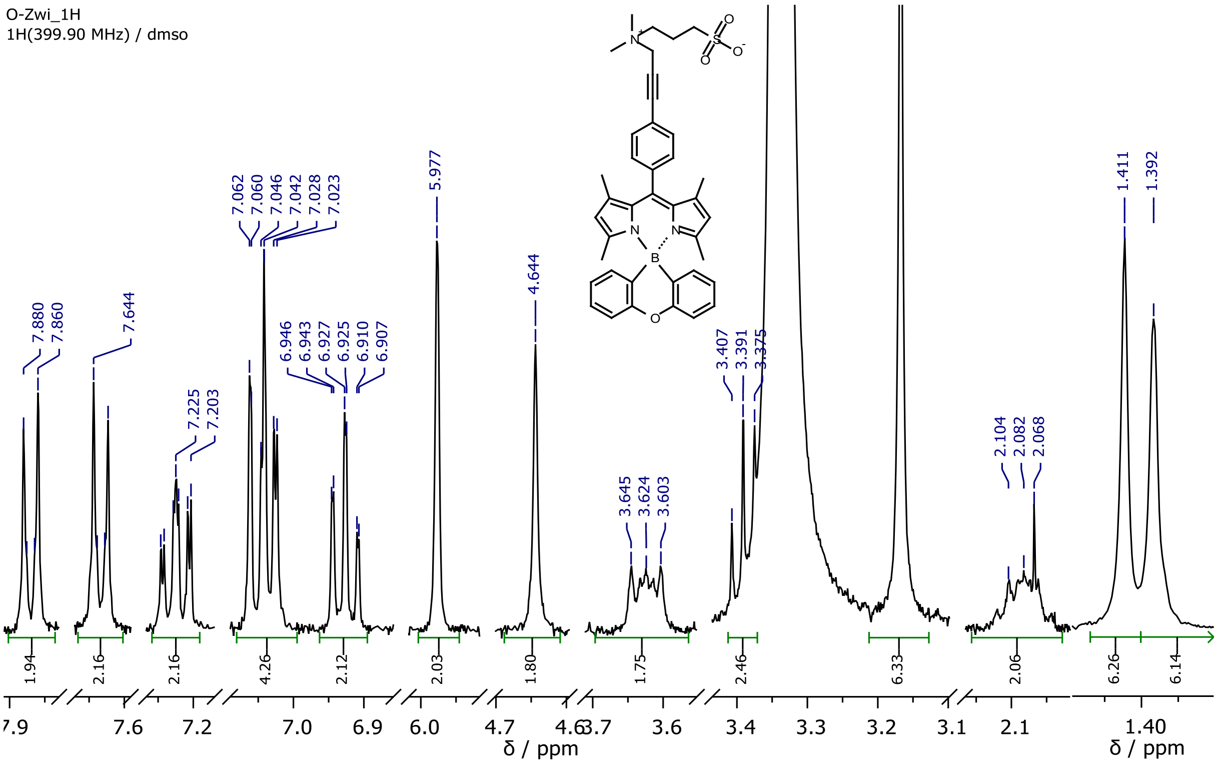

Figure S135. ^{1}H NMR spectrum of **O-BDP-ZWIT** (400 MHz, DMSO-d_6) – zoom.

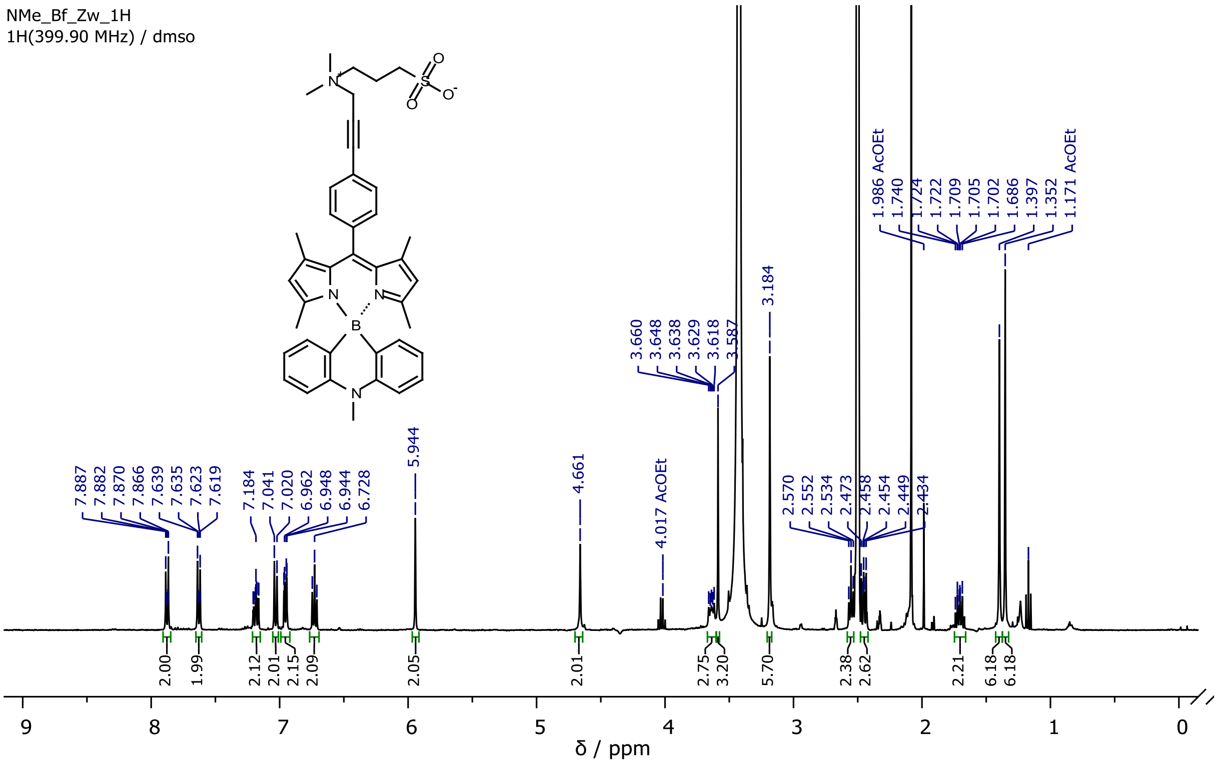

Figure S136. ^{1}H NMR spectrum of **NMe-BDP-ZWIT** (400 MHz, DMSO-d_6).

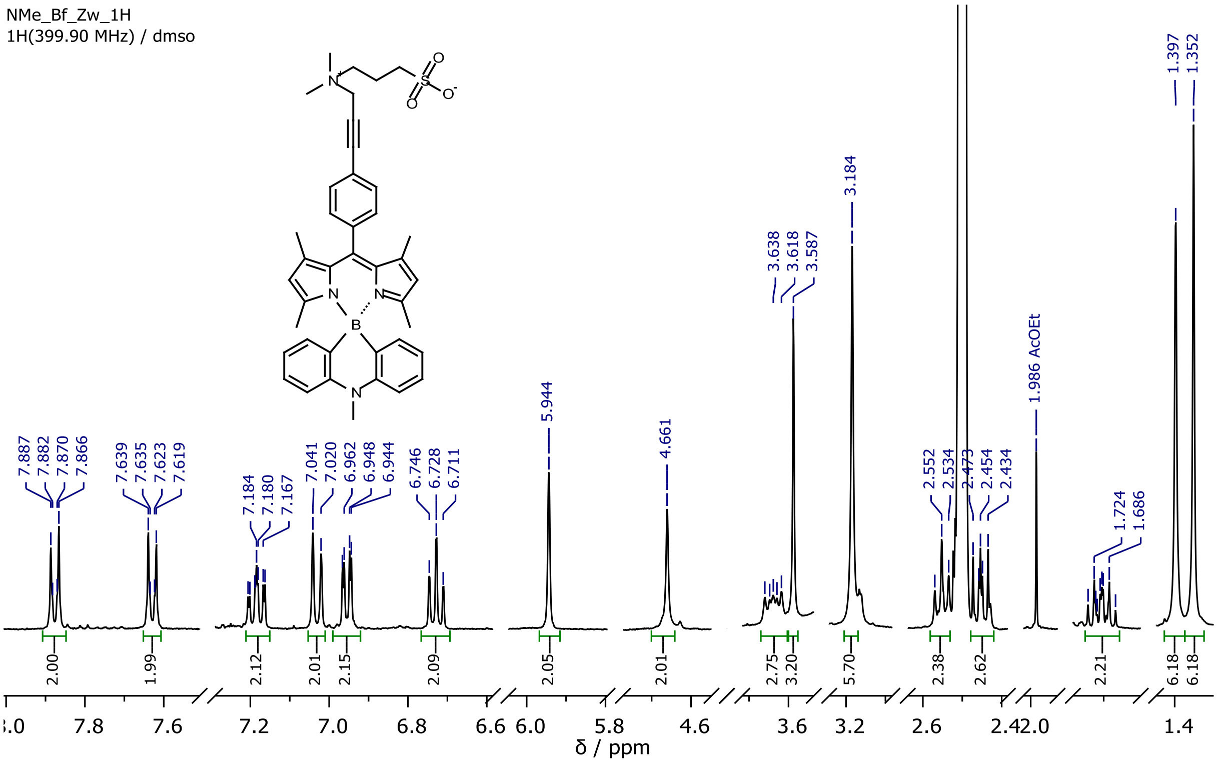

Figure S137. ^{1}H NMR spectrum of **NMe-BDP-ZWIT** (400 MHz, DMSO-d_6) -zoom.

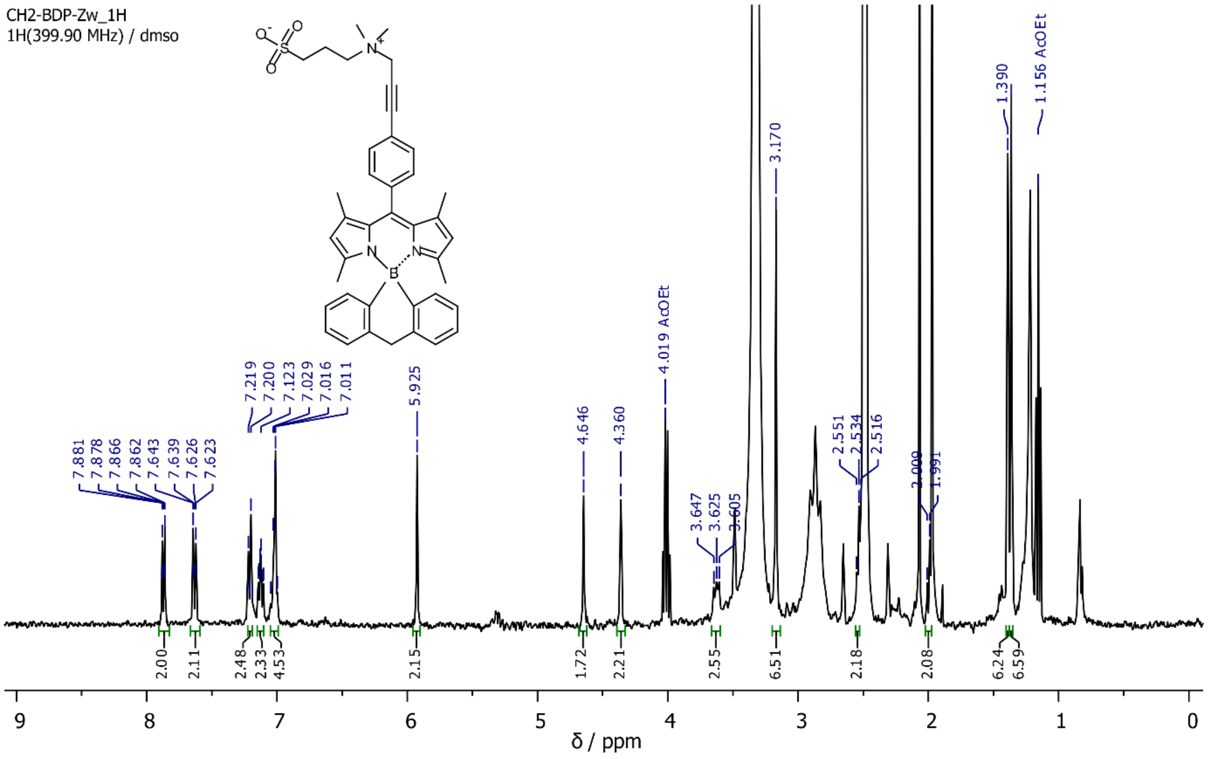

Figure S138. ^{1}H NMR spectrum of **CH$_2$-BDP-ZWIT** (400 MHz, DMSO-d_6).

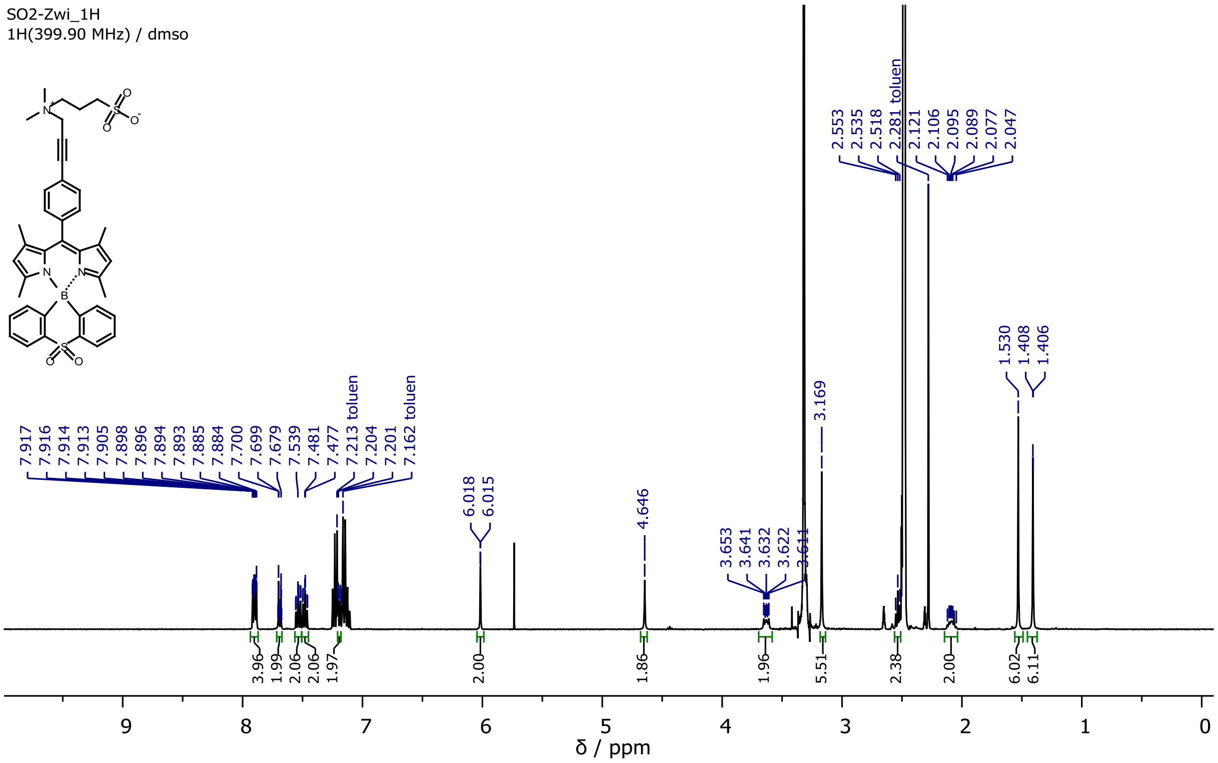

Figure S139. ^{1}H NMR spectrum of **SO$_2$-BDP-ZWIT** (400 MHz, DMSO-d_6).

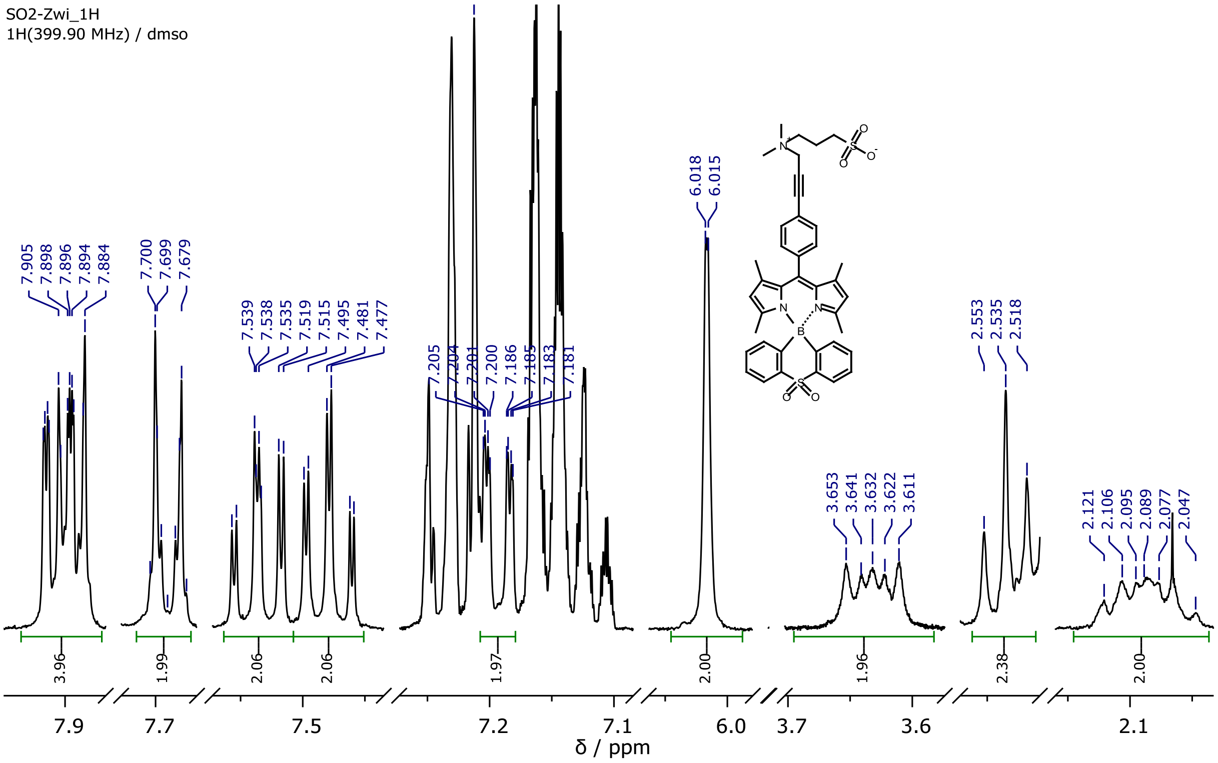

Figure S140. ^{1}H NMR spectrum of **SO$_2$-BDP-ZWIT** (400 MHz, DMSO-d_6) - zoom.

9.6 NMR spectra of X-BDP

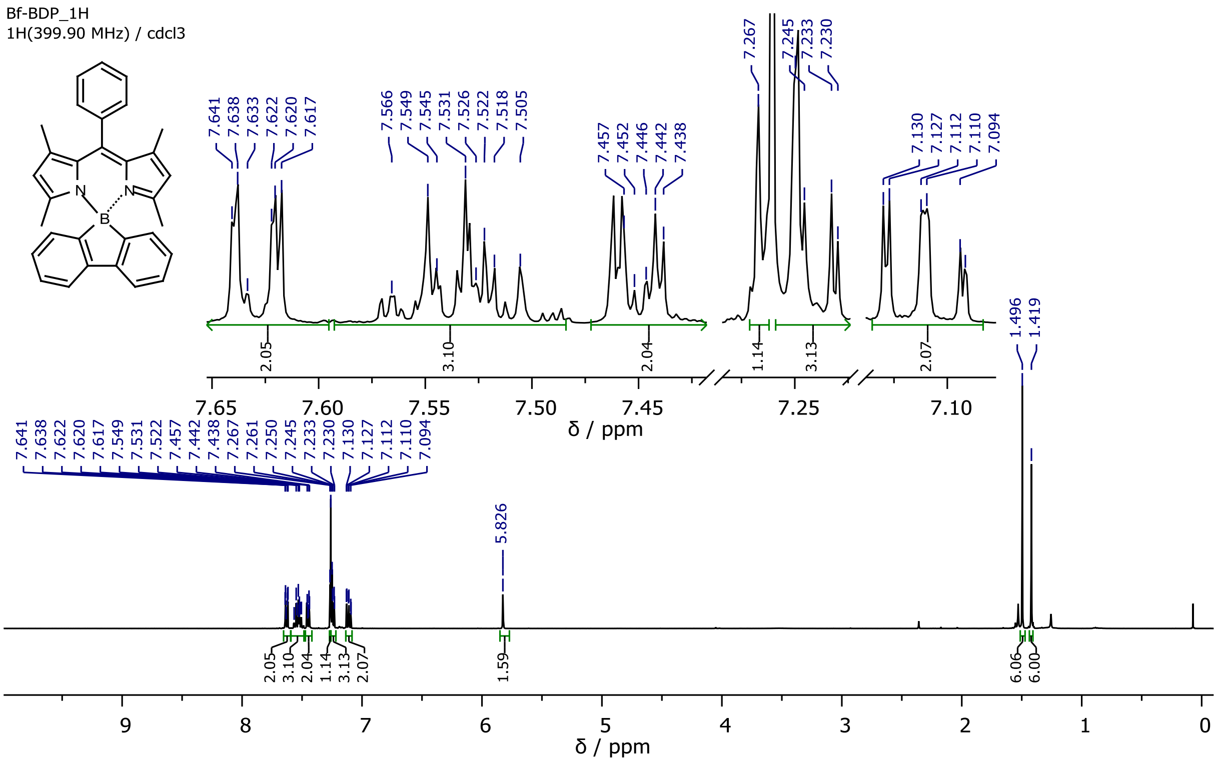

Figure S141. ^{1}H NMR spectrum of **Bf-BDP** (400 MHz, $CDCl_3$).

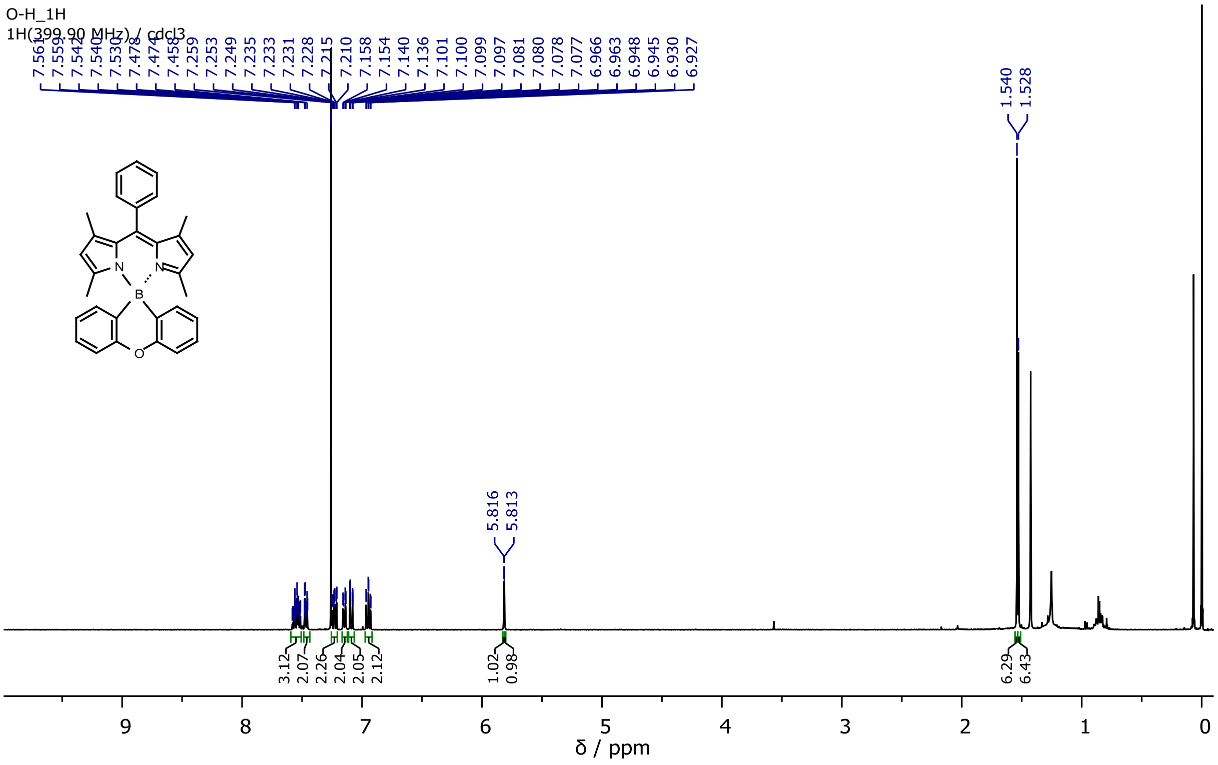

Figure S142. ^{1}H NMR spectrum of **O-BDP** (400 MHz, $CDCl_3$).

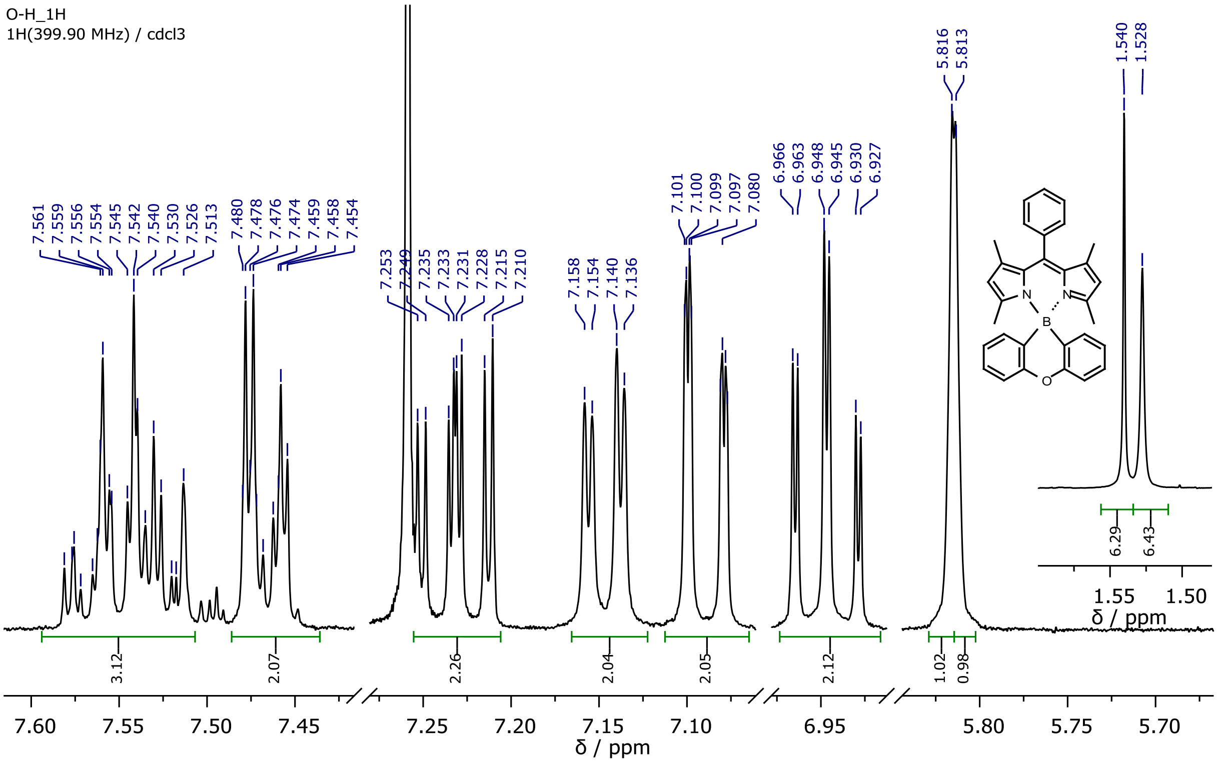

Figure S143. ^{1}H NMR spectrum of **O-BDP** (400 MHz, $CDCl_3$) – zoom at aromatic region.

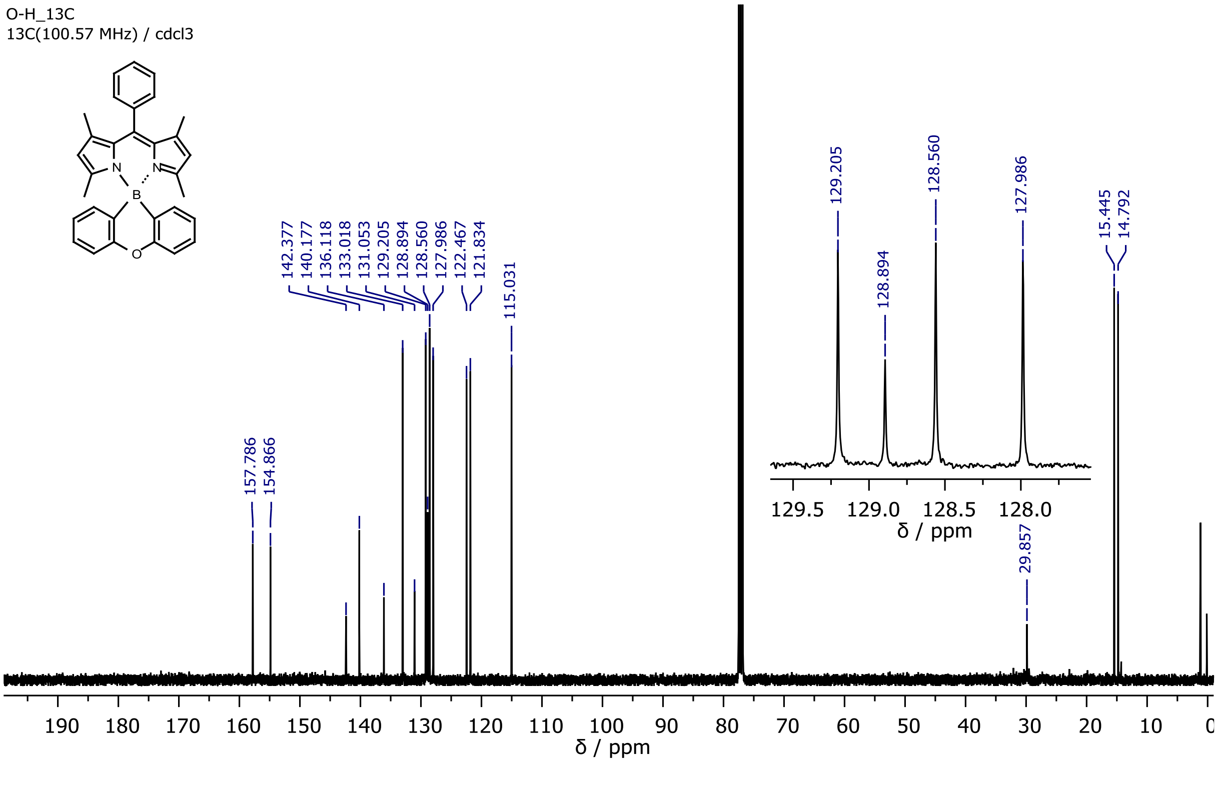

Figure S144. ^{13}C NMR spectrum of **O-BDP** (101 MHz, $CDCl_3$).

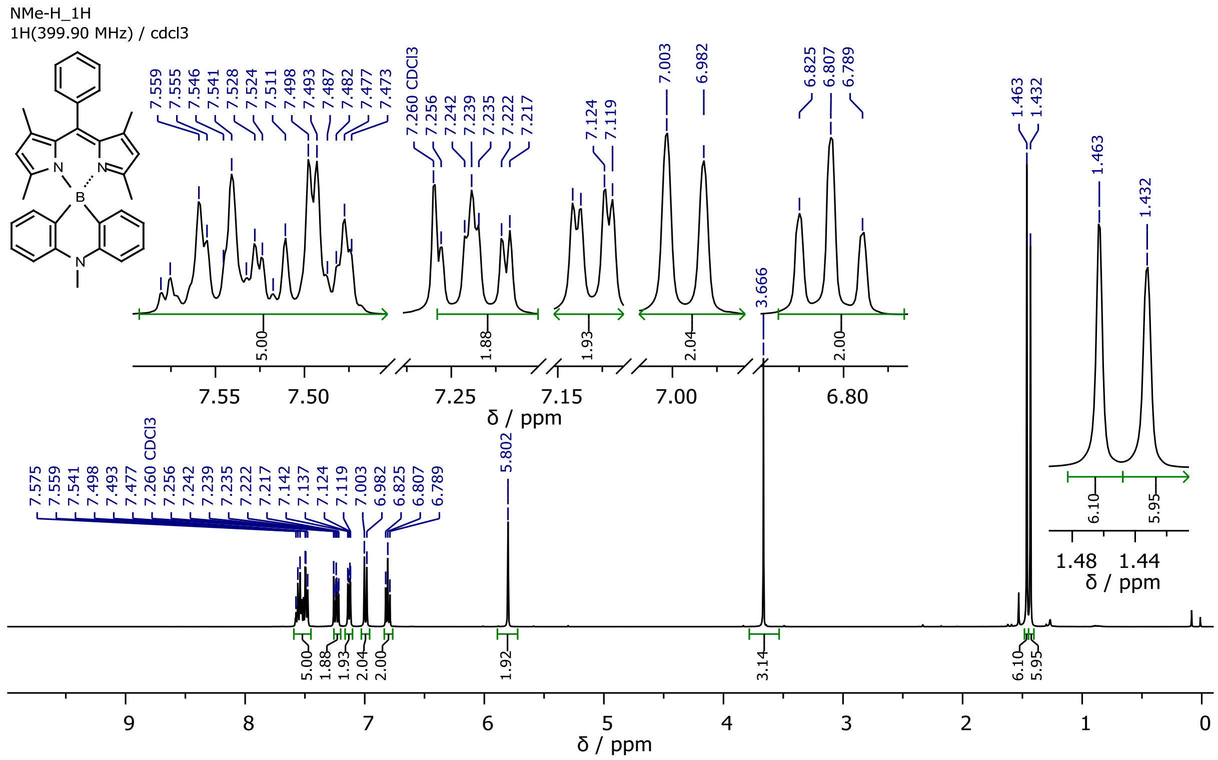

Figure S145. ^{1}H NMR spectrum of **NMe-BDP** (400 MHz, $CDCl_3$).

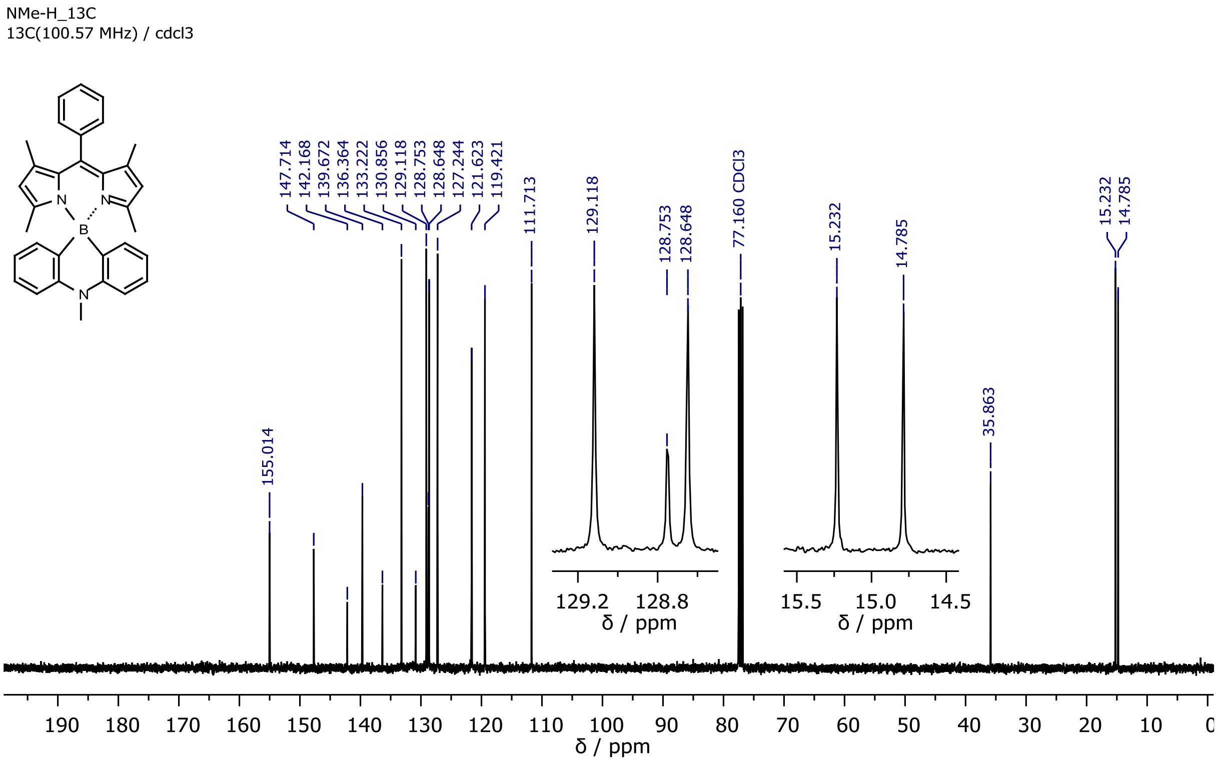

Figure S146. ^{13}C NMR spectrum of **NMe-BDP** (101 MHz, $CDCl_3$).

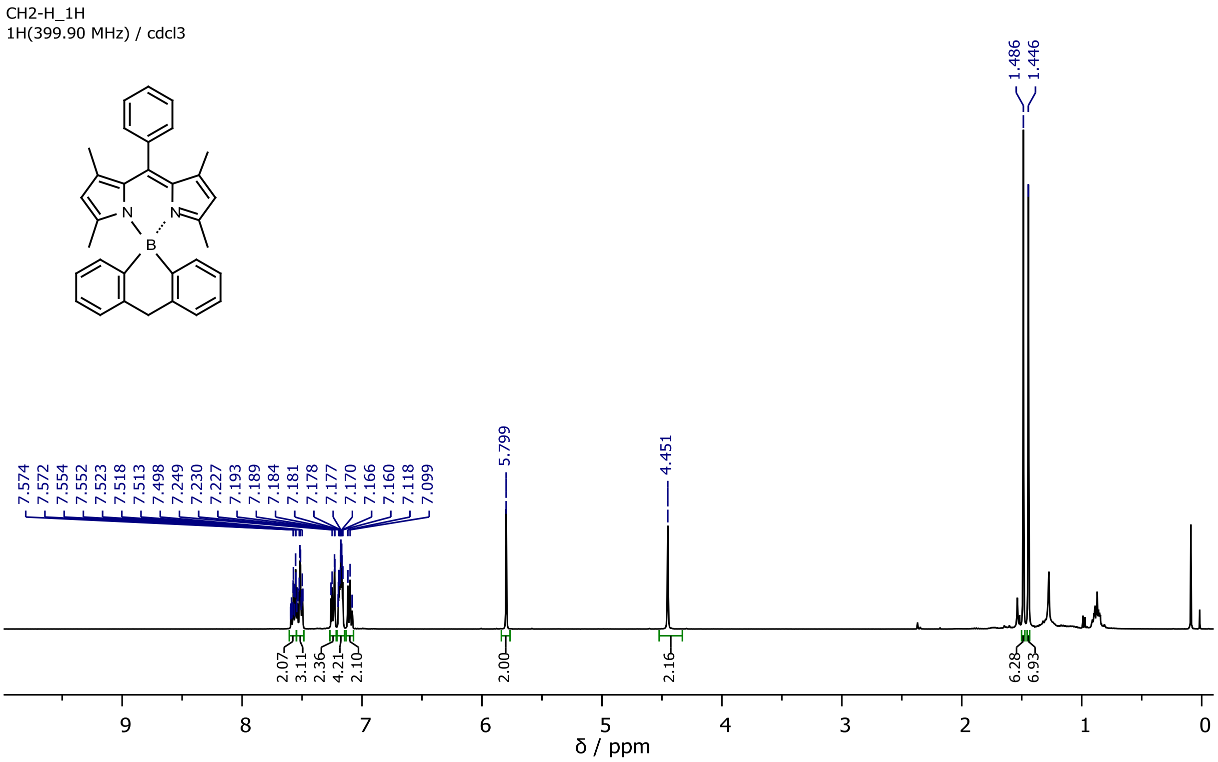

Figure S147. ^{1}H NMR spectrum of **CH_2-BDP** (400 MHz, $CDCl_3$).

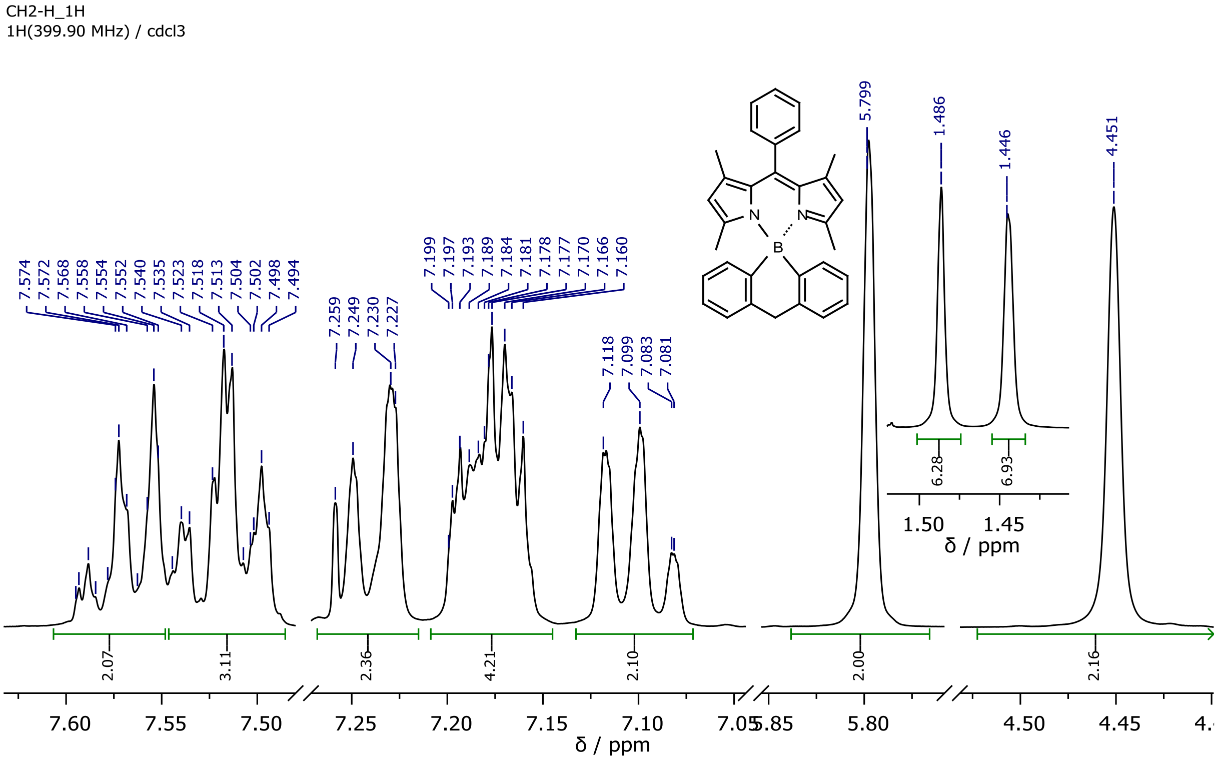

Figure S148. ^{1}H NMR spectrum of **CH_2-BDP** (400 MHz, $CDCl_3$) – zoom.

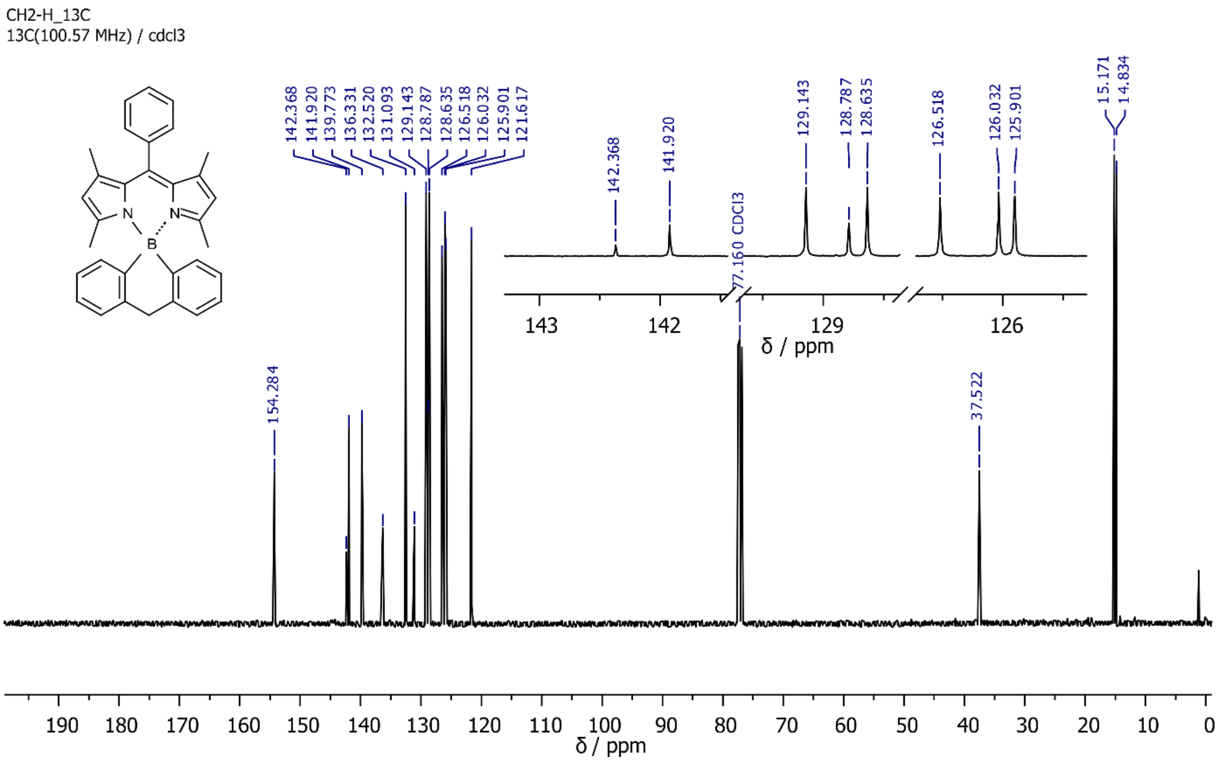

Figure S149. ^{13}C NMR spectrum of **CH_2-BDP** (101 MHz, $CDCl_3$).

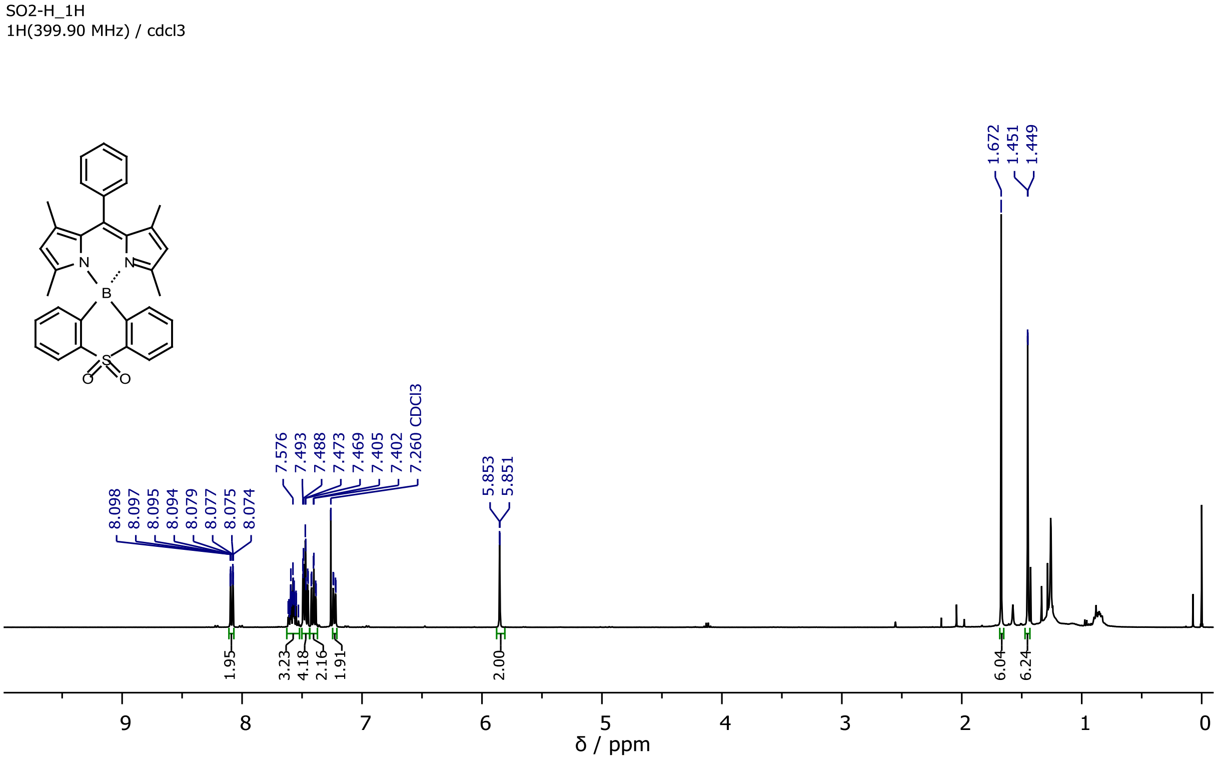

Figure S150. ^{1}H NMR spectrum of **SO_2-BDP** (400 MHz, $CDCl_3$).

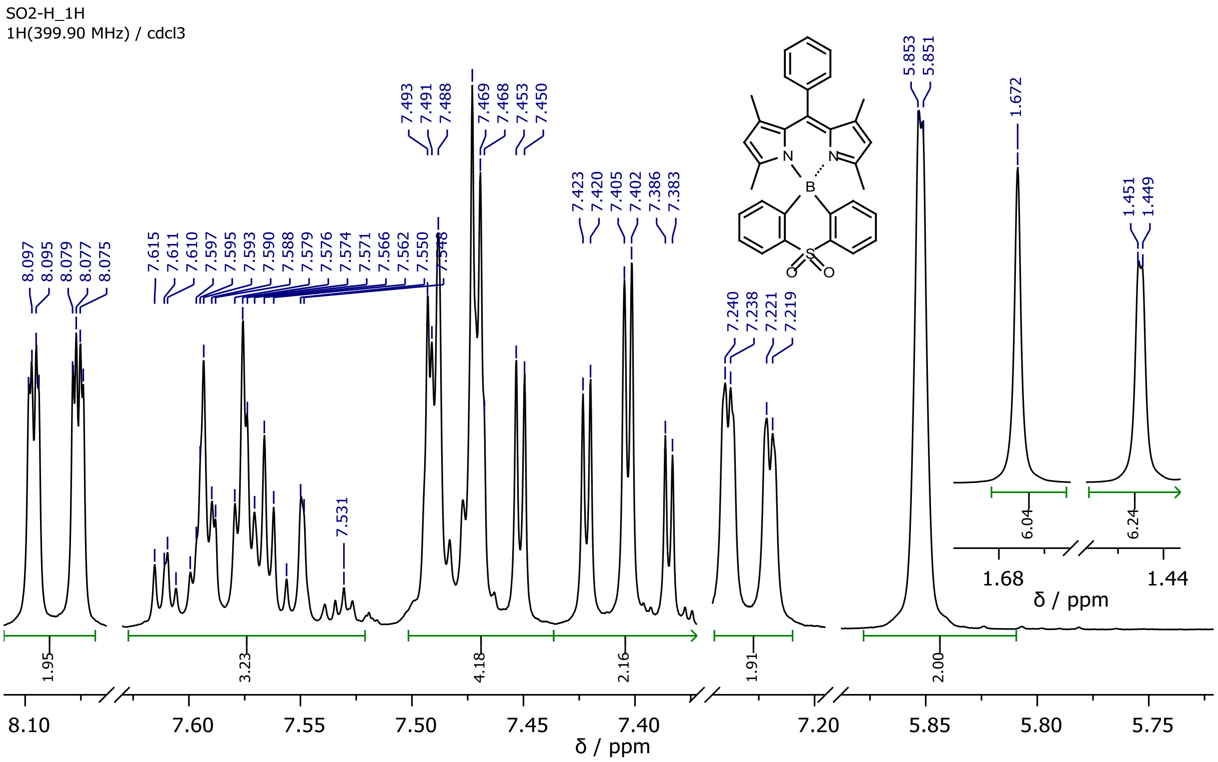

Figure S151. ^{1}H NMR spectrum of **SO$_2$-BDP** (400 MHz, $CDCl_3$) – zoom.

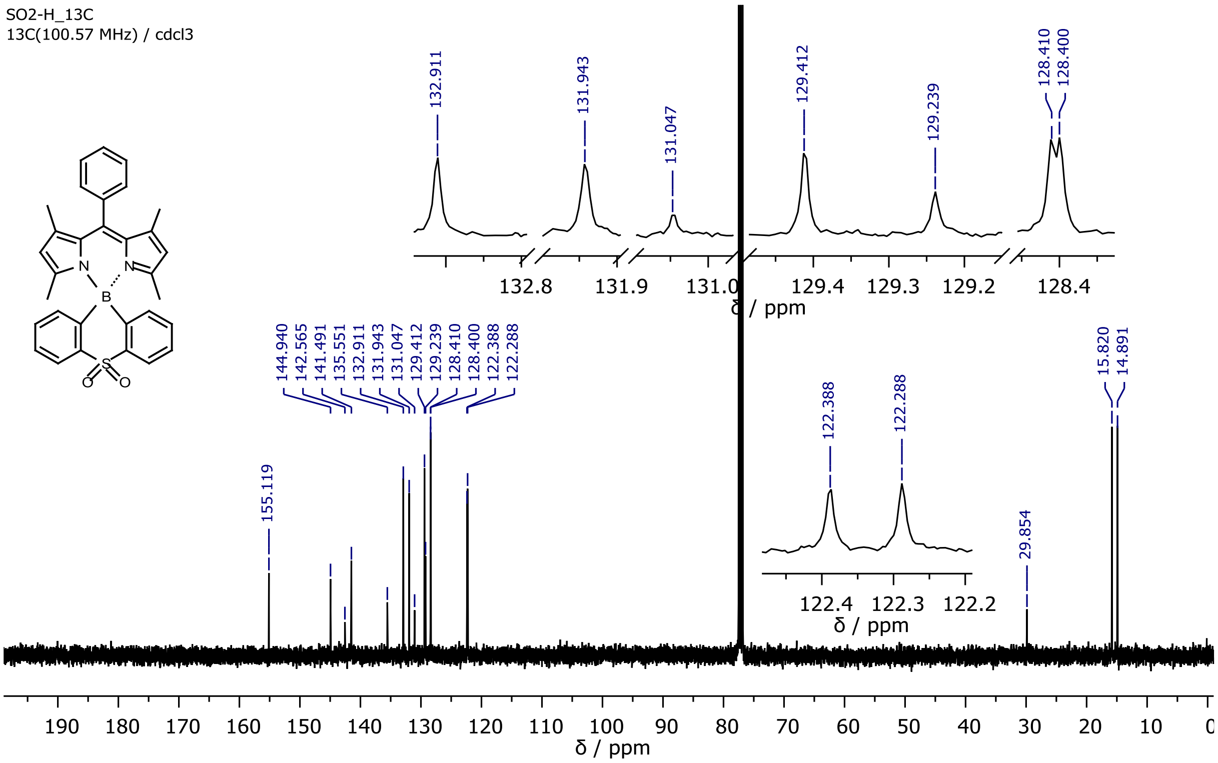

Figure S152. ^{13}C NMR spectrum of **SO$_2$-BDP** (101 MHz, $CDCl_3$).

9.7 NMR spectra of BF2-BDP-R references

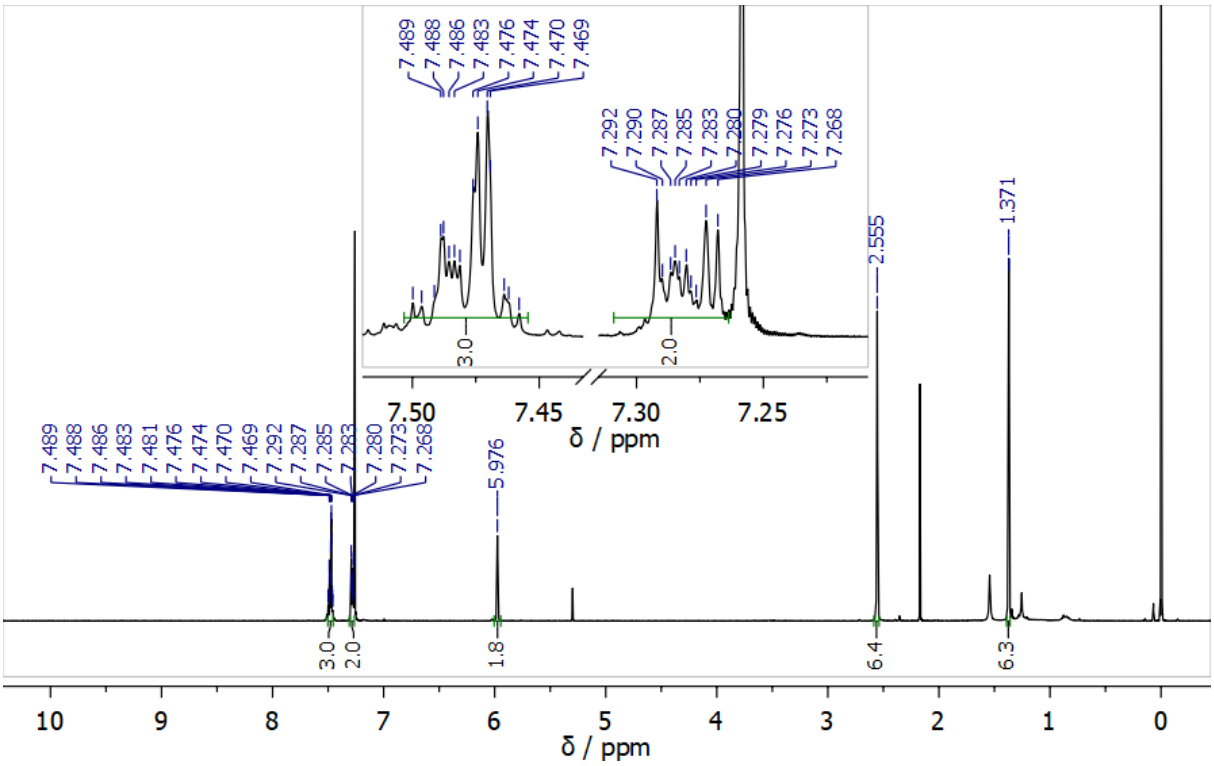

Figure S153. ^{1}H NMR spectrum of **BF$_2$-BDP** (400 MHz, $CDCl_3$).

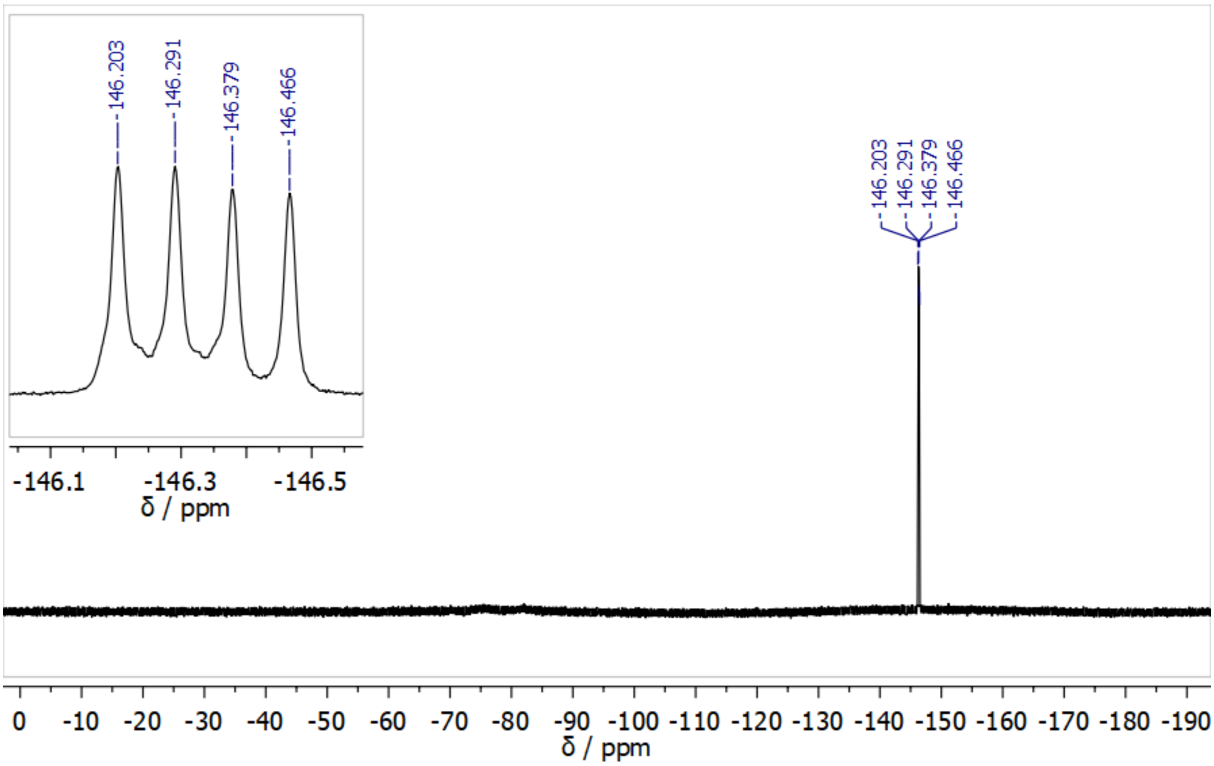

Figure S154. ^{19}F NMR spectrum of **BF$_2$-BDP** (376 MHz, $CDCl_3$).

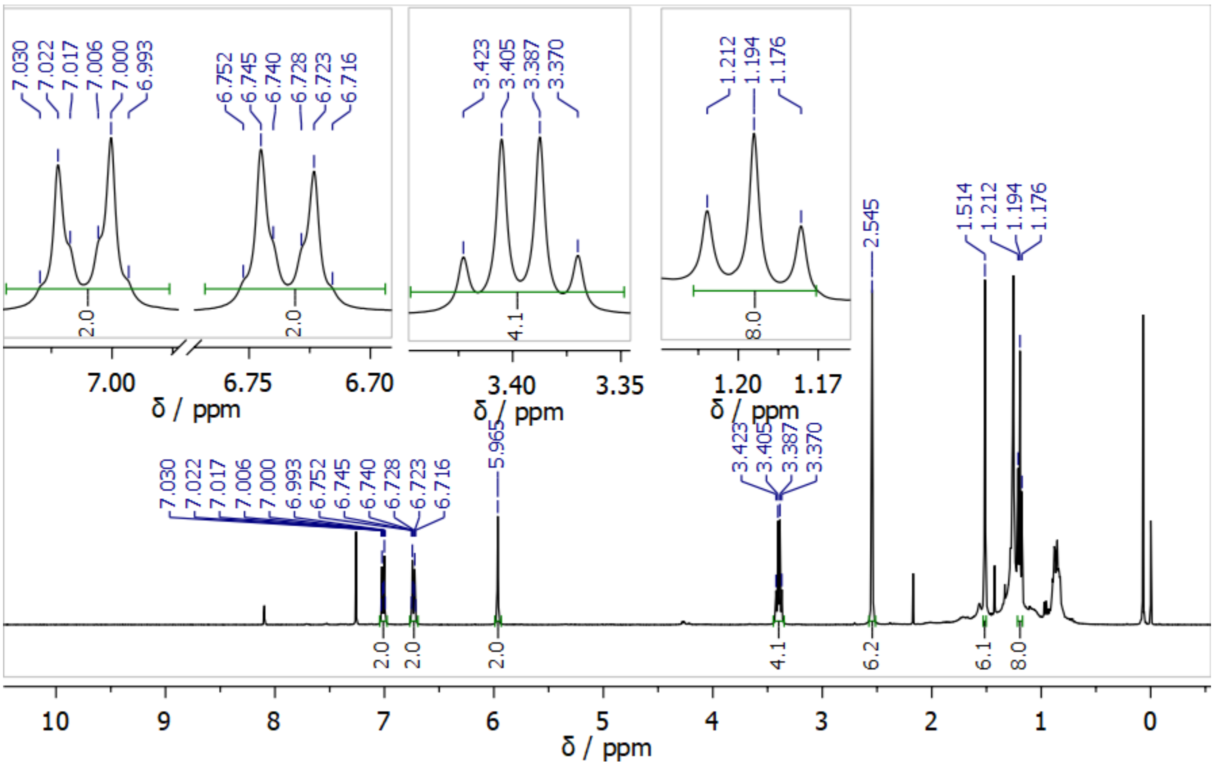

Figure S155. ^{1}H NMR spectrum of **BF$_2$-BDP-NEt2** (400 MHz, $CDCl_3$).

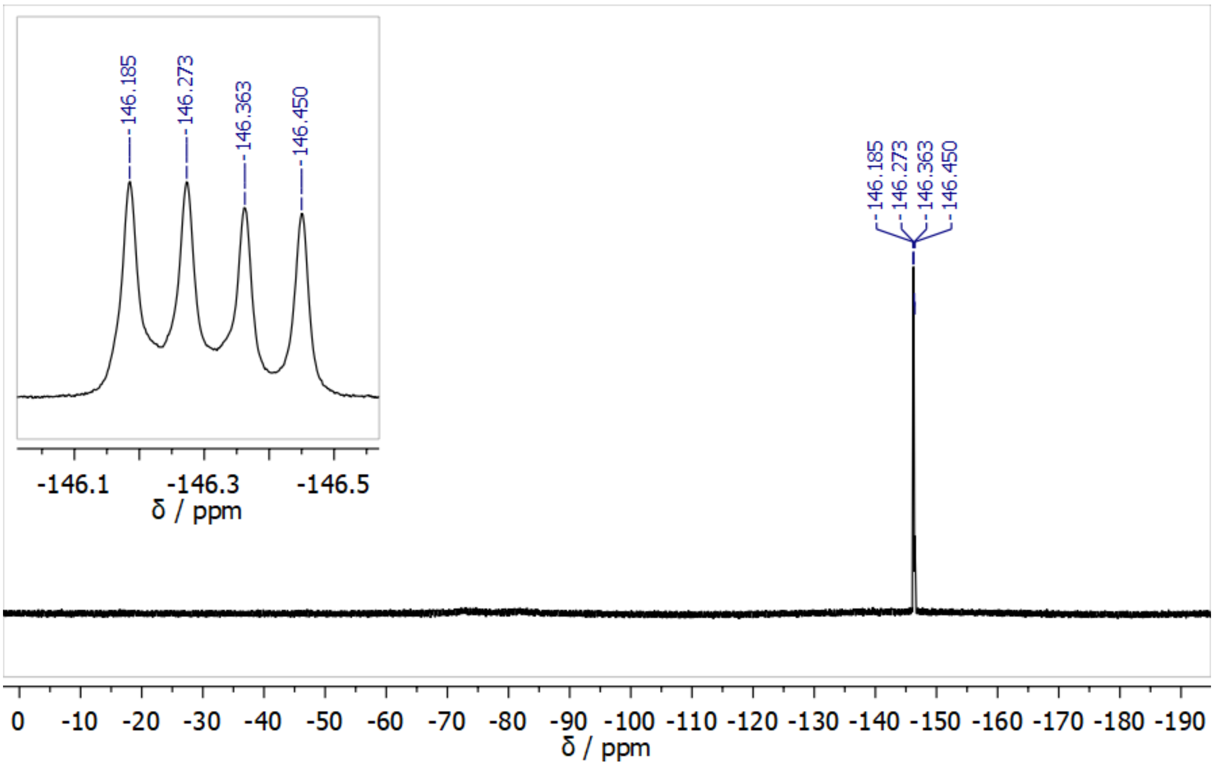

Figure S156. ^{19}F NMR spectrum of **BF$_2$-BDP-NEt2** (376 MHz, $CDCl_3$).

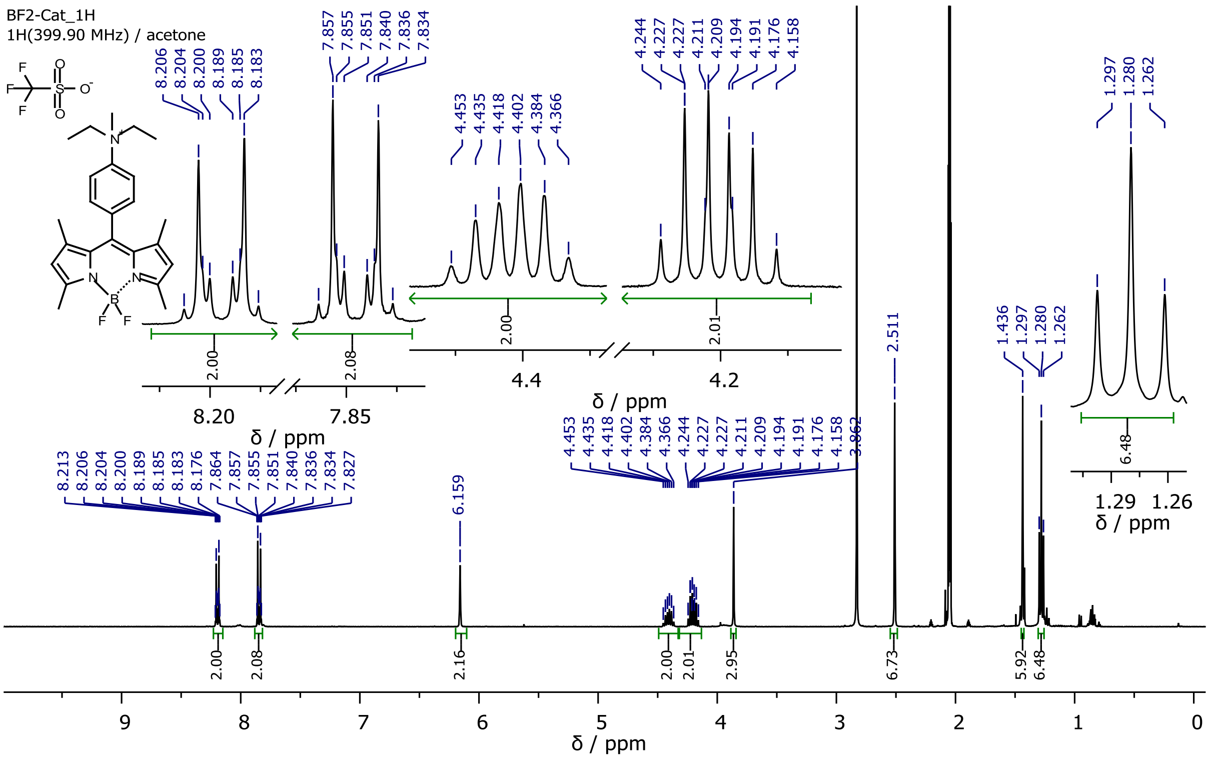

Figure S157. ^{1}H NMR spectrum of **BF$_2$-BDP-CAT** (400 MHz, $CDCl_3$).

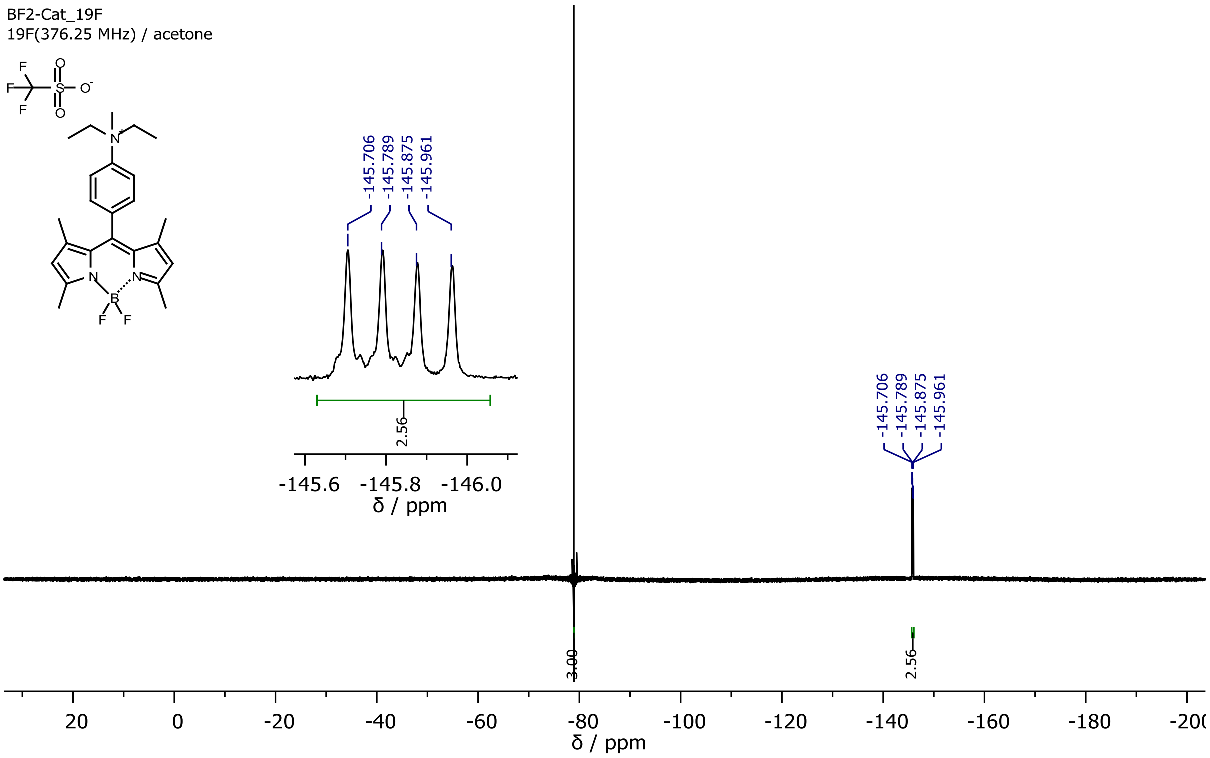

Figure S158. ^{19}F NMR spectrum of **BF$_2$-BDP-CAT** (376 MHz, $CDCl_3$).

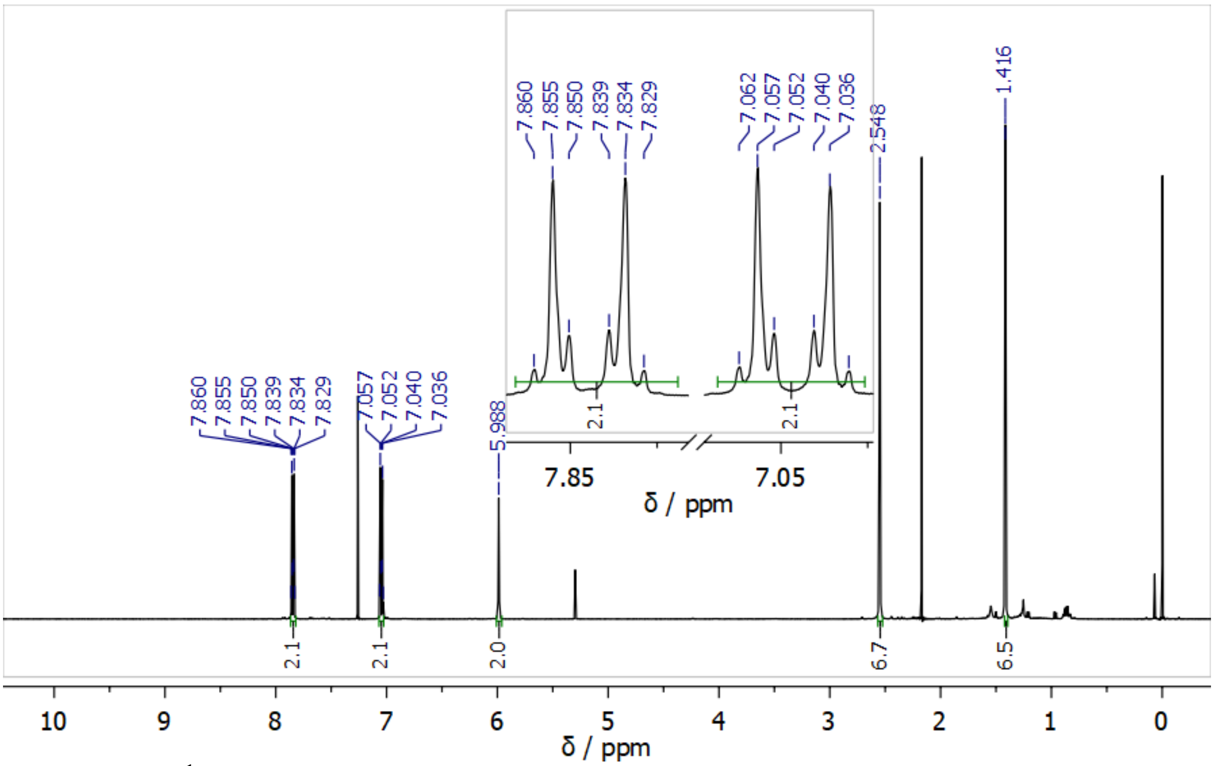

Figure S159. ^{1}H NMR spectrum of **BF$_2$-BDP-I** (400 MHz, $CDCl_3$).

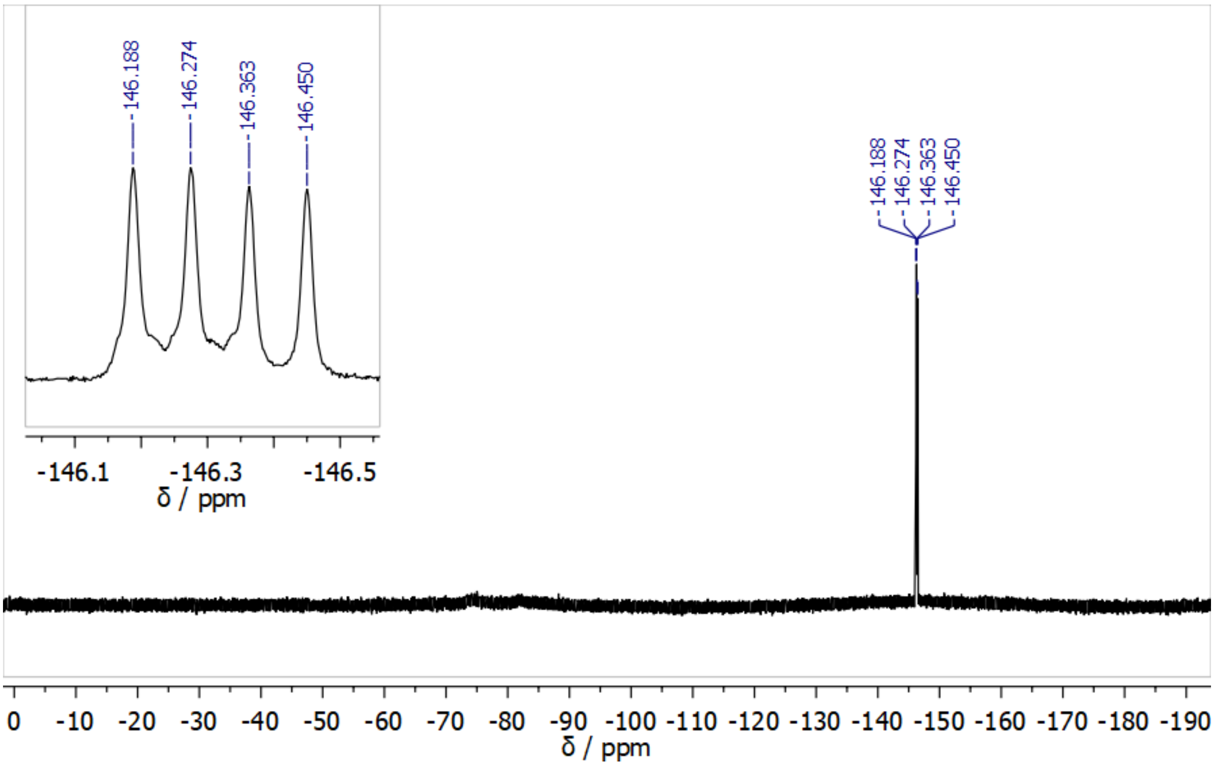

Figure S160. ^{19}F NMR spectrum of **BF$_2$-BDP-I** (376 MHz, $CDCl_3$).

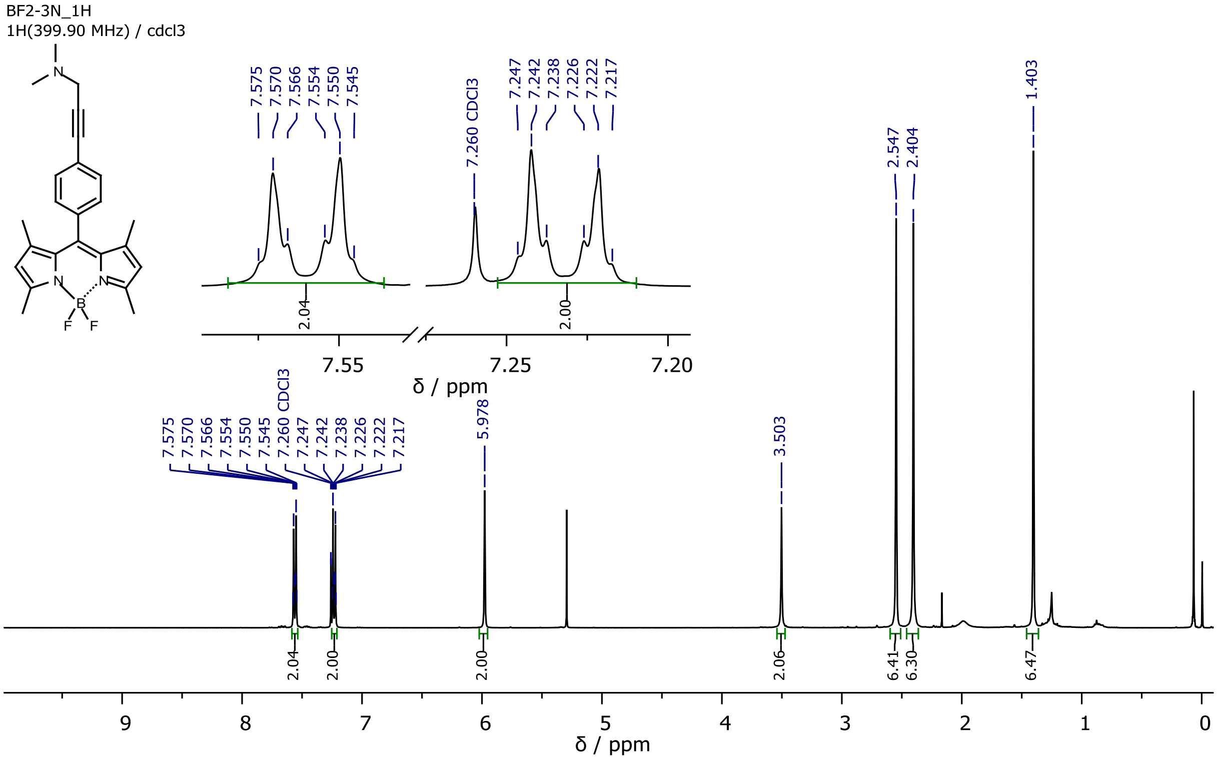

Figure S161. ^{1}H NMR spectrum of **BF$_2$-BDP-CCCH2NMe2** (400 MHz, $CDCl_3$).

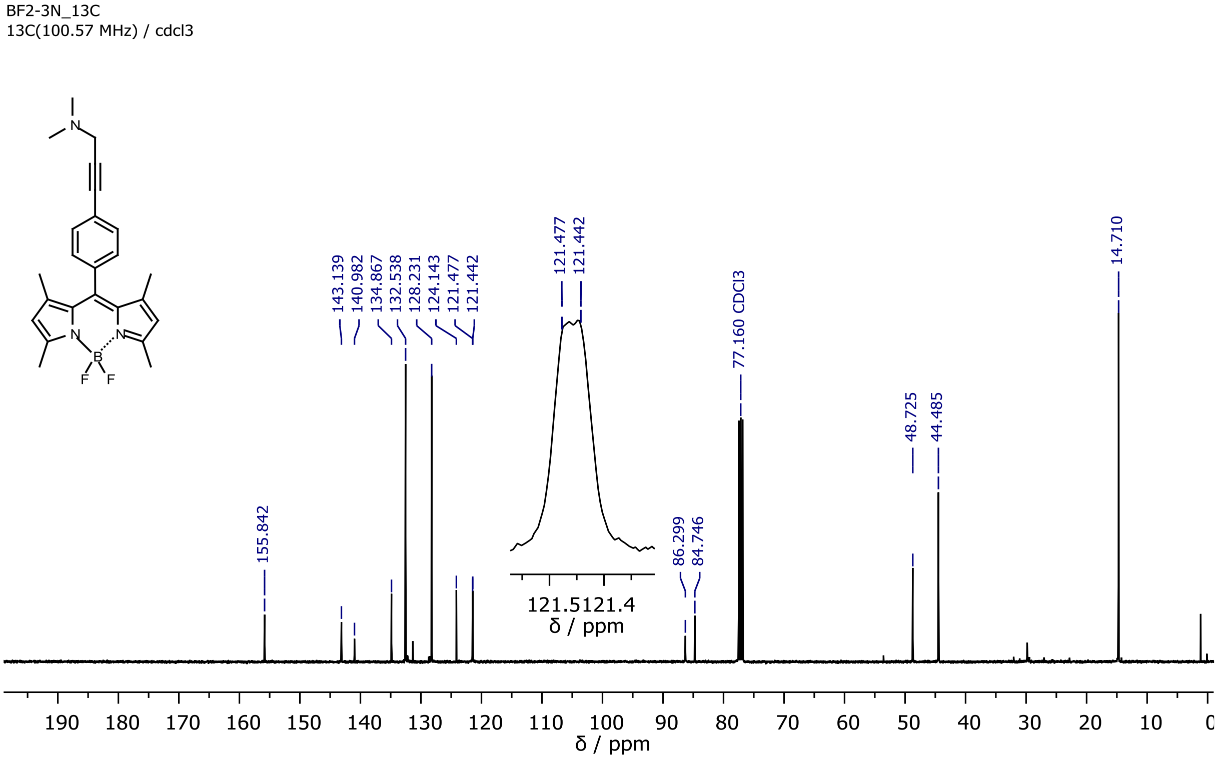

Figure S162. ^{13}C NMR spectrum of **BF$_2$-BDP-CCCH2NMe2** (101 MHz, $CDCl_3$).

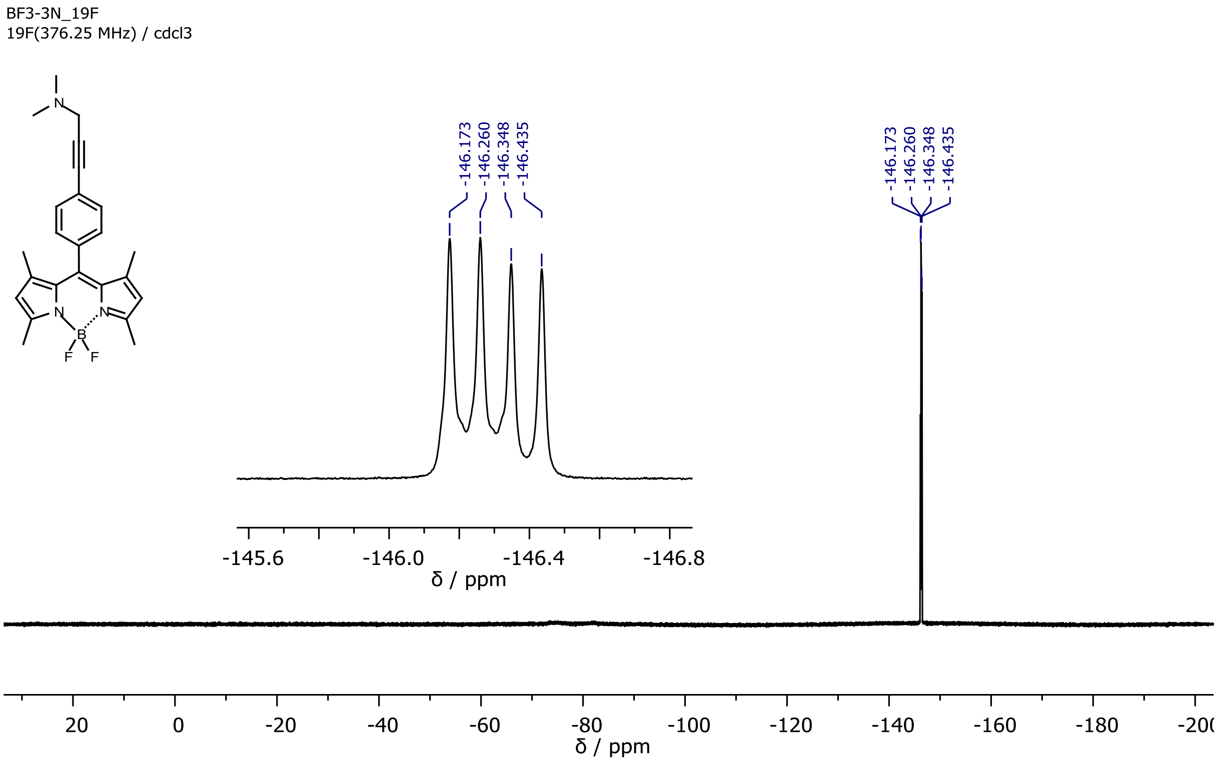

Figure S163. ^{19}F NMR spectrum of **BF$_2$-BDP-CCCH2NMe2** (376 MHz, CDCl$_3$).

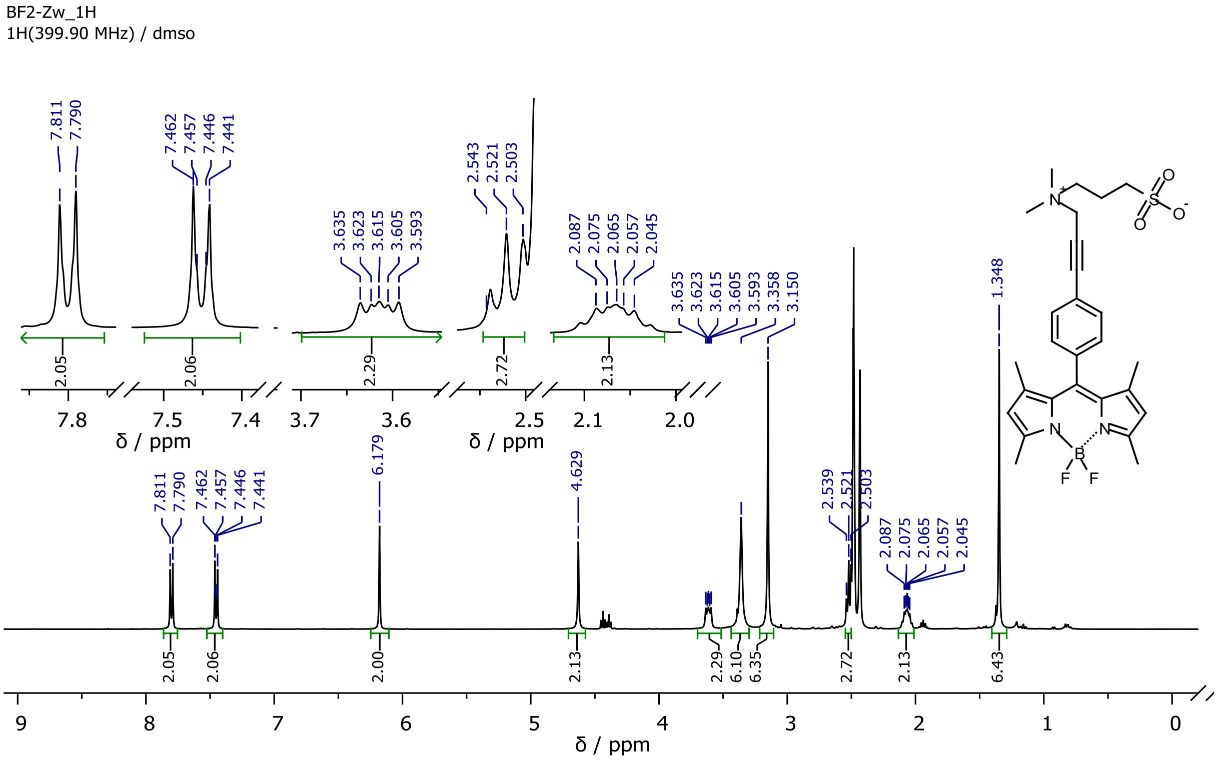

Figure S164. ^{1}H NMR spectrum of **BF$_2$-BDP-ZWIT** (400 MHz, CDCl$_3$).

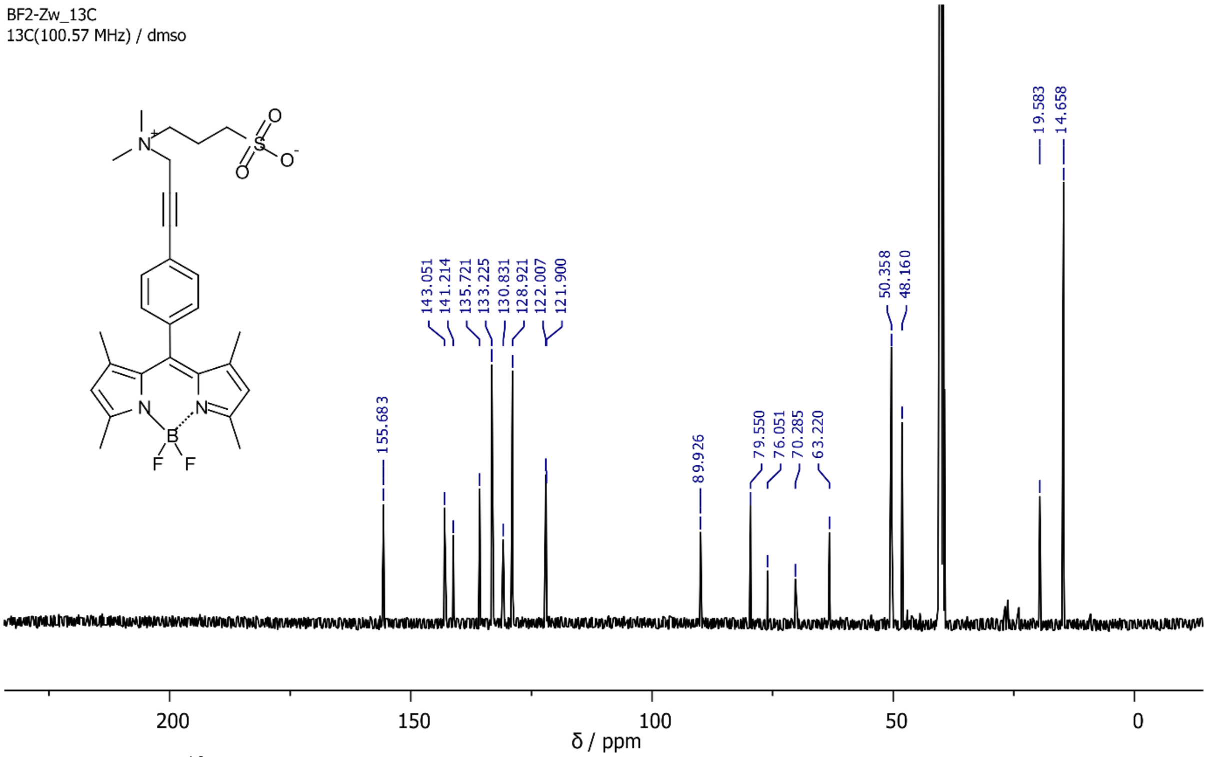

Figure S165. ^{13}C NMR spectrum of **BF$_2$-BDP-ZWIT** (101 MHz, $CDCl_3$).

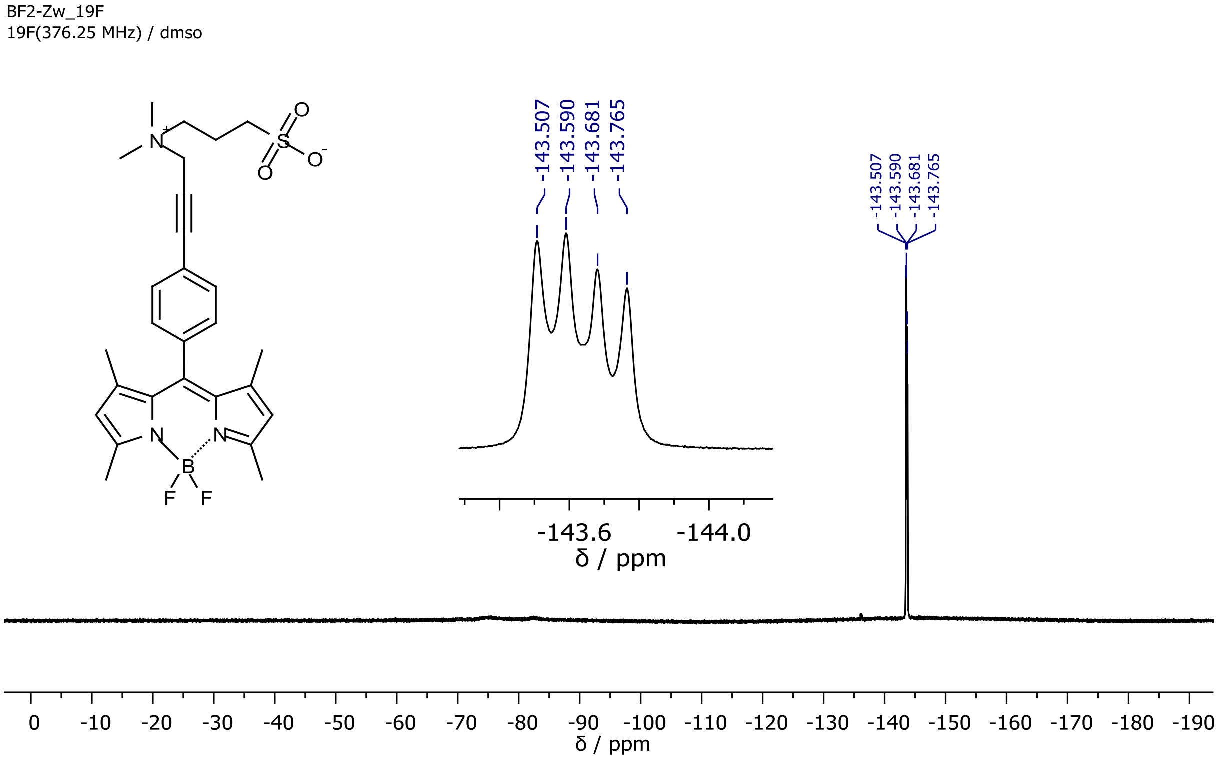

Figure S166. ^{19}F NMR spectrum of **BF$_2$-BDP-ZWIT** (376 MHz, $CDCl_3$).

9.8 NMR spectra of dipyrromethene ligand and organoboron precursors

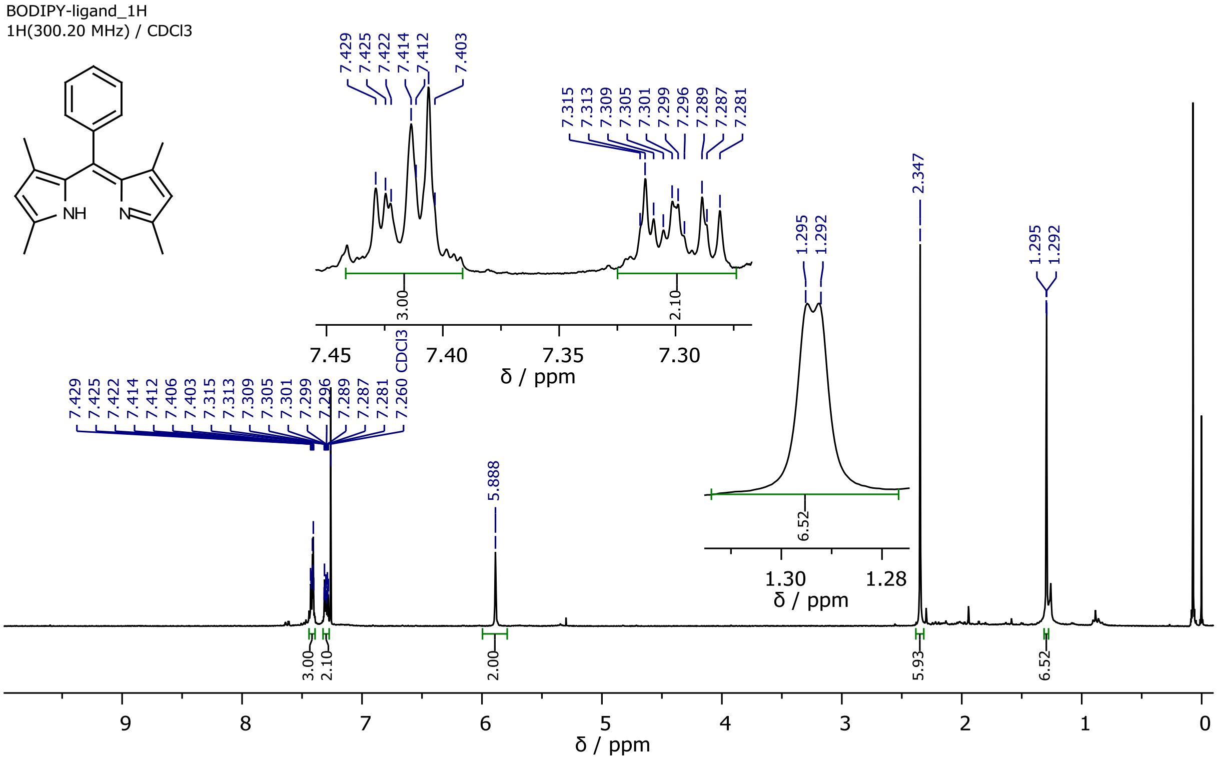

Figure S167. ^{1}H NMR spectrum of (*Z*)-2-((3,5-dimethyl-2*H*-pyrrol-2-ylidene)(phenyl)methyl)-3,5-dimethyl-1*H*-pyrrole (300 MHz, $CDCl_3$).

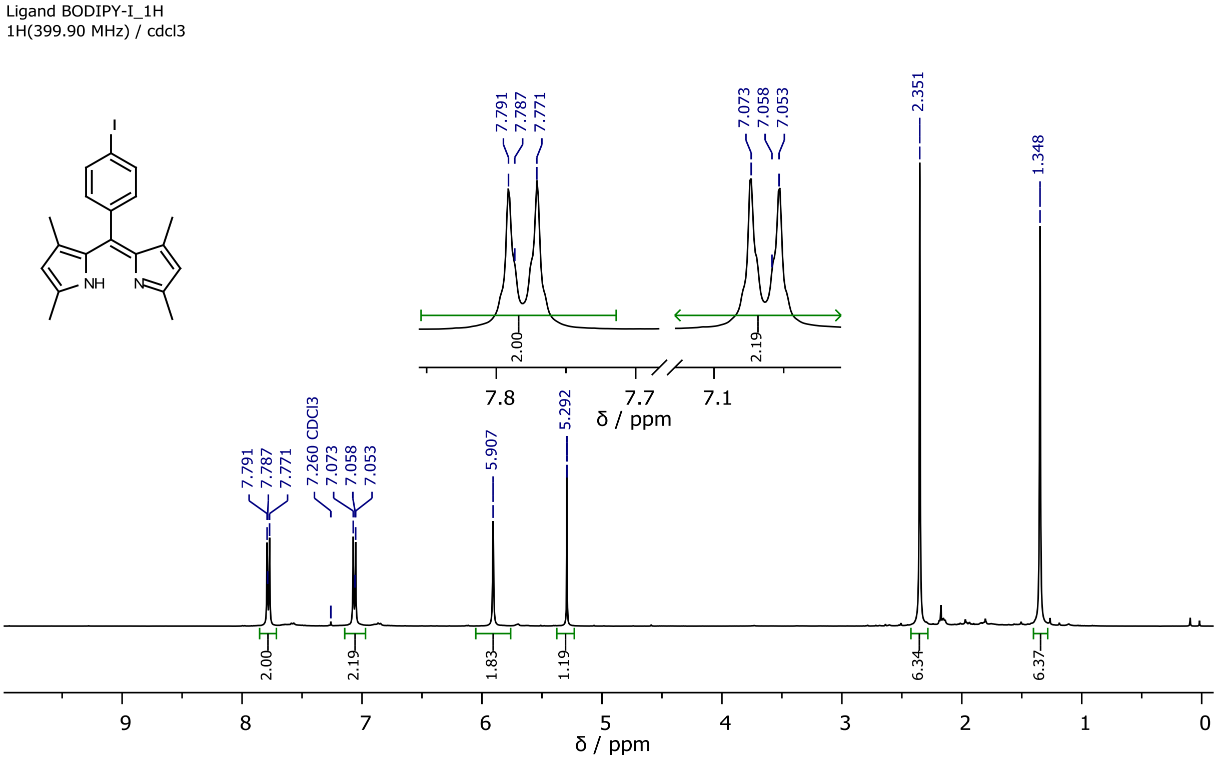

Figure S168. ^{1}H NMR spectrum of (*Z*)-2-((3,5-dimethyl-2*H*-pyrrol-2-ylidene)(4-iodophenyl)methyl)-3,5-dimethyl-1*H*-pyrrole (400 MHz, $CDCl_3$).

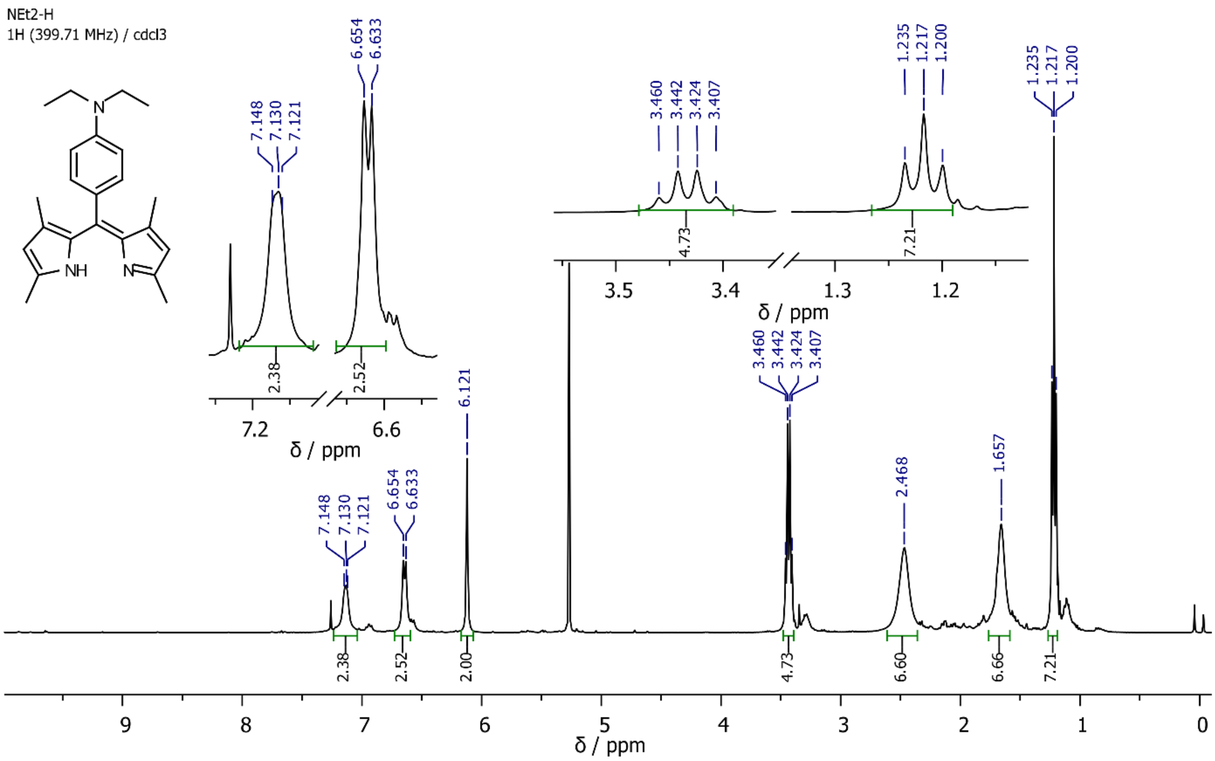

Figure S169. ^{1}H NMR spectrum of (*Z*)-4-((3,5-dimethyl-1*H*-pyrrol-2-yl)(3,5-dimethyl-2*H*-pyrrol-2-ylidene)methyl)-*N*,*N*-diethylaniline (400 MHz, $CDCl_3$).

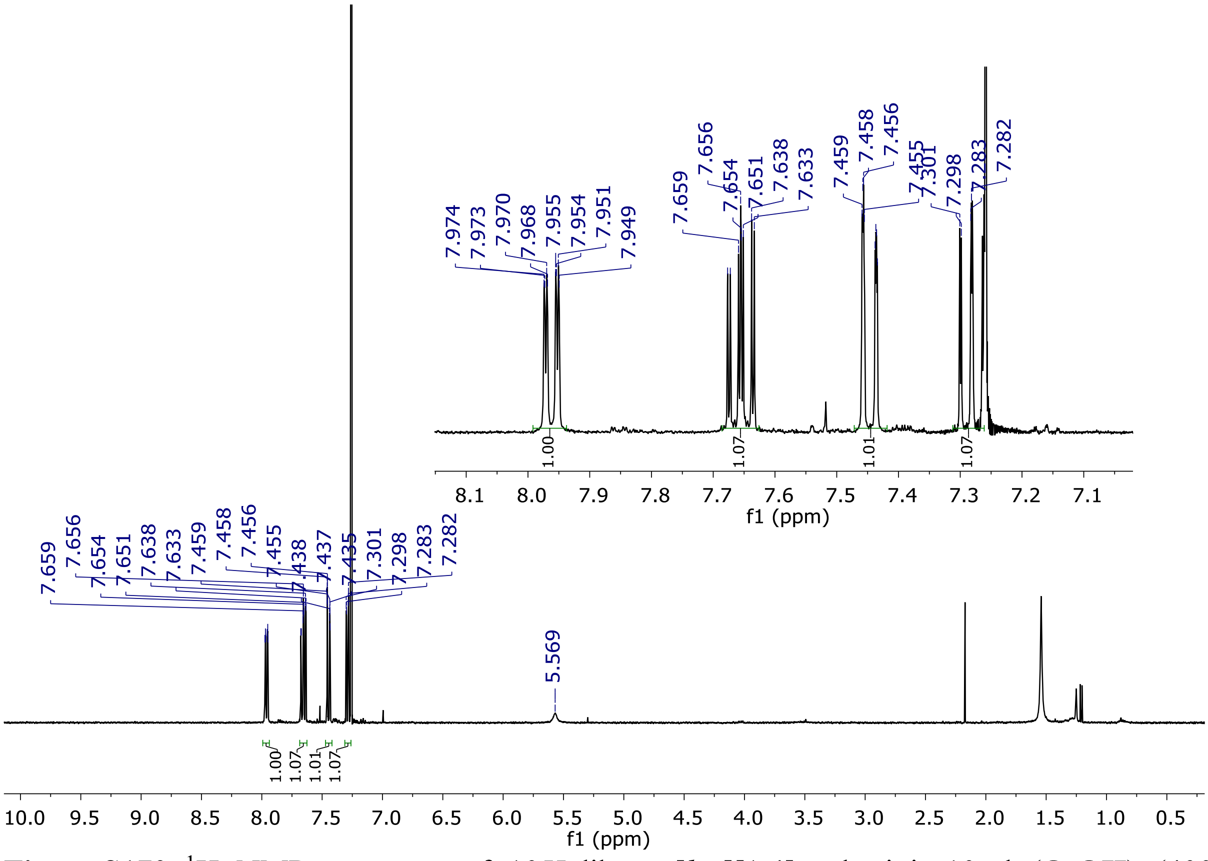

Figure S170. ^{1}H NMR spectrum of 10*H*-dibenzo[*b*,*e*][1,4]oxaborinin-10-ol (**O-OH**) (400 MHz, $CDCl_3$ +acetone-d_6).

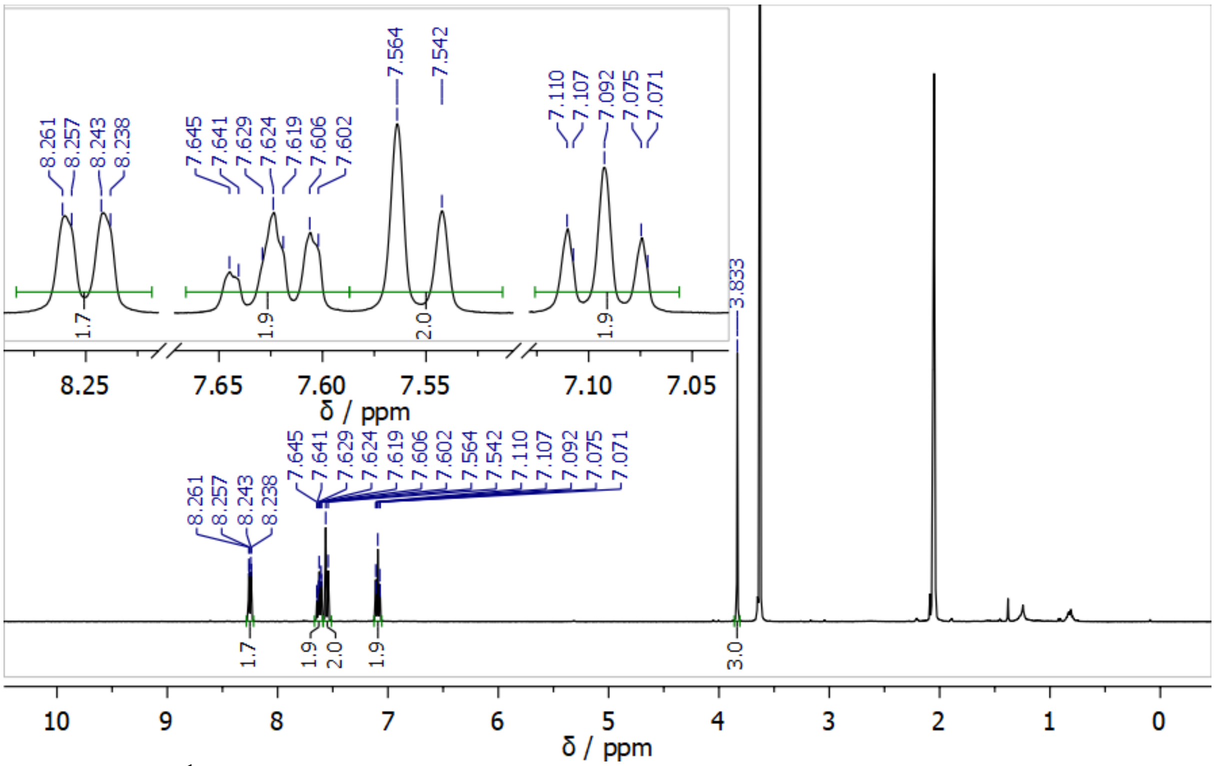

Figure S171. ^{1}H NMR spectrum of 5-methyldibenzo[*b*,*e*][1,4]azaborinin-10(5*H*)-ol (**NMe-OH**) (400 MHz, acetone-d_6+D_2O).

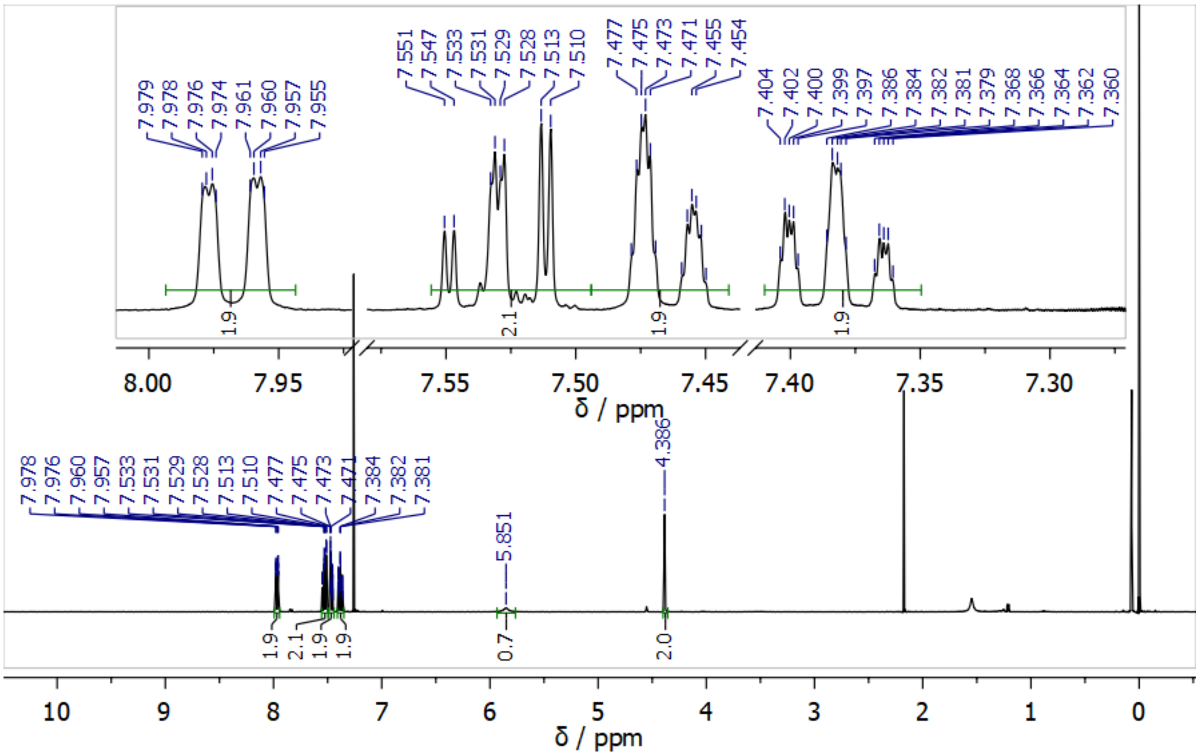

Figure S172. ^{1}H NMR spectrum of dibenzo[*b*,*e*]borinin-5(10*H*)-ol (**CH2-OH**) (400 MHz, $CDCl_3$ + acetone-d_6).

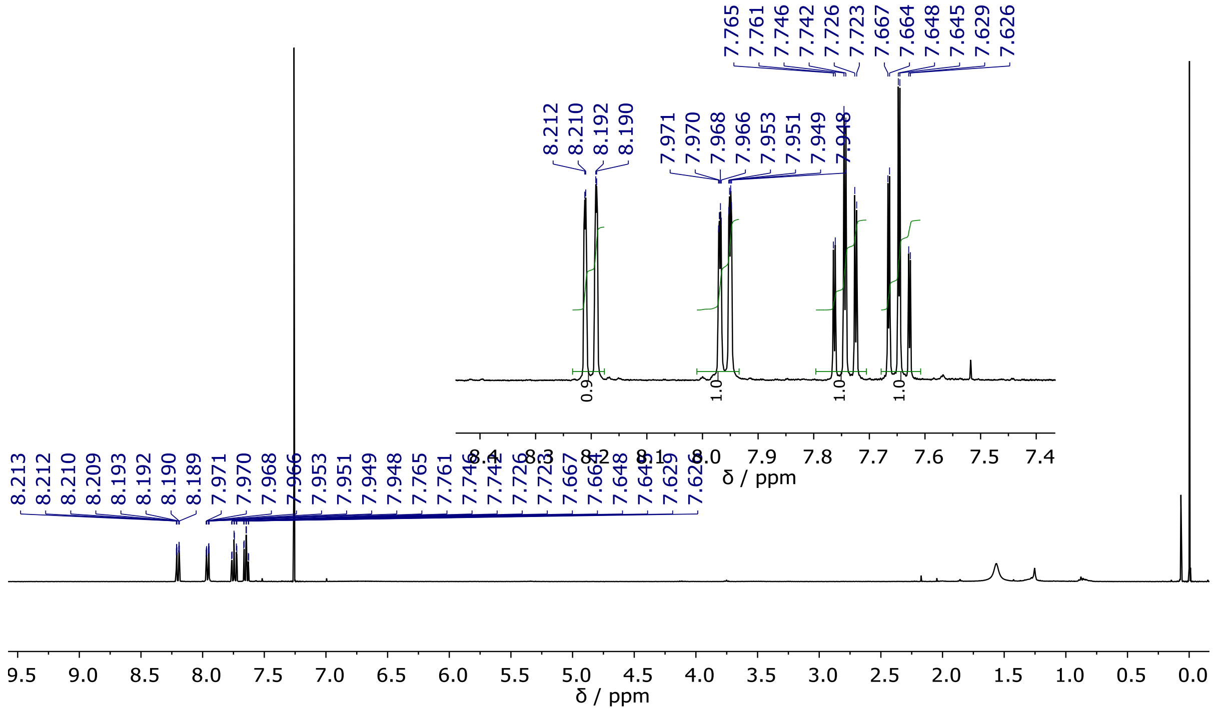

Figure S173. ^{1}H NMR spectrum of 10-Hydroxy-10*H*-dibenzo[*b*,*e*][1,4]thiaborinine 5,5-dioxide (**SO2-OH**) (400 MHz, $CDCl_3$).

10. HR-MS

10.1 HR-MS data for X-BDP-NEt2

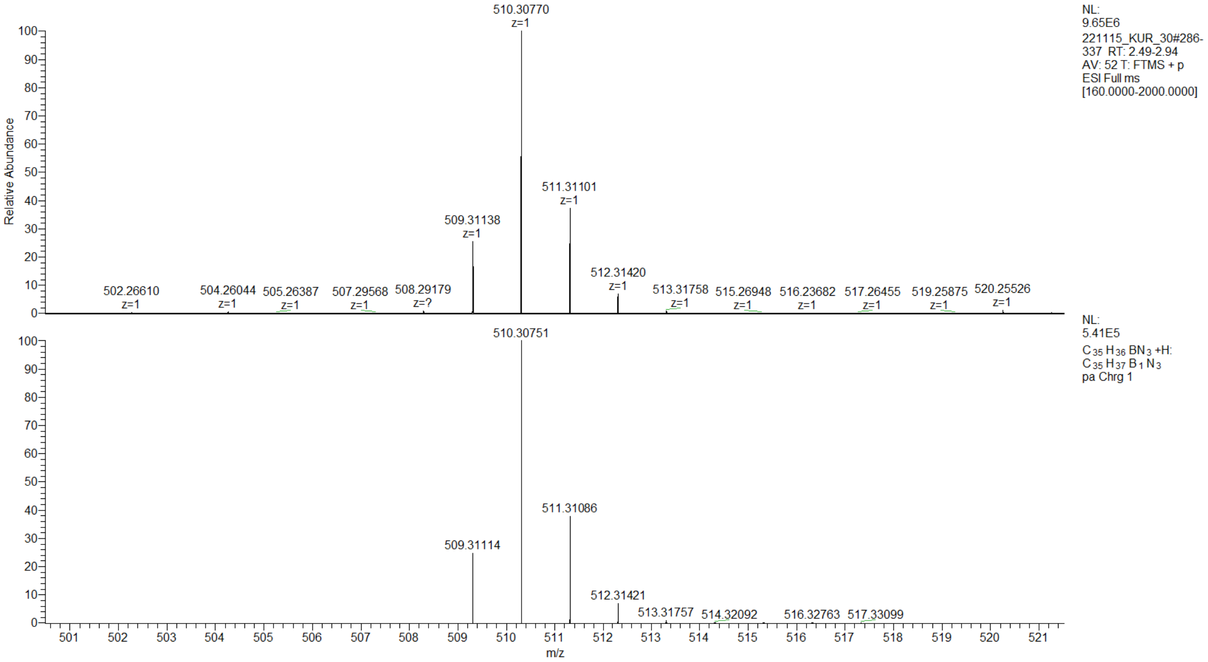

Figure S174. HR-MS (ESI) analyses of **Bf-BDP-NEt2** with positive ion mode.

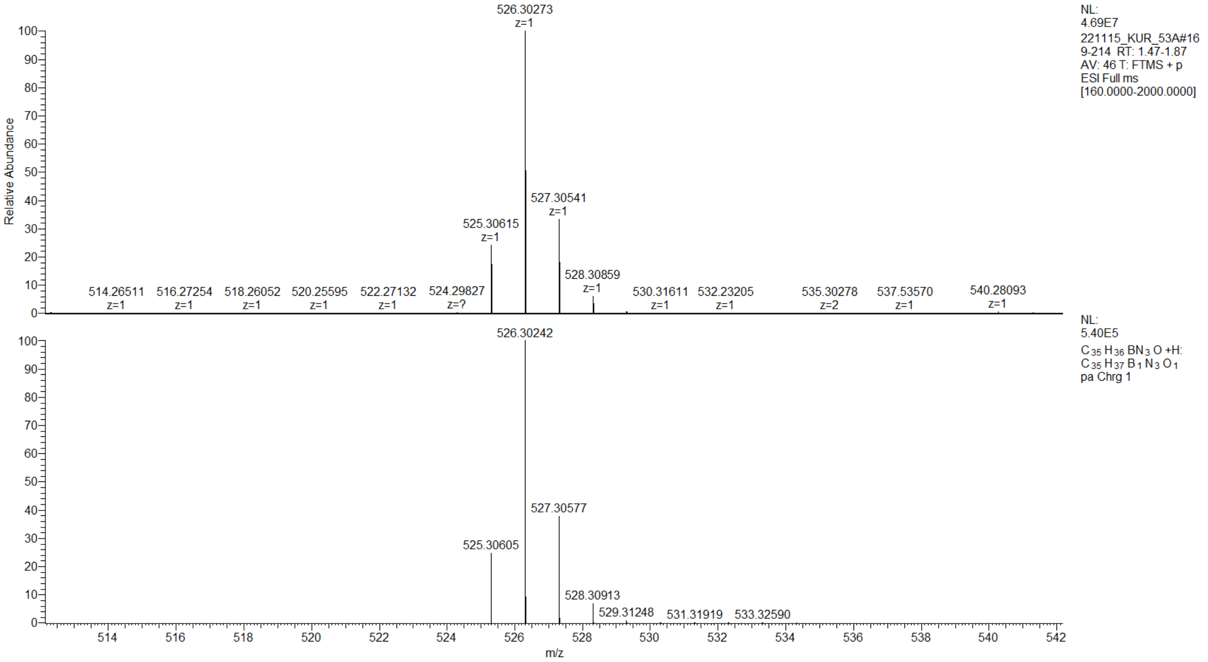

Figure S175. HR-MS (ESI) analyses of **O-BDP-NEt2** with positive ion mode.

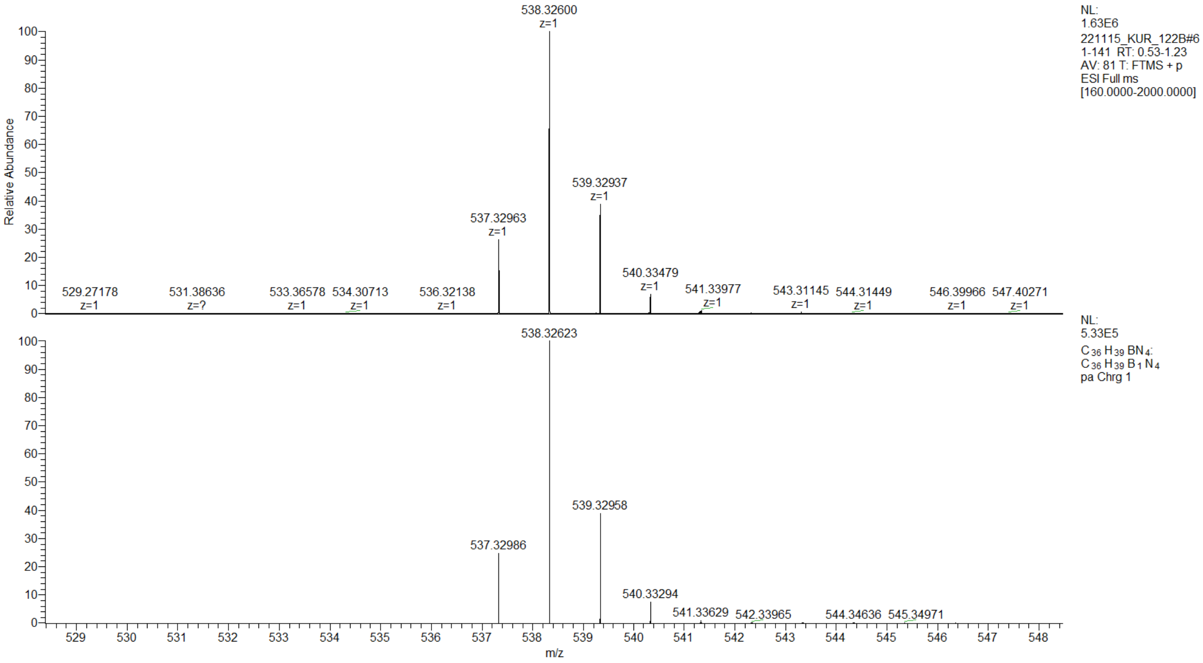

Figure S176. HR-MS (ESI) analyses of **NMe-BDP-NEt2**.

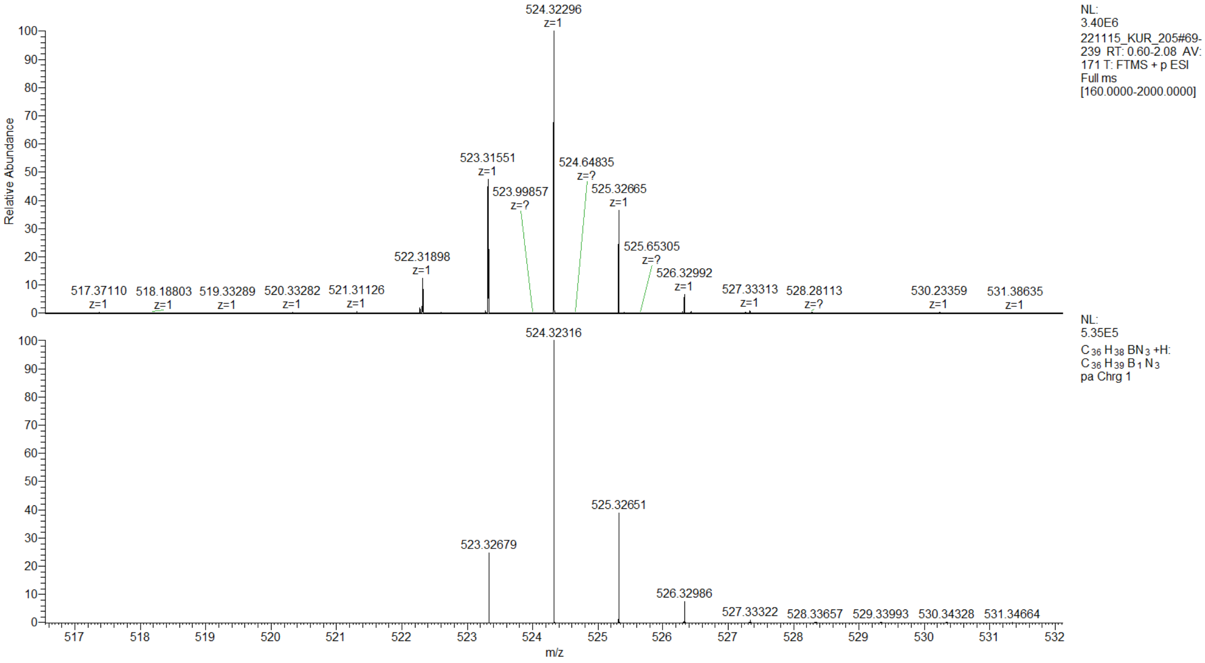

Figure S177. HR-MS (ESI) analyses of **CH2-BDP-NEt2** with positive ion mode.

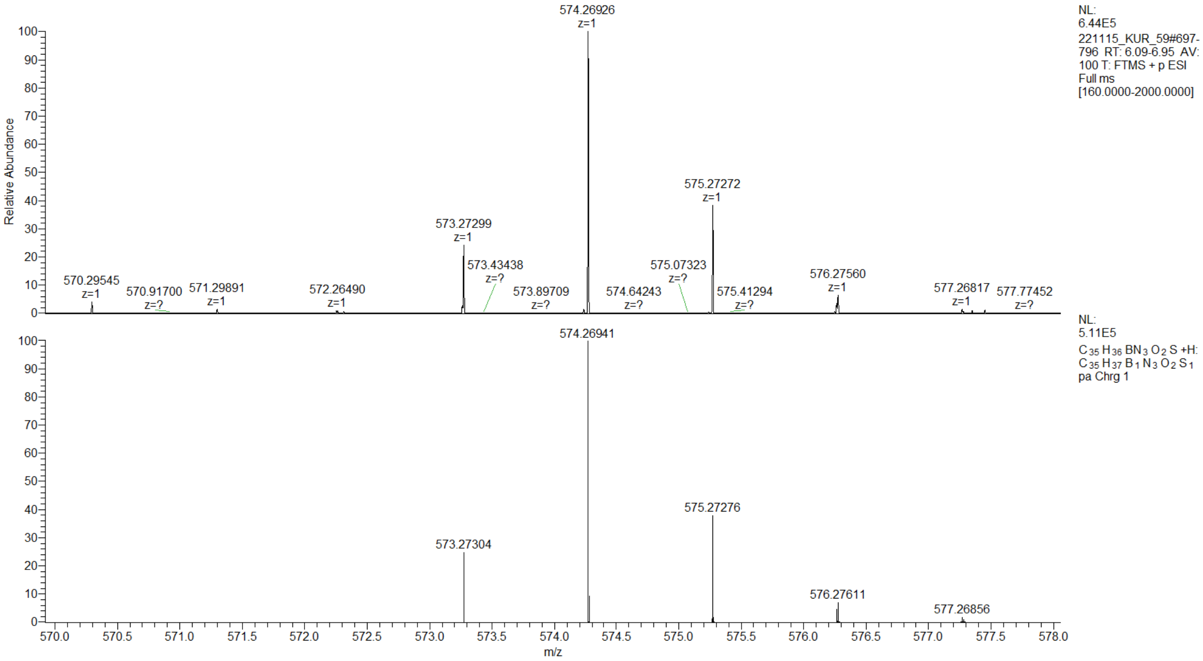

Figure S178. HR-MS (ESI) analyses of **SO2-BDP-NEt2** with positive ion mode.

10.2 HR-MS data for X-BDP-CAT

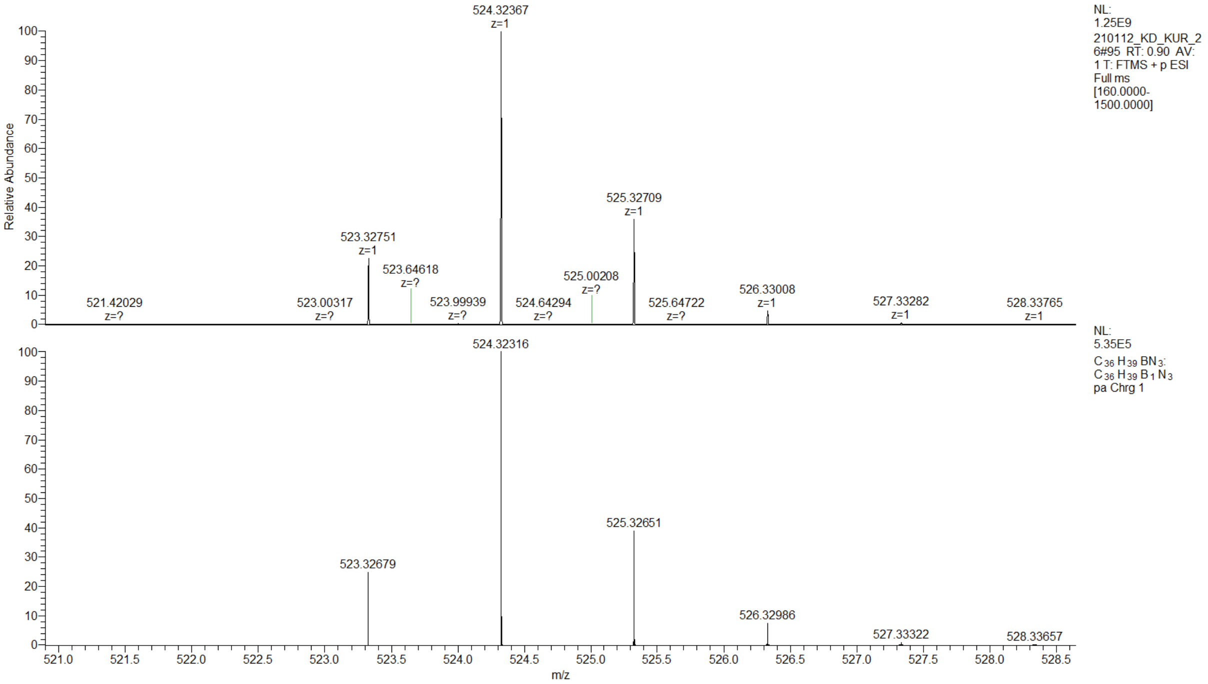

Figure S179. HR-MS (ESI) analyses of **Bf-BDP-CAT** with positive ion mode.

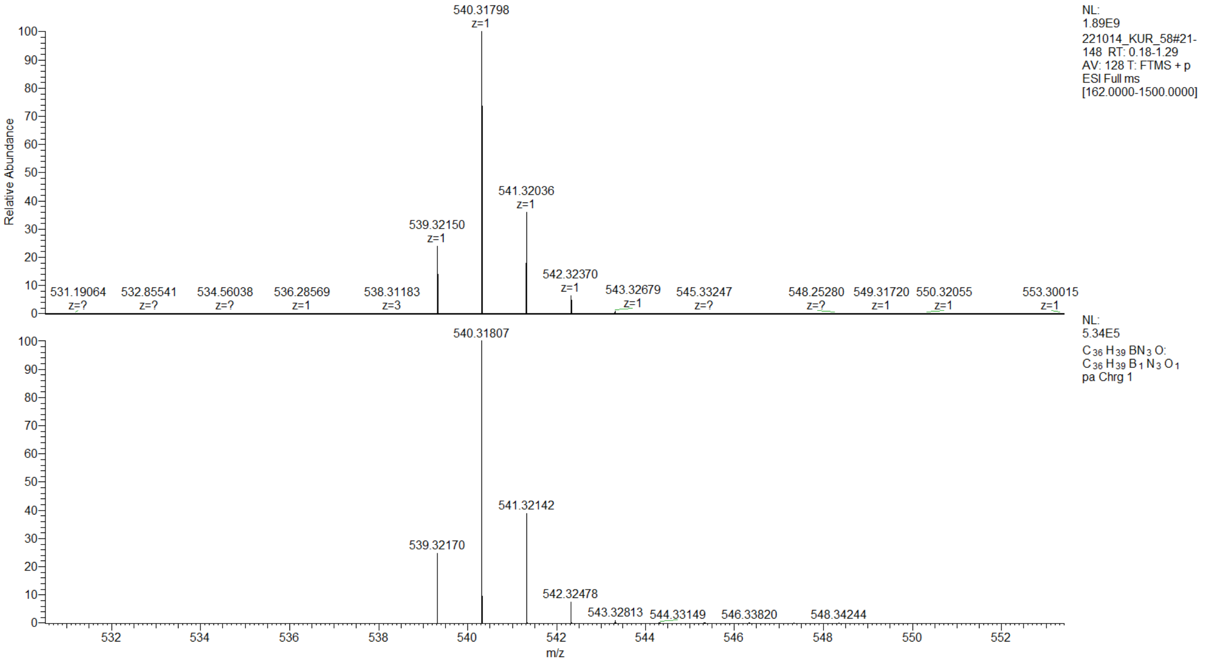

Figure S180. HR-MS (ESI) analyses of **O-BDP-CAT** with positive ion mode.

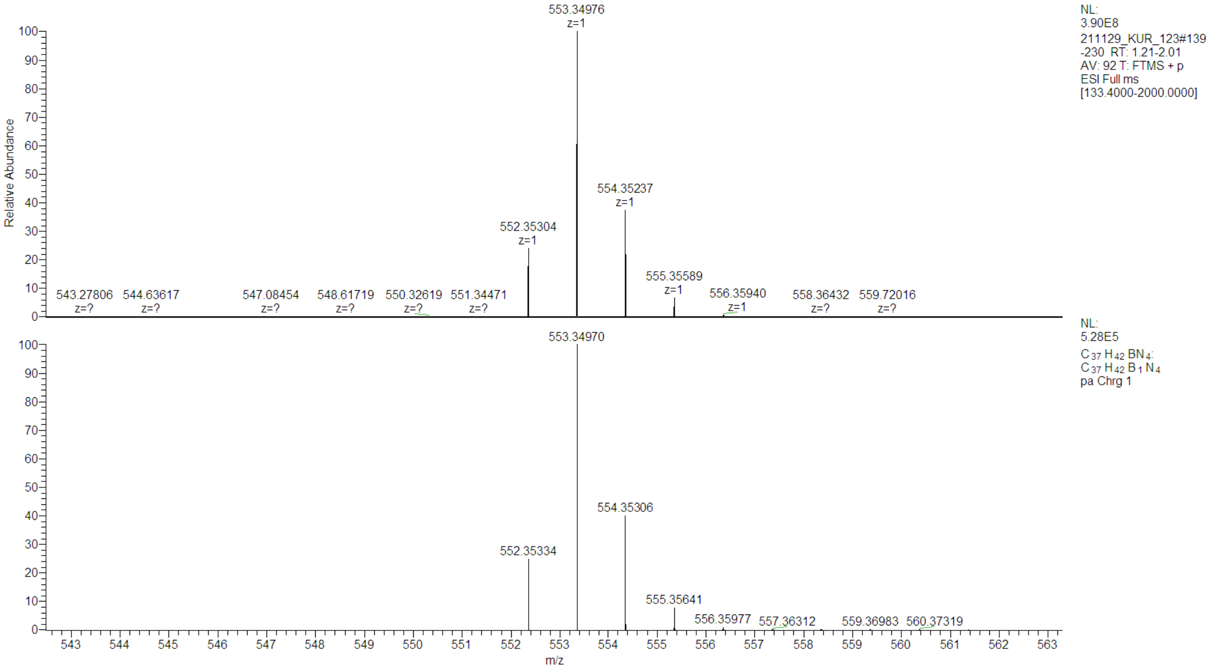

Figure S181. HR-MS (ESI) analyses of **NMe-BDP-CAT** with positive ion mode.

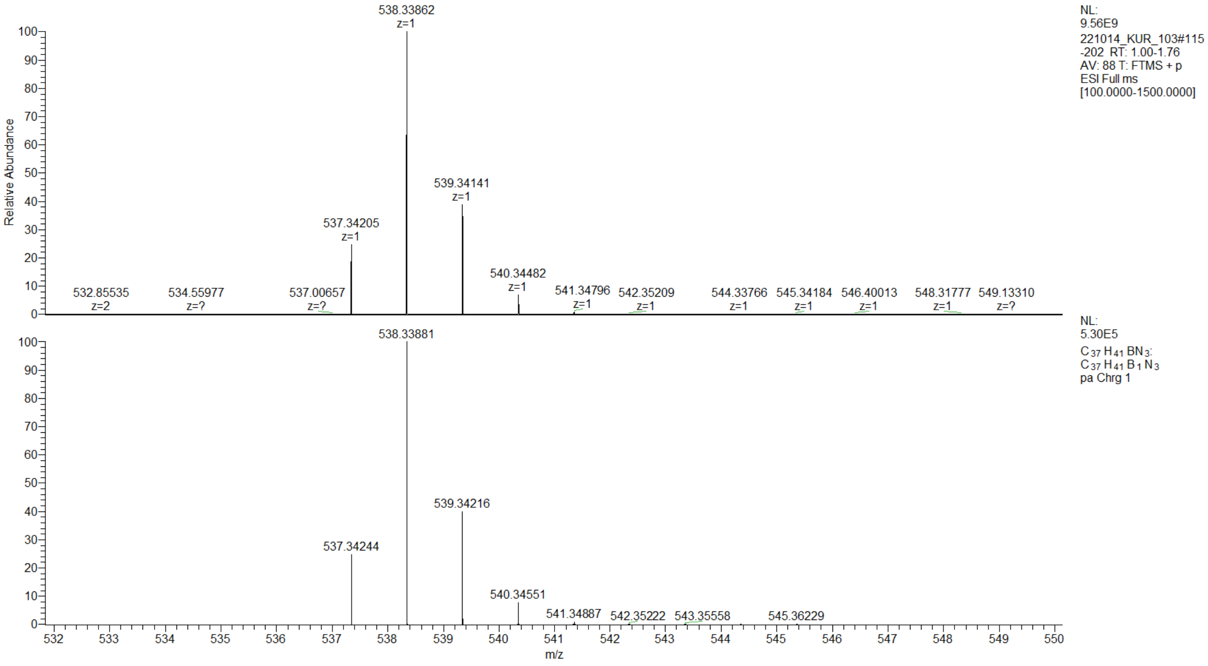

Figure S182. HR-MS (ESI) analyses of **CH2-BDP-CAT** with positive ion mode.

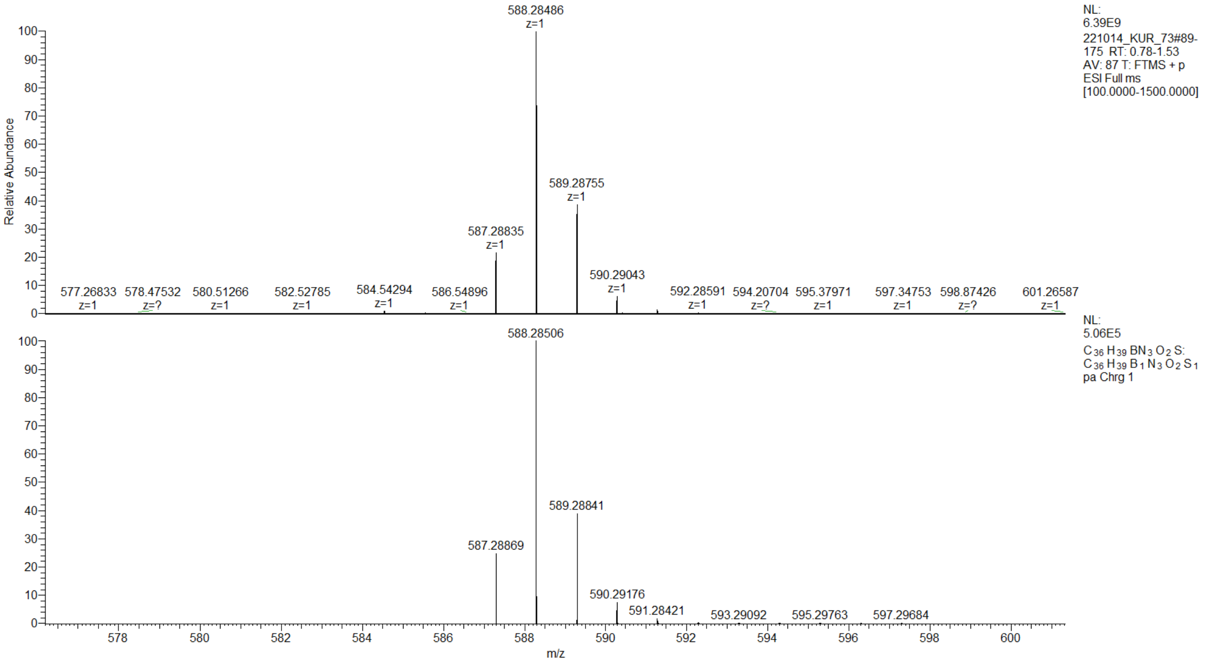

Figure S183. HR-MS (ESI) analyses of **SO2-BDP-CAT** with positive ion mode.

10.3 HR-MS data for X-BDP-I

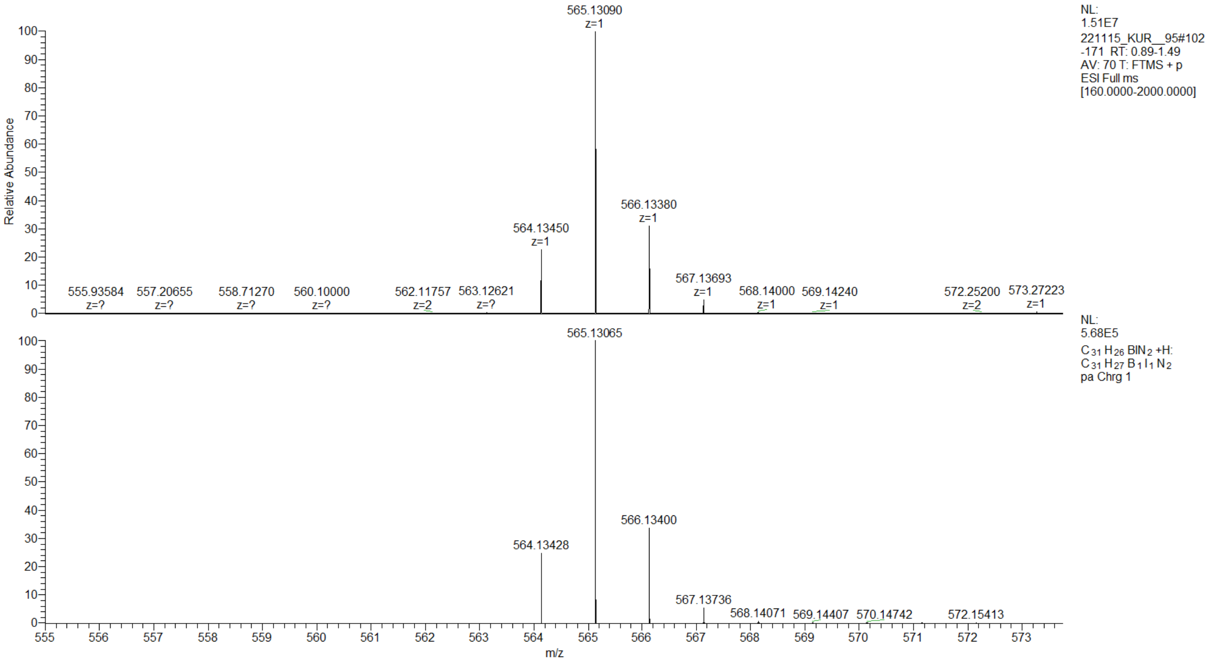

Figure S184. HR-MS (ESI) analyses of **Bf-BDP-I** with positive ion mode.

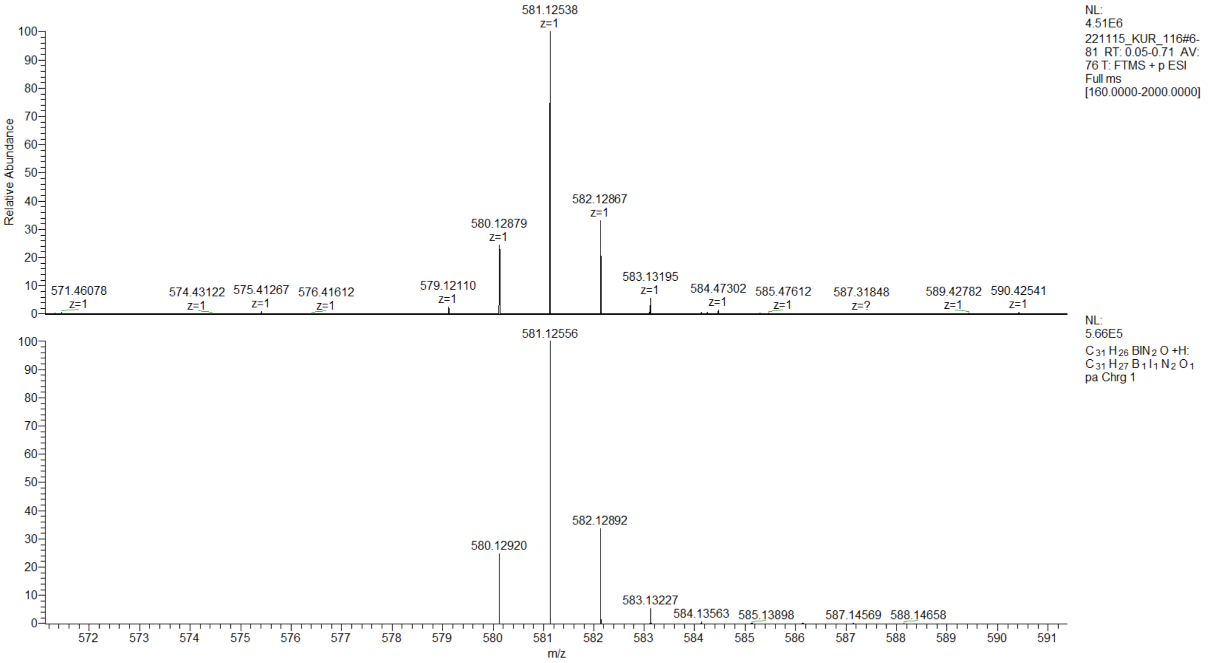

Figure S185. HR-MS (ESI) analyses of **O-BDP-I** with positive ion mode.

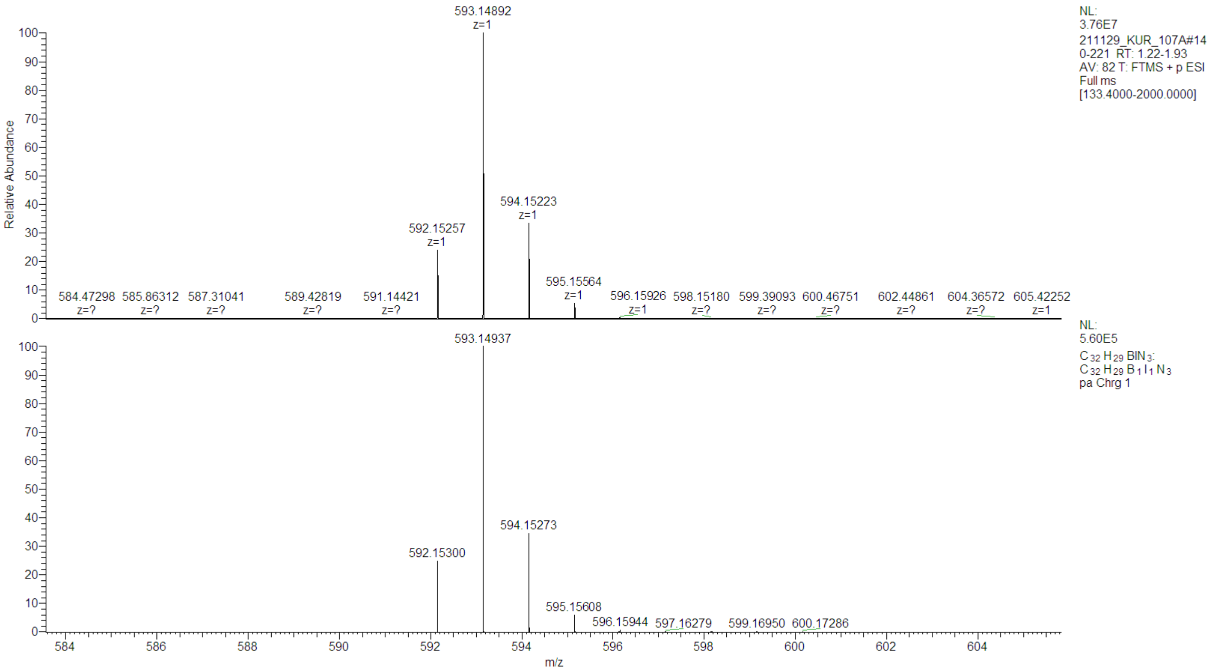

Figure S186. HR-MS (ESI) analyses of **NMe-BDP-I**.

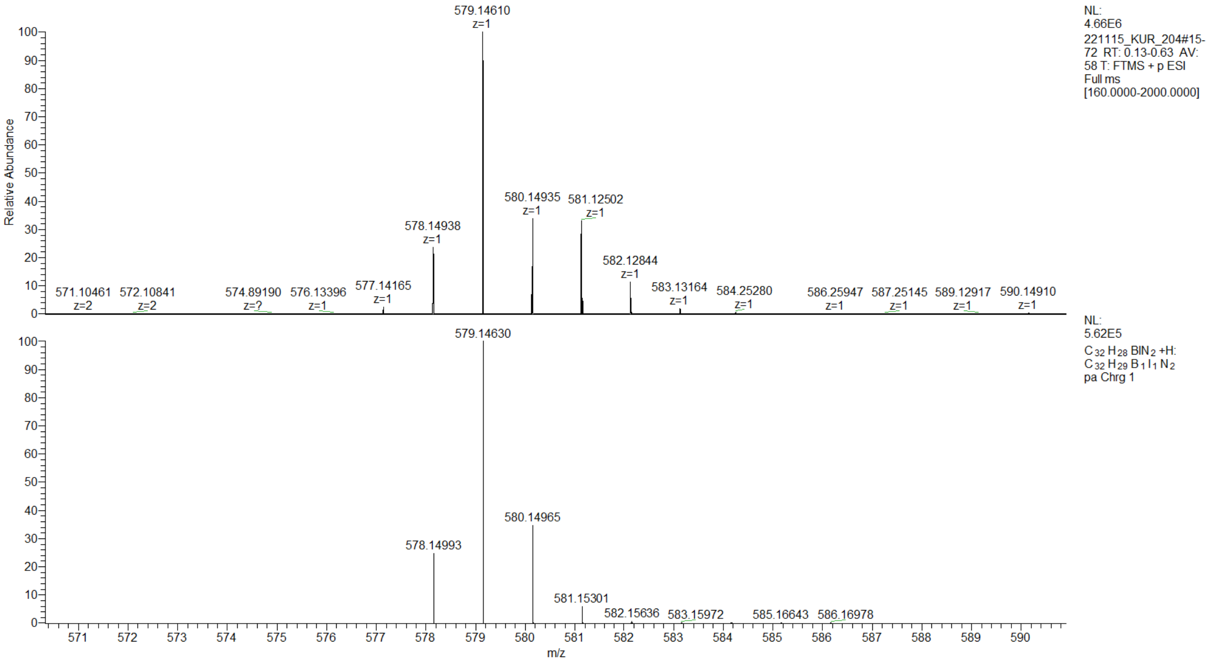

Figure S187. HR-MS (ESI) analyses of **CH2-BDP-I** with positive ion mode.

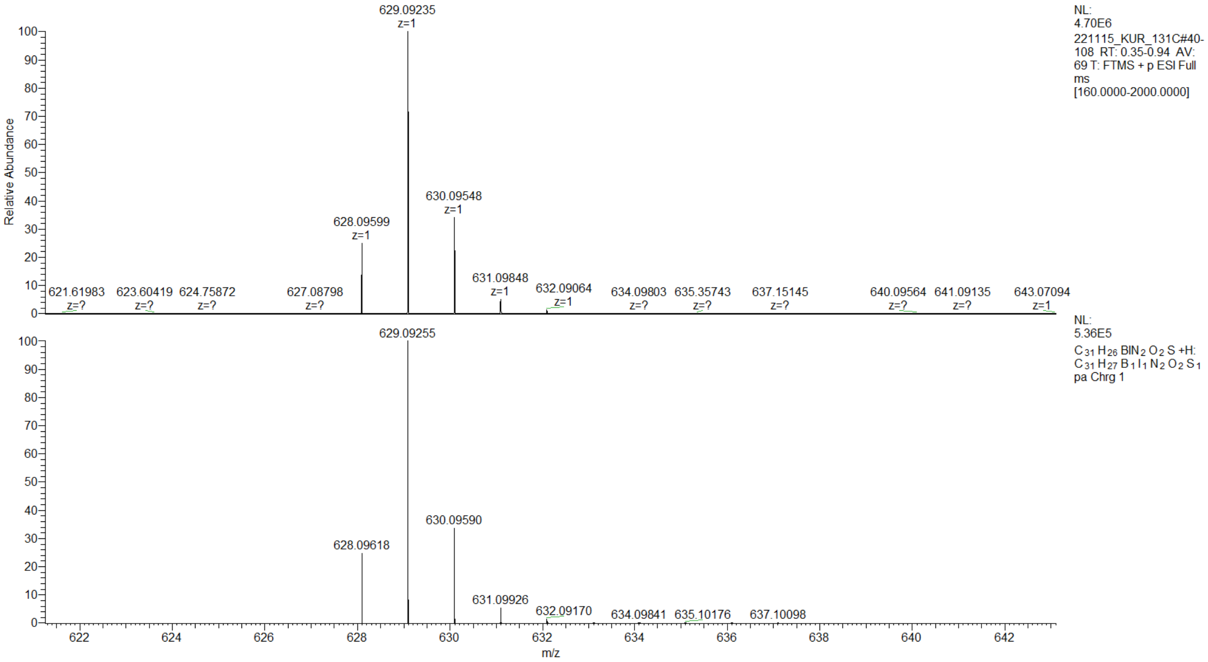

Figure S188. HR-MS (ESI) analyses of **SO2-BDP-I** with positive ion mode.

10.4 HR-MS data for X-BDP-CCCH2NMe2

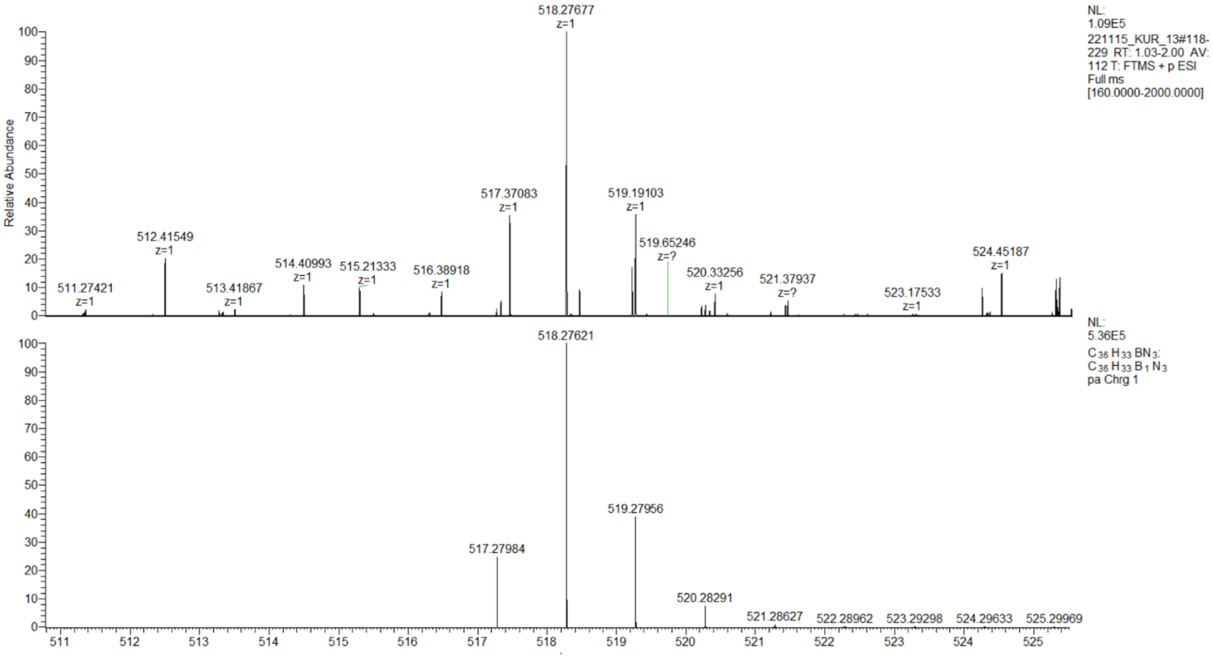

Figure S189. HR-MS (ESI) analyses of **Bf-BDP-CCCH2NMe2**.

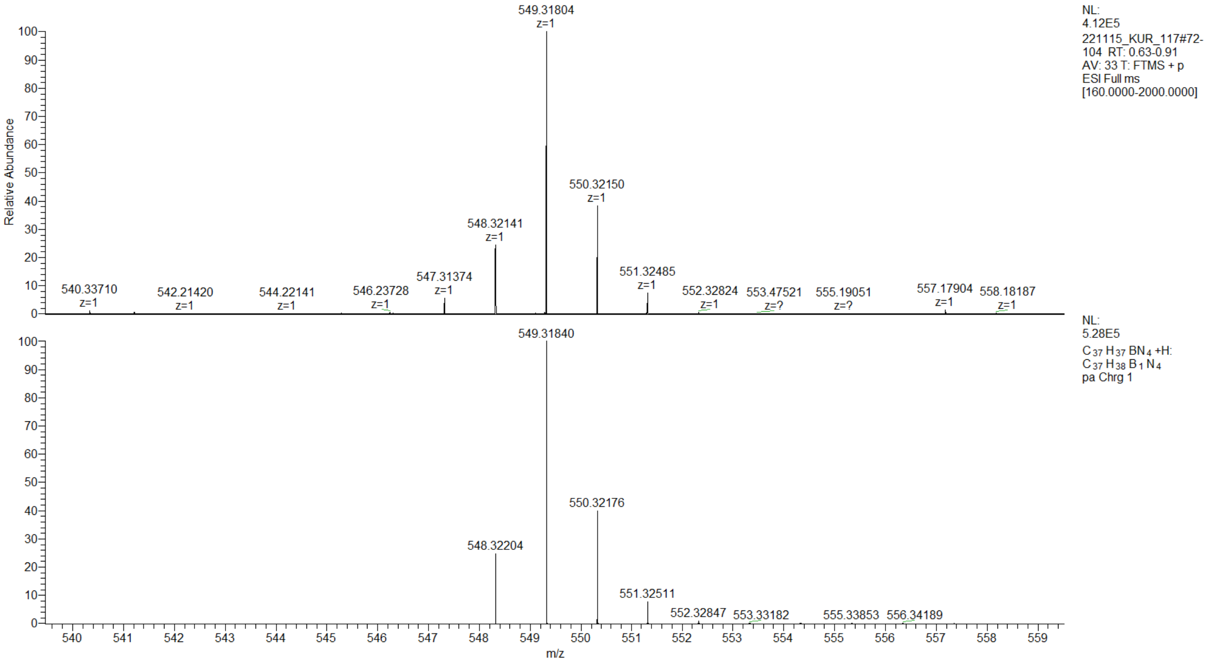

Figure S190. HR-MS (ESI) analyses of **NMe-BDP-CCCH2NMe2** with positive ion mode.

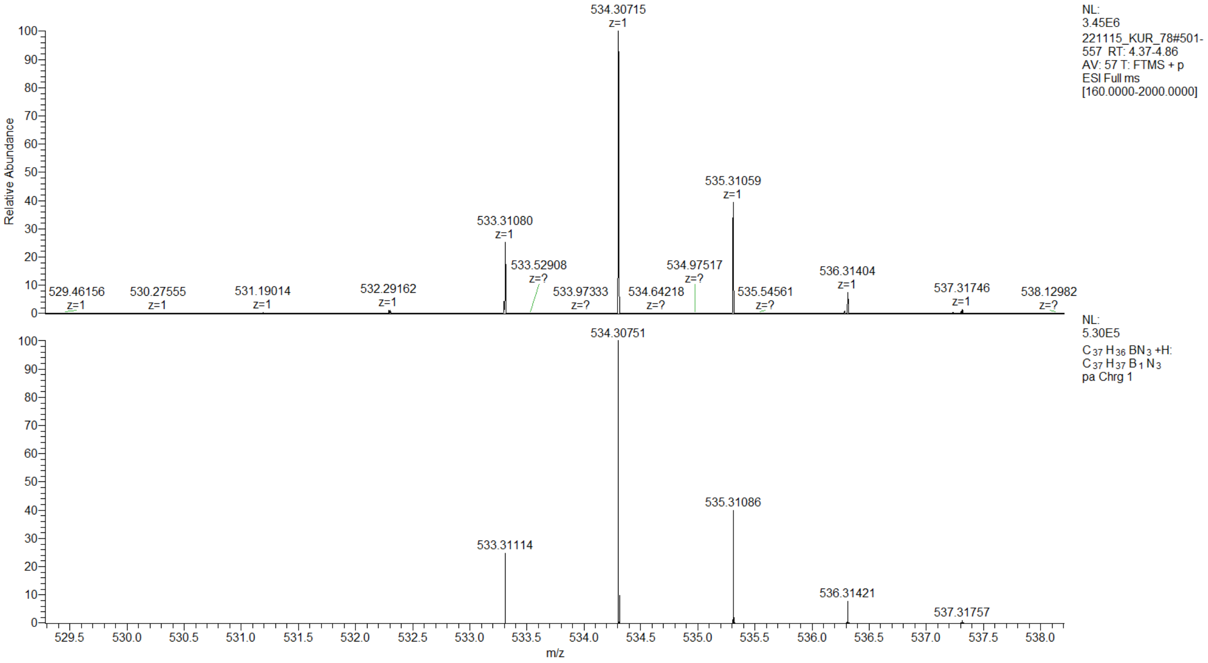

Figure S191. HR-MS (ESI) analyses of **CH2-BDP-CCCH2NMe2** with positive ion mode.

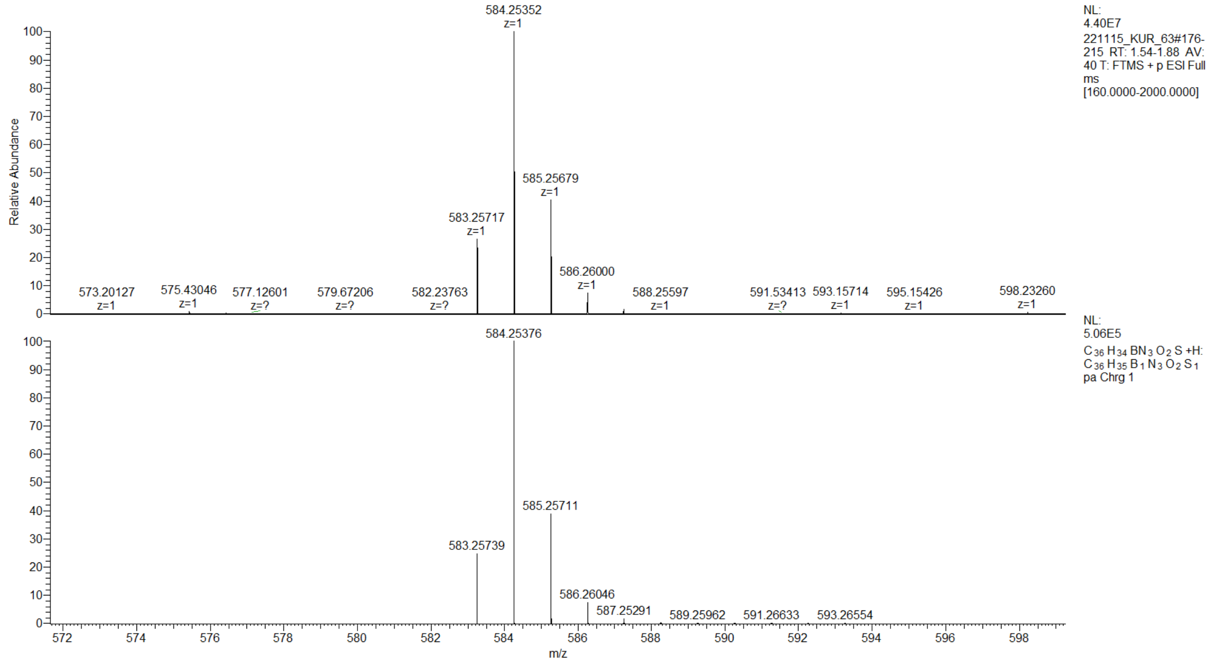

Figure S192. HR-MS (ESI) analyses of **SO2-BDP-CCCH2NMe2** with positive ion mode.

10.5 HR-MS data for X-BDP-ZWIT

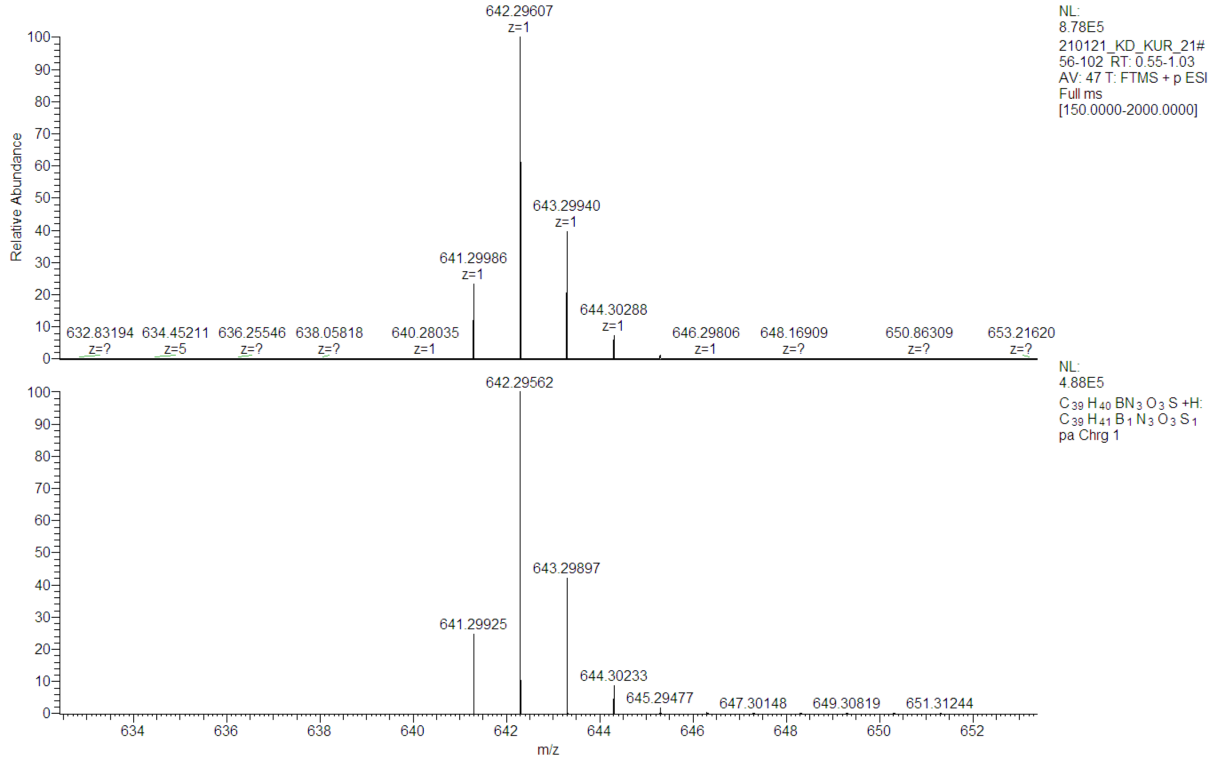

Figure S193. HR-MS (ESI) analyses of **Bf-BDP-ZWIT** with positive ion mode.

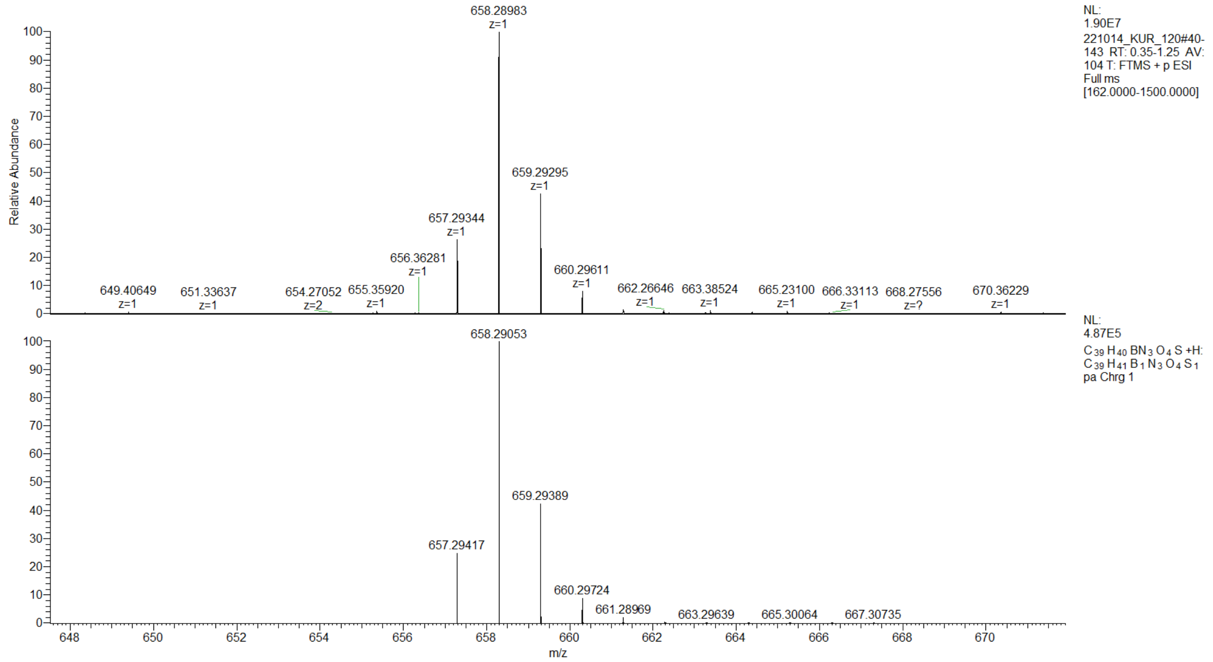

Figure S194. HR-MS (ESI) analyses of **O-BDP-ZWIT** with positive ion mode.

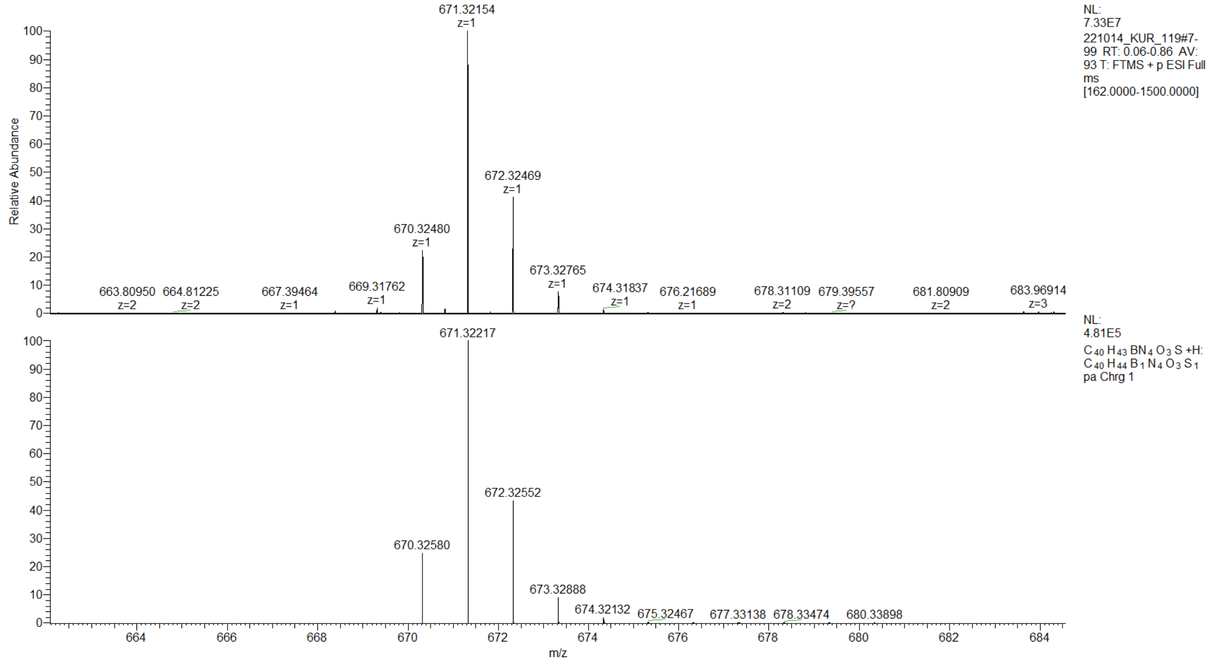

Figure S195. HR-MS (ESI) analyses of **NMe-BDP-ZWIT** with positive ion mode.

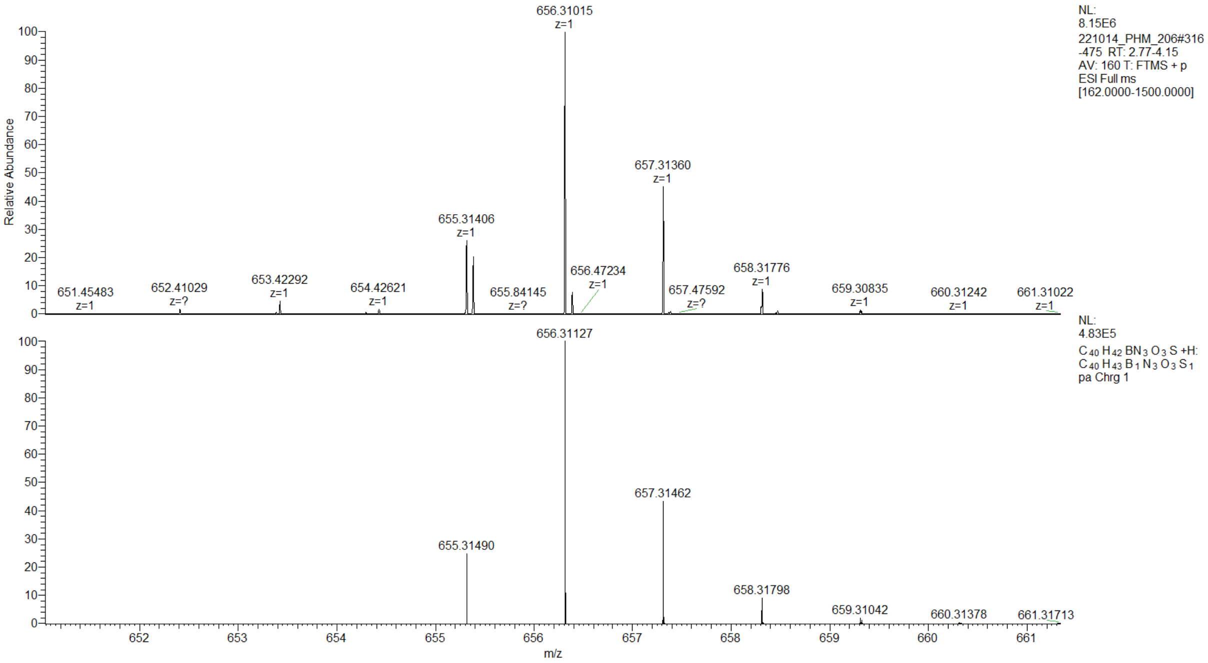

Figure S196. HR-MS (ESI) analyses of **CH2-BDP-ZWIT** with positive ion mode.

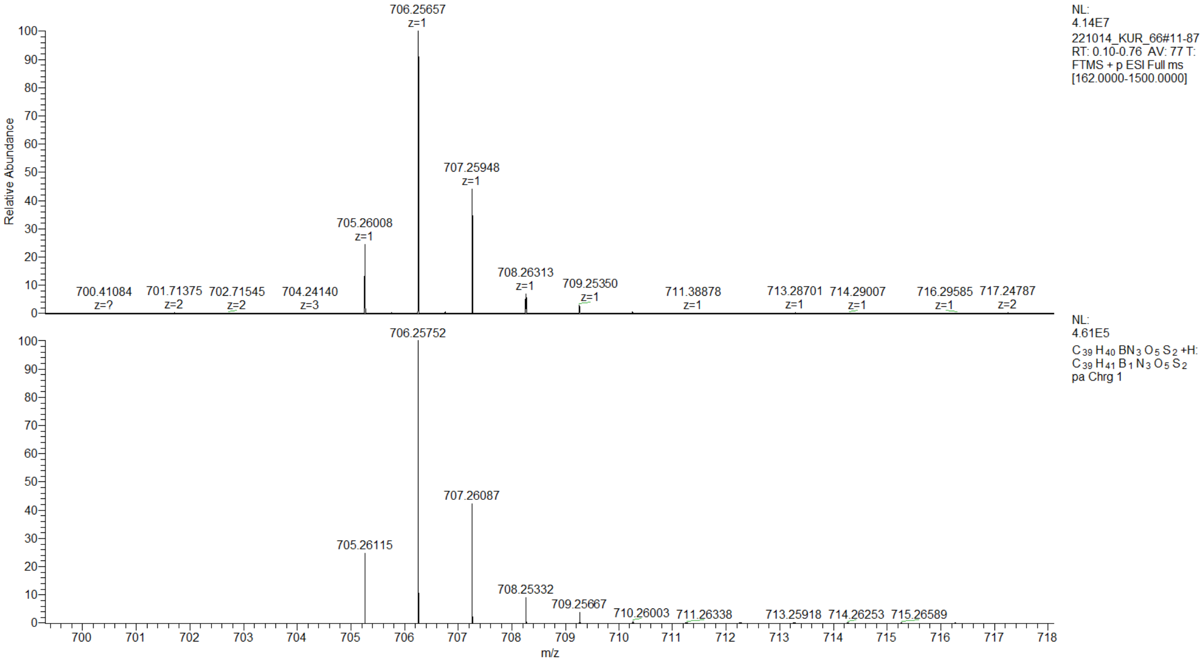

Figure S197. HR-MS (ESI) analyses of **SO2-BDP-ZWIT** with positive ion mode.

10.6 HR-MS data for X-BDP

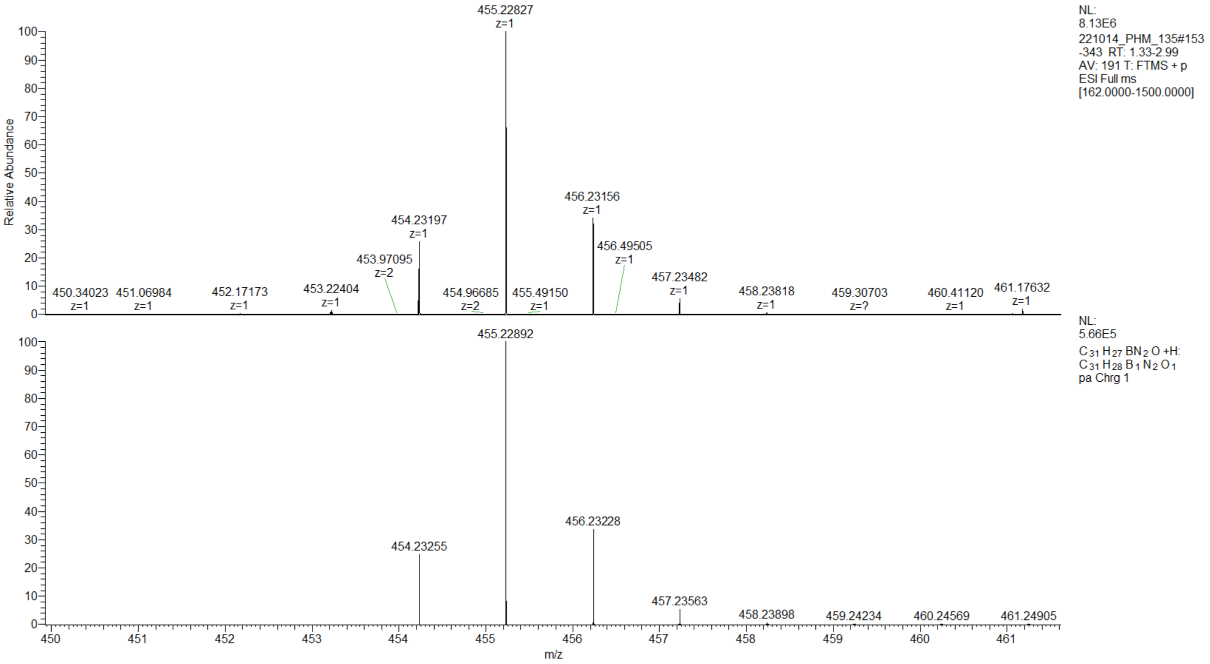

Figure S198. HR-MS (ESI) analysis of **O-BDP** with positive ion mode.

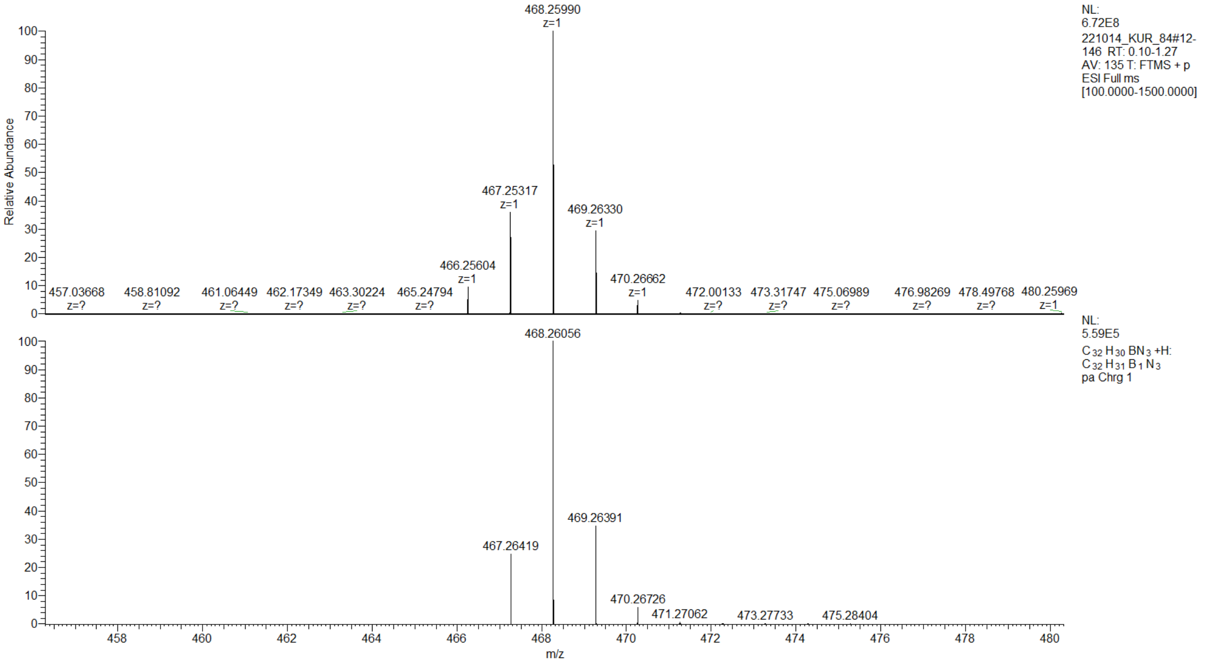

Figure S199. HR-MS (ESI) analysis of **NMe-BDP** with positive ion mode.

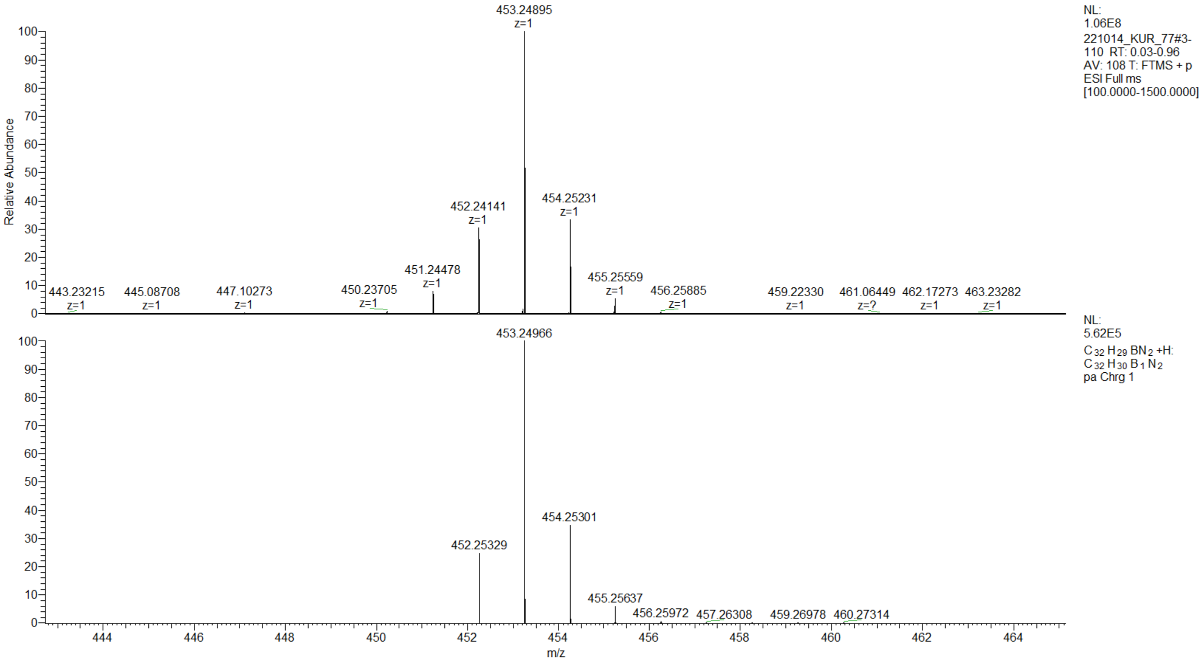

Figure S200. HR-MS (ESI) analyses of **CH2-BDP** with positive ion mode.

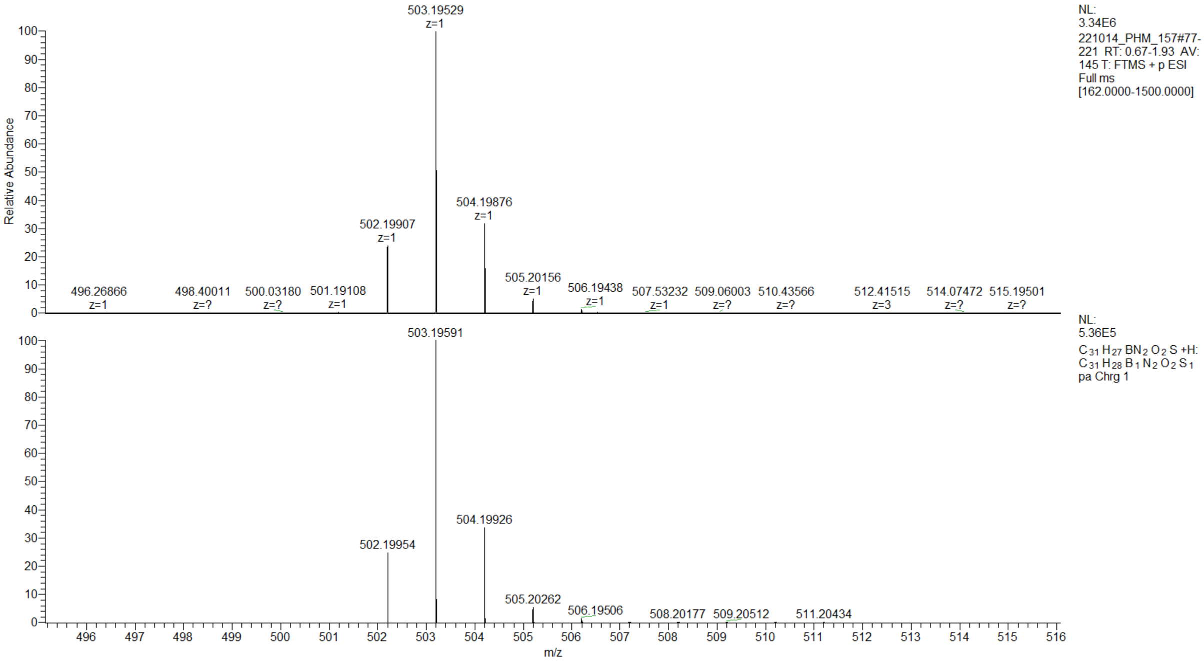

Figure S201. HR-MS (ESI) analyses of **SO2-BDP** with positive ion mode.

11. HPLC traces of target compounds

Analysis Report

Sample Information

**Sample Name**	Bf-BDP-CAT	**Data File Path**	D:\Projects\Maloczasteczkowe z LC\Data\KChF\Durka\Bf-BDP-CAT-50ppm_DAD.d
**Sample ID**		**Acq Method Path**	D:\Projects\Maloczasteczkowe z LC\Methods\Durka_pos.m
**Instrument**	LC+Revident	**Acq SW Version**	6500 series Q-TOF (12.1.98.0)
**MS Type**	QTOF (G6575A)	**Acq Operator**	SYSTEM (SYSTEM)
**MS SN / FW Ver**	SG2512Z105 / 1.1.74	**Acq Workstation**	DESKTOP-FC81A29
**Chromatogram Info**	See Instrument Info in Data File	**DA Method Path**	D:\Projects\Maloczasteczkowe z LC\Data\KChF\Durka\Bf-BDP-CAT-50ppm_DAD.d\Results\Qual\Version4\Qual_13.0_Default.m
**Inj Vol (ul)**	5	**Qualitative Analysis Version**	13.0 (13.0.384.0)
**Sample/Plate Position**	P1-B4	**DA Operator**	SYSTEM (SYSTEM)
**Barcode (Expected)**		**DA Workstation**	DESKTOP-FC81A29
**Barcode (Actual)**		**Target Source Path**	
**Barcode Status**		**IRM Status**	Success
**Acq Time (UTC)**	2026-04-23-18:34:48+02:00	**Result Summary**	

Custom Columns

Original Order	34
Randomized Order	34

Sample Chromatograms

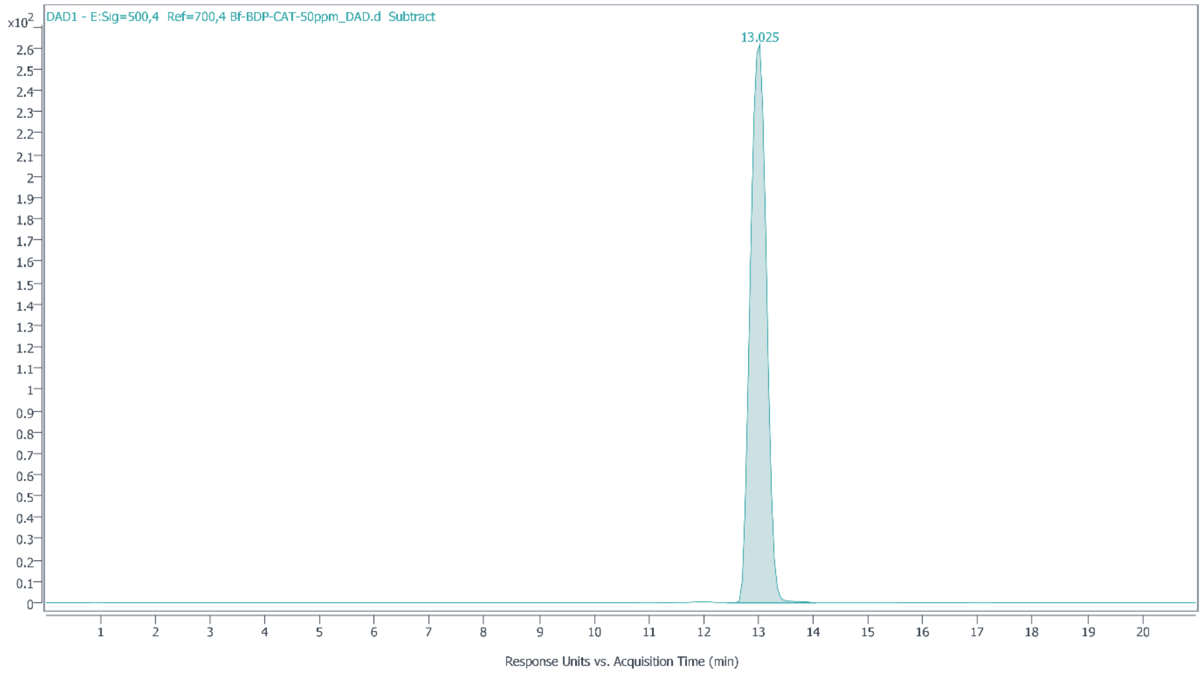

Chromatogram Peaks

Peak	Start	RT	End	Height	Area	Area %	Area Sum %
1	12.438	13.025	14.038	261.45	5369.61	100.00	100.00

Report Information

**Report Template Path**	D:\Projects\Maloczasteczkowe z LC\Report Templates\Qual 13.0\en-US_Analysis.template_2.xml.template.xml
**Report Operator**	SYSTEM (SYSTEM)
**Report Time (Local)**	2026-04-27-14:15:20+02:00

MassHunter Qual 13.0
(End of Report)

Figure S202. HPLC traces of **Bf-BDP-CAT**. Purity 100%.

Analysis Report

Agilent

Sample Information

**Sample Name**	O-BDP-CAT	**Data File Path**	D:\Projects\Maloczasteczkowe z LC\Data\KChF\Durka\O-BDP-CAT-50ppm_DAD_2.d
**Sample ID**		**Acq Method Path**	D:\Projects\Maloczasteczkowe z LC\Methods\Durka_pos.m
**Instrument**	LC+Revident	**Acq SW Version**	6500 series Q-TOF (12.1.98.0)
**MS Type**	QTOF (G6575A)	**Acq Operator**	SYSTEM (SYSTEM)
**MS SN / FW Ver**	SG2512Z105 / 1.1.74	**Acq Workstation**	DESKTOP-FC81A29
**Chromatogram Info**	See Instrument Info in Data File	**DA Method Path**	D:\Projects\Maloczasteczkowe z LC\Data\KChF\Durka\O-BDP-CAT-50ppm_DAD_2.d\Results\Qual\Version4\Qual_13.0_Default.m
**Inj Vol (ul)**	5	**Qualitative Analysis Version**	13.0 (13.0.384.0)
**Sample/Plate Position**	P1-B1	**DA Operator**	SYSTEM (SYSTEM)
**Barcode (Expected)**		**DA Workstation**	DESKTOP-FC81A29
**Barcode (Actual)**		**Target Source Path**	
**Barcode Status**		**IRM Status**	Success
**Acq Time (UTC)**	2026-04-23-15:40:43+02:00	**Result Summary**	

Custom Columns

Original Order	28
Randomized Order	28

Sample Chromatograms

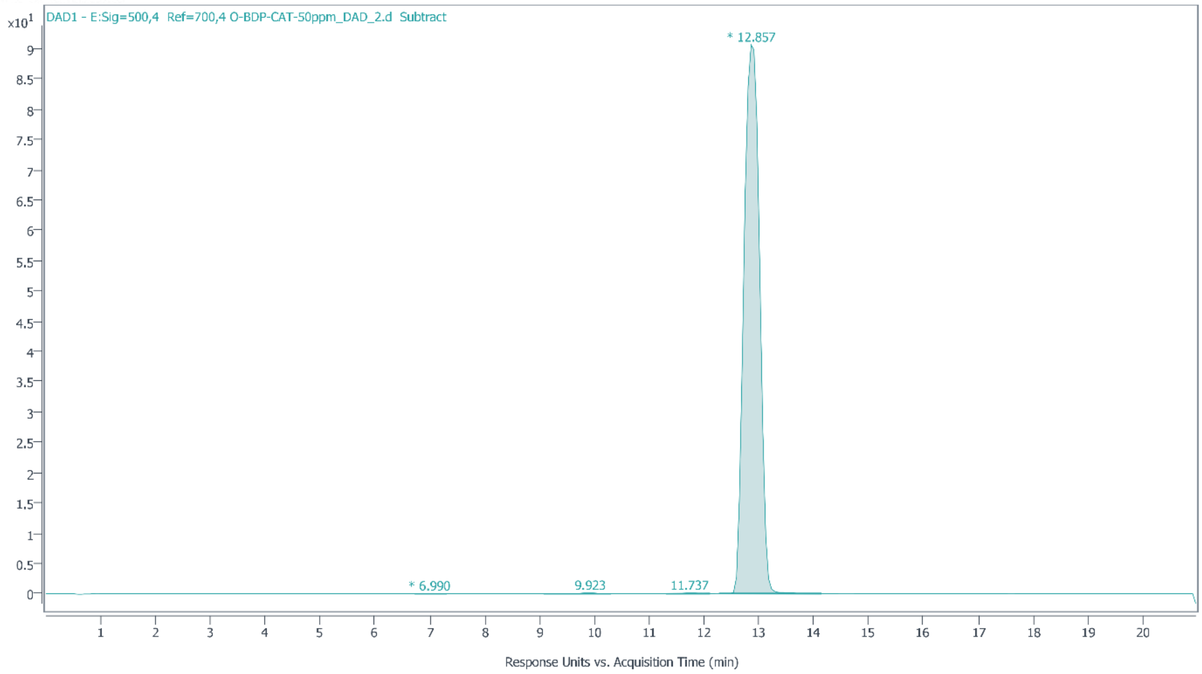

Chromatogram Peaks

Peak	Start	RT	End	Height	Area	Area %	Area Sum %
1	6.723	6.990	7.310	0.03	0.58	0.03	0.03
2	9.070	9.923	10.297	0.12	2.56	0.14	0.14
3	11.310	11.737	12.110	0.13	2.75	0.15	0.15
4	12.270	12.857	14.137	90.55	1793.08	100.00	99.67

Report Information

**Report Template Path**	D:\Projects\Maloczasteczkowe z LC\Report Templates\Qual 13.0\en-US_Analysis.template_2.xml.template.xml
**Report Operator**	SYSTEM (SYSTEM)
**Report Time (Local)**	2026-04-27-13:56:24+02:00

MassHunter Qual 13.0
(End of Report)

Figure S203. HPLC traces of **O-BDP-CAT**. Purity 99.67%.

Analysis Report

Agilent

Sample Information

**Sample Name**	NMe-BDP-CAT	**Data File Path**	D:\Projects\Maloczasteczkowe z LC\Data\KChF\Durka\NMe-BDP-CAT-50ppm_DAD_2.d
**Sample ID**		**Acq Method Path**	D:\Projects\Maloczasteczkowe z LC\Methods\Durka_pos.m
**Instrument**	LC+Revident	**Acq SW Version**	6500 series Q-TOF (12.1.98.0)
**MS Type**	QTOF (G6575A)	**Acq Operator**	SYSTEM (SYSTEM)
**MS SN / FW Ver**	SG2512Z105 / 1.1.74	**Acq Workstation**	DESKTOP-FC81A29
**Chromatogram Info**	See Instrument Info in Data File	**DA Method Path**	D:\Projects\Maloczasteczkowe z LC\Data\KChF\Durka\NMe-BDP-CAT-50ppm_DAD_2.d\Results\Qual\Version4\Qual_13.0_Default.m
**Inj Vol (ul)**	5	**Qualitative Analysis Version**	13.0 (13.0.384.0)
**Sample/Plate Position**	P1-B8	**DA Operator**	SYSTEM (SYSTEM)
**Barcode (Expected)**		**DA Workstation**	DESKTOP-FC81A29
**Barcode (Actual)**		**Target Source Path**	
**Barcode Status**		**IRM Status**	Success
**Acq Time (UTC)**	2026-04-29-12:52:21+02:00	**Result Summary**	

Custom Columns

Original Order	57
Randomized Order	57

Sample Chromatograms

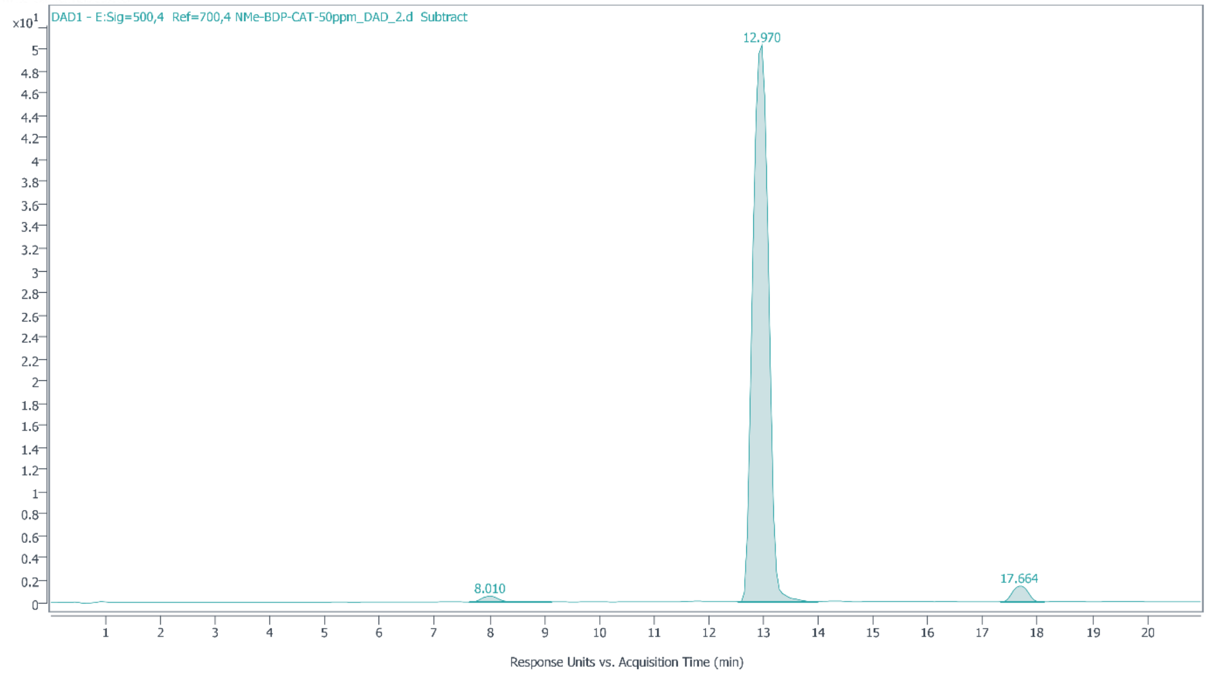

Chromatogram Peaks

Peak	Start	RT	End	Height	Area	Area %	Area Sum %
1	7.637	8.010	9.130	0.53	11.94	1.18	1.14
2	12.544	12.970	13.984	50.30	1008.15	100.00	96.20
3	17.318	17.664	18.131	1.41	27.84	2.76	2.66

Report Information

**Report Template Path**	D:\Projects\Maloczasteczkowe z LC\Report Templates\Qual 13.0\en-US_Analysis.template_2.xml.template.xml
**Report Operator**	SYSTEM (SYSTEM)
**Report Time (Local)**	2026-04-29-14:06:50+02:00

MassHunter Qual 13.0
(End of Report)

Figure S204. HPLC traces of **NMe-BDP-CAT**. Purity 96.20%.

Analysis Report

Agilent

Sample Information

**Sample Name**	CH2-BDP-CAT	**Data File Path**	D:\Projects\Maloczasteczkowe z LC\Data\KChF\Durka\CH2-BDP-CAT-50ppm_DAD_2.d
**Sample ID**		**Acq Method Path**	D:\Projects\Maloczasteczkowe z LC\Methods\Durka_pos.m
**Instrument**	LC+Revident	**Acq SW Version**	6500 series Q-TOF (12.1.98.0)
**MS Type**	QTOF (G6575A)	**Acq Operator**	SYSTEM (SYSTEM)
**MS SN / FW Ver**	SG2512Z105 / 1.1.74	**Acq Workstation**	DESKTOP-FC81A29
**Chromatogram Info**	See Instrument Info in Data File	**DA Method Path**	D:\Projects\Maloczasteczkowe z LC\Data\KChF\Durka\CH2-BDP-CAT-50ppm_DAD_2.d\Results\Qual\Version4\Qual_13.0_Default.m
**Inj Vol (ul)**	5	**Qualitative Analysis Version**	13.0 (13.0.384.0)
**Sample/Plate Position**	P1-B9	**DA Operator**	SYSTEM (SYSTEM)
**Barcode (Expected)**		**DA Workstation**	DESKTOP-FC81A29
**Barcode (Actual)**		**Target Source Path**	
**Barcode Status**		**IRM Status**	Success
**Acq Time (UTC)**	2026-04-29-13:50:23+02:00	**Result Summary**	

Custom Columns

Original Order	59
Randomized Order	59

Sample Chromatograms

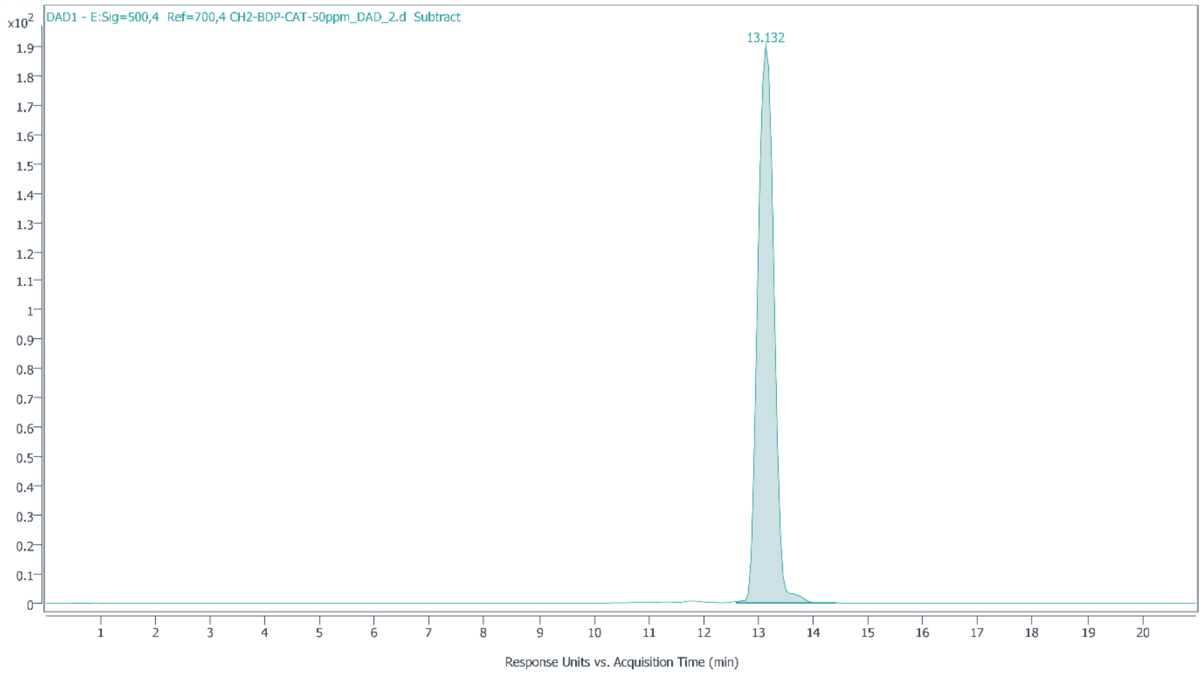

Chromatogram Peaks

Peak	Start	RT	End	Height	Area	Area %	Area Sum %
1	12.599	13.132	14.412	190.37	3929.00	100.00	100.00

Report Information

**Report Template Path**	D:\Projects\Maloczasteczkowe z LC\Report Templates\Qual 13.0\en-US_Analysis.template_2.xml.template.xml
**Report Operator**	SYSTEM (SYSTEM)
**Report Time (Local)**	2026-04-29-14:57:50+02:00

MassHunter Qual 13.0
(End of Report)

Figure S205. HPLC traces of **CH2-BDP-CAT**. Purity 100%.

Analysis Report

Sample Information

**Sample Name**	SO2-BDP-CAT	**Data File Path**	D:\Projects\Maloczasteczkowe z LC\Data\KChF\Durka\SO2-BDP-CAT-50ppm_DAD.d
**Sample ID**		**Acq Method Path**	D:\Projects\Maloczasteczkowe z LC\Methods\Durka_pos.m
**Instrument**	LC+Revident	**Acq SW Version**	6500 series Q-TOF (12.1.98.0)
**MS Type**	QTOF (G6575A)	**Acq Operator**	SYSTEM (SYSTEM)
**MS SN / FW Ver**	SG2512Z105 / 1.1.74	**Acq Workstation**	DESKTOP-FC81A29
**Chromatogram Info**	See Instrument Info in Data File	**DA Method Path**	D:\Projects\Maloczasteczkowe z LC\Data\KChF\Durka\SO2-BDP-CAT-50ppm_DAD.d\Results\Qual\Version4\Qual_13.0_Default.m
**Inj Vol (ul)**	5	**Qualitative Analysis Version**	13.0 (13.0.384.0)
**Sample/Plate Position**	P1-B2	**DA Operator**	SYSTEM (SYSTEM)
**Barcode (Expected)**		**DA Workstation**	DESKTOP-FC81A29
**Barcode (Actual)**		**Target Source Path**	
**Barcode Status**		**IRM Status**	Success
**Acq Time (UTC)**	2026-04-23-16:38:45+02:00	**Result Summary**	

Custom Columns

Original Order	30
Randomized Order	30

Sample Chromatograms

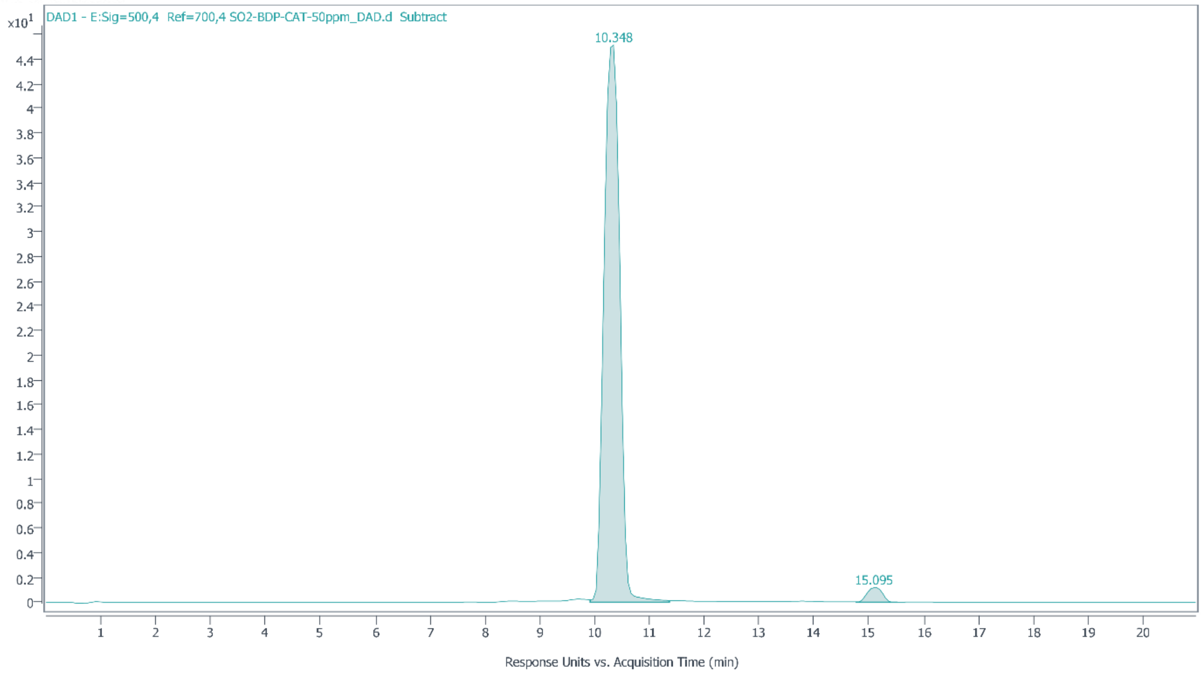

Chromatogram Peaks

Peak	Start	RT	End	Height	Area	Area %	Area Sum %
1	9.922	10.348	11.362	45.11	905.44	100.00	97.48
2	14.775	15.095	15.522	1.18	23.40	2.58	2.52

Report Information

**Report Template Path**	D:\Projects\Maloczasteczkowe z LC\Report Templates\Qual 13.0\en-US_Analysis.template_2.xml.template.xml
**Report Operator**	SYSTEM (SYSTEM)
**Report Time (Local)**	2026-04-27-13:58:41+02:00

MassHunter Qual 13.0
(End of Report)

Figure S206. HPLC traces of **SO2-BDP-CAT**. Purity 97.48%.

Analysis Report

Agilent

Sample Information

**Sample Name**	Bf-BDP-ZWIT	**Data File Path**	D:\Projects\Maloczasteczkowe z LC\Data\KChF\Durka\Bf-BDP-ZWIT-50ppm_DAD.d
**Sample ID**		**Acq Method Path**	D:\Projects\Maloczasteczkowe z LC\Methods\Durka_pos.m
**Instrument**	LC+Revident	**Acq SW Version**	6500 series Q-TOF (12.1.98.0)
**MS Type**	QTOF (G6575A)	**Acq Operator**	SYSTEM (SYSTEM)
**MS SN / FW Ver**	SG2512Z105 / 1.1.74	**Acq Workstation**	DESKTOP-FC81A29
**Chromatogram Info**	See Instrument Info in Data File	**DA Method Path**	D:\Projects\Maloczasteczkowe z LC\Data\KChF\Durka\Bf-BDP-ZWIT-50ppm_DAD.d\Results\Qual\Version4\Qual_13.0_Default.m
**Inj Vol (ul)**	5	**Qualitative Analysis Version**	13.0 (13.0.384.0)
**Sample/Plate Position**	P1-B7	**DA Operator**	SYSTEM (SYSTEM)
**Barcode (Expected)**		**DA Workstation**	DESKTOP-FC81A29
**Barcode (Actual)**		**Target Source Path**	
**Barcode Status**		**IRM Status**	Success
**Acq Time (UTC)**	2026-04-23-21:28:53+02:00	**Result Summary**	

Custom Columns

Original Order	40
Randomized Order	40

Sample Chromatograms

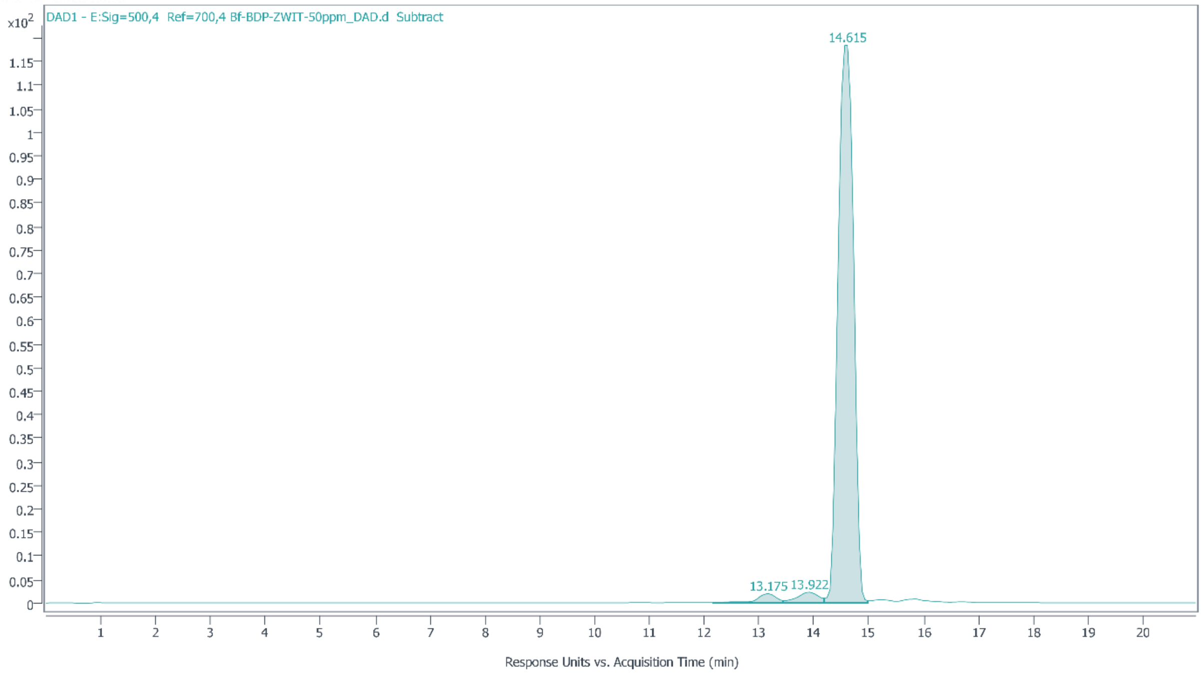

Chromatogram Peaks

Peak	Start	RT	End	Height	Area	Area %	Area Sum %
1	12.162	13.175	13.442	1.92	49.01	2.10	2.00
2	13.442	13.922	14.189	2.24	61.39	2.63	2.51
3	14.189	14.615	14.989	118.52	2336.52	100.00	95.49

Report Information

**Report Template Path**	D:\Projects\Maloczasteczkowe z LC\Report Templates\Qual 13.0\en-US_Analysis.template_2.xml.template.xml
**Report Operator**	SYSTEM (SYSTEM)
**Report Time (Local)**	2026-04-27-14:37:12+02:00

MassHunter Qual 13.0
(End of Report)

Figure S207. HPLC traces of **Bf-BDP-ZWIT**. Purity 95.49%.

12. References for Supporting Information

(1) Durka, K.; Głowacki, I.; Luliński, S.; Łuszczyńska, B.; Smętek, J.; Szczepanik, P.; Serwatowski, J.; Wawrzyniak, U. E.; Wesela-Bauman, G.; Witkowska, E.; Wiosna-Sałyga, G.; Woźniak, K. Efficient 8-Oxyquinolinato Emitters Based on a 9,10-Dihydro-9,10-Diboraanthracene Scaffold for Applications in Optoelectronic Devices. *J. Mater. Chem. C* **2015**, *3*, 1354–1364.

(2) Dimitrijević, E.; Cusimano, M.; Taylor, M. S. Synthesis of Benzannulated Heterocycles by Twofold Suzuki–Miyaura Couplings of Cyclic Diarylborinic Acids. *Org. Biomol. Chem.* **2014**, *12*, 1391–1394.

(3) Cheung, K. Y.; Miao, Q. A Ketone-Functionalized Aromatic Saddle as a Potential Building Block for Negatively Curved Carbon Nanobelts. *Chin. Chem. Lett.* **2019**, *30*, 1506–1508.

(4) Urban, M.; Marek-Urban, P. H.; Durka, K.; Luliński, S.; Pander, P.; Monkman, A. P.; TADF Invariant of Host Polarity and Ultralong Fluorescence Lifetimes in a Donor-Acceptor Emitter Featuring a Hybrid Sulfone-Triarylboron Acceptor. *Angew. Chem. Int. Ed.* **2023**, *62*, e202217530.

(5) Marek-Urban, P. H.; Urban, M.; Wiklińska, M.; Paplińska, K.; Woźniak, K.; Blacha-Grzechnik, A.; Durka, K. Heavy-Atom Free Spiro Organoboron Complexes As Triplet Excited States Photosensitizers for Singlet Oxygen Activation. *J. Org. Chem.* **2021**, *86*, 12714–12722.

(6) Yuan, K.; Wang, X.; Mellerup, S. K.; Kozin, I.; Wang, S. Spiro-BODIPYs with a Diaryl Chelate: Impact on Aggregation and Luminescence. *J. Org. Chem.* **2017**, *82*, 13481–13487.

(7) Uddin, A.; Allen, S. R.; Rylski, A. K.; O'Dea, C. J.; Ly, J. T.; Grusenmeyer, T. A.; Roberts, S. T.; Page, Z. A. Do The Twist: Efficient Heavy-Atom-Free Visible Light Polymerization Facilitated by Spin-Orbit Charge Transfer Inter-System Crossing. *Angew. Chem. Int. Ed.* **2023**, *62*, e202219140.

(8) Chi, W.; Chen, J.; Liu, W.; Wang, C.; Qi, Q.; Qiao, Q.; Tan, T. M.; Xiong, K.; Liu, X.; Kang, K.; Chang, Y.-T.; Xu, Z.; Liu, X. A General Descriptor ΔE Enables the Quantitative Development of Luminescent Materials Based on Photoinduced Electron Transfer. *J. Am. Chem. Soc.* **2020**, *142*, 6777–6785.

(9) Chansaenpak, K.; Wang, H.; Wang, M.; Giglio, B.; Ma, X.; Yuan, H.; Hu, S.; Wu, Z.; Li, Z. Synthesis and Evaluation of [18F]-Ammonium BODIPY Dyes as Potential Positron Emission Tomography Agents for Myocardial Perfusion Imaging. *Chem. Europ. J.* **2016**, *22*, 12122–12129.

(10) Lama, A. D.; Sestelo, J. P.; Sarandeses, L. A.; Martínez, M. M. Microwave-Assisted Direct Synthesis of BODIPY Dyes and Derivatives. *Org. Biomol. Chem.* **2022**, *20*, 9132–9137.
